# Regioselective Synthesis of Ambipolar B–N Lewis Pair Functionalized Pyrenes: Structural Dynamics, Emission Tuning, and Applications in Live Cell Imaging and as Electrochemiluminescent Materials

**DOI:** 10.1021/jacs.5c22679

**Published:** 2026-03-09

**Authors:** Mukundam Vanga, Sara Jahanghiri, Justin Davis, Rajendra Prasad Nandi, Ashutosh Sahoo, Pavel Kucheryavy, Roger A. Lalancette, Edward M. Bonder, Zhifeng Ding, Frieder Jäkle

**Affiliations:** † Department of Chemistry, 67206 Rutgers University − Newark, 73 Warren Street, Newark, New Jersey 07102, United States; ‡ Department of Chemistry, University of Western Ontario, London, ON N6A 5B7, Canada; § Department of Biological Sciences, Rutgers University − Newark, 195 University Avenue, Newark, New Jersey 07102, United States

## Abstract

Intramolecular B←N Lewis pair functionalization of polycyclic aromatic hydrocarbons (PAHs) is emerging as a powerful strategy for tailoring their optical and electronic properties to specific applications. Herein, we report the design and synthesis of four quadrupolar pyrene fluorophores containing triarylamine donor and pyridyl-borane acceptor substituents by N-directed electrophilic C–H borylation in the K-region. Introduction of rigid six-membered B–N heterocycles greatly decreased the lowest unoccupied molecular orbital (LUMO) energy levels and red-shifted the absorption and emission maxima compared to the nonborylated precursors. These pyrenes exhibit absorptions spanning the entire visible region and intense green to deep red and near-infrared fluorescence, with impressive quantum yields as high as 82%. The simultaneous presence of pyridyl-borane acceptor and arylamine donor units also enables facile reversible multiredox oxidation and reduction processes. The advantageous photophysical and electrochemical characteristics are highly attractive for optoelectronic devices and (bio)­imaging applications. The pyrene derivative with the most red-shifted emission, **5-Pf**, was further evaluated in live cell imaging studies, showing effective staining of acidic compartments, including lysosomes. In addition, we demonstrate the outstanding performance of **5-Pf** as a novel electrochemiluminescent (ECL) luminophore through annihilation and coreactant experiments in cyclic voltammetry and pulsing modes. Spooling ECL spectroscopy revealed a strong emission with a maximum at 646 nm, in good agreement with the photoinduced emission maximum at 660 nm. A superior absolute ECL efficiency (Φ_ECL_) of up to 0.9 ± 0.1%, exceeding that of typically employed [Ru­(bpy)_3_]^2+^, was achieved during CV scanning with benzoyl peroxide as a coreactant.

## Graphical abstract

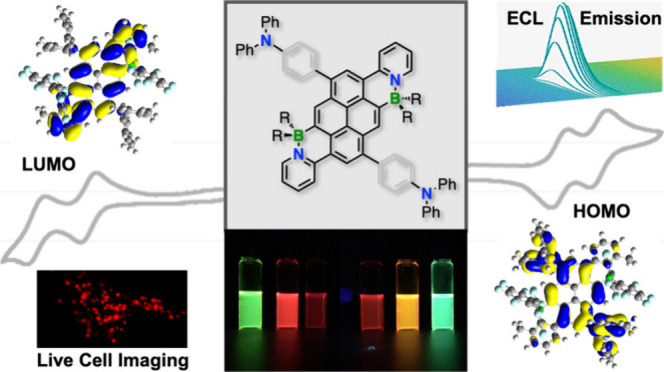

## Introduction

The development of organic chromophores based on polycyclic aromatic hydrocarbons (PAHs) has gained widespread interest.^1^ Among them, pyrene and its derivatives have garnered considerable attention because of their advantageous photophysical properties including high fluorescence quantum yield, exceptionally long-lived singlet excited state, excimer and exciplex formation, and excellent thermal and photochemical stability. Therefore, pyrene derivatives have been employed as fluorescence probes for biomedical applications, as components in optoelectronic devices, and as scaffolds for the development of new fluorescent sensors.^2^ Further progress relies on new methods to introduce diverse functional groups to the pyrene framework. The selective placement of substituents on pyrene is essential for fine-tuning the electronic structure to attain desirable properties for specific applications.

The photophysical and electronic properties of PAHs are often modulated by introducing electron donor (D) or acceptor (A) moieties of different strength at their periphery, thereby influencing the highest occupied molecular orbital (HOMO) and LUMO levels. Salient properties such as a permanent dipole moment, strong solvatochromism, environmentally influenced photophysics, narrowed energy gaps, and the possibility for energy or electron transfer have triggered intense interest in D–A PAHs.^3^ The substitution of pyrene with borane acceptor groups at predetermined positions can bring about interesting optoelectronic properties that originate from intramolecular charge transfer (ICT) processes. The introduction of electron-deficient BMes_2_ groups (*tricoordinate boron*) at different positions of pyrene has been widely studied (**A**-**C**, Figure 1), and corresponding D/A compounds with arylamine donors (**D**-**E**, Figure 1)^3b,4^ have been reported independently by Marder and Chujo.^4,5^ On the other hand, we^6,7^ and others^8^ have demonstrated that functionalization of PAHs with intramolecular B←N Lewis pairs (*tetracoordinate boron*) generates rigidified π-extended structures with strong polarization, resulting in red-shifted absorption and emission maxima, intensified luminescence, and enhanced intermolecular π-π interactions. Moreover, B←N Lewis pair functionalization often increases the electron accepting strength of conjugated materials because the LUMO levels are lowered. Taking advantage of these desirable photophysical and electronic characteristics, C,N-chelate complexes of boron have been utilized in organic light-emitting devices (OLEDs), organic photovoltaics (OPVs), and organic field-effect transistors (OFETs), and have also garnered interest as singlet oxygen sensitizers, photochromic, molecular switching, sensory materials, and in photocatalysis.^9^ However, applications in bioimaging and therapeutic fields are only now emerging,^7a,10^ with further advances requiring the design of new robust and tunable chromophores with superior absorption and emission characteristics.

**Figure 1:**
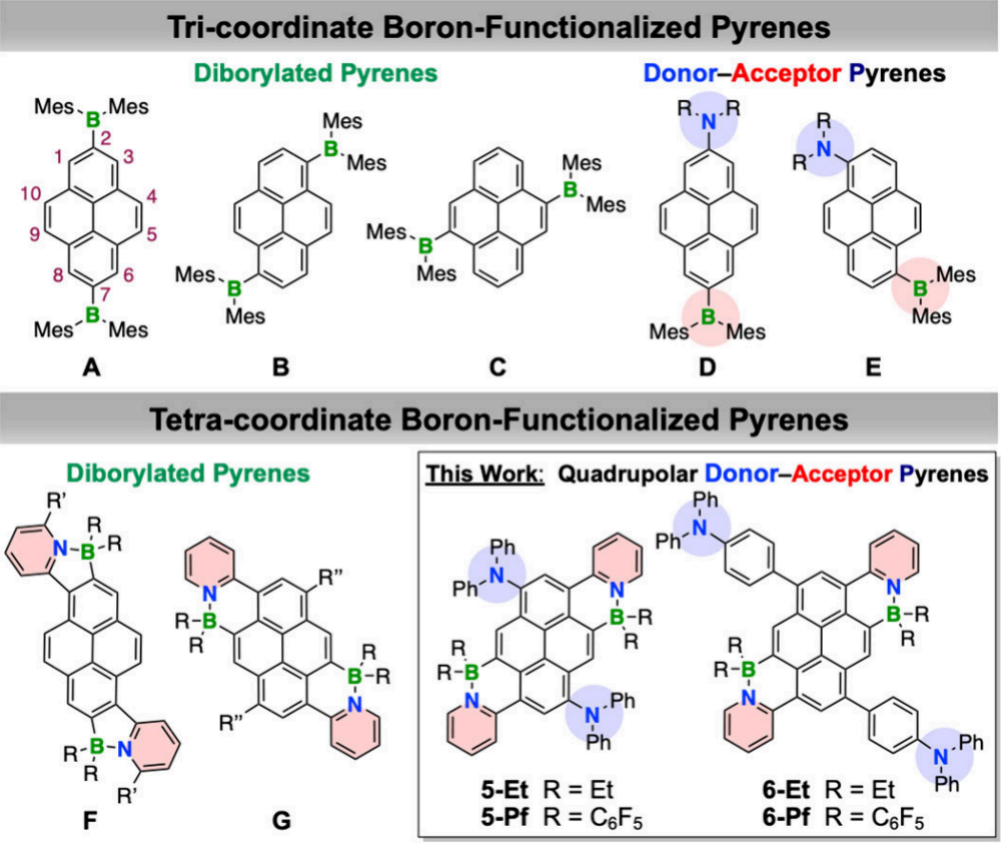
Representative structures of boron-functionalized pyrenes and new donor–acceptor systems reported here (for **D** and **E**: R = *p*-C_6_H_4_OMe, for **F** and **G**: R = Et, Ph and R′ = C_13_H_27_, R′′ = C_8_H_17_; Mes = 2,4,6-trimethylphenyl)

Previously, we demonstrated that N-directed electrophilic C–H borylation^11^ of 1,6-dipyridylpyrene occurs in the 2,7-positions with generation of 5-membered B–N heterocycles (**F**) whereas that of 3,8-dialkylated 1,6-dipyridylpyrene results in selective borylation in the K-region with formation of 6-membered B–N heterocycles (**G**).^12^ Introduction of B←N Lewis pairs in the K-region of pyrene leads to chromophores with lower LUMO levels and red-shifted absorption and emission maxima in comparison to pyrenes with boryl groups attached in the 2,7-positions (a nodal plane passes through the 2,7-positions in both the HOMO and LUMO limiting orbital overlap).^3b,5b,5c^ In pursuit of pyrenes that show further enhanced emission at even longer wavelengths reaching into the near-infrared (NIR),^2h,13^ we designed a new class of D–A pyrene derivatives that feature diphenylamino or triphenylamine donor moieties in the 1,6-positions in addition to pyridyl acceptor moieties in the 3,8-positions. The acceptor strength of the pyridyl groups is greatly enhanced by N-directed electrophilic borylation which generates quadrupolar B←N D–A pyrene derivatives **5** and **6** (Figure 1) that exhibit strong emission with maxima up to 660 nm and high quantum yields of up to 82%, attributes that are very attractive for optoelectronic device and (bio)­imaging applications. We provide detailed insights into the electronic structures and photophysical properties of these novel dyes and present preliminary live cell imaging studies for pyrene derivative **5-Pf** which shows the most red-shifted emission. In addition, we explore the performance of **5-Pf** as a novel electrochemiluminescent (ECL) luminophore through annihilation and coreactant pathways. ECL techniques have revolutionized fields such as immunoassays, ultrasensitive biological analysis, environmental monitoring, and single-photon-level imaging of tissues and cells.^14^ Despite these advancements, the development of high-quality ECL luminophores remains a significant challenge.^15^

## Results and Discussion

### Synthetic Approach

To ensure favorable solubility of the final products in organic solvents, we introduced pyridyl functional groups on pyrene with long alkyl substituents at the 2-position. 1,6-bis­(6-tridecylpyrid-2-yl)­pyrene (**2**) was obtained as a pale-yellow solid in 67% yield by Suzuki–Myaura coupling of 1,6-bis­(pinacolatoboryl)­pyrene (**1**) with 2-bromo-6-tridecylpyridine and then treated with 2.2 equiv of NBS to afford 6,6′-(3,8-dibromopyrene-1,6-diyl)­bis­(2-tridecylpyridine) (**3**) in 86% yield (Scheme S1 (SI)). The latter was subjected to Buchwald–Hartwig coupling with bis­(4-(*tert*-butyl)­phenyl)­amine to give ligand **L1** as a yellow solid in 79% yield (Scheme 1). Ligand **L2** was obtained as a yellow solid in 75% yield by Suzuki–Miyaura cross-coupling reaction of **3** with *N,N*-bis­(4-(*tert*-butyl)*-N*-(4-(4,4,5,5-tetramethyl-1,3,2-dioxaborolan-2-yl)­phenyl)­amine (**4**). **L1** and **L2** were isolated by recrystallization from a mixture of chloroform and methanol and their structures verified by ^1^H, ^13^C, H,H–COSY NMR, and high-resolution MS analysis.

**Scheme 1:**
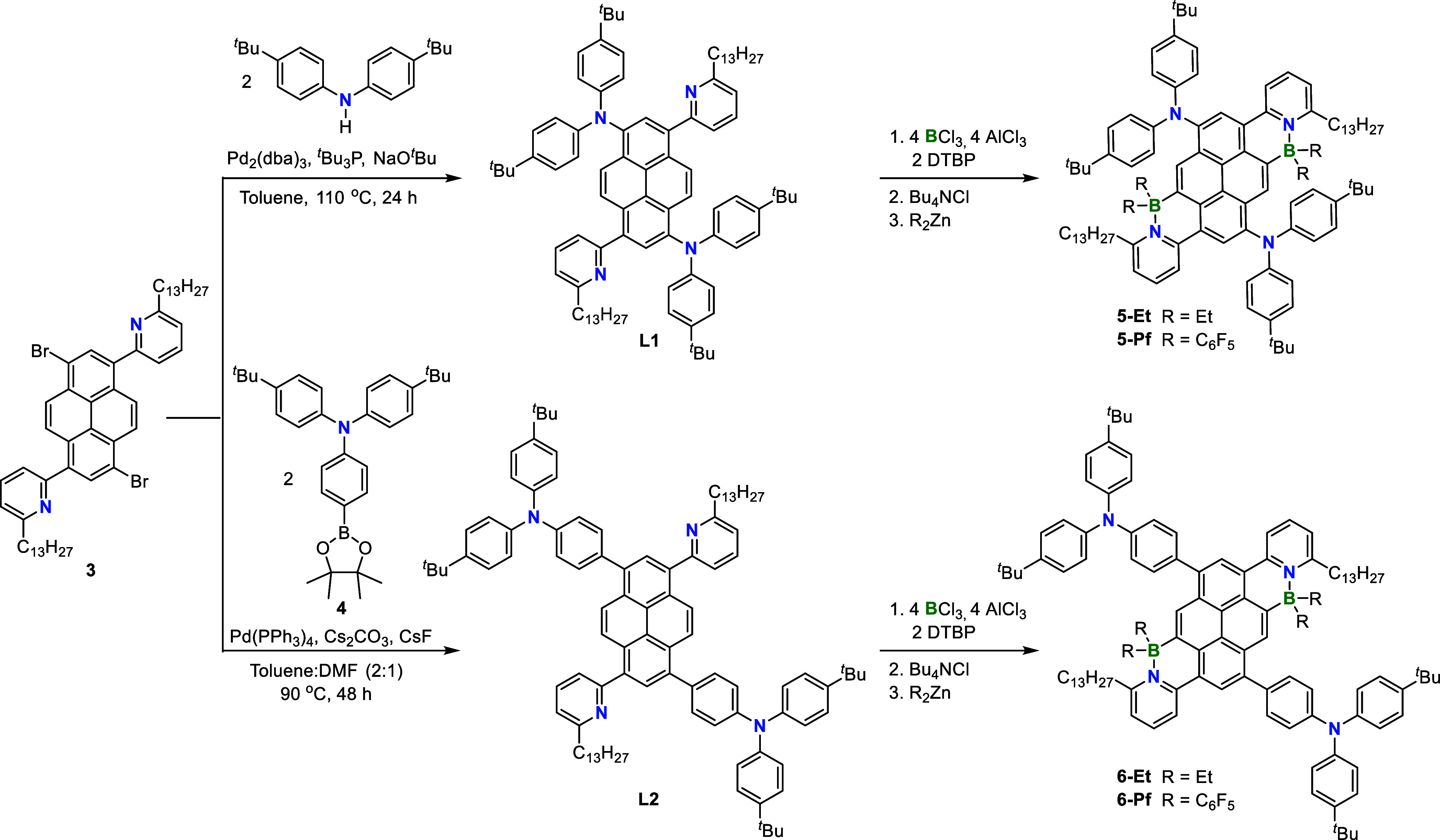
Synthesis of BN LP-Functionalized Donor–Acceptor Pyrenes

Regioselective N-directed C–H borylation^11a^ of **L1** and **L2** respectively was accomplished in anhydrous CH_2_Cl_2_ by treatment with BCl_3_ (4 equiv), AlCl_3_ (4 equiv), and 2,6-di-*tert*-butylpyridine as a base (2 equiv), followed by quenching of the borenium ion intermediates by addition of Bu_4_NCl (2 equiv) to generate the BCl_2_-substituted species (**5-Cl** and **6-Cl**). To convert **5-Cl** and **6-Cl** to the ethyl derivatives, Et_2_Zn (4 equiv) was added neat and the mixture stirred for 16 h at room temperature. Filtration through a small plug of silica gel, removal of the solvent under reduced pressure, and washing of the residue, first with methanol and then cold pentane, gave **5-Et** as a red solid (83%) and **6-Et** as an orange-red solid (84%). To generate the respective pentafluorophenyl-substituted derivatives (**5-Pf**, **6-Pf**) the solvent was removed from **5-Cl** and **6-Cl** under reduced pressure, and the resultant residue suspended in anhydrous toluene. Bis­(pentafluorophenyl)­zinc was then added, the mixture stirred for 24 h at room temperature, and stirring was then continued for a further 2 h at 40 °C. The products were purified by column chromatography on base-treated silica gel using hexanes and ethyl acetate as eluent (9:2) and isolated as dark red (**5-Pf**, 50%) and red (**6-Pf**, 61%) solids, respectively.

The structures were confirmed by multinuclear NMR and high-resolution MS analysis, and in the case of **5-Et** and **6-Et** H,H–COSY and HSQC 2D NMR spectra were also acquired. The ^11^B NMR spectra showed a single resonance in CDCl_3_ in the region typical for tetracoordinate boron, but slightly more shielded for **5-Pf** and **6-Pf** (−4.6, −4.7 ppm) than **5-Et** and **6-Et** (3.3, 2.9 ppm), possibly indicating a stronger B–N interaction in the presence of the electron-withdrawing C_6_F_5_ groups. The typical pattern of 3 resonances for the *ortho*- (−131.0 ppm), *para*- (−157.5 ppm), and *meta*-fluorines (−163.4/–163.8 ppm) is seen in the ^19^F NMR spectra of **5-Pf** and **6-Pf**. However, these signals are slightly broadened at RT, indicating a dynamic process. Indeed, variable temperature (VT) NMR studies in CD_2_Cl_2_ revealed further broadening with decreasing temperature, followed by splitting of the *ortho*-fluorines into multiple signals at ca. −40 °C (**5-Pf**) and −60 °C (**6-Pf**) respectively. This suggests that the diastereotopic C_6_F_5_ groups become inequivalent in the slow exchange regime at low temperature as discussed in more detail later. In contrast to the free ligands **L1** and **L2**, which give rise to two characteristic doublets and a singlet for the pyrene moiety in the downfield region of the ^1^H NMR spectra (**L1**, δ (ppm) = 8.26 (d), 8.17 (d), 7.96 (s)); **L2**, δ (ppm) = 8.33 (d), 8.30 (d), 8.16 (s)), the boron complexes show only two singlets in the range from 8.18–8.48 ppm. This confirms the regioselectivity for borylation in the K-region rather than the 2,7-positions of pyrene,^5f,12,16^ the latter being sterically shielded by the diphenylamino or triphenylamine groups.

### X-ray Structure Analyses

The regioselective borylation in the K-region was further confirmed by X-ray structure analyses of **5-Et** and **5-Pf** (Figure 2). Crystals of **5-Et** were grown by slow evaporation of a solution in CHCl_3_, and those of **5-Pf** by evaporation of a solution in a mixture of THF and acetonitrile. Both compounds crystallized in the triclinic *P* 1 space group, showing a center of inversion at the pyrene core. The structure of **5-Pf** was twinned and showed some disorder in the *tert*-butylphenyl and tridecyl substituents leading to relatively large standard deviations on the metric parameters (see Figure S35). The B–N distances of 1.682(3) Å for **5-Et** and 1.648(7) Å for **5-Pf** are in the typical range for these types of C,N-chelate complexes (Table 1); they are slightly longer than those for related complexes with nonalkylated pyridyl rings,^12^ which is consistent with the moderate steric strain exerted by the 2-alkyl groups. The B–C bonds to pyrene (1.619(3), 1.620(9) Å) are slightly shorter than those to the ethyl (1.629(3), 1.630(4) Å) and C_6_F_5_ (1.643(9), 1.650(9) Å) groups. The boron atoms are found in a distorted tetrahedral environment with the most notable deviation seen for the large C_Et_–B–C_Et_ angle in **5-Et** (115.1(2)°) and even larger C_Pf_–B–C_Pf_ angle in **5-Pf** (117.5(5)°); the latter is counterbalanced by relatively smaller C_pyr_–B–C_Pf_ (105.4(4), 109.2(5)°) and N1–B1–C_Pf_ (105.6(4), 109.8(5)°) angles. The arylamine moieties are oriented at large angles relative to the pyrene plane (**5-Et** 58.5°, **5-Pf** 79.2°) because of the close proximity to and steric demands of the neighboring boryl groups. The pyridyl moieties are rotated relative to the pyrene backbone by 25.3° for **5-Et** and 26.9° for **5-Pf**. As a result, the boron atoms are oriented in a *trans*-arrangement with one boron positioned above and one below the pyrene plane by 0.351 Å (**5-Et**) and 0.472 Å (**5-Pf**) respectively. As such, the ethyl/C_6_F_5_ groups adopt axial and equatorial positions respectively relative to the distorted six-membered B–N heterocycles. As discussed further in the following section, it is this unsymmetric arrangement of the R groups that makes them inequivalent in the static structure and leads to signal splitting at low temperature in the ^19^F NMR spectra of **5-Pf** and **6-Pf** when inversion of the B–N heterocycles becomes slow on the NMR time scale. This effect is similar to observations made for B–N Lewis pair-functionalized anthracenes, for which interconversion was proposed to involve B–N bond cleavage followed by rotation of the pyridyl ring.^6^ Related processes have also been seen for bowl-shaped quasi[7]­circulenes derived from borylation of aza[5]­helicenes.^9d^

**Figure 2:**
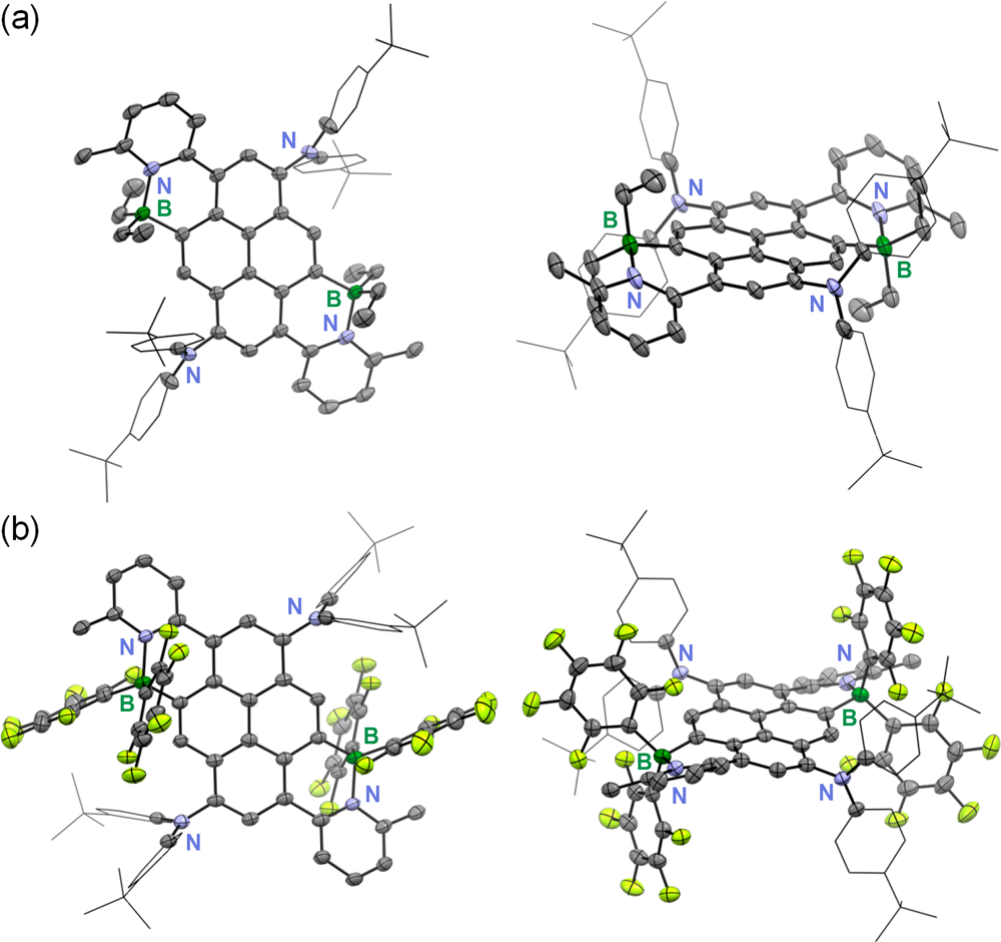
Top and side views of the X-ray crystal structures of (a) **5-Et** and (b) **5-Pf** (B green, N blue, F yellow; thermal ellipsoid at 50% probability; hydrogen atoms and a disordered THF molecule in the crystal of **5-Pf** are omitted for clarity); for the tridecyl chains only the first methylene unit is shown and *tert*-butylphenyl groups are displayed as wireframe to more clearly illustrate the arrangement of the B atoms, pyridyl and ethyl/C_6_F_5_ groups relative to pyrene. Complete structures are displayed in Figure S35 (SI).

**Table 1:** Geometric Parameters (Distances in Å, Angles in °) of B–N Lewis Pair Complexes Obtained from X-ray Crystal Structure Analyses and DFT Calculations (Gaussian 16; RB3LYP/6-31G­(d))

	B–N	B–C_Pyr_ ^a^	B–C_R_ ^a^	C_R_–B–C_R_ ^a^	C_R_–B–N^a^	C_Pyr_–B–N^a^	C_Pyr_–B–C_R_ ^a^	B···Pyr^b^	Pyr//Py^c^
**5-Et** CCDC 2512851	1.682(3)	1.619(3)	1.629(3)	115.1(2)	109.0(2)	107.8(2)	107.5(2)	0.35	25.3
			1.630(4)		109.4(2)		107.8(2)		
**5-Me′**	1.694	1.622	1.641	115.6	107.3	108.1	106.4	0.38	21.6
			1.641		108.8		110.4		
**5-Pf** CCDC 2516943	1.648(7)	1.620(9)	1.643(9)	117.5(5)	105.6(4)	109.3(5)	105.4(4)	0.47	26.9
			1.650(9)		109.8(5)		109.2(5)		
**5-Pf′**	1.646	1.624	1.649	117.6	107.3	109.2	106.1	0.48	26.2
			1.663		108.8		107.7		
**6-Me′** ^d^	1.691	1.621	1.641	115.5	105.3 110.8	108.1	106.6	0.38	21.5
			1.642		110.8		110.2		
	1.692	1.618	1.641	115.8	107.3	108.6	107.7	0.21	20.7
			1.644		108.9		108.5		
**6-Pf′**	1.651	1.627	1.653	117.4	106.4	109.3	106.4	0.47	26.5
			1.660		108.8		108.5		

a C_Pyr_ refers to boron-bound pyrene carbon; C_R_ refers to boron-bound carbon of R groups.

b Displacement of B from pyrene plane.

c Interplanar angle between pyrene and pendent pyridyl ring.

d For **6-Me′** the reported unsymmetric structure was relatively lower in energy than the respective inversion-symmetric conformer.

### Structural Dynamics in Solution

Variable temperature (VT) NMR data offer further insights into the conformational flexibility and preferred orientation of the molecules in the solution state. Cooling of solutions of **5-Pf** or **6-Pf** in CD_2_Cl_2_ led to gradual broadening and ultimately splitting of the ^19^F NMR signals; the corresponding spectral data for **5-Pf** are presented in Figure 3a and those for **6-Pf** in Figure S40 (SI). Based on low temperature ^19^F,^19^F-COSY spectral data, acquired at −110 °C and illustrated in Figure 3b, the four major signals in the range from −124 to −142 ppm can be attributed to *ortho*-fluorines, two signals from −156 to −160 ppm to *para*-fluorines, and four signals from −161 to −165 ppm to *meta*-fluorines on the boron-bound C_6_F_5_ rings. This indicates that the two C_6_F_5_ groups on each boron center are not only chemically inequivalent because of the nonplanarity of the B–N heterocycles, but the *ortho*- and *meta*-fluorines also show distinct signals because of hindered rotation about the B–C_6_F_5_ bonds. The signals for the pairs of *ortho*- and *meta*-fluorines merge simultaneously when the temperature reaches ca. −40 °C, making it difficult to deduce thermodynamic data through analysis of the exchange processes. Fortunately, for the *para*-fluorines only the exchange between C_6_F_5_ groups is detected. Line shape analysis of the *para*-fluorine signals in the range from −65 to −30 °C (red box in Figure 3a) was performed, and an Eyring plot was generated as described in detail in the SI. The data analysis provided an estimate of the thermodynamic parameters for the exchange process in **5-Pf** of Δ*H* ^‡^ = 26.9 kJ mol^–1^, Δ*S* ^‡^ = −48.6 J (mol K)^−1^, and Δ*G* ^‡^_298_ = 41.4 kJ mol^–1^ (Figure S41c, Table S1b; SI). Variable temperature ^1^H NMR data revealed a similar process in which the *ortho*- and *meta*-phenyl protons in the NPh_2_ groups merged once the temperature reached about −75 °C. The thermodynamic parameters for this exchange process were determined to Δ*H* ^‡^ = 27.1 kJ mol^–1^, Δ*S* ^‡^ = −56.8 J (mol K)^−1^, and Δ*G* ^‡^_298_ = 44.0 kJ mol^–1^ (Figure S41a,b, Table S1a; SI). They are very similar to those derived from the ^19^F NMR spectra, suggesting that the ring-flip of the B–N heterocycle with reversal of the orientation of the C_6_F_5_ groups directly impacts the neighboring NPh_2_ groups that are for steric reasons also oriented perpendicular to the pyrene plane (see structures in Figure 2). The negative entropy value is indicative of a more rigid structure in the corresponding transition state. We postulate that the barrier to inversion of the 6-membered B–N heterocycles, which leads to a switch in the orientation (equatorial/axial) of the C_6_F_5_ groups on boron and the Ph rings on nitrogen is responsible for this process. Similar phenomena were also observed for **6-Pf**, but in this case the additional phenylene linker between the pyrene core and NPh_2_ groups lowered the coalescence temperature for the *para*-fluorines to *T*_c_ = −68 °C (Figure S40; SI) and prevented observation of coalescence phenomena in the ^1^H NMR spectra (Figure S39; SI). An Eyring analysis of the ^19^F VT NMR data (Figure S41d, Table S1c; SI) revealed a relatively lower barrier of Δ*G* ^‡^_298_ = 26.3 kJ mol^–1^ because of a far larger (positive) entropy term (Δ*S* ^‡^ = 89.2 J­(mol K)^−1^) that outweighed a relatively larger enthalpic barrier (Δ*H* ^‡^ = 52.9 kJ mol^–1^), suggesting a more flexible transition state geometry. The finding that the energy barrier is relatively low and the positive entropy term poses the question whether the exchange mechanism of **6-Pf** may involve a B–N bond dissociation process, a possibility that had previously been considered also for B–N Lewis pair-functionalized anthracenes.^6^ Furthermore, related work on the bowl-to-bowl inversion of B–N bridged helicenes suggested that either direct inversion or a dissociative pathway is feasible.^9d^ Whereas π-donating diol substituents in the latter case are expected to lower the B–N dissociation energies, especially in comparison to C_6_F_5_ groups present in **5-Pf** and **6-Pf**, the additional steric strain exerted by alkylation of **5-Pf** and **6-Pf** in the *ortho*-pyridyl positions may also lower the free energy barrier of such a process.

**Figure 3:**
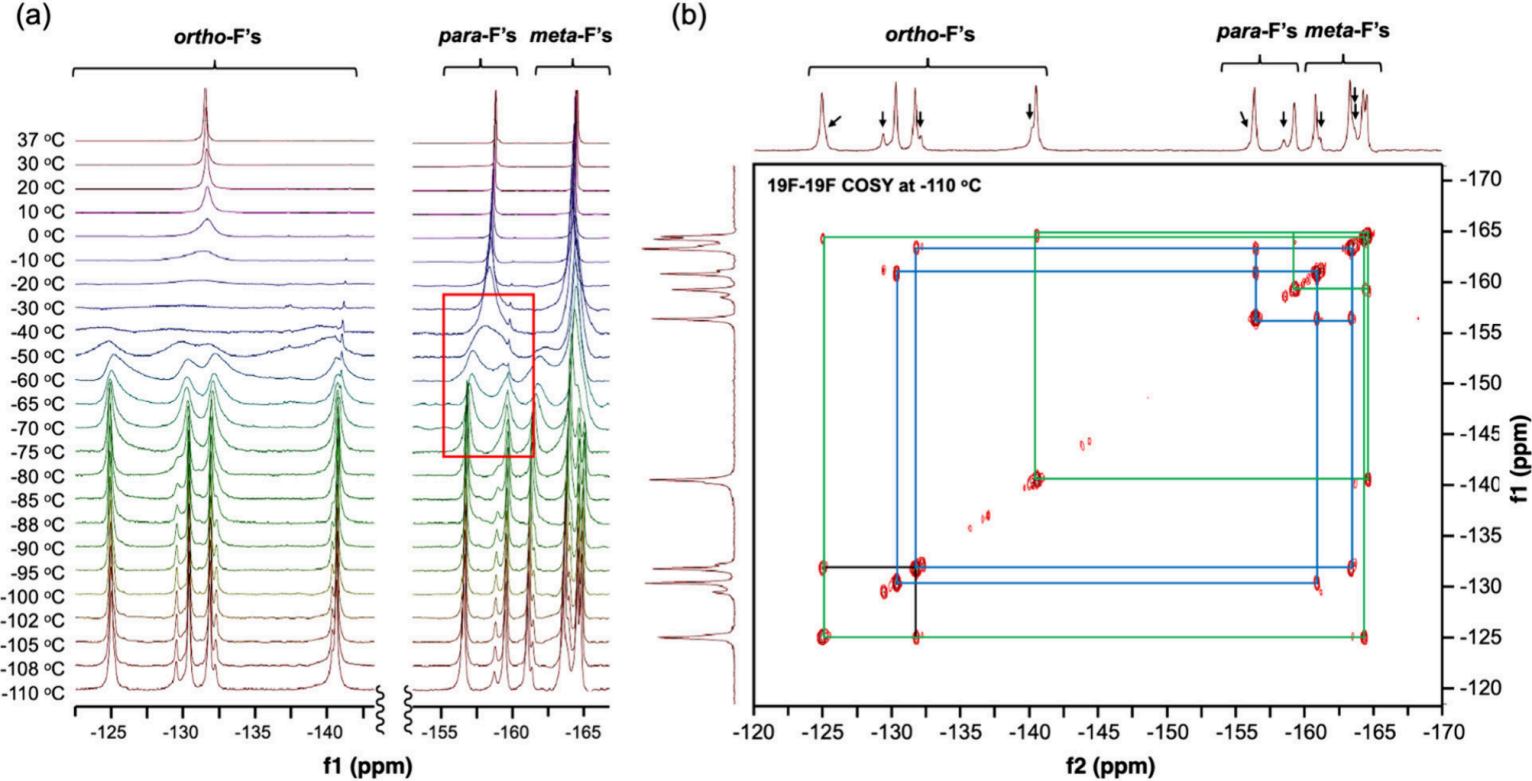
(a) ^19^F NMR spectra of **5-Pf** in CD_2_Cl_2_ at variable temperatures; (b) ^19^F,^19^F-COSY NMR spectrum of **5-Pf** at −110 °C (arrows indicate signals for minor isomer, see Figure S38b, SI).

Interestingly, at temperatures below −70 °C the ^19^F NMR spectra for **5-Pf** showed the appearance of a second set of minor signals that is indicated with black arrows in Figure 3b, and a similar phenomenon is seen in the low temperature ^1^H NMR data of **5-Pf** (Figure S36; SI). The corresponding correlations in the ^19^F,^19^F-COSY NMR spectrum are illustrated in Figure S38b (SI) and strongly suggest that the total number of signals is the same as for the major component. Hence, these signals are tentatively assigned to a less favorable isomer in which both boron centers are positioned on the same side relative to the pyrene plane (approximate *C*_s_ rather than inversion symmetry). Integration of the ^19^F NMR signals reveals a ratio of 4:1 for the major versus the minor isomer of **5-Pf** (Figure S38b; SI). Similar results are obtained from analysis of the ^19^F NMR spectrum of **6-Pf** at −95 °C, but in this case the signals for the minor component for most part overlap with those of the major component (Figure S40b; SI). Computational studies on **5-Pf**′ indicate that the corresponding “*cis*”-isomer with both boron groups on the same side relative to the pyrene plane is indeed slightly higher in energy by ΔΔ*H* = 4.8 kJ mol^–1^, ΔΔ*S* = −2.4 J­(mol K)^−1^, and ΔΔ*G*_298_ = 5.5 kJ mol^–1^ (Tables S5–S6; SI). Based on these values, the computed ratio of *trans*- to *cis*-isomer is expected to be 96.5:3.5 at −110 °C, which is higher than the experimentally determined ratio of 80:20. At room temperature the computed ratio decreases to 90:10, but fast exchange prevents the observation of individual isomers expected to be present in solution.

### Electronic Structure Calculations

To assess the donor–acceptor character of the compounds, DFT calculations were performed at the B3LYP/6–31G­(d) level of theory. The geometric parameters for the optimized structures generally agree well with those derived from the X-ray structure analyses (Table 1). Notably, the computed B–N bond distances for **5-Pf′** and **6-Pf′** (1.646–1.651 Å) are relatively shorter than those for **5-Me′** and **6-Me′** (1.691–1.694 Å), which is expected because of the enhanced Lewis acid strength of the perfluoroaryl-substituted borane moieties. Consistent with the X-ray structures, relatively large C_R_–B–C_R_ angles of 115.5–117.6° are seen between the pendent R groups for all compounds. Most importantly, the distortions of the B–N heterocycles with significant displacements of the boron atoms from the pyrene plane (0.19–0.48 Å) and rotation of the pyridyl groups relatively to the pyrene plane (20.7–26.5°) are well reproduced.

The computed frontier molecular orbital distributions and relative energy levels for the boron complexes and the arylamine-substituted dipyridylpyrene precursors are illustrated in Figure 4. For all compounds, the HOMO orbital is delocalized over the pyrene core and into the arylamine pendent donor groups. However, some differences are seen in the relative contributions of the arylamine and pyrene units to the HOMO. Most notably, for **6-Pf′** but not **5-Pf′**, the contributions of the arylamine groups to the HOMO largely outweigh that of pyrene. The opposite is true for the HOMO–2 which together with the HOMO contributes to the lowest energy S_0_ →S_1_ transitions of **L2′**, **6-Me′**, and **6-Pf′** (see Figure S55 and Table S7). This is attributed to the separation of the electron-rich arylamines from the pyrene core by the phenylene linkers. Attachment of B­(C_6_F_5_)_2_ moieties makes the pyrene core in **6-Pf′** less electron-rich and disfavors orbital delocalization into the arylamine pendent groups. While the HOMO and HOMO–2 feature contributions from the pyrene and arylamine groups, the LUMOs extend from the pyrene to the pyridyl substituents with much increased contributions from the pyridyl groups after boron complexation. The differences in the HOMO and LUMO localization suggest that these compounds are best viewed as quadrupolar ‘cruciform’-type donor–acceptor compounds with the arylamine moieties acting as the donor and the boron-complexed pyridyl groups as the acceptor components.^17^

**Figure 4:**
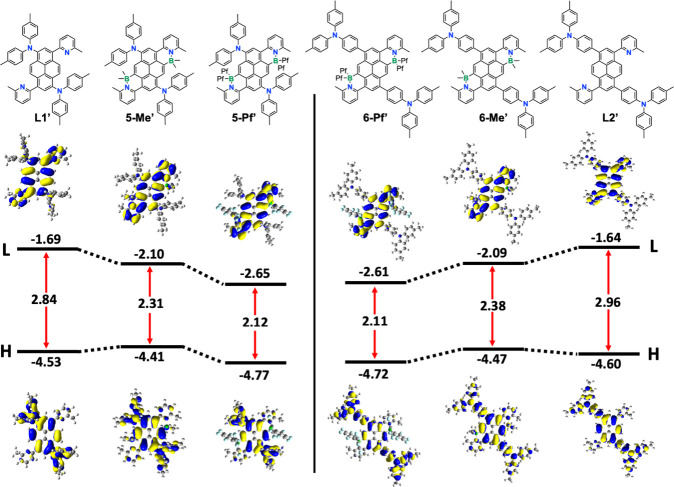
Calculated orbital energy levels and HOMO/LUMO (H/L) orbital plots of free ligands and B←N Lewis pair complexes (RB3LYP/6–31G­(d), energies given in eV; Me in place of longer alkyl and ^*t*^Bu groups, Pf = pentafluorophenyl).

Some interesting trends are also seen in the relative frontier orbital energy levels when complexing **L1′** and **L2′** with BMe_2_ and B­(C_6_F_5_)_2_ groups, respectively. The HOMO energy levels slightly increase when introducing the BMe_2_ groups, which can be rationalized by the inductive electron-donating effect of the tetracoordinate boron moieties on the pyrene core. This trend is reversed when strongly electron-withdrawing C_6_F_5_ groups are attached to boron, resulting in a decrease in the HOMO energies as previously observed for related B­(C_6_F_5_)_2_ complexes.^18^ The LUMO energy levels decrease upon B–N Lewis pair formation in all cases because of the electron-withdrawing effect on the pyridyl groups, but the effect is much stronger for the B­(C_6_F_5_)_2_ than the BMe_2_ groups. The decrease in the LUMO energy levels largely outweighs the changes in the HOMO energy levels, resulting in a large decrease in the HOMO–LUMO gaps after boron complexation. This effect is especially pronounced for the perfluoroaryl-substituted derivatives **5-Pf′** and **6-Pf′**.

### Electrochemical Studies

We subjected the B←N Lewis pair complexes and their respective ligand precursors to cyclic and square wave voltammetry measurements to experimentally verify the ambipolar character and the predicted trends in the orbital energy levels caused by substituent effects. To achieve good reversibility of the redox waves, reduction processes were studied in THF and oxidation processes in CH_2_Cl_2_ containing 0.1 M Bu_4_N­[PF_6_] (**5-Pf** and **6-Pf** showed well reversible oxidation and reduction events in CH_2_Cl_2_, see Figure S44, but the ethyl derivatives performed better when using different solvents for the oxidation and reduction processes). Cyclic voltammetry data are illustrated in Figure 5 and summarized in Table 2, and square wave voltammetry data are presented in Figure S43 and Tables S2–S3 in the SI. The reduction scans in THF reveal two well-separated reversible redox waves for both the ligands and the boron complexes. The potential for these processes increases gradually in the order **L1** < **5-Et** < **5-Pf** and **L2** < **6-Et** < **6-Pf**, in excellent agreement with the computational results. The nature of the amine substituents (NPh_2_ versus Ph-NPh_2_) has only a minor effect on the reduction processes. The oxidation scans also reveal two well-separated distinct redox waves (except for **6-Et**, discussed further below). For **L1**, **5-Et**, and **5-Pf**, these waves correspond to transfer of a single electron. As was predicted computationally, the oxidation for **5-Et** occurs at much lower potential that for **5-Pf** (and even **L1**); the inductive electron-donating effect is even more pronounced in the experimental data because of the use of ethyl rather than methyl groups on boron. Comparison of the relative peak intensities (Figure S44) shows that for **L2** and **6-Pf** the first oxidation wave corresponds to transfer of two electrons. The different behavior is attributed to the spatial separation of the arylamine moieties from the pyrene core, leading to essentially simultaneous oxidation of the two arylamine groups. At higher potential, a third electron is transferred, and this process is believed to be pyrene-centered. For **6-Et**, the electron-donating effect of the BEt_2_ group again shifts the pyrene-centered oxidation to lower potential, so much so that this process occurs even prior to oxidation of the arylamines. The corresponding redox wave is not fully reversible, indicating that the corresponding tricationic species suffers from poor stability. Overall, we conclude that compounds **5** and **6** show characteristics typical of ambipolar molecules; especially **5-Pf** and **6-Pf** combine attractive features that include reversible reductions at moderately negative potentials due to low-lying pyrene/pyridine-centered LUMO levels, reversible oxidations at moderate positive potentials due to pyrene/arylamine-centered HOMO levels, as well as superior chemical and electrochemical stability.

**Figure 5:**
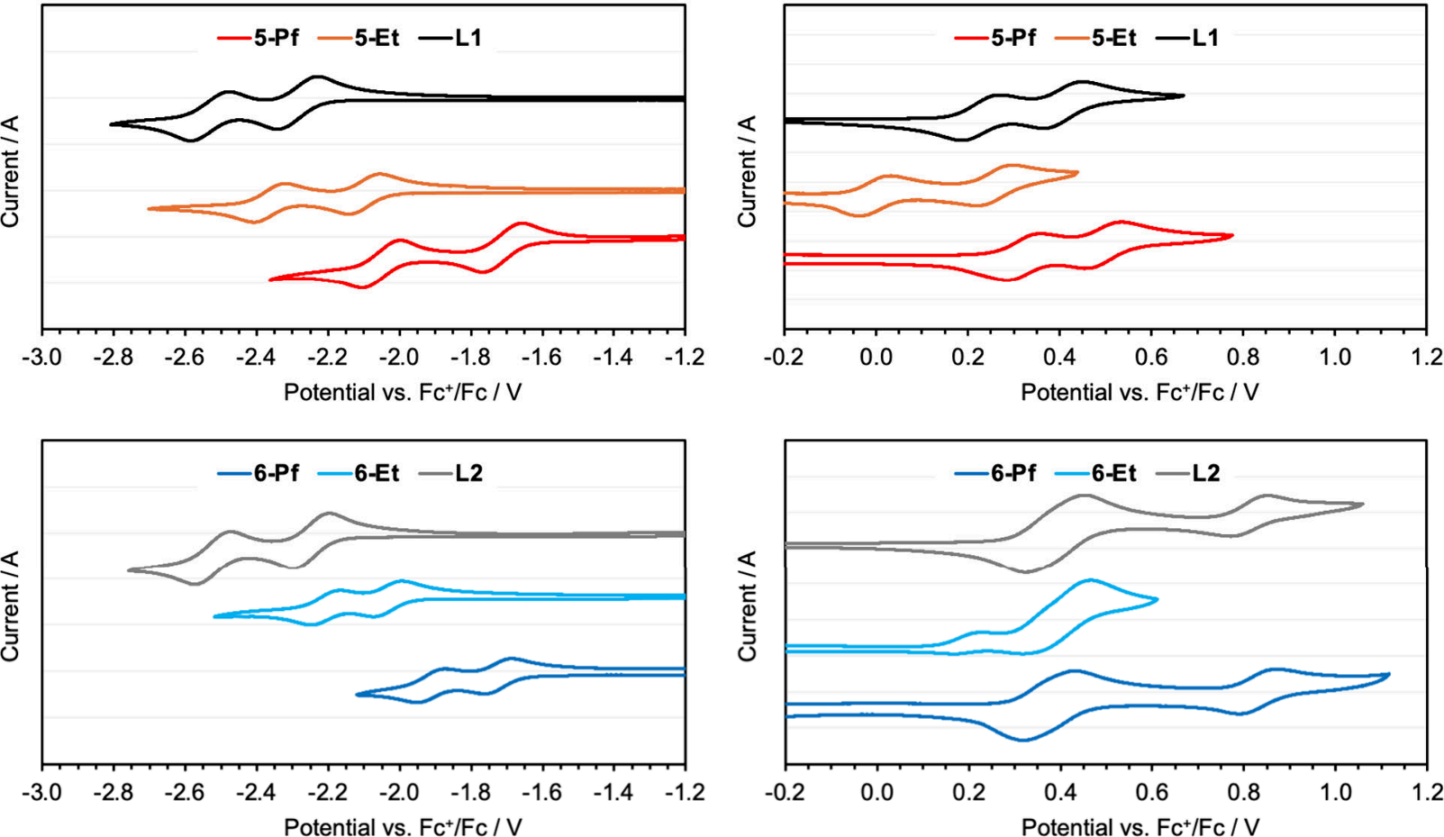
Cyclic voltammetry (CV) data for the reduction (left) and oxidation (right) of B–N Lewis pair functionalized donor–acceptor pyrenes and their precursors as ca. 1 mM solutions in THF (reduction) or DCM (oxidation) containing 0.1 M Bu_4_N­[PF_6_]. Data recorded at ν = 100 mV s^–1^ and reported vs Fc^+/0^; for oxidation scans decamethylferrocene (Fc*) was used as the internal reference and the data converted to *E*(Fc^0/+^) = 0 V using the equation *E*(Fc*^0/+^) = *E*(Fc^0/+^) −0.54 V.

**Table 2:** Summary of Electrochemical Data of B–N Lewis Pair Functionalized Donor–Acceptor Pyrenes and Their Precursors, and Comparison with Energy Gaps from UV-vis and DFT Studies

	*E*_ox 1,2_ ^a^/V (CV)	*E*_red 1,2_ ^a^/V (CV)	*E*_HOMO_ ^b^/eV (CV)	*E*_LUMO_ ^b^/eV (CV)	Δ*E*_gap_ ^b^/eV (CV)	*E*_HOMO_ ^c^/eV (DFT)	*E*_LUMO_ ^c^/eV (DFT)	Δ*E*_g_ ^c^/eV (DFT)	Δ*E*_g_ ^d^/eV (UV–vis)
**L1**	0.23, 0.41	–2.29, −2.53	–5.03	–2.51	2.52	–4.53	–1.69	2.84	2.53
**5-Et**	0.04, 0.26	–2.10, −2.37	–4.84	–2.70	2.14	–4.41	–2.10	2.31	2.06
**5-Pf**	0.32, 0.50	–1.71, −2.05	–5.12	–3.09	2.03	–4.77	–2.65	2.12	2.00
**L2**	0.39,^e^ 0.81	–2.25, −2.52	–5.19	–2.55	2.64	–4.60	–1.64	2.96	2.68
**6-Et**	0.20,^f^ 0.39	–2.03, −2.21	–5.00	–2.77	2.23	–4.47	–2.09	2.38	2.21
**6-Pf**	0.38,^e^ 0.83	–1.72, −1.91	–5.18	–3.08	2.10	–4.72	–2.61	2.11	2.12

a Derived from cyclic voltammetry data, *E*_red_ or *E*_ox_ = 0.5 (*E*_pc_ + *E*_pa_).

b *E*_LUMO_ = −(4.8+*E*_red_), *E*_HOMO_ = −(4.8+*E*_ox_).

c For compounds with Me in place of longer alkyl and ^*t*^Bu groups from DFT calculations (RB3LYP/6–31G­(d)).

d From absorption onset.

e First and second oxidation process overlap (2-electron process).

f First oxidation process is irreversible and the following oxidation processes overlap (2-electron process).

### Photophysical Studies

The absorption and emission spectral features further demonstrate the pronounced effect of B–N Lewis pair functionalization on the electronic structure and photophysical properties, as well as distinct differences depending on the mode of attachment of the arylamine moieties (Figure 6). While **L1** shows an absorption maximum at 408 nm in THF solution, those of **5-Et** (489 nm) and **5-Pf** (515 nm) are largely shifted to longer wavelengths. The heightened electron-withdrawing character of the B­(C_6_F_5_)_2_ Lewis acid groups results in a larger shift. A similar trend is seen for **L2** (383 nm), **6-Et** (470 nm), and **6-Pf** (480 nm), but the additional phenylene linker to the amino groups leads to absorptions that occur consistently at higher energy. Time-dependent density functional theory (TDDFT) calculations suggest that the lowest energy transitions are largely HOMO-to-LUMO in nature, involving significant charge transfer from arylamine to pyridyl-centered orbitals (Table S7).

**Figure 6:**
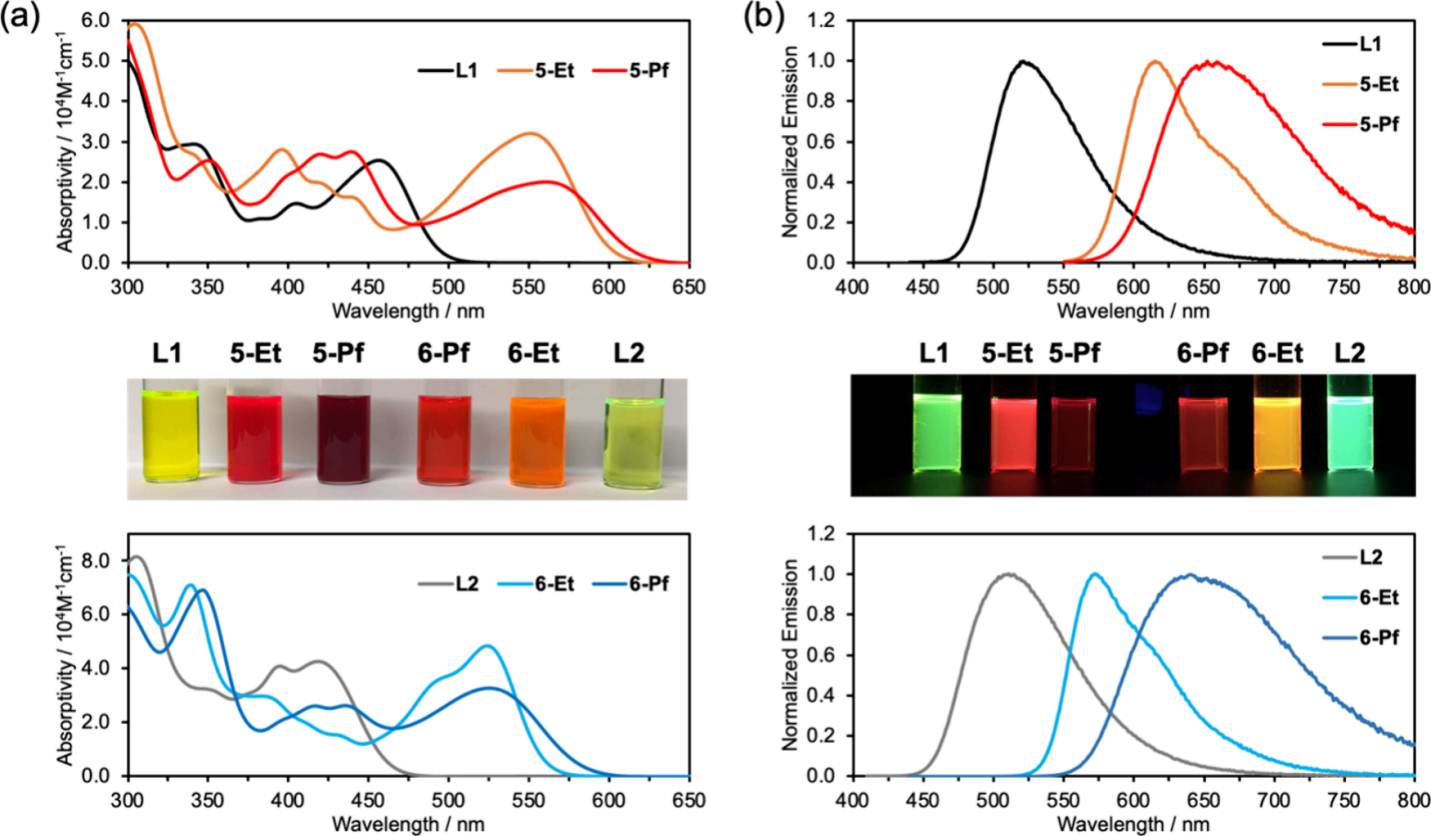
(a) UV–vis absorption and (b) fluorescence spectra of B–N Lewis pair functionalized donor–acceptor pyrenes and their precursors in THF excited at longest wavelength absorption maxima; photographs of THF solutions under natural light and under irradiation with a hand-held 365 nm UV lamp.

All compounds proved to be highly emissive with the emission colors ranging from green to deep red (Figure 6). Broad bands were seen in THF solution for the ligands and B­(C_6_F_5_)_2_ complexes and more structured bands for the BEt_2_ complexes. The wavelengths of maximum emission reflect well the trends seen in the absorption spectra. The quantum yields are very high for the green-emissive ligands (69%, 78%) and yellow-orange emissive complexes **5-Et** (615 nm, 77%) and **6-Et** (573 nm, 82%). Even the deep red emissive complexes **5-Pf** (652 nm, 23%) and **6-Pf** (640 nm, 44%) emit with appreciable quantum yields. The high quantum yields are reflective of the rigid structure and extended π-conjugated system of the chromophores, while the decrease in the emission quantum yield of **5-Pf** and **6-Pf** is due to enhanced nonradiative decay for these red emitters as expected based on the band gap law (Table 3). The fluorescence lifetimes fall into the low nanosecond range (3.2–6.8 ns), typical for this type of fluorophore.

**Table 3:** Comparison of Photophysical Properties of B–N Lewis Pair Functionalized Donor–Acceptor Pyrenes and Their Precursors in THF Solution and the Solid State

	λ_abs,THF_ (ε)/nm (10^4^ M^–1^ cm^–1^)	λ_abs,TD‑DFT_/nm	λ_Fl,THF_/nm^a^	Stokes/cm^–1^	Φ_Fl,THF_/%^b^	τ_Fl,THF_/ns^c^	*k*_r_ ^d^/10^7^ s^–1^	*k*_*nr*_ ^d^/10^7^ s^–1^	λ_Fl,solid_/nm^e^	Φ_Fl,solid_/%^d^	τ_Fl,solid_/ns^f^
**L1**	456 (2.54)	408	523	2809	69	6.8 (χ^2^ = 1.25)	10	4.6	527	9.1	τ_1_ = 1.2, 87%
	405 (1.48)										τ_2_ = 4.4, 13%
	340 (2.95)										(χ^2^ = 1.01)
**5-Et**	550 (3.20)	485	615	1922	77	6.6 (χ^2^ = 1.18)	12	3.6	643	7.1	τ_1_ = 1.4,57%
	440 (1.63)										τ_2_ = 3.1,4.1%
	420 (1.98)										τ_3_ = 9.3, 2%
	396 (2.80)										(χ^2^ = 1.08)
**5-Pf**	560 (2.01)	512	652	2520	23	τ_1_ = 4.8, 81%	4.2	14	661	15	τ_1_ = 4.7, 29%
	439 (2.76)					τ_2_ = 8.5, 19%					τ_2_ = 8.3, 71%
	420 (2.70)					(χ^2^ = 1.28)					(χ^2^ = 1.41)^c^
	349 (3.00)										
**L2**	418 (4.26)	383	511	4354	78	3.2 (χ^2^ = 1.00)	24	6.9	512	25	τ_1_ = 1.6, 61%
	395 (4.09)										τ_2_ = 4.7, 31%
	348 (3.30)										τ_3_ = 29.3, 8%
											(χ^2^ = 1.38)
**6-Et**	524 (4.85)	470	573	1632	82	4.8 (χ^2^ = 1.09)	17	3.8	616	9.6	τ_1_ = 1.3, 76%
	494 (3.62)										τ_2_ = 3.4, 24%
	431 (1.54)										(χ^2^ = 1.08)
	409 (2.04)										
	385 (2.98)										
**6-Pf**	525 (3.27)	480	640	3423	44	5.6 (χ^2^ = 1.13)	7.9	10	646	14	τ_1_ = 1.0, 15%
	435 (2.62)										τ_2_ = 4.9, 4%
	417 (2.61)										τ_3_ = 0.4, 81%
	347 (6.91)										(χ^2^ = 1.57)^c^

a Excited at longest wavelength absorption maximum.

b Absolute quantum yield determined using an integrating sphere.

c Excited with a nanoLED at 450 nm.

d Radiative (*k*_r_) and nonradiative (*k*_nr_) decay rate constants in solution estimated using the equations *k*_r_ = Φ/τ, *k*_nr_ = (1 – Φ)/τ).

e Excited at 390 nm (**L1**, **5-Et**, **L2**, **6-Et**), 500 nm (**5-Pf**), or 526 nm (**6-Pf**).

f Excited with a nanoLED at 390 nm unless noted otherwise.

Large Stokes shifts are seen for the free ligands and the perfluoroaryl-substituted complexes (2520–4354 cm^–1^), whereas those for the ethyl-substituted complexes (1922, 1632 cm^–1^) are somewhat less prominent, suggesting subtle differences in the charge transfer character of the excited states depending on the substitution pattern on boron. A pronounced solvatochromic effect is observed in the emission spectra, where especially the free ligands and the perfluoroaryl-substituted complexes **5-Pf** and **6-Pf** display a strong redshift with increasing solvent polarity, consistent with an increased polarization and charge transfer character in the excited state (Table S4, Figure S45–S46). In the solid state, the emission bands of compounds **5-Et** and **6-Et** are further red-shifted (Figure S47), and the quantum yields are largely reduced (7.1%, 9.6%). In contrast, for **5-Pf** and **6-Pf**, the bathochromic shifts are small, and the quantum yields remain relatively high (15%, 14%). We attribute the stronger solid-state emission of **5-Pf** and **6-Pf** to the steric effect of the pentafluorophenyl groups that disfavors bimolecular quenching mechanisms.

### Aggregation-Induced Emission Characteristics

Pyrenes are well-known to form aggregates, and depending on the nature of the substituents, these aggregates can exhibit either aggregation-caused quenching (ACQ) or aggregation-induced emission enhancement (AIEE).^19^ Prompted by the relatively high solid-state quantum yields of **5-Pf** and **6-Pf**, we examined their aggregation-dependent fluorescence behavior by gradually adding water as a poor solvent to solutions in THF as a good solvent (Figure 7). For both compounds, increasing the water fraction to 40% led to a significant decrease in emission intensity to approximately 20% of the value observed in pure THF, likely because of the increased polarity of the solvent medium. However, upon further increasing the water content from 50% to 90%, the fluorescence intensity greatly increased. For **5-Pf**, the emission intensity recovered to approximately 60%, and for **6-Pf**, it even reached 130%, exceeding that in pure THF. For both compounds, the normalized fluorescence spectra recorded in a 10:90 THF/water mixture showed narrower emission profiles compared to those in pure THF. The emission maximum of **5-Pf** remained essentially unchanged, whereas that of **6-Pf** exhibited a blue shift from 640 to 629 nm. The more pronounced aggregation effect observed for **6-Pf** can be attributed to the reduced steric shielding of the pyrene core and the greater rotational freedom of the phenyl rings in the pendent NPh_2_ moieties. The results contrast the slight redshift observed in the solid-state spectra, along with a lower fluorescence quantum yield relative to that in pure THF. This suggests that the molecular packing and intermolecular interactions in the aggregated state differ from those in the solid state.

**Figure 7:**
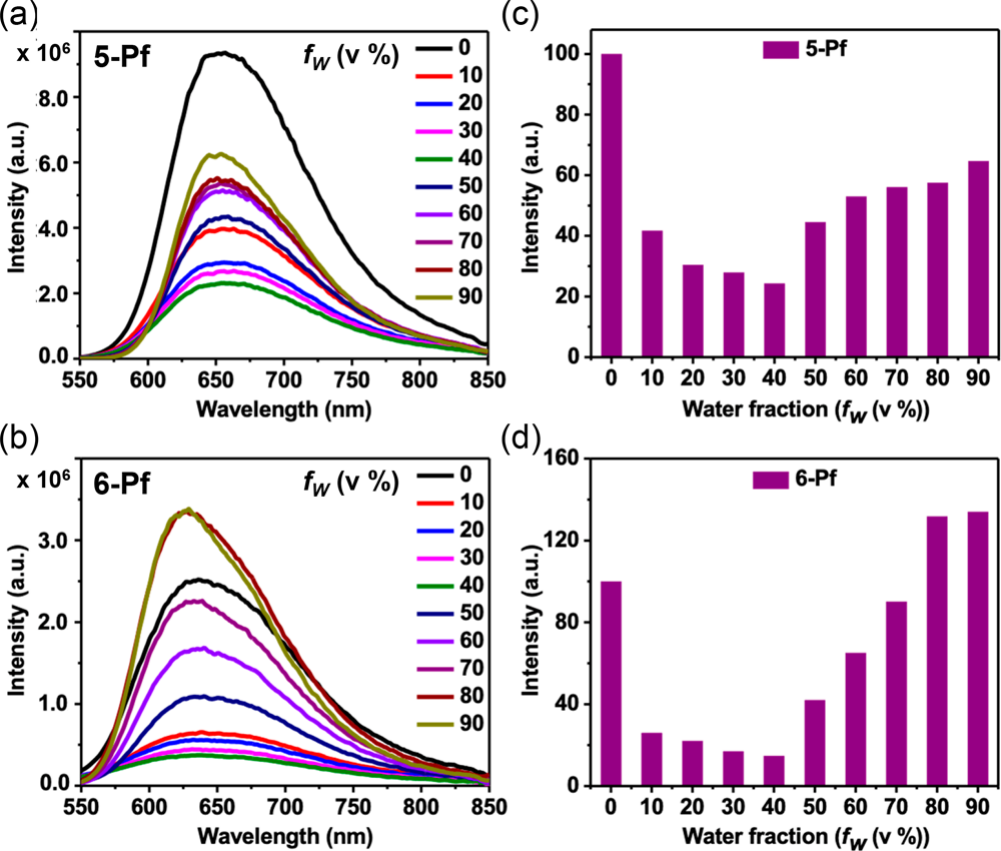
Emission spectra of (a) **5-Pf** and (b) **6-Pf** in THF containing different water fractions *f*_*w*_ (v%) at 10 μM concentration (λ_ex_ = 540 nm for **5-Pf** and 500 nm for **6-Pf**). Fluorescence intensity versus solvent composition of the THF–water mixtures for (c) **5-Pf** and (d) **6-Pf**.

Dynamic light scattering (DLS) measurements confirmed aggregate formation in aqueous-rich mixtures (Figures S57–S58). In 10:90 THF/water, the Z-average hydrodynamic diameter of the particles for **5-Pf** and **6-Pf** was measured to 264 and 286 nm, respectively. In a 50:50 THF/water mixture, larger aggregate sizes were observed, suggesting the formation of more loosely packed assemblies.

### Exploration of **5-Pf** in Live Cell Imaging

While BODIPY and triarylborane-based dyes have been widely used for live cell imaging applications,^10,20^ the development of new effective fluorogenic probes is of continued interest. For imaging of the cytoskeleton, probes that are highly fluorogenic and nontoxic, exhibit absorptions and emissions in the far-red, and show high specificity in living cells are highly advantageous.^21^ Considering the intense low energy emission of **5-Pf** and **6-Pf** in solution and the solid state, as well as their enhanced stability due to the presence of electron-deficient pentafluorophenyl groups on boron,^22^ those compounds were considered for cell imaging applications. Despite the slightly lower solution quantum yield, bioimaging studies were performed using **5-Pf** because of the relatively longer wavelength of the emission and higher solubility in DMF. A solution of **5-Pf** in DMF was diluted into aqueous Dulbecco’s Modified Eagle Medium (DMEM) for staining, and DLS analysis confirmed the expected formation of nanoaggregates of the dye (Figure S59). Photostability studies suggested that the dye could be utilized for both short- and long-term live cell analysis (Figure S60).

Comparison of differential interference contrast brightfield and epifluorescence images of **5-Pf** dye-stained live mouse embryonic fibroblasts (MEF) cells identified that **5-Pf** was localizing to discrete, motile vesicular structures. To investigate further, we utilized Madin Darby Canine Kidney (MDCK) epithelial cells, which have been widely employed to study vesicular motility and intracellular trafficking.^23^ Analysis by confocal microscopy of live MDCK cell cultures costained for both **5-Pf** and commercially available LysoTracker dyes identified the presence of extensive colocalization of **5-Pf** and LysoTracker to numerous intracellular puncta (Figure 8a, blue arrows).^24^ There was no observable generalized staining or staining of other vesicular compartments. Previously, it had been reported that uptake and retention of some lipid soluble dyes may be sensitive to the action of calcium efflux transporters which can be inhibited by treatment with verapamil.^21,25^ Treatment of MDCK cells with 10 μM of verapamil significantly increased the fluorescence signal in LysoTracker positive vesicles further, without detectable change in the localization pattern or overall cell morphology (Figure 8b,c). The high degree of colocalization of **5-Pf** with LysoTracker indicated that **5-Pf** dye was preferentially localizing to acidic compartments, including lysosomes, within the cell. To identify whether the staining pattern was pH sensitive, we treated cell cultures with a weak penetrating base, NH_4_Cl, which has been previously used to increase the pH of acidic compartments.^26^ Treatment of MDCK cells with 20 mM of NH_4_Cl resulted in effectively complete loss of vesicular staining by **5-Pf** dye (Figure 8d). Under the same conditions, LysoTracker dye continued to retain some vesicular staining, possibly indicating that **5-Pf** is more sensitive to changes in pH than LysoTracker.

**Figure 8:**
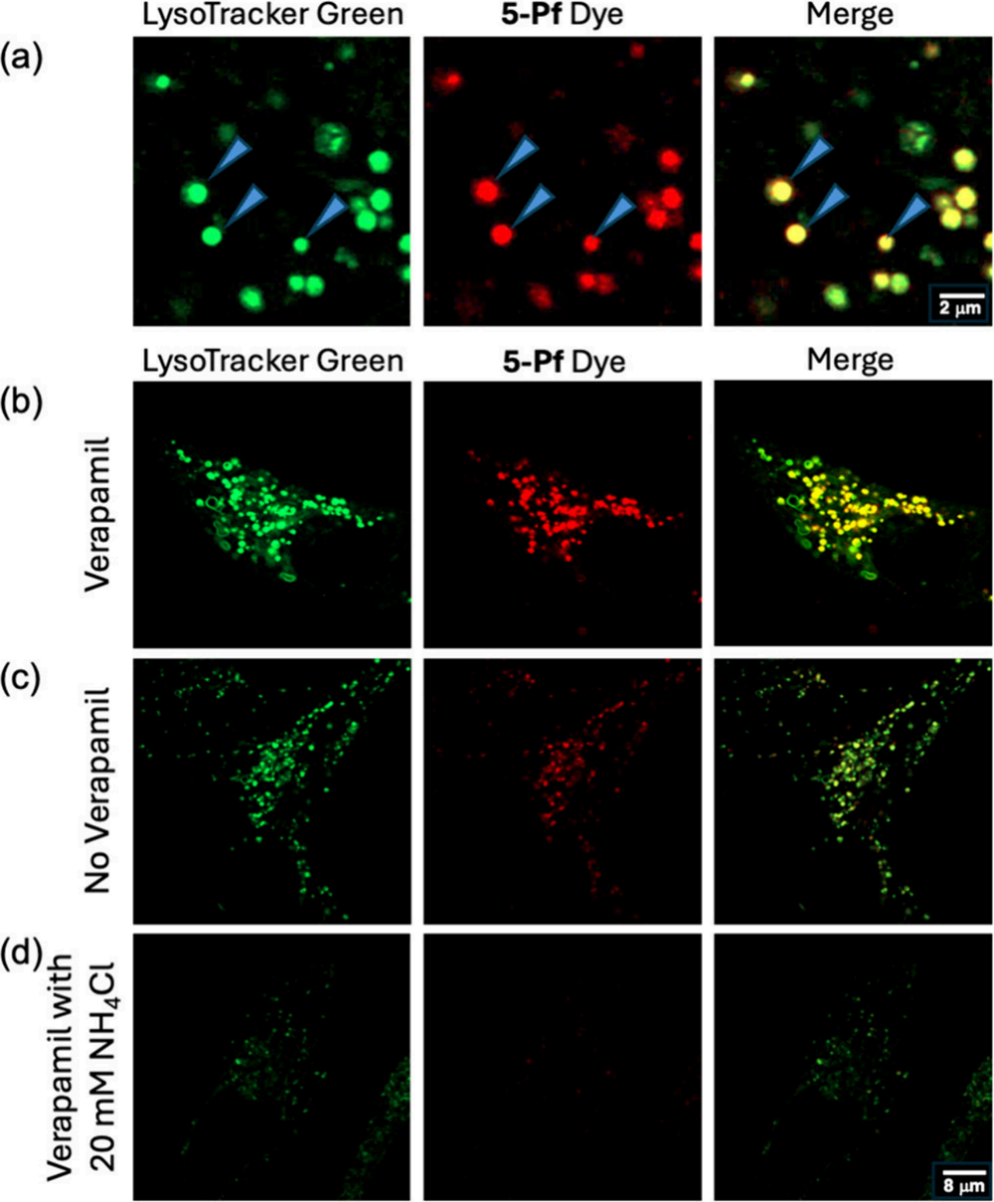
MDCK epithelial cells stained by LysoTracker Green and **5-Pf** (each at 100 nM concentrations in DMEM, which also contained 0.02% DMF). All images were collected using a Zeiss LSM980 confocal microscope with Airyscan 2 detection at 561 nm (2% laser strength) and 488 nm (0.10% laser strength). (a) High magnification field of individual vesicles costained with **5-Pf** and LysoTracker Green; note that not every vesicle is costained with the dyes, indicating differential staining specificity. (b–d) Lower magnification fields of individual cells costained with **5-Pf** and LysoTracker in the (b) presence of 10 μM of verapamil, (c) absence of verapamil, and (d) presence of 10 μM of verapamil and 20 mM of NH_4_Cl. Each row is an image of the same cell at the same magnification.

### Electrochemiluminescence (ECL) of **5-Pf**

Boron chelates are also of significant interest as luminophores that exhibit electrochemiluminescence properties. While much of the earlier studies had focused on BODIPY derivatives,^27^ new boron chromophores are starting to emerge as promising candidates.^15^ Electrochemiluminescence or electrogenerated chemiluminescence (ECL),^14^ is a light emission phenomenon where radicals generated through electrochemical reactions engage in electron-transfer reactions. This results in the creation of excited species, which emit light when they return to their ground state. Remarkably, this phenomenon occurs without the requirement of an external light source,^14c^ via annihilation and coreactant mechanisms as two different ECL pathways.^28^ The annihilation ECL pathway involves the direct recombination of electrogenerated radical cations and anions produced at an electrode by alternate sweeping of potential, generating an excited state species which can eventually generate light.^28,29^ Conversely, the coreactant ECL pathway utilizes additional chemical species (coreactants) to generate an active radical that reacts with a luminophore radical, producing an excited state more efficiently and thus enhancing ECL emission.^14a,14d,29,30^ ECL plays a vital role in advancing the development of new materials,^31^ including those that emit light for optoelectronics and sensors.^14d,31,32^ It also provides outstanding selectivity through the utilization of specific chemiluminescent reactions, antibodies, or molecular recognition elements, particularly in applications like immunoassays and DNA analysis.^14a,33^

We carried out an in-depth study involving photoelectrochemical and spectroelectrochemical measurements during ECL processes in dichloromethane as the solvent. Figure 9 illustrates the DPVs of compound **5-Pf**, with an inset of the CV and ECL-voltage curves. When sweeping in the positive potential direction from 0 V to +1.00 V, two anodic peaks of the two consecutive oxidations were discernible at +0.48 V and +0.62 V vs SCE. The DPVs remove the background currents and enhance sensitivity, resulting in well aligned peak heights and peak potentials and indicating good reversibility. Scanning from 0 V to −2.20 V revealed two cathodic peaks at −1.68 V and −1.92 V for the consecutive reductions which were followed by two anodic peaks for the corresponding reductions in the reverse scan from −2.20 to 0 V. Analysis of the peak potentials and heights for the set of observed redox processes demonstrated again a reversible system. The inset of Figure 9 displays the CV curve in red and the corresponding ECL-voltage curve in blue for **5-Pf** in the annihilation pathway. The oxidation set of peaks in the CV scan showed slightly greater reversibility than the reduction peaks as their peak heights and peak potentials are more identical, indicating stable behavior of the electrogenerated radical cation and dication species. This was not the case for the reduction set of peaks, possibly due to solvent effects. DCM solvent and **5-Pf** had similar reduction potentials, therefore the coupled reduction reactions with the solvent presented less reversible peaks in this reduction region. The ECL intensifies to 5.2 nA when scanning toward a negative potential of −1.7 V (Figure 9, inset). This indicates that the previously generated radical cation is more stable, allowing it to persist in solution until the radical anion is produced. Subsequently, these entities can diffuse together, leading to the generation of ECL.

**Figure 9:**
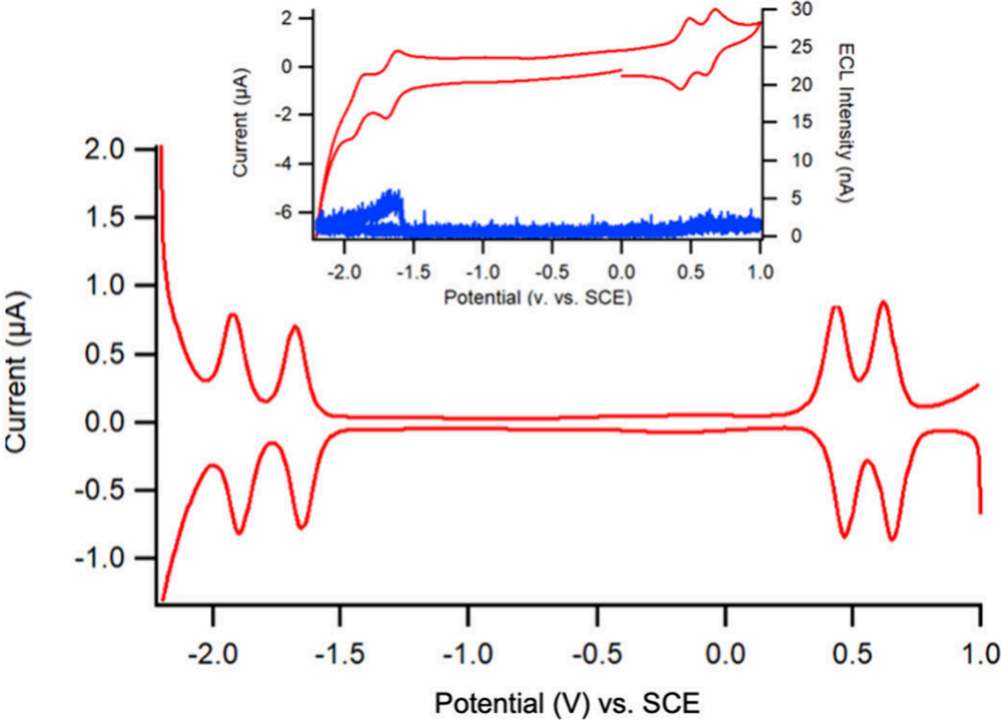
Differential pulse voltammograms (DPVs) along with an inset for the CV (red) and corresponding ECL-voltage (blue) curves of a 0.2 mM solution of **5-Pf** at a 2 mm platinum electrode submerged in a 3 mL electrolyte solution of dichloromethane (DCM) containing 0.1 M of TBAPF_6_ (potential reported vs SCE).

Next, potential pulsing ECL experiments were conducted to see whether the intensity of ECL could be enhanced by rapid stepping between the oxidation and reduction potentials. Figures 10a-c illustrate the pulsing ECL graphs of compound **5-Pf** in the annihilation mechanism. Employing a potential profile that rapidly steps between the first oxidation (+0.55 V) and first reduction (−1.80 V) within a brief 100 ms time frame ensures an ECL enhancement because this quick potential stepping process effectively preserves the radical species over their lifetime in the vicinity of the electrode before they decay (Figure 10a and 11a).^34^ It is then plausible that an electron transfer takes place from the radical anion’s HOMO to the radical cation’s semioccupied molecular orbital (SOMO), leading to the creation of an excited state that subsequently relaxes back to the ground state, with ECL released. Notably, the ECL intensity increased considerably, reaching around 9.0 μA. This very substantial enhancement is attributed to the likely promotion of the annihilation reaction before the radical cation decays by rapid potential stepping, which is essential for producing the excited state for ECL generation.

**Figure 10:**
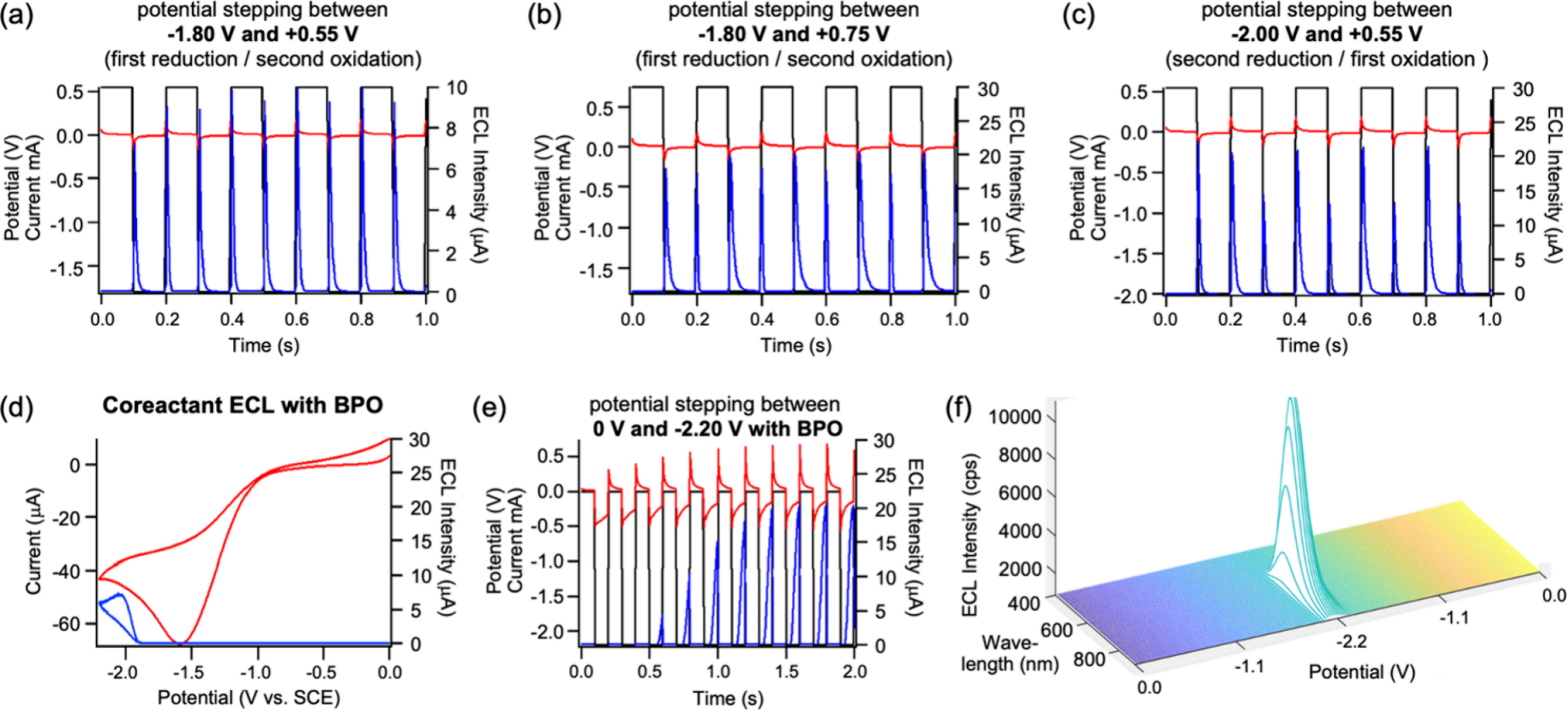
ECL annihilation of a 0.2 mM solution of **5-Pf** at a 2 mm platinum electrode submerged in a 3 mL electrolyte solution of DCM containing 0.1 M of TBAPF_6_ where current–time (red), ECL-time (blue), and potential-time (black) profiles are illustrated in potential stepping experiments between (a) the first reduction at −1.80 V and first oxidation at +0.55 V; (b) the first reduction at −1.80 V and second oxidation at +0.75 V; (c) the second reduction at −2.0 V and first oxidation at +0.55 V, all at a pulsing frequency of 10 Hz; (d) CV (red) and corresponding ECL-voltage curve (blue) of 0.2 mM **5-Pf** and 5 mM BPO in a 3 mL electrolyte solution of DCM containing 0.1 M of TBAPF_6_; (e) current–time (red), ECL-time (blue), and potential-time (black) profiles of the electrolyte solution in (d), obtained in potential stepping experiments between 0 V and −2.20 V, at a pulsing frequency of 10 Hz; (f) spooling ECL spectra of a 0.1 mM solution of **5-Pf** at a 2 mm platinum electrode submerged in a 3 mL electrolyte solution of DCM containing 5 mM BPO, and 0.1 M of Bu_4_NPF_6_ with a scan rate of 0.025 V/s and exposure time for each spectrum of 1s.

**Figure 11:**
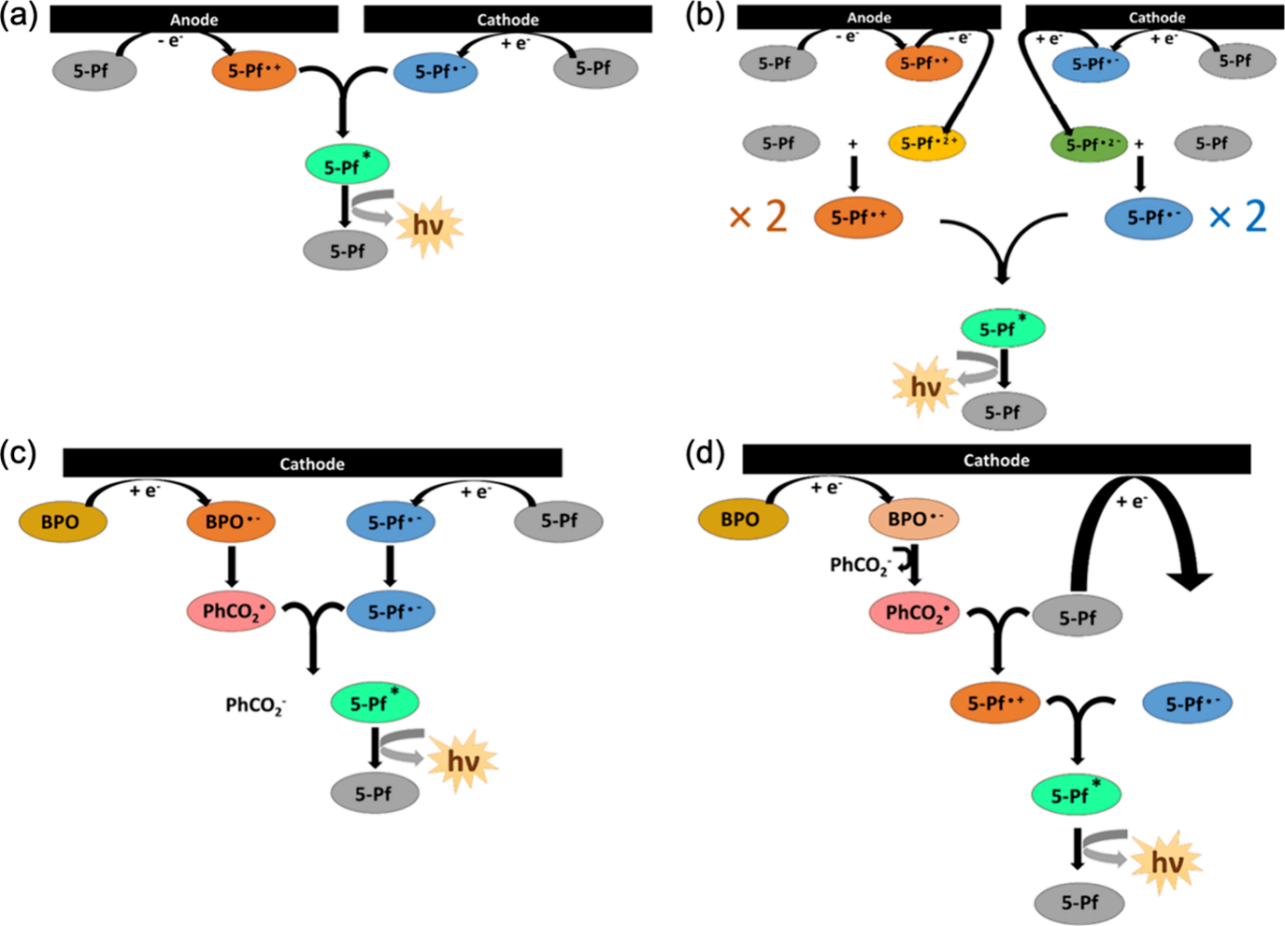
(a) and (c) Annihilation ECL reaction mechanisms of **5-Pf**; (b) and (d) coreactant ECL reaction mechanisms for the **5-Pf**/BPO system.

Pulsing experiments with potential switches between the first reduction (−1.80 V) and the second oxidation (+0.75 V) were also conducted to explore the possibility of further ECL enhancement (see Figure 10b and 11b).^34^ Figure 10b clearly shows an increase in the ECL signal to 20 μA. This is likely due to the formation of dications, which can then react with neutral **5-Pf** to produce twice the radical cation concentration, thereby enhancing the ECL signal intensity. In general, radical cations tend to exhibit higher stability than radical anions, prompting us to also explore the effect of implementing two reductions. The pulsing experiments yielded similar results when switching between potentials for formation of the dianion (−2.00 V) and radical cation (+0.55 V), leading to an even further improved ECL signal of about 22 μA (Figure 10c). Again, this is attributed to the generation of dianions which can react with the neutral species to generate double the concentration of the radical anion near the surface of the electrode. This approach yielded the highest ECL intensity observed in annihilation pulsing experiments.

Experiments were also performed exploring the second pathway of ECL that involves the coreactant route, where 5 mM of benzoyl peroxide (BPO) was introduced into the previously described ECL solution. Figure 10d shows the CV (red) and corresponding ECL-voltage curve (blue) of **5-Pf** in the coreactant system. We observed that when BPO is introduced in the solution, the reduction reactions for **5-Pf** are no longer visible as the BPO concentration is much higher than that of **5-Pf**. The onset potential for ECL is approximately −1.90 V, as indicated in Figure 10d, after the second reduction occurs (see Figure 9). This signifies the initiation of dianion generation, where the dianion subsequently reacts with neutral species to yield twice the radical anion. Subsequently, this radical anion can react with the benzoate radical. The benzoate radical in turn can extract an electron from the HOMO of the radical anion, resulting in the production of the excited state species of **5-Pf**, leading to the generation of ECL (Figure 11c). The reduction potential of BPO occurs at approximately −1.52 V, indicating that the reduction of **5-Pf** occurs concurrently, at a similar potential value. However, this contrasts with the ECL scenario. Since the negative ECL onset potential is −1.90 V (Figure 10d), it is imperative to first generate the dianion before ECL can be obtained. We presume that BPO has the capability to generate a benzoate radical which can quickly accept an electron from **5-Pf** and is a very strong oxidizing agent (E°_benzoate_ = 1.5 V vs SCE).^35^ The benzoate radical exhibits the capability to partake in redox reactions as an oxidative coreactant, in conjunction with **5-Pf**, facilitating energy transfer and resulting in augmented luminescence. In this context, the coreactant participates in an electron transfer reaction, resulting in the formation of a BPO radical anion (BPO^•–^) through the acceptance of an electron. Subsequently, this radical anion can decompose, releasing benzoate (PhCO_2_^–^) and forming a benzoate radical (PhCO_2_^•^). This benzoate radical can then interact with the radical anion of the luminophore (**5-Pf** ^•–^) ultimately leading to the generation of ECL (Figure 11c).

Applying potential pulsing techniques in the presence of BPO can further amplify the ECL signal, as demonstrated in Figure 10e, where an ECL peak intensity of 20 μA is observed. Pulsing experiments were conducted by potential stepping from 0 V to −2.20 V with a pulsing frequency of 10 Hz, revealing a delay in ECL rising reactivity. When the potential is switched to −2.20 V, ECL is generated, but the intensity rises only slowly. At −2.20 V, both the benzoate radical and the **5-Pf** radical anion must be generated and may react with each other. A possible explanation for the delayed rise in ECL is then that the rate of the reaction between radical anion **5-Pf** ^•‑^ and the smaller benzoate radical is slow because the larger size of the **5-Pf** luminophore results in slower diffusion. The effect is noticeable as the pulse gradually rises after a 90 ms delay before the components react, meet, and eventually the peak ECL emission is reached (Figure 10d).

To conclude, contrary to expectations, the formation of PhCO_2_^•^ appears to play a crucial role in the coreactant mechanism. Once PhCO_2_^•^ is established, it has the capacity to oxidize neutral **5-Pf**, transforming it into a radical cation **5-Pf** ^•+^ at the electrode. This mechanism enables the radical cation **5-Pf** ^•+^ to interact with the radical anion **5-Pf** ^•–^ already present near the electrode surface (Figure 11d). The two radicals can then engage in a reaction, giving immediate rise to the generation of ECL. However, it is crucial to note that the rise in ECL intensity was delayed, suggesting that the presence of bulky substituents complicates the process. Consequently, the ECL onset potential does not align well with the expected mechanism. Instead, it leads us to conclude that the coreactant ECL experiments are more likely to follow the mechanism described in Figure 11c, which involves oxidation of the larger radical anion **5-Pf** ^•–^ by the smaller PhCO_2_^•^ to generate ECL.

#### Spectroscopy Experiments and Absolute ECL Quantum Efficiencies of **5-Pf**

We performed spooling ECL spectroscopy^35,36^ experiments for 0.1 mM **5-Pf** in a 3 mL DCM solution containing 5 mM BPO and 0.1 M of TBAPF_6_ to measure ECL spectra at specific time intervals while systematically scanning the applied potential.^37^ The 3D images in Figure 10f were obtained sequentially through a CCD attached to a spectrograph during voltammetric scanning from 0 V to −2.20 V, and then back to 0 V. Throughout the scanning process at a rate of 0.025 V/s, the ECL peak wavelength remained constant, while the peak intensities varied as the scan moved to the cathodic limit and back. The resulting spectra reveal an ECL onset potential of −1.90 V and a potential of −2.20 V at which maximum emission is reached at a peak wavelength of 646 nm. The data match reasonably well the ECL profile in the CV experiment presented in Figure 9. The spooling ECL spectra are compared with the PL spectra in Figure S62. The PL and ECL emission wavelengths for **5-Pf** were similar, with the PL spectra in the same electrolyte solution showing maximum emission at around 660 nm and the ECL spectra at around 646 nm, suggesting the presence of a single excited state species. We note that in the case of ECL, the multistep electrochemical reaction takes time to generate excited states, allowing for subsequent relaxation, while PL involves a much faster excitation process.

Absolute ECL quantum efficiency (Φ_ECL_) is a very important measure in ECL reaction systems, which looks at the efficiency of an electrochemical reaction in producing light and is quantified as the ratio between the emitted photons to the number of electrons involved. A new technique for precisely measuring the absolute quantum efficiency in ECL processes was applied as described in the SI. The absolute quantum efficiencies (Φ_ECL_) determined for **5-Pf** under different experimental conditions are summarized in Table S9. In the ECL annihilation route, Φ_ECL_ was modest at 0.00016 ± 0.00002%. Interestingly, the Φ_ECL_ for the ECL annihilation route increased significantly in pulsing experiments, as also seen in the clear rise in ECL intensity from 5.2 nA to 9.0 μA for **5-Pf**. Notably, when the BPO coreactant was employed, a substantial further increase in annihilation ECL intensity was seen, boosting the efficiency to Φ_ECL_ = 0.9 ± 0.1%, a value that far surpasses that of a previously reported B–N Lewis pair functionalized corannulene luminophore^15b,15d,38^ and is also above that determined for the ECL gold standard [Ru­(bpy)_3_]^2+^ of Φ_ECL‑CV_ = 0.53 ± 0.07% with BPO as the coreactant. BPO effectively aided in enhancing radical formation and electron transfer to/from the luminophore, leading to a higher likelihood of excited state formation and increased ECL quantum efficiency. When employing BPO in potential pulsing experiments, **5-Pf** exhibited a relatively lower Φ_ECL_ of 0.40 ± 0.02%. Thus, it is evident that **5-Pf** in the presence of BPO favors a CV scan due to its slower pace, allowing ample time for the radical anion of the luminophore and the benzoate radical to diffuse and react together. The BPO experiments not only demonstrated its reliability as a coreactant, substantially enhancing ECL intensity, but revealed that **5-Pf** performs better in CV scans than high-speed potential pulsing experiments when the coreactant is present.

## Conclusions

Most pyrene derivatives emit in the 400–500 nm region, limiting their application potential, especially in the biomedical field, and making the development of efficient methods to further optimize the electronic structures and luminescence characteristics critically important. An effective approach to modulate the emission of pyrene by introducing arylamine donors and B–N Lewis-pair acceptors in orthogonal positions is presented. X-ray crystal structure analyses of the newly synthesized ambipolar pyrene derivatives show the selective formation of distorted six-membered B–N heterocycles, with the boron atoms attached to the 4,9-positions of the K region and positioned slightly above and below the pyrene plane. VT ^1^H and ^19^F NMR revealed dynamic processes in solution and the presence of two different conformers at low temperature, which, based on computational studies, are attributed to different conformers in which the boron atoms are located either on opposite or the same side of the pyrene plane.

The quadrupolar structure promotes intramolecular charge transfer (ICT) as the HOMO is delocalized over the pyrene core and arylamine pendent donor groups, and the LUMO extends from the pyrene core to the pyridyl substituents, with greatly increased contributions from the pyridyl groups after boron complexation. The lowest HOMO and LUMO levels and the smallest HOMO–LUMO gaps of 2.11 eV (**6-Pf**) and 2.12 eV (**5-Pf**) are achieved when C_6_F_5_ groups are attached to boron. The computed trends are well reproduced by electrochemical measurements, which reveal two consecutive one-electron-reductions and two reversible one-electron-oxidations for the compounds with the arylamine moieties directly attached to pyrene (**5**), indicative of strong electronic communication. In contrast, for compounds with the arylamine moieties separated from the pyrene core (**6**), a two-electron wave for oxidation of the arylamine groups is followed by an additional pyrene-centered one-electron oxidation.

The effect of B–N Lewis pair formation is also reflected in large redshifts in the absorption and emission compared to the nonborylated precursors. The borylated complexes are strongly emissive with quantum yields reaching to 82% for the yellow-orange emitting BEt_2_ complexes and to 44% for the deep red to near-IR emitting B­(C_6_F_5_)_2_ derivatives, the latter showing particularly large Stokes shifts of up to 4354 cm^–1^. Importantly, for the B­(C_6_F_5_)_2_ derivatives, the quantum yields remain relatively high (up to 15%) even in the solid state, because of AIEE effects. The photophysical attributes are ideal for potential applications as lipid-soluble dyes in biomedical imaging. As **5-Pf** stands out for its strong long wavelength emission and relatively higher solubility in polar media, we further explored this dye in the staining of MDCK epithelial cells. Extensive colocalization of **5-Pf** and LysoTracker suggests preferential accumulation in acidic cellular compartments, including lysosomes. The fluorescence signal intensity could be effectively increased by staining with verapamil as inhibitor of calcium efflux transporters.

The formation of strongly emissive excited states, combined with the reversible formation of multiply charged cations and anions, proved ideal for achieving electrochemiluminescence in the red-to-near-IR spectral region. Studies on the ECL properties of **5-Pf** show that rapid potential pulsing between the second reduction potential and the first or second oxidation produces a large ECL signal of up to 22 μA, indicating that the dianion of **5-Pf** reacts with neutral **5-Pf**, effectively doubling the radical anion concentration. The addition of BPO as a coreactant during CV scanning also greatly enhances the ECL intensity of **5-Pf** in annihilation experiments. Spooling ECL experiments show clear evolution and devolution of ECL, yielding a single ECL peak at 646 nm. The latter is in good agreement with the photoluminescence data for **5-Pf**, thus confirming that ECL and PL emission arise from the same excited state. An absolute ECL efficiency (Φ_ECL_) of 0.269 ± 0.002% for **5-Pf** is achieved in annihilation pulsing mechanisms, with BPO enhancing the efficiency further to an impressive 0.9 ± 0.1% during CV scanning. Overall, this investigation uncovered critical insights into the electrochemical and ECL properties of pyrene donor–acceptor systems, further highlighting their promising potential in optoelectronic devices and novel light-responsive materials.

## Supplementary Material



## Notes

Experimental details and characterization data (PDF)
