# Supplementary material for: Regioselective Synthesis of Ambipolar B–N Lewis Pair Functionalized Pyrenes: Structural Dynamics, Emission Tuning, and Applications in Live Cell Imaging and as Electrochemiluminescent Materials
Source: J Am Chem Soc. 2026 Mar 9;148(11):12044–60. doi: 10.1021/jacs.5c22679 (PMC13022892; doi:10.1021/jacs.5c22679)
Supplement: Supplementary file 1 [file ja5c22679_si_001.pdf]

SUPPORTING INFORMATION

FOR

Regioselective Synthesis of Ambipolar B−N Lewis Pair Functionalized Pyrenes: Structural Dynamics, Emission Tuning, and Applications in Live Cell Imaging and as Electrochemiluminescent Materials

Mukundam Vanga,[a] Sara Jahanghiri,[b] Justin Davis,[c] Rajendra Prasad Nandi,[a] Ashutosh Sahoo,[a] Pavel Kucheryavy,[a] Roger A. Lalancette,[a] Edward M. Bonder,[c,]* Zhifeng Ding,[b,]* Frieder Jäkle[a,]*

[a] Department of Chemistry, Rutgers University-Newark, 73 Warren Street, Newark, NJ 07102, USA

[b] Department of Chemistry, University of Western Ontario, London ON N6A 5B7, Canada

[c] Department of Biological Sciences, Rutgers University-Newark, 195 University Avenue, Newark, NJ 07102, USA

* Email: ebonder@newark.rutgers.edu; zfding@uwo.ca; fjaekle@newark.rutgers.edu

1. Materials and General Synthesis and Characterization Methods

Synthetic Methods

All reactions were carried out under an atmosphere of pre-purified nitrogen using either Schlenk techniques or an inert-atmosphere glovebox, whereas the products are air stable and were isolated under ambient conditions. THF was distilled from Na/benzophenone prior to use. Hydrocarbon and chlorinated solvents were purified using a solvent purification system (alumina/copper columns for hydrocarbon solvents, alumina columns for chlorinated solvents), and the chlorinated solvents were subsequently distilled from CaH_2 and degassed via several freeze-pump-thaw cycles. All chemicals were purchased from commercial sources and directly used without further purification unless noted otherwise. Compounds **2** and **4** were synthesized by adopting the reported procedures.[S1, S2]

Characterization

NMR data were acquired at ambient temperature. 160 MHz $^{11}B\{^1H\}$ and 470 MHz ^{19}F NMR data were recorded on a 500 MHz Bruker AVANCE III HD spectrometer, 500 MHz ^1H and 126 MHz $^{13}C\{^1H\}$ NMR data on a 500 MHz Bruker Auto AVANCE III HD spectrometer, and 600 MHz ^1H and 151 MHz ^{13}C NMR data on a Varian INOVA 600 spectrometer. $^{11}B\{^1H\}$ NMR spectra were acquired with boron-free quartz NMR tubes on the 500 MHz Bruker AVANCE with a boron-selective 5 mm PH SEX 500S1 ^{11}B-H/F-D probe. ^1H and $^{13}C\{^1H\}$ NMR spectra were referenced internally to solvent signals ($CDCl_3$: 7.26 ppm for ^1H NMR, 77.16 ppm for ^{13}C NMR, and all other NMR spectra externally (trifluorotoluene $\delta(^{19}F)$ = -63.72 ppm, $BF_3{\bullet}Et_2O$ $\delta(^{11}B)$ = 0 ppm). Abbreviations used for signal assignments: Pyr = pyrenyl, Ph = phenyl, Py = pyridyl, Et = ethyl, s = singlet, d = doublet, t = triplet, q = quartet, p = pentet, dd = doublet of doublets, m = multiplet, br = broad.

High-resolution atmospheric pressure chemical ionization (APCI) and electrospray ionization mass spectrometry (ESI-MS) data were obtained on an Apex Ultra 7.0 Hybrid FTMS or an Orbitrap Exploris 240 instrument.

Photophysical and Electrochemical Studies

UV-visible absorption data were acquired on a Varian Cary 5000 UV-Vis/NIR spectrophotometer. The fluorescence data and lifetimes were measured using a Horiba Fluorolog-3 spectrofluorometer equipped with a 388 or 450 nm nanoLED and a FluoroHub R-928 detector. Absolute quantum yields (Φ_F) were measured on the HORIBA Fluorolog-3 using a pre-calibrated Quanta-φ integrating sphere. Light from the sample compartment is directed into the sphere via a fiber-optic cable and an F-3000 Fiber-Optic Adapter, and then returned to the sample compartment (and to the emission monochromator) via a second fiber-optic cable and an F-3000 Fiber-Optic Adapter.

Cyclic voltammetry (CV) and square wave voltammetry (SWV) experiments were carried out on a BASI CV-50 W analyzer. The three-electrode system consisted of a gold disk as working

electrode, a Pt wire as secondary electrode, and an Ag wire as the pseudo-reference electrode. The voltammograms were recorded with ca. 1×10^{-3} M solutions in THF (reduction scans) or DCM (oxidation scans) containing $Bu_4N[PF_6]$ (0.1 M) as the supporting electrolyte. The scans were referenced after the addition of a small amount of ferrocene (reduction scans) or decamethylferrocene (Fc*) (oxidation scans) as internal standard. The potentials are reported relative to the ferrocene/ferrocenium couple. When Fc* was used as the internal reference the data was converted to $E(Fc^{0/+}) = 0$ V using the equation $E(Fc^{*0/+}) = E(Fc^{0/+}) - 0.54$ V.

X-ray crystallography

Single crystals suitable for X-ray analysis of **5-Et** were grown by slow evaporation of a solution in $CHCl_3$ and those of **5-Pf** by slow evaporation of a solution in a mixture of THF and acetonitrile. X-ray diffraction intensities were recorded at 100 K on a Rigaku XtaLAB Synergy-S Dual Source diffractometer equipped with a PhotonJet Cu-microfocus source (λ = 1.54178 Å) and a HyPix-6000HE detector. Data reduction and processing was performed with CrysAlisPro.[S3] Empirical and numerical (Gaussian) absorption corrections, determined by face indexing and integration were applied to the data. The structure was solved by the intrinsic phasing method in SHELXT and refined by full-matrix least-squares techniques against F^2 (SHELXL) in the Olex2 graphical user interface.[S4] Anisotropic thermal factors were applied for all atoms except for the hydrogen atoms. H atoms were placed in idealized positions and refined using a riding model. The disorder of one of the tridecyl chains in **5-Et** was modelled with major/minor contributions of 80:20 for C43-C47 and 60:40 for C48-C50; additional disorder in the *t*-butyl groups could not be modelled satisfactorily, primarily due to the presence of a disordered tridecyl chain in proximity. For the twinned structure of **5-Pf**, most of the disorder was found in the *t*-butyl groups. One of the carbon atoms, C44, had very large anisotropic displacements; we used the ISOR command to lessen these effects. Crystallographic data for the structures of **5-Et** and **5-Pf** have been deposited with the Cambridge Crystallographic Data Center as supplementary publication CCDC 2512851 and 2516943 respectively. Copies of the data can be obtained free of charge on application to CCDC, 12 Union Road, Cambridge CB2 1EZ, UK (fax: (+44) 1223-336-033; email: deposit@ccdc.cam.ac.uk).

2. Synthesis of BN LP-functionalized Donor-Acceptor Pyrenes

Scheme S1.

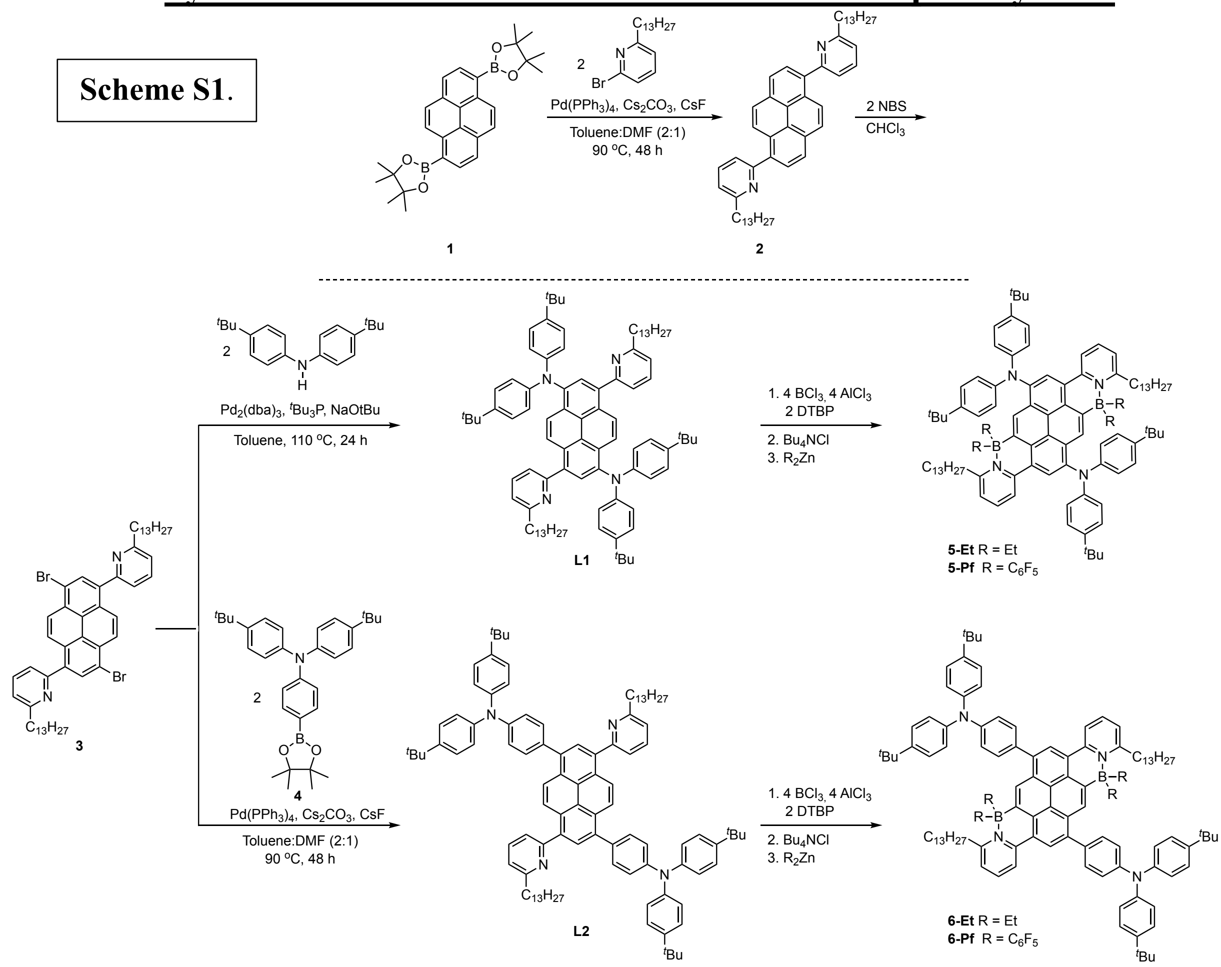

Synthesis of 6,6'-(3,8-dibromopyrene-1,6-diyl)bis(2-tridecylpyridine) (3)

A solution of 1,6-bis(6-tridecylpyridin-2-yl)pyrene (2.00 g, 2.77 mmol) in $CHCl_3$ (300 mL) was cooled to 0 ºC in an ice bath and then N-bromosuccinimide (1.09 g, 6.12 mmol, 2.2 equiv) was added. The resulting mixture was allowed to stir at RT overnight. Deionized H_2O (150 mL) was added, and the mixture was extracted with CH_2Cl_2 (3 x 50 mL). The combined organic layers were dried over Na_2SO_4, filtered and evaporated under reduced pressure. The resultant residue was recrystallized from dichloromethane and methanol to obtain the desired product as a pale-yellow solid. Yield: 2.10 (86 %).

^{1}H NMR (500 MHz, $CDCl_3$): δ = 8.50 – 8.43 (m, 6H, Pyr), 7.81 (t, J = 7.7 Hz, 2H, Py), 7.51 (d, J = 7.5 Hz, 2H, Py), 7.28 (d, J = 7.7 Hz, 2H, Py), 2.96 (t, J = 7.7 Hz, 4H), 1.89 – 1.85 (m, 4H), 1.48 – 1.44 (m, 4H), 1.42 – 1.37 (m, 4H), 1.35 – 1.26 (m, 32H), 0.88 (t, J = 6.9 Hz, 3H). ^{13}C{^{1}H} NMR (126 MHz, $CDCl_3$): δ = 163.15, 157.33, 137.66, 136.86, 132.45, 129.75, 128.57, 127.04, 126.70, 126.19, 123.08, 121.58, 120.87, 38.83, 32.07, 30.28, 29.86, 29.82, 29.73, 29.68, 29.51, 22.84, 14.26. HRMS (APCI in THF, pos. mode) m/z = 877.3673 ($[M+H]^+$, calcd for $^{12}C_{52}{}^{1}H_{67}{}^{79}Br_2{}^{14}N_2$ 877.3666).

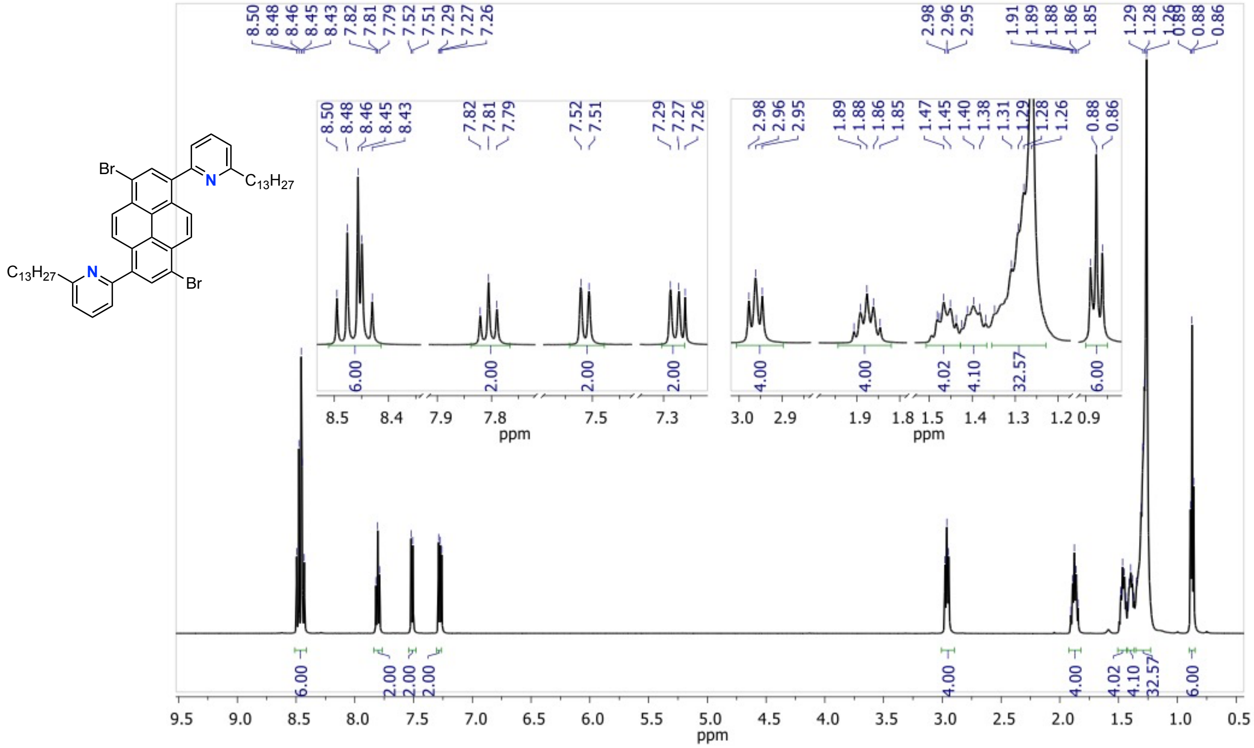

Figure S1. ^{1}H NMR spectrum and expansions of **3** in $CDCl_3$

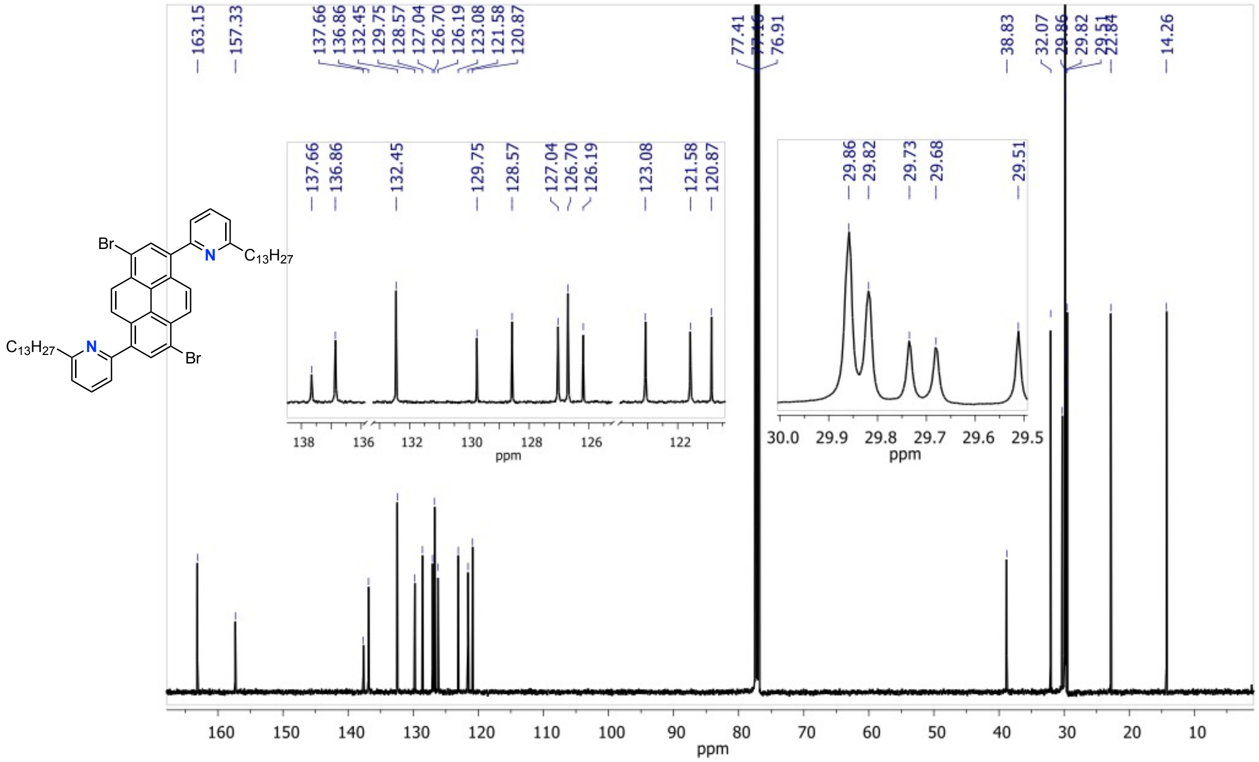

Figure S2. $^{13}C\{^{1}H\}$ NMR spectrum and expansions of **3** in $CDCl_3$

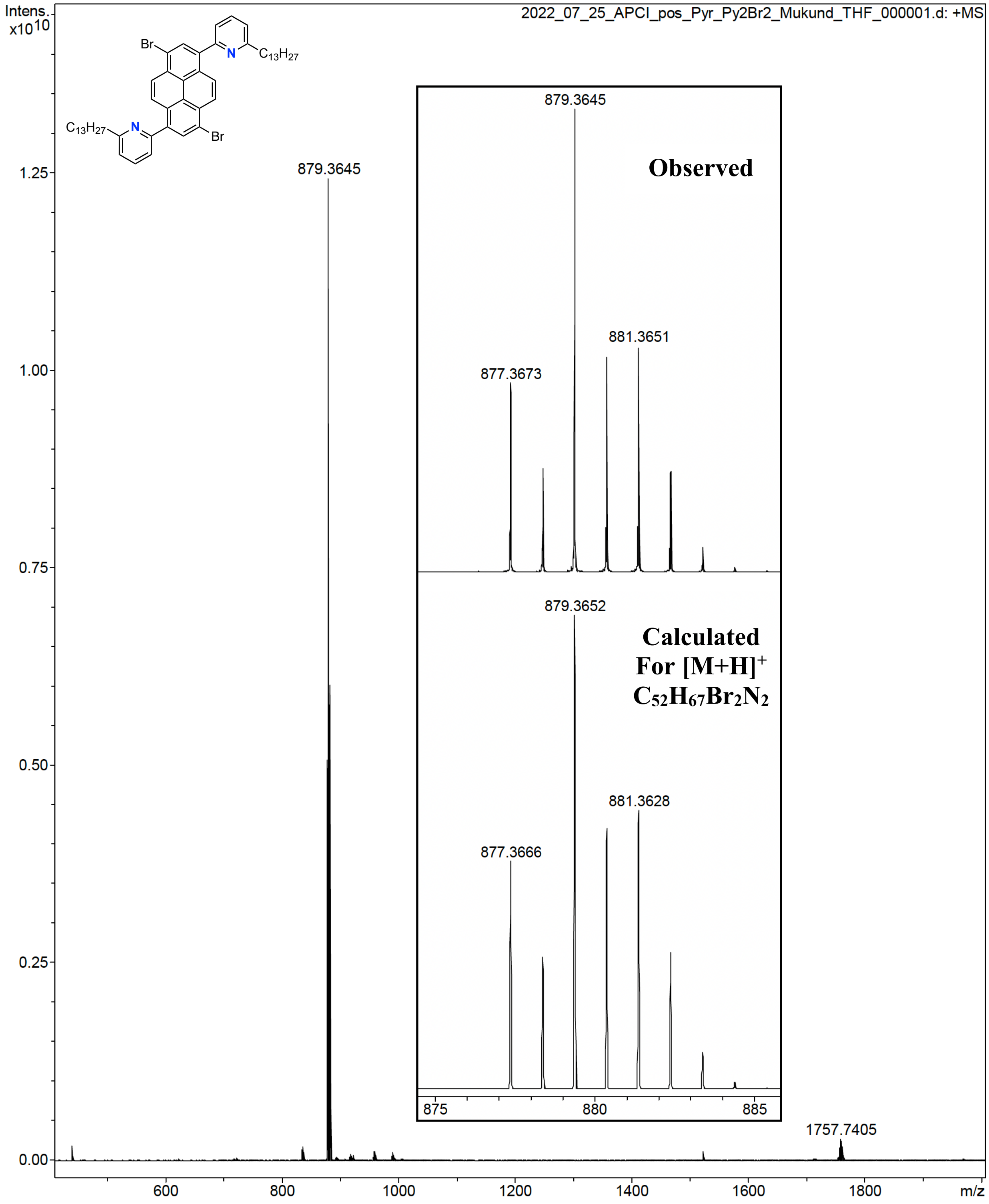

Figure S3. APCI-MS (pos. mode) of **3** in THF

Synthesis of *N*[1],*N*[1],*N*[6],*N*[6]-tetrakis(4-(*tert*-butyl)phenyl)-3,8-bis(6-tridecylpyridin-2-yl)pyrene-1,6-diamine (L1)

In a glove box, **3** (1.40 g, 1.59 mmol), bis(4-(*tert*-butyl)phenyl)amine (1.17 g, 4.14 mmol, 2.6 quiv), $Pd_2(dba)_3$ (117 mg, 127 µmol), tri-*tert*-butylphosphane (155 mg, 765 µmol), sodium *t*-butoxide (1.53 g, 15.9 mmol) were taken in 200 mL screw capped Schlenk tube and then 100 mL of anhydrous toluene was added. After removing the Schlenk tube from the glovebox, the mixture was stirred at 110 °C for 24 h, cooled to RT, and the solvent was then removed by rotary evaporation. Dichloromethane (200 mL) and water (100 mL) were added, the organic phase was collected, and the aqueous phase was extracted with dichloromethane. The organic extracts were combined, washed with brine, dried over Na_2SO_4 and evaporated to dryness. The residue was dissolved in $CHCl_3$, passed through a small plug of silica gel and celite, and the plug of silica gel and celite rinsed with chloroform (3 x 15 mL). The solvent was then removed under reduced pressure and the resultant residue was recrystallized from chloroform and methanol solution at -20 °C to give the desired product as a yellow solid. Yield: 1.61 g (79%).
^{1}H NMR (500 MHz, $CDCl_3$): δ = 8.26 (d, J = 9.5 Hz, 2H, Pyr), 8.17 (d, J = 9.5 Hz, 1H, Pyr), 7.96 (s, 2H, Pyr), 7.70 (t, J = 7.7 Hz, 2H, Py), 7.38 (d, J = 7.6 Hz, 2H, Py), 7.19 – 7.16 (m, 10H, ^{t}BuPh, Py), 7.02 (d, J = 8.7 Hz, 8H, ^{t}BuPh), 2.88 (t, J = 7.7 Hz, 4H), 1.79 (p, J = 7.5 Hz, 4H), 1.43 –1.38 (m, 4H), 1.35-1.32 (m, 4H), 1.28 (s, 36H, ^{t}Bu), 1.260 (s, 32H), 0.89 (t, J = 6.9 Hz, 3H). ^{13}C{^{1}H} NMR (126 MHz, $CDCl_3$): δ = 162.41, 158.80, 146.10, 144.14, 140.73, 137.49, 136.82, 129.74, 128.96, 127.67, 125.96, 125.81, 123.69, 123.04, 121.40, 120.97, 38.81, 34.27, 32.07, 31.57, 30.26, 29.86, 29.82, 29.82, 29.80, 29.71, 29.71, 29.51, 22.84, 14.28 ppm. HRMS (ESI in THF, pos. mode) m/z = 1278.9312 ([M]$^{+}$, calcd for $^{12}C_{92}{}^{1}H_{118}{}^{14}N_4$ 1279.9351).

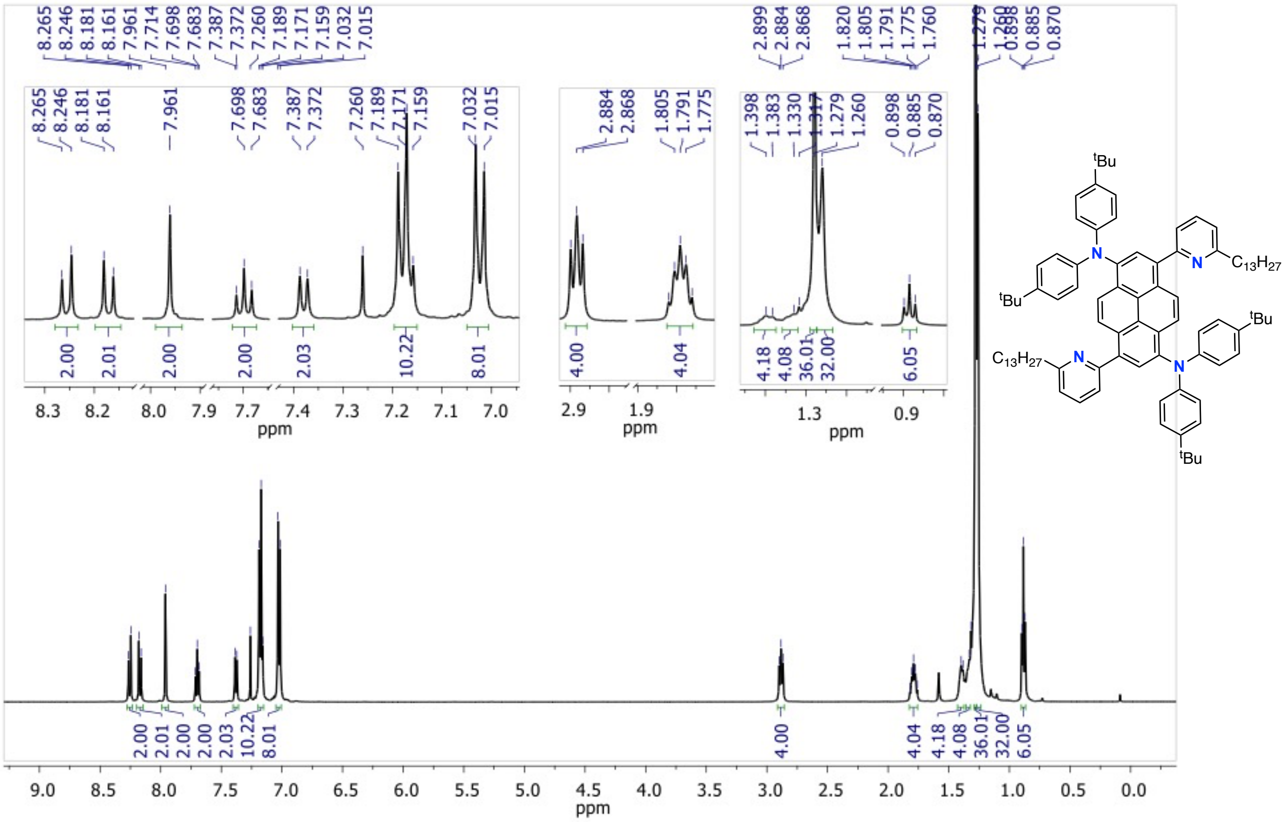

Figure S4. ^{1}H NMR spectrum and expansions of **L1** in $CDCl_3$

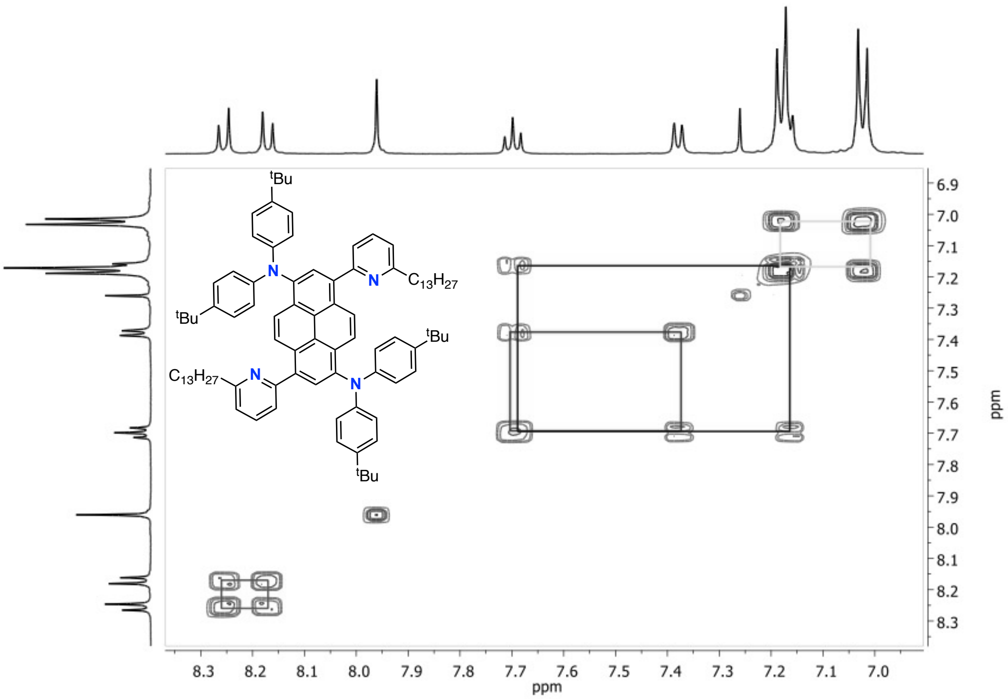

Figure S5. Aromatic region of the H,H-COSY NMR spectrum of **L1** in $CDCl_3$

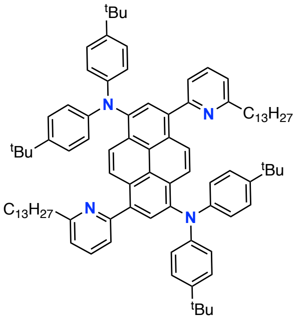

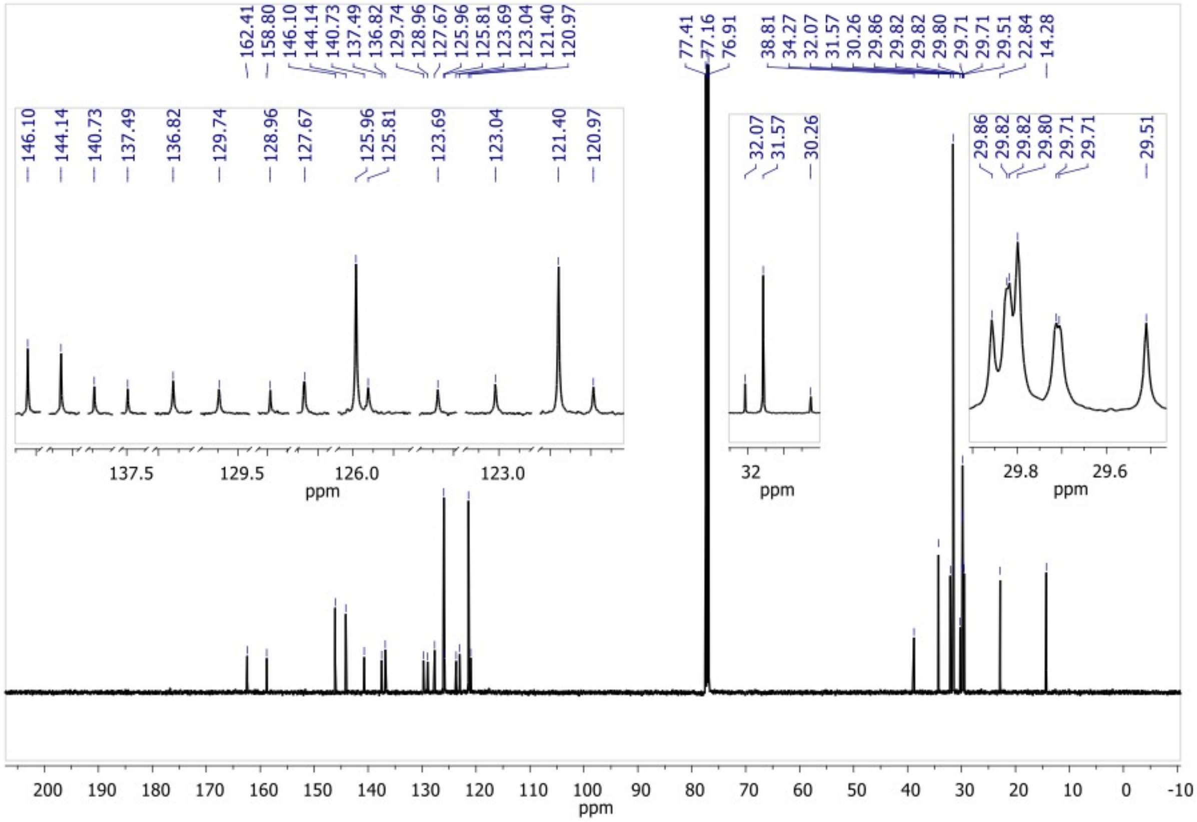

Figure S6. $^{13}C\{^1H\}$ NMR spectrum and expansions of **L1** in $CDCl_3$

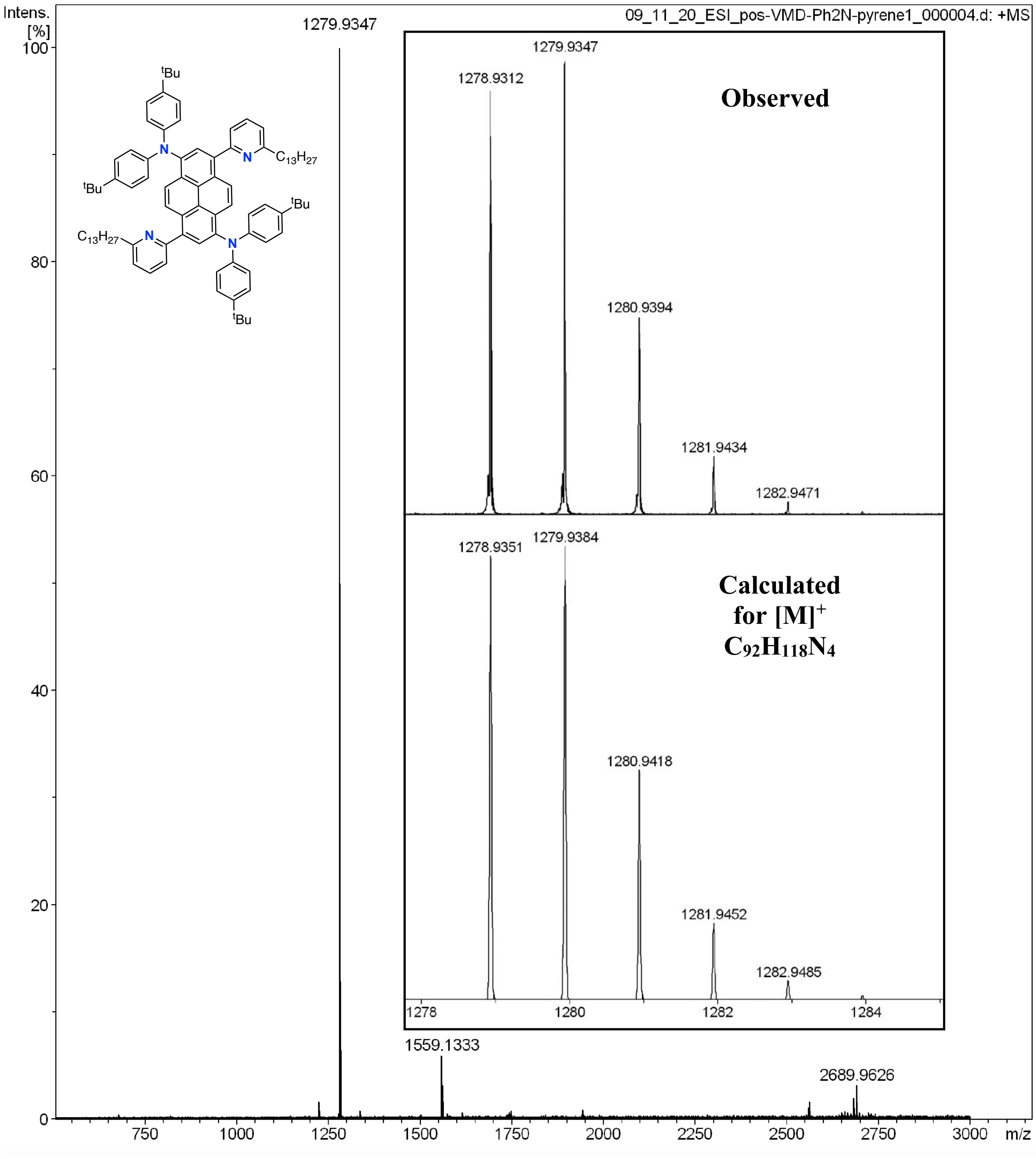

Figure S7. ESI-MS (pos. mode) of **L1** in THF

Synthesis of 5-Et

In a glove box, to a solution of **L1** (0.500 g, 0.391 mmol) in anhydrous CH_2Cl_2 (80 mL) were added BCl_3 (1.6 mL, 1.6 mmol, 1M in dichloromethane, 4 equiv), 2,6-di-*tert*-butylpyridine (175 μL, 0.78 mmol, 2 equiv) and $AlCl_3$ (208 mg, 1.56 mmol, 4 equiv) sequentially. After stirring at room temperature for 16 h, Bu_4NCl (228 mg, 0.78 mmol, 2 equiv) was added and the mixture allowed to stir for 15 min. Then Et_2Zn (160 μL, 1.56 mmol, 4 equiv) was added neat and the mixture was stirred for a further 16 h at room temperature. The reaction flask was removed from the glove box and the mixture was filtered through a small plug of silica gel. The solvent was removed under reduced pressure and the resultant residue was rinsed first with methanol and then cold pentane solution to give the desired product as a red solid. Yield: 0.46 g (83%).

^{1}H NMR (500 MHz, $CDCl_3$): δ = 8.27 (s, 2H, Pyr), 8.21 (s, 2H, Pyr), 7.87 (d, *J* = 8.0 Hz, 2H, Py), 7.74 (t, *J* = 7.9 Hz, 2H, Py), 7.24 (overlapped, 2H, Py), 7.23 (d, *J* = 8.6 Hz, 8H, ^{t}BuPh), 7.11 (d, *J* = 8.6 Hz, 8H, ^{t}BuPh), 3.43 (t, *J* = 8.0 Hz, 4H), 1.72 – 1.66 (m, 4H), 1.52 – 1.46 (m, 5H), 1.38 – 1.35 (d, *J* = 6.6 Hz, 4H), 1.29 (s, 36H, ^{t}Bu), 1.27 (s, 32H), 0.92 – 0.86 (m, 10H, $B\underline{CH_2}CH_3$ & tridecyl-CH_3), 0.55 – 0.47 (m, 4H, $B\underline{CH_2}CH_3$), 0.17 (t, *J* = 7.5 Hz, 12H, $BCH_2\underline{CH_3}$) ppm. $^{13}C\{^1H\}$ NMR (126 MHz, $CDCl_3$): δ = 163.97 (Py), 156.65 (br, B-C_{Pyr}), 156.44 (Py), 146.59 (NPh ipso), 143.94 (^{t}BuPh ipso), 140.45 (Pyr), 138.36 (Py-H), 131.50 (Pyr), 130.38 (Pyr), 126.57 (Pyr-H), 125.97 (^{t}BuPh), 125.65 (Pyr), 124.33 (Pyr-H), 124.21 (Py-H), 122.95 (Pyr), 122.19 (Py-H), 121.65 (^{t}BuPh), 35.65, 34.29 (^{t}Bu), 32.08, 31.84, 31.60 (^{t}Bu-H), 30.06, 29.84, 29.81, 29.78, 29.69, 29.59, 29.51, 22.85, 22.07 (br, $B\underline{C}H_2CH_3$), 14.27 (tridecyl-$\underline{C}H_3$), 10.53 (br, $BCH_2\underline{C}H_3$) ppm. ^{11}B NMR (160 MHz, $CDCl_3$): δ = 3.3 ppm. HRMS (ESI in THF, pos. mode) *m*/*z* = 1415.0923 ([M]$^+$, 100 %, calcd for $^{12}C_{100}{}^{1}H_{136}{}^{11}B_2{}^{14}N_4$ 1415.0954).

<u>HMBC Correlations:</u>

δ(^{1}H) = 8.288 ppm correlated to δ(^{13}C) = 156.44, 140.45, 131.50

δ(^{1}H) = 8.232 ppm correlated to δ(^{13}C) = 140.45, 130.38, 122.95

δ(^{1}H) = 7.898 ppm correlated to δ(^{13}C) = 156.44, 124.21

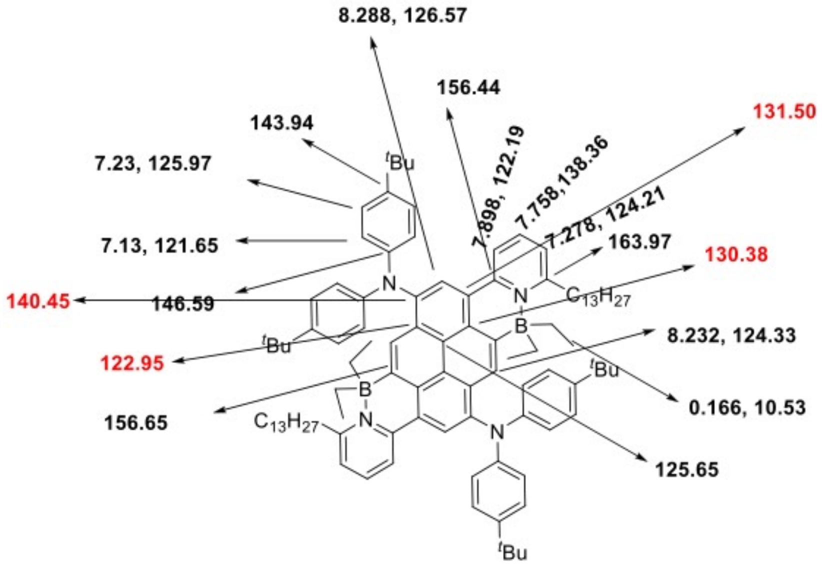

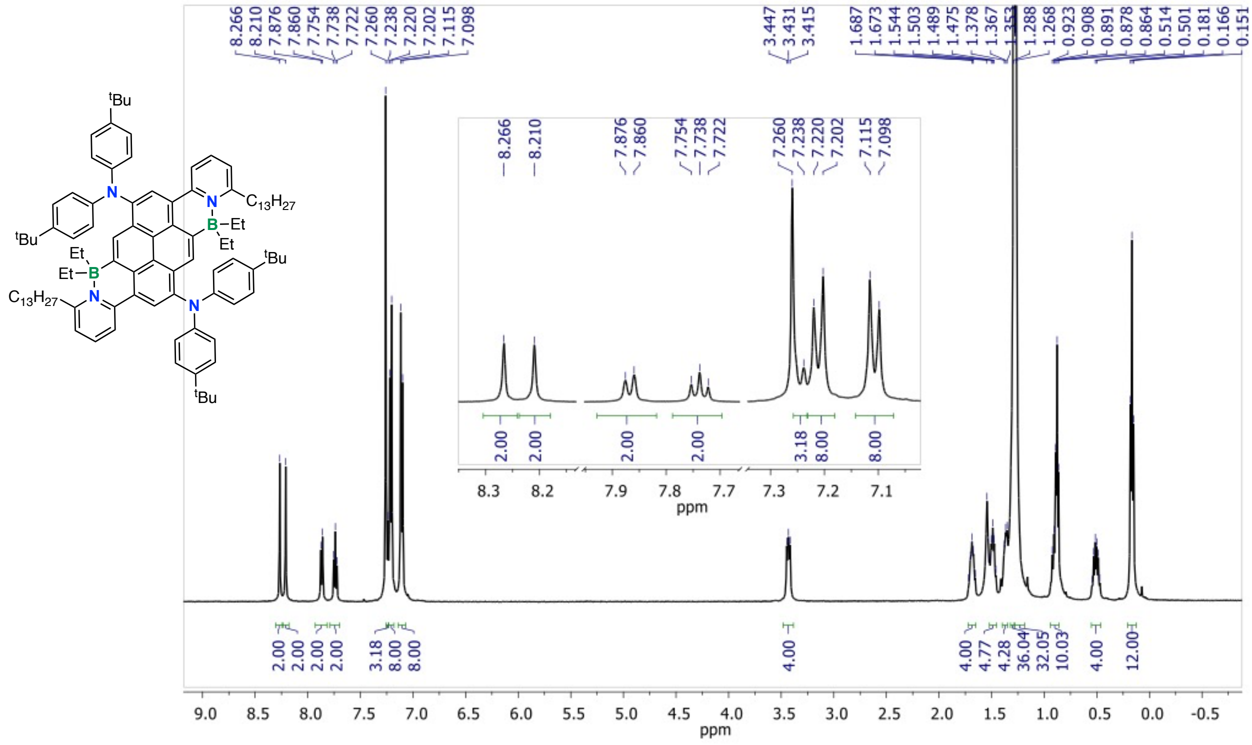

Figure S8. ^{1}H NMR spectrum and expansions of **5-Et** in $CDCl_3$

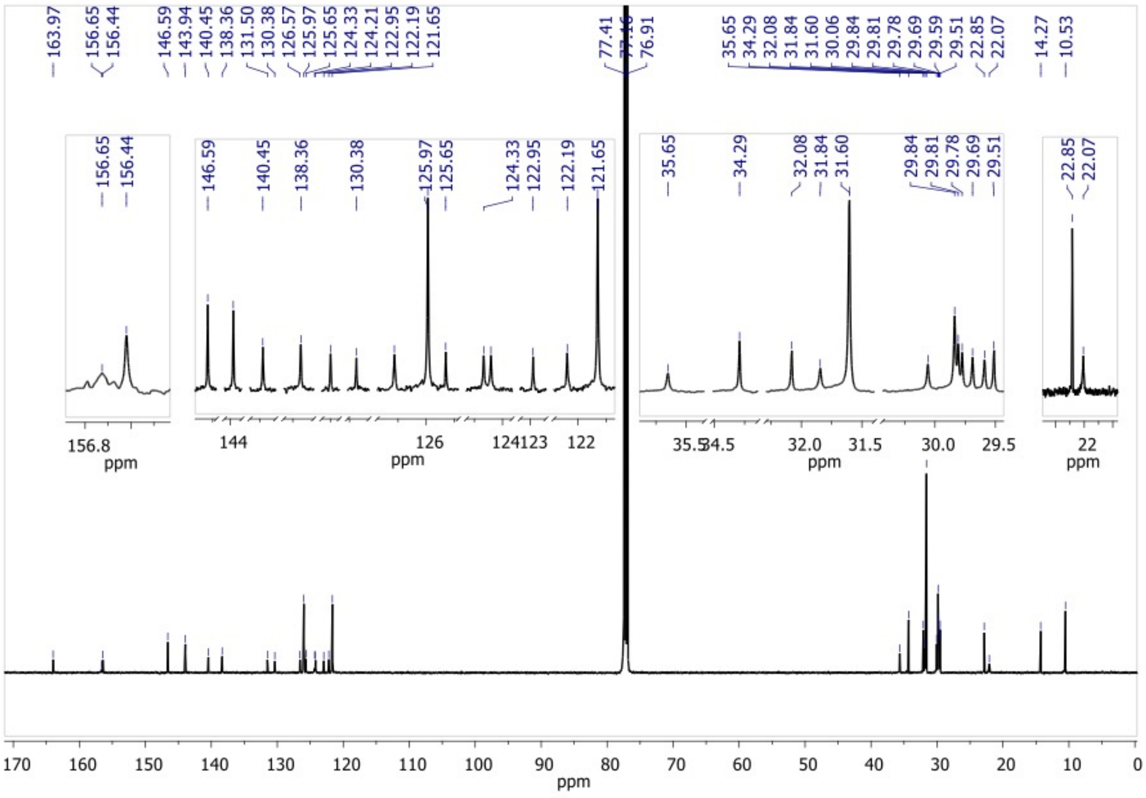

Figure S9. $^{13}C\{^1H\}$ NMR spectrum and expansions of **5-Et** in $CDCl_3$

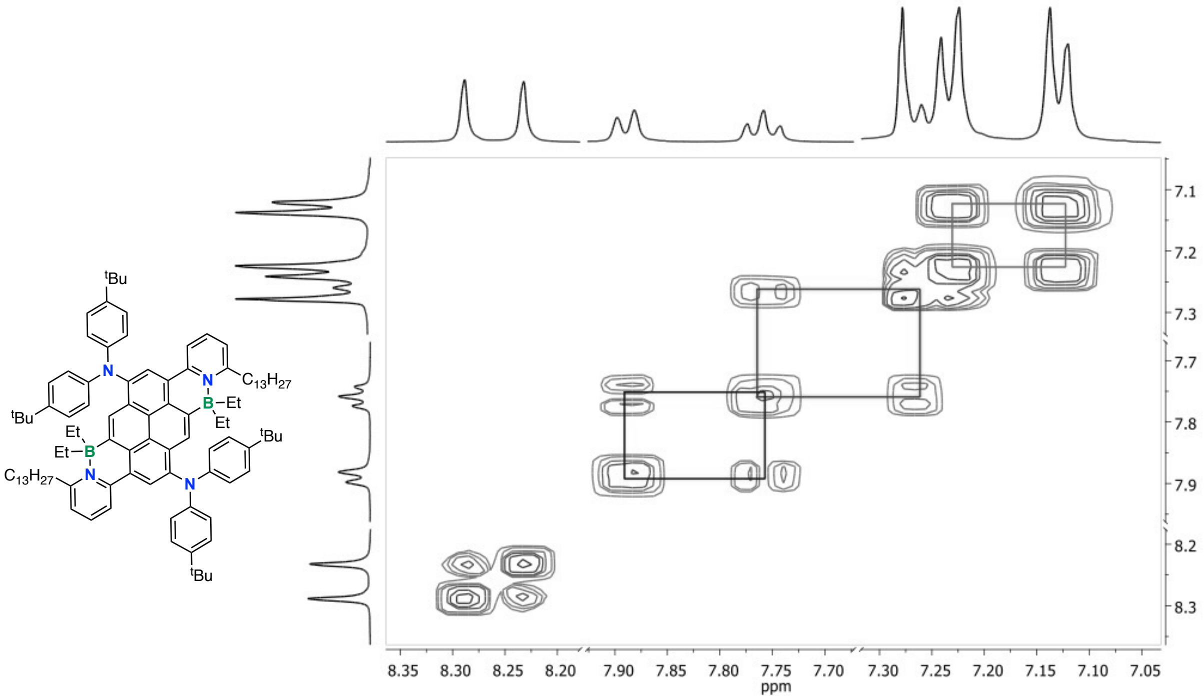

Figure S10. Aromatic region of the H,H-COSY NMR spectrum of **5-Et** in $CDCl_3$

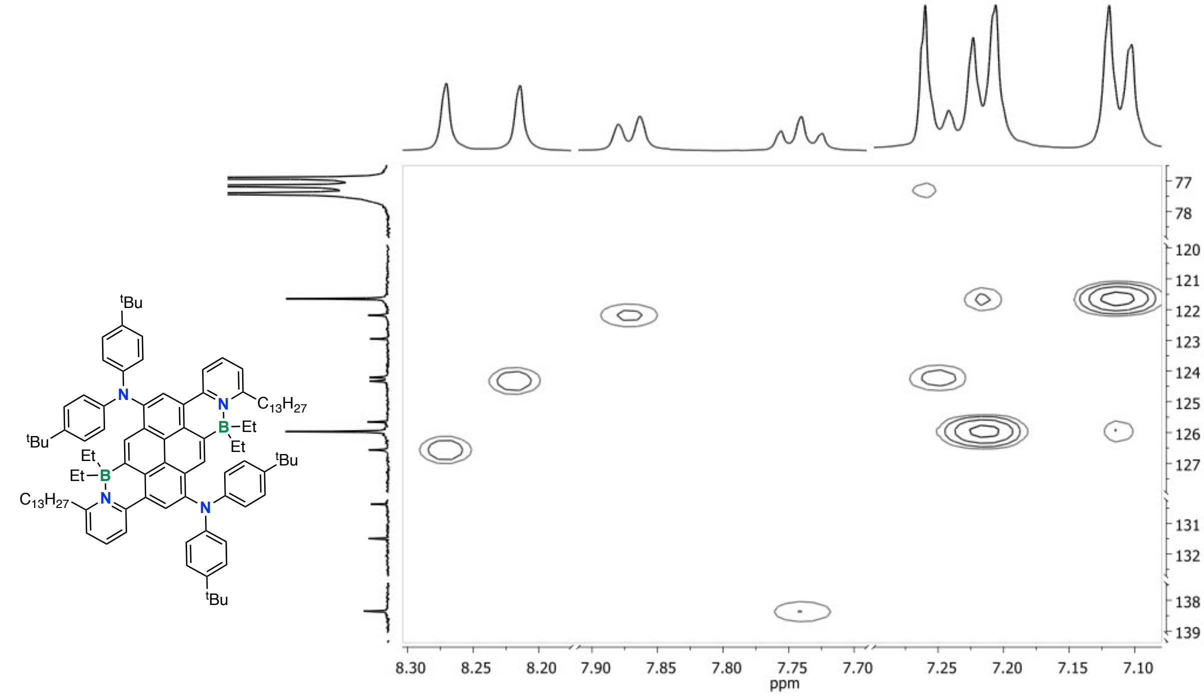

Figure S11a. Section of the HSQC NMR spectrum of **5-Et** in $CDCl_3$

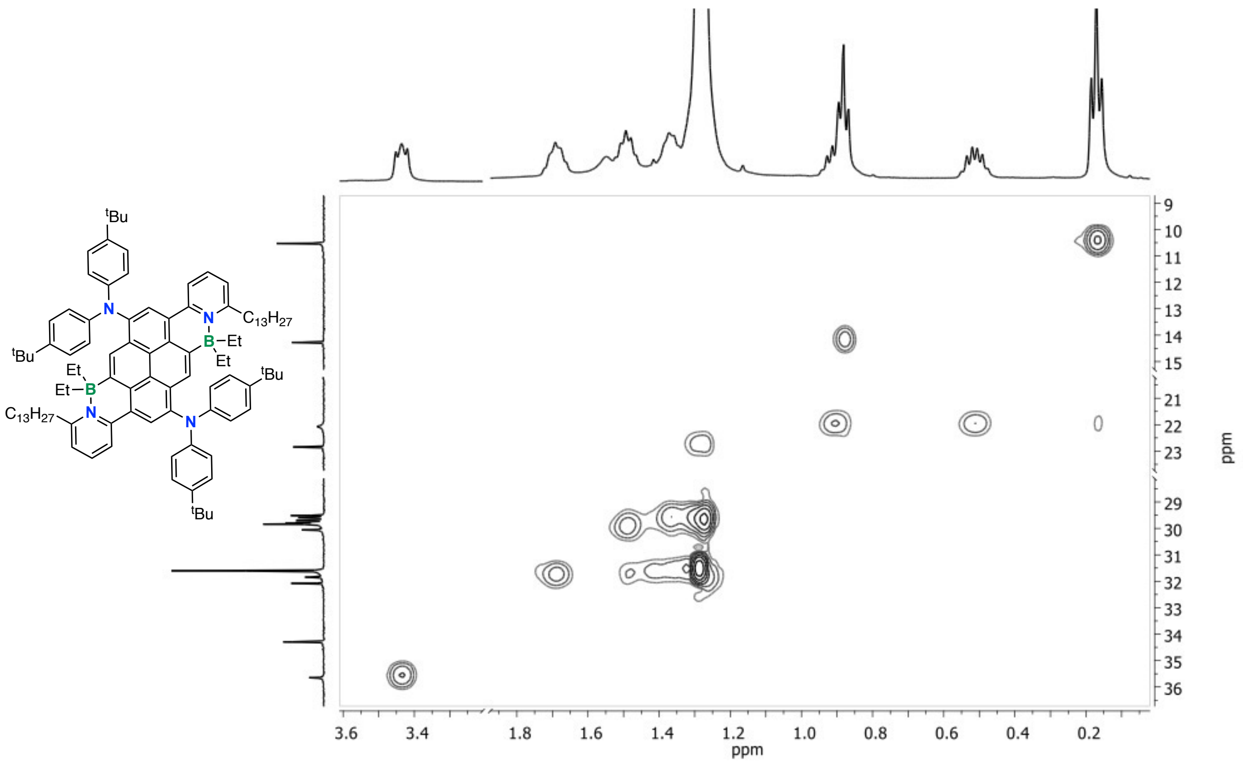

Figure S11b. Section of the HSQC NMR spectrum of **5-Et** in $CDCl_3$

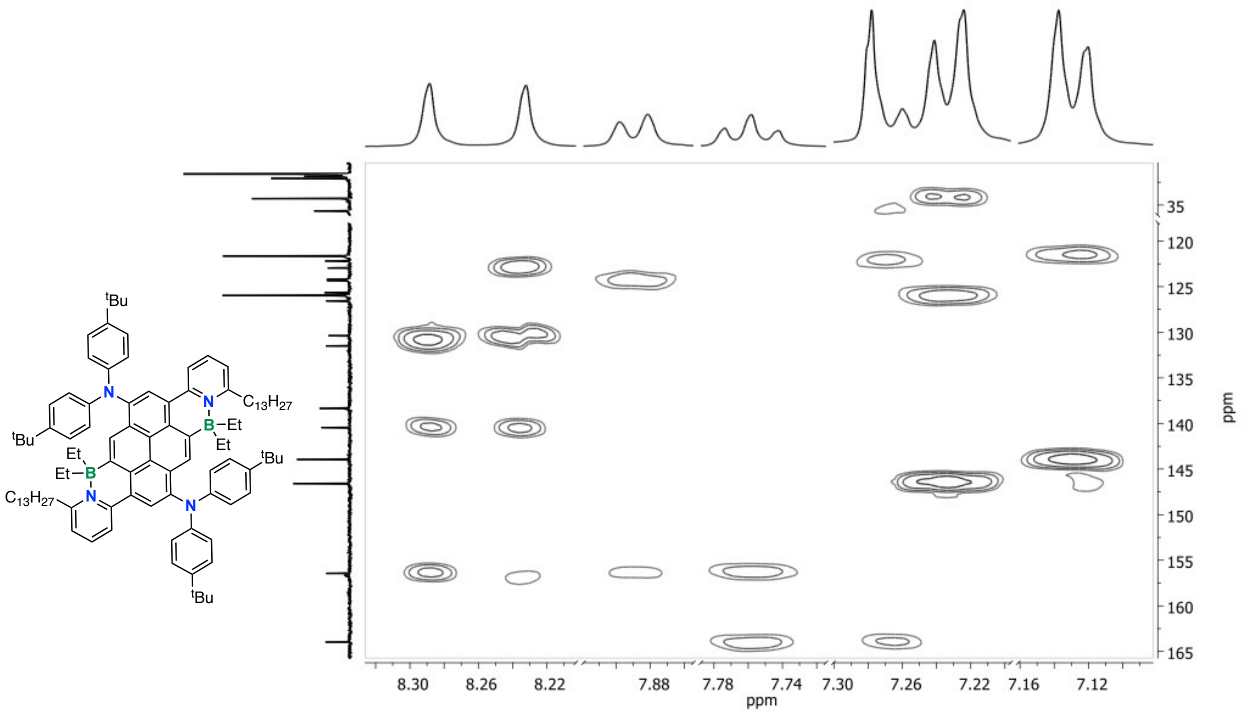

Figure S12a. Section of the HMBC NMR spectrum of **5-Et** in $CDCl_3$

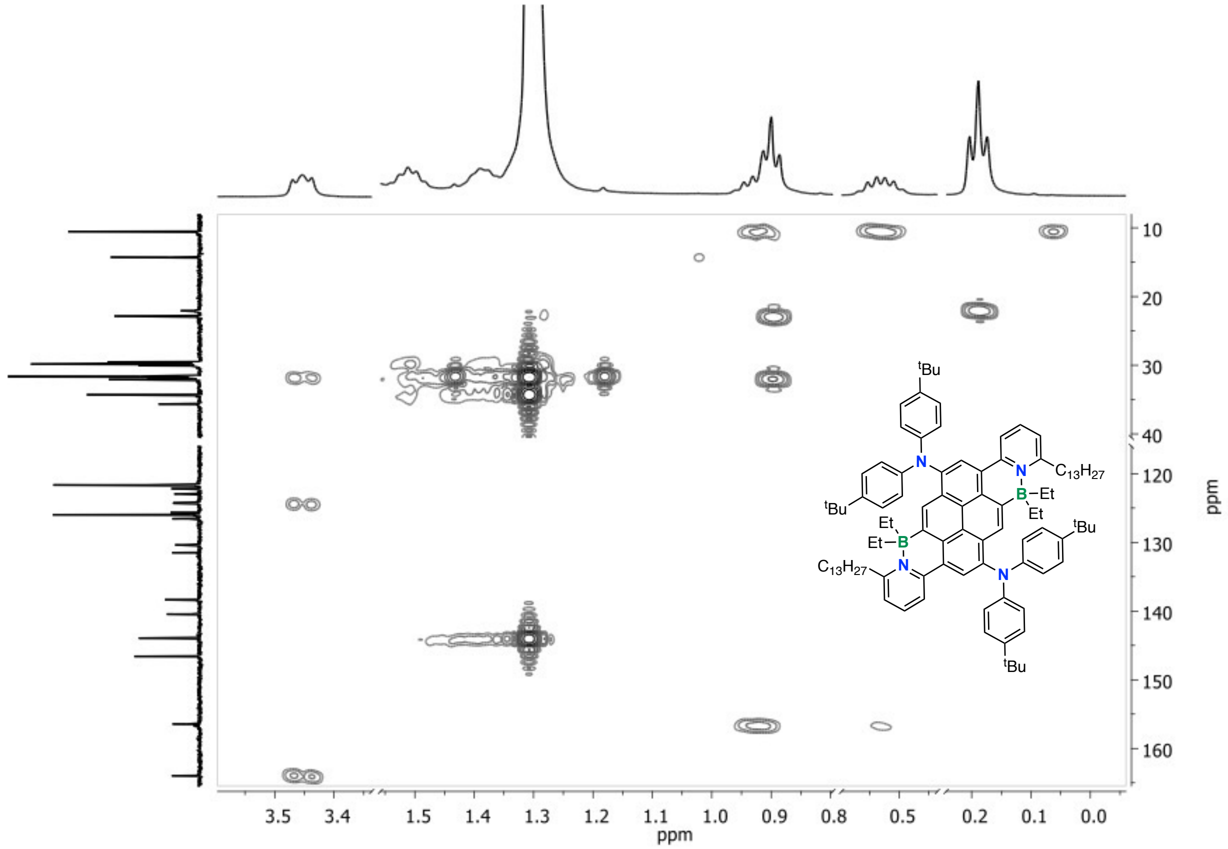

Figure S12b. Section of the HMBC NMR spectrum of **5-Et** in $CDCl_3$

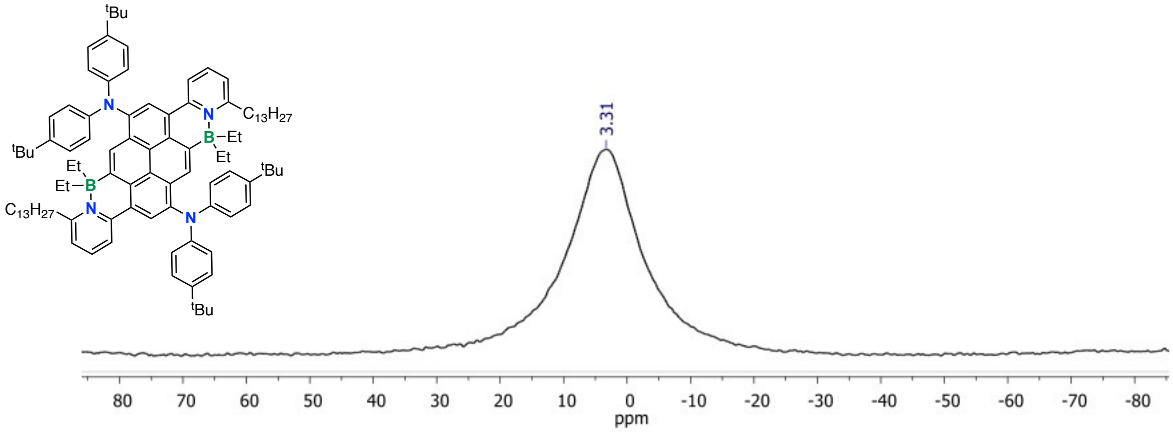

Figure S13. ^{11}B NMR spectrum of **5-Et** in $CDCl_3$

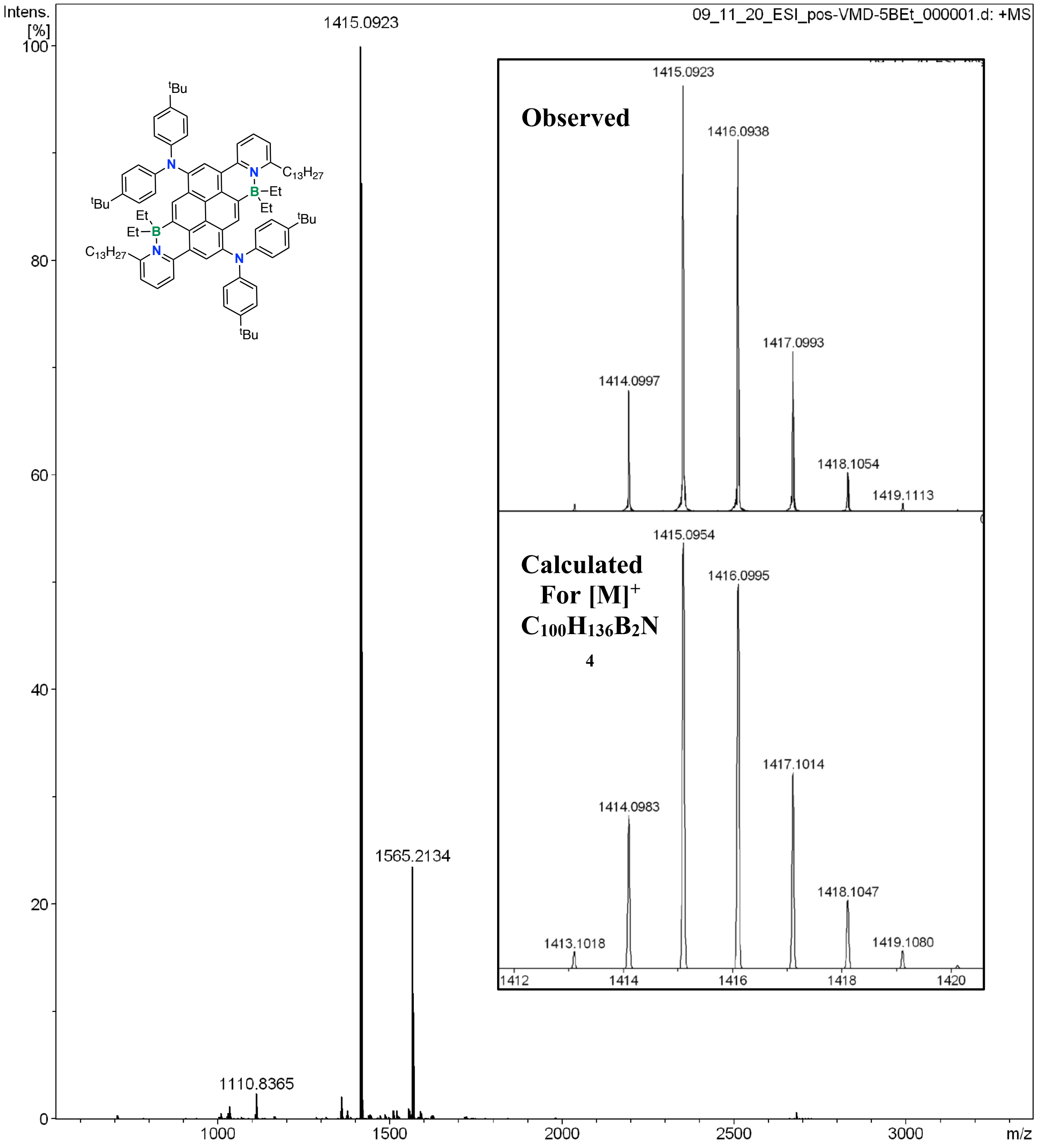

Figure S14. ESI-MS (pos. mode) of **5-Et** in THF

Synthesis of 5-Pf

In a glove box, to a solution of **L1** (0.250 g, 0.195 mmol) in anhydrous CH_2Cl_2 (40 mL) were added BCl_3 (0.80 mL, 0.80 mmol, 1M in dichloromethane), 2,6-di-*tert*-butylpyridine (90 µL, 0.40 mmol, 2 equiv) and $AlCl_3$ (107 mg, 0.80 mmol, 4 equiv) sequentially. After stirring at room temperature for 16 h, Bu_4NCl (117 mg, 0.40 mmol, 2 equiv) was added, the mixture was stirred for 1 h and then evaporated to dryness. The resultant residue was suspended in anhydrous toluene (30 mL) and then bis(perfluorophenyl)zinc (0.312 g, 0.78 mmol, 4 equiv) was added while stirring. The mixture was stirred for 24 h at room temperature. The reaction flask was then removed from the glove box and stirring continued for a further 2 h at 40 ºC. The mixture was filtered through a small plug of basic alumina and the residue rinsed with dichloromethane (30 mL). The filtrate was concentrated under reduced pressure and the residue was subjected to a base-treated silica gel column chromatography using hexanes and ethyl acetate as eluent (9:2). The product was obtained as a dark red colored solid. Yield: 0.19 g (50%).

^{1}H NMR (600 MHz, $CDCl_3$): δ = 8.30 (s, 2H, Pyr), 8.27 (s, 2H, Pyr), 7.94 (t, J = 7.9 Hz, 2H, Py), 7.84 (d, J = 7.9 Hz, 2H, Py), 7.36 (dd, J = 7.6, 0.8 Hz, 2H, Py), 7.17 (d, J = 8.8 Hz, 8H, ^{t}BuPh), 6.85 (d, J = 8.7 Hz, 8H, ^{t}BuPh), 2.87 (t, J = 7.0 Hz, 4H), 1.31 (s, 36H, ^{t}Bu), 1.27 -1.22 (m, 30H), 1.12 – 1.16 (m, 14H), 0.89 (t, J = 7.0 Hz, 6H). ^{13}C{^{1}H} NMR (151 MHz, $CDCl_3$): δ = 163.92, 154.67, 148.27 (br d, ^{1}J(^{13}C, ^{19}F) = 241 Hz, Pf), 146.37 (br, B-C_{Pyr}), 146.12, 144.57, 142.03, 140.70, 139.69 (br d, ^{1}J(^{13}C, ^{19}F) = 251 Hz, Pf), 137.14 (br d, ^{1}J(^{13}C, ^{19}F) = 258 Hz, Pf), 133.04, 127.72, 127.53, 126.85, 125.96, 124.45, 123.68, 123.27, 121.40, 35.17, 34.30, 32.07, 31.49, 29.77, 29.69, 29.50, 29.44, 29.38, 22.85, 14.27, 1.19 ppm. ^{11}B NMR (160 MHz, $CDCl_3$): δ = -4.6 ppm. ^{19}F NMR (471 MHz, $CDCl_3$): δ = -130.97 (8F, *o*-Pf), 157.48 (4F, *p*-Pf), -163.44 (8F, *m*-Pf) ppm. HRMS (APCI in toluene, pos. mode) m/z = 1966.9106 ([M]$^{+}$, 100 %, calcd for $^{12}C_{116}{}^{1}H_{116}{}^{11}B_2{}^{19}F_{20}{}^{14}N_4$ 1966.9069).

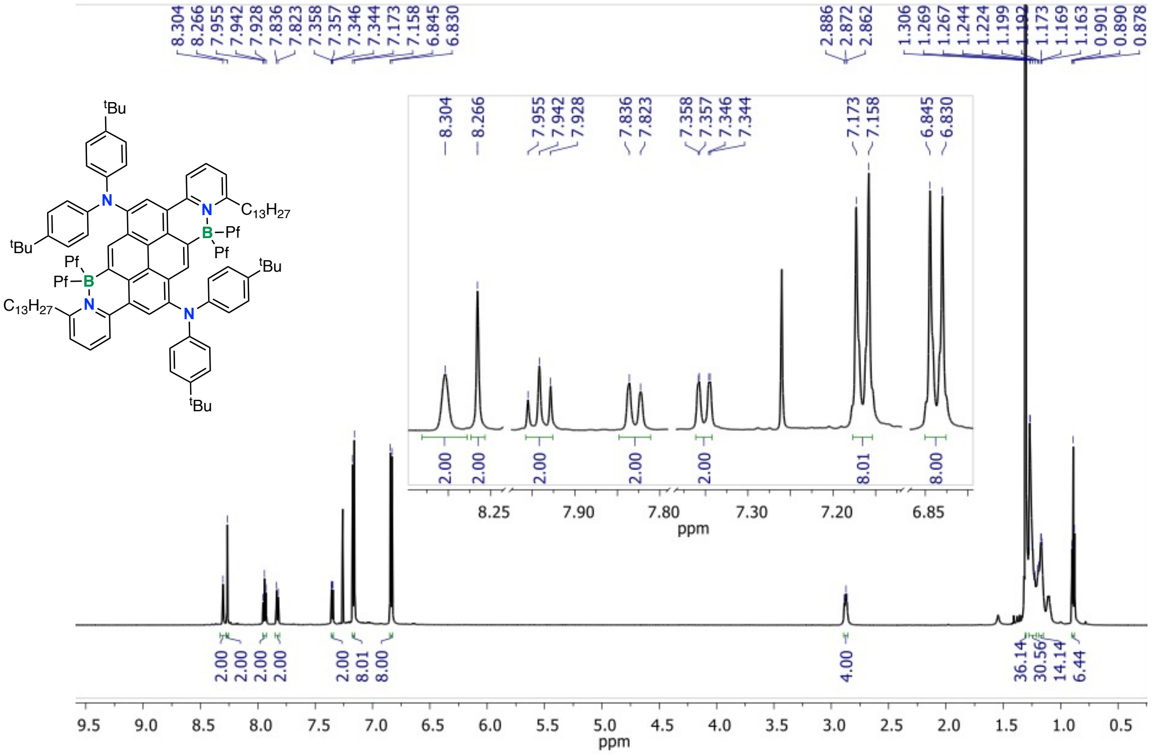

Figure S15. ^{1}H NMR spectrum and expansion of **5-Pf** in $CDCl_3$

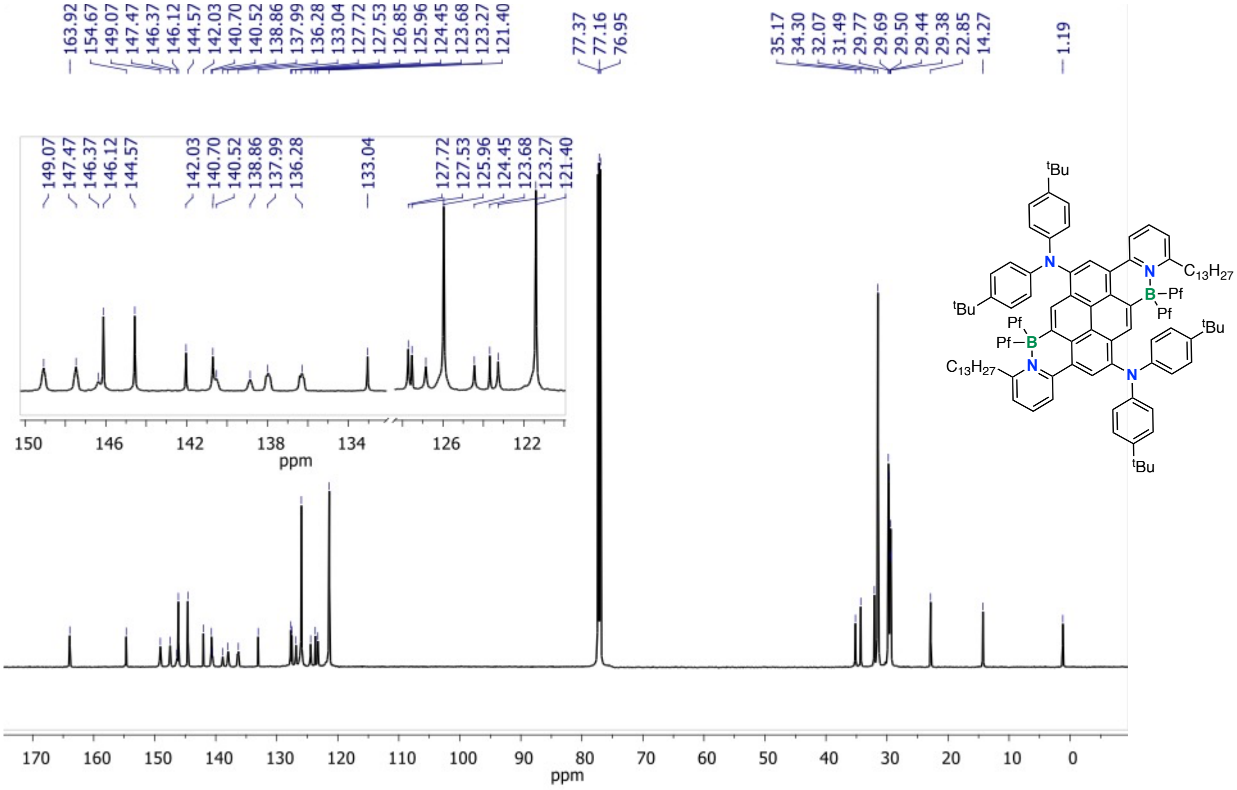

Figure S16. $^{13}C\{^{1}H\}$ NMR spectrum and expansion of **5-Pf** in $CDCl_3$

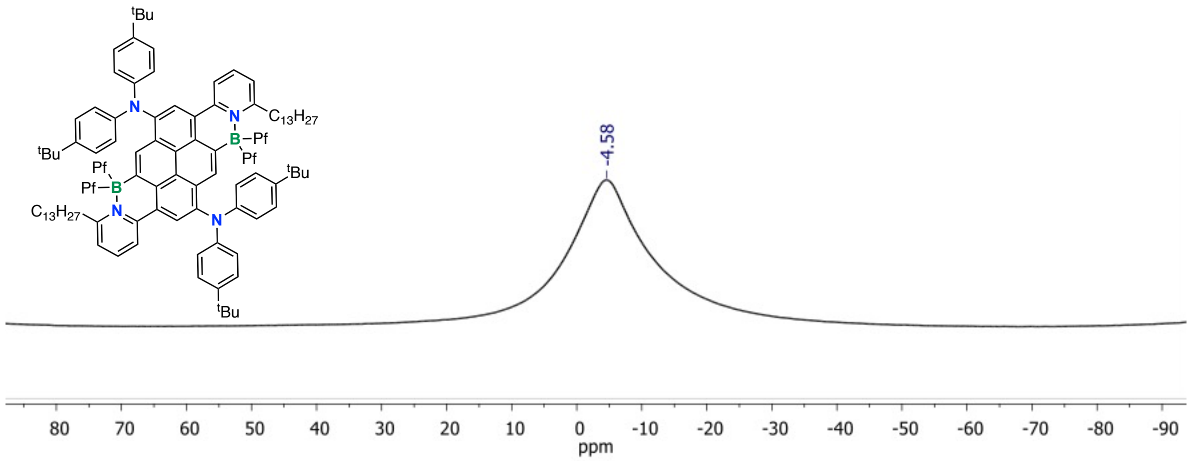

Figure S17. ^{11}B NMR spectrum of **5-Pf** in $CDCl_3$

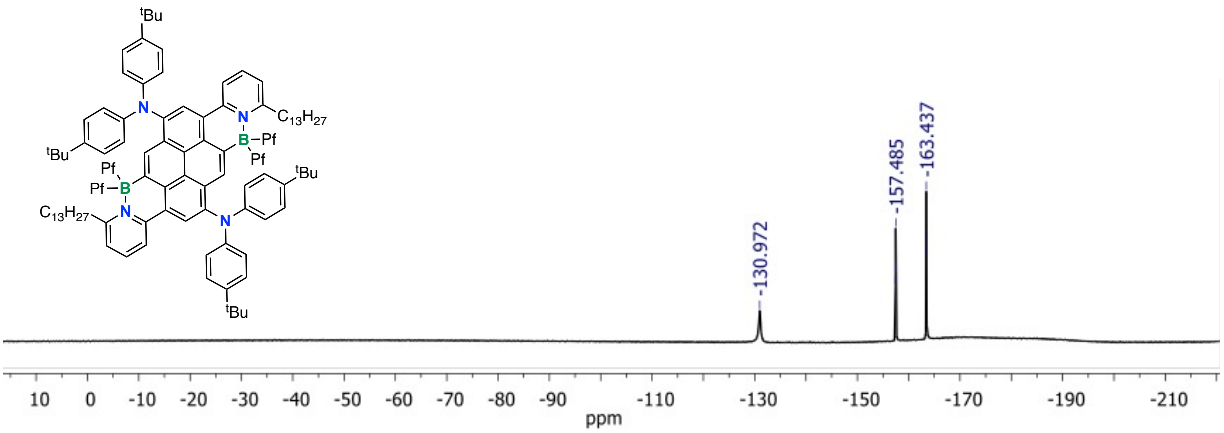

Figure S18. ^{19}F NMR spectrum of **5-Pf** in $CDCl_3$

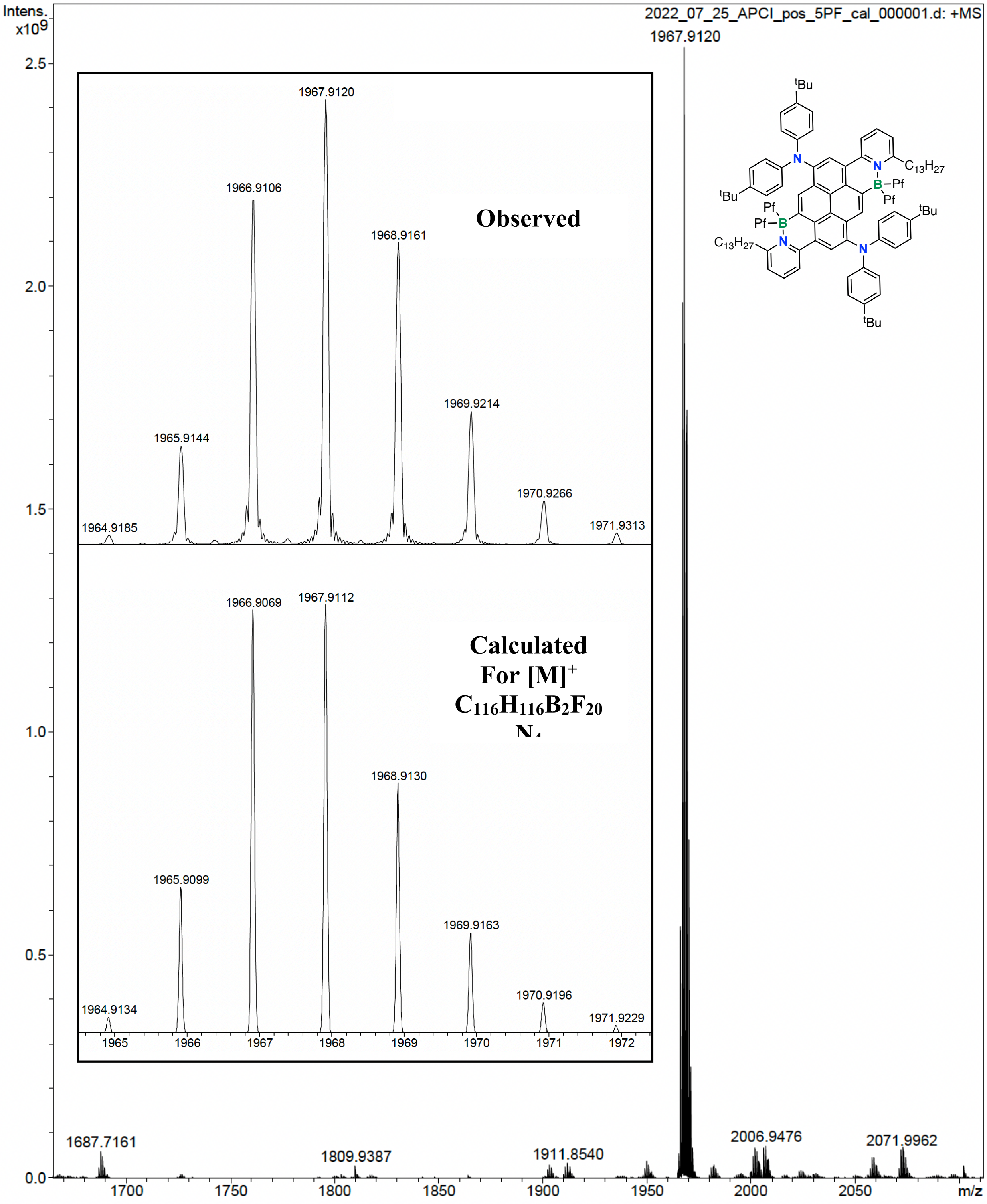

Figure S19. APCI-MS (pos. mode) of **5-Pf** in toluene

Synthesis of 4,4'-(3,8-bis(6-tridecylpyridin-2-yl)pyrene-1,6-diyl)bis(N,N-bis(4-(tert-butyl)phenyl)aniline) (L2)

In a glove box, **3** (1.20 g, 1.37 mmol), **4** (1.65 g, 3.41 mmol, 2.5 equiv), cesium fluoride (622 mg, 4.10 mmol), cesium carbonate (1.33 g, 4.10 mmol) and $Pd(PPh_3)_4$ (158 mg, 137 µmol, 10 mol%) were taken in a 200 mL Schlenk flask. After removing the flask from the glovebox, 90 mL of a degassed mixture of toluene:dmf (60:30, v/v) were added under positive N_2 gas flow. The reaction mixture was stirred at 90 °C for 24 h, cooled to rt and the solvent was removed by rotary evaporation. 100 mL of toluene and one spatula of charcoal were added, the mixture was heated to 80 °C, then quickly passed through a small plug of silica gel and celite and rinsed with hot toluene (3 x 30 mL). The solvent was removed under reduced pressure and the resultant residue was recrystallized from chloroform and methanol solution at -20 °C to give the desired product as a yellow solid. Yield: 1.47 g (75%).

^{1}H NMR (500 MHz, $CDCl_3$): δ = 8.33 (d, J = 10 Hz, 2H, Pyr), 8.30 (d, J = 10 Hz, 2H, Pyr), 8.16 (s, 2H, Pyr), 7.77 (t, J = 7.7 Hz, 2H, Py), 7.56-7.52 (m, 6H, Py, NPh), 7.30 (d, J = 8.6 Hz, 8H, ^{t}BuPh), 7.24-7.21 (m, 6H, Py, NPh), 7.13 (d, J = 8.6 Hz, 8H, ^{t}BuPh), 2.95 (t, J = 8.0 Hz, 4H), 1.86 (p, 4H), 1.46 (p, 4H), 1.40-1.36 (m, 2H), 1.36 (s, 36H), 1.29 – 1.21 (m, 32H), 0.86 (t, J = 6.9 Hz, 6H). ^{13}C{^{1}H} NMR (126 MHz, $CDCl_3$) δ = 162.77, 159.13, 147.51, 145.86, 145.22, 137.38, 136.54, 135.87, 134.51, 131.54, 129.55, 128.98, 128.20, 126.34, 126.22, 125.73, 125.40, 124.23, 123.16, 122.75, 120.89, 38.93, 34.45, 32.06, 31.61, 30.36, 29.84, 29.82, 29.80, 29.72, 29.69, 29.50, 22.83, 14.27 ppm. HRMS (ESI in THF, pos. mode) m/z = 1430.9953 ($[M]^+$, calcd for $^{12}C_{104}{}^{1}H_{126}{}^{14}N_4$ 1430.9977).

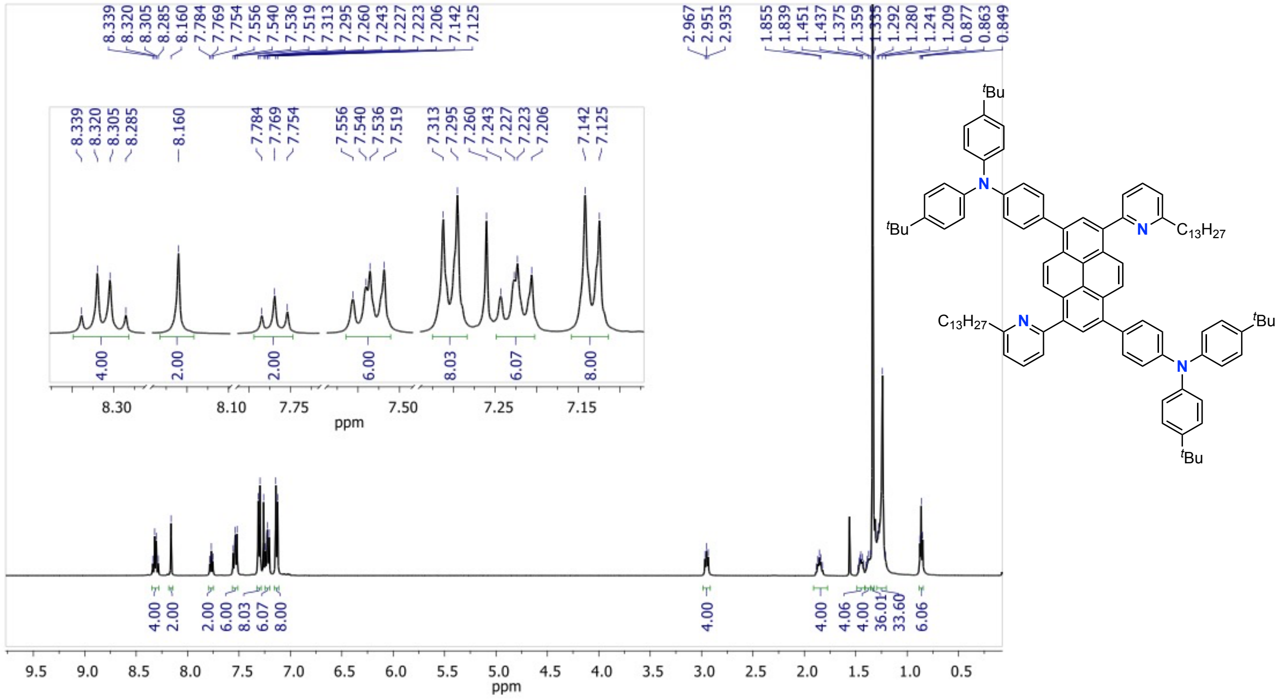

Figure S20. ^{1}H NMR spectrum and expansions of **L2** in $CDCl_3$

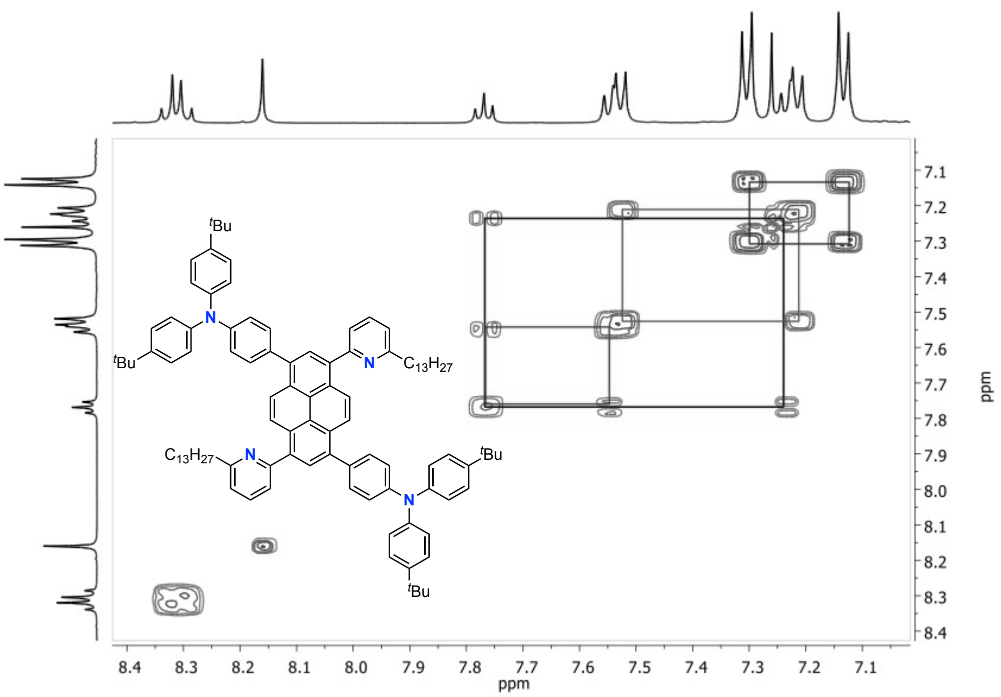

Figure S21. Aromatic region of the H,H-COSY NMR spectrum of **L2** in $CDCl_3$

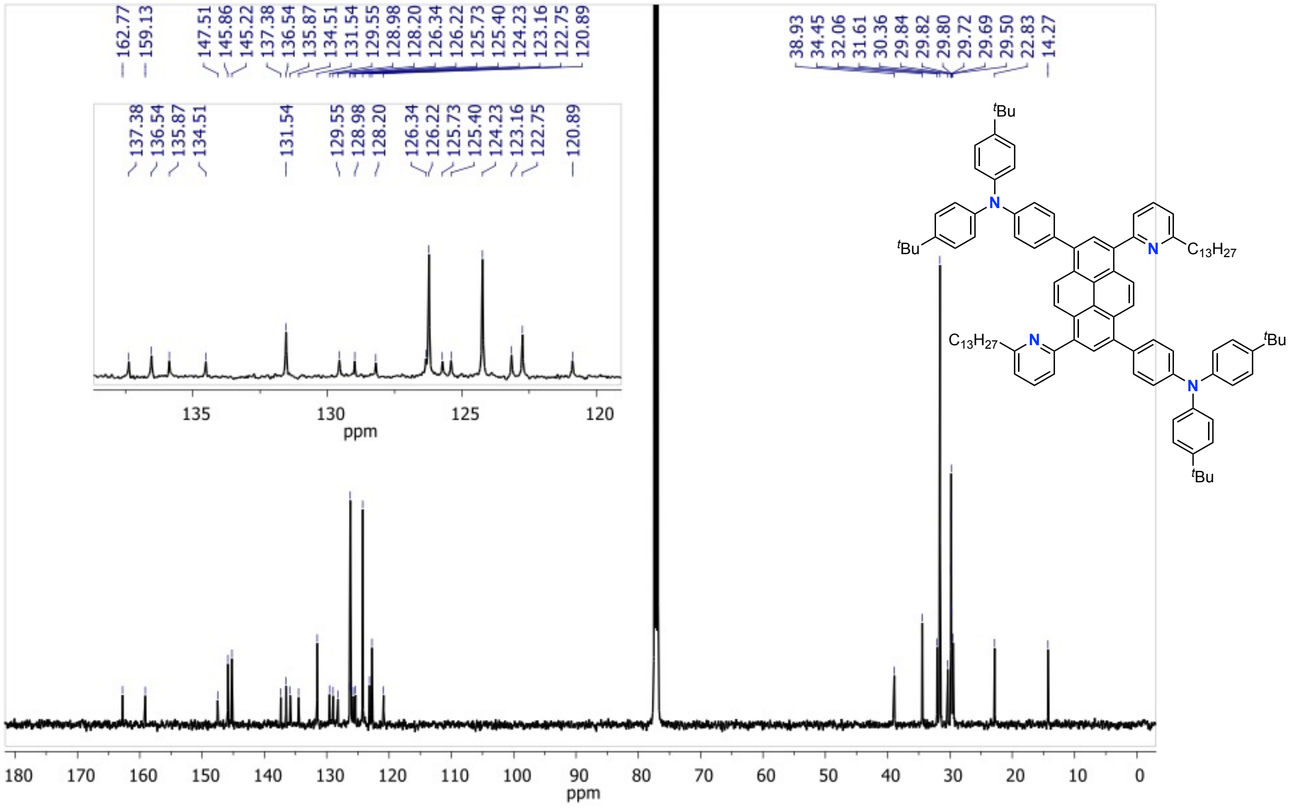

Figure S22. $^{13}C\{^1H\}$ NMR spectrum and expansion of **L2** in $CDCl_3$

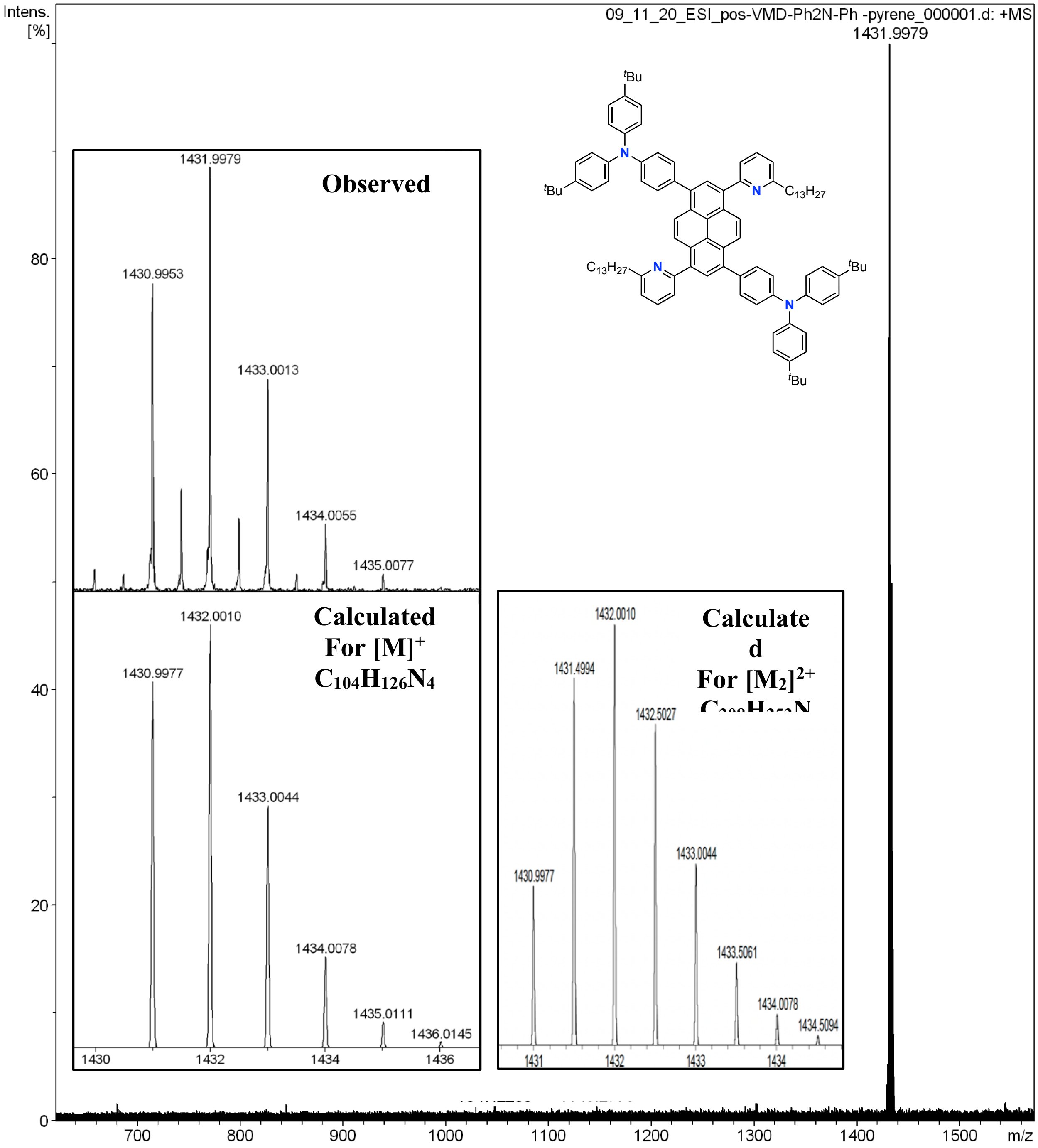

Figure S23. ESI-MS (pos. mode) of **L2** in THF; smaller additional peaks are tentatively assigned to doubly charged $[M_2]^{2+}$ ions that overlap with the dominant $[M]^+$ ions

Synthesis of 6-Et

In a glove box, to a solution of **L2** (0.390 g, 0.272 mmol) in anhydrous CH_2Cl_2 (60 mL) were added BCl_3 (1.1 mL, 1.1 mmol, 1M in dichloromethane, 4 equiv), 2,6-di-*tert*-butylpyridine (122 µL, 0.545 mmol, 2 equiv) and $AlCl_3$ (145 mg, 1.09 mmol, 4 equiv) sequentially. After stirring at room temperature for 16 h, Bu_4NCl (159 mg, 0.545 mmol, 2 equiv) was added and the mixture allowed to stir for 15 min. Then Et_2Zn (112 µL, 1.09 mmol, 4 equiv) was added neat and the mixture was stirred for a further 16 h at room temperature. The reaction flask was removed from the glove box and the mixture was filtered through a small plug of silica gel. The solvent was removed under reduced pressure and the resultant residue was rinsed first with methanol and then cold pentane solution to give the desired product as an orange-red solid. Yield: 0.36 (84%).

^{1}H NMR (500 MHz, $CDCl_3$): δ = 8.48 (s, 2H, Pyr), 8.42 (s, 2H, Pyr), 8.28 (d, *J* = 7.9 Hz, 2H, Py), 7.87 (t, *J* = 7.9 Hz, 2H, Py), 7.65 (d, *J* = 8.5 Hz, 4H, Ph-Pyr), 7.33 (d, *J* = 8.6 Hz, 10H, PhN, Py), 7.29 (d, *J* = 8.5 Hz, 4H, Ph-Pyr), 7.19 (d, *J* = 8.7 Hz, 8H, PhN), 3.54 (t, *J* = 8.3 Hz, 4H), 1.80 – 1.73 (m, 4H), 1.58 – 1.52 (m, 4H), 1.44 – 1.41 (m, 4H), 1.35 (s, 36H, ^{t}Bu), 1.32 – 1.27 (m, 16H), 1.18 – 1.11 (m, 4H, BC$\underline{H_2}$CH$_3$), 0.87 (t, *J* = 6.8 Hz, 6H, methyl groups of tridecyl chains), 0.84 – 0.82 (m, 4H, BC$\underline{H_2}$CH$_3$), 0.37 (t, *J* = 7.5 Hz, 12H, BCH$_2$C$\underline{H_3}$) ppm. ^{13}C{^{1}H} NMR (126 MHz, $CDCl_3$): δ =163.97 (Py), 157.14, 156.82, 147.31, 145.77, 145.35, 138.43, 136.41, 135.34, 132.39, 131.54, 131.35, 126.81, 126.23, 126.15, 124.32, 122.73, 122.11, 121.54, 35.77, 34.47, 32.08, 31.96, 31.65, 30.17, 29.85, 29.84, 29.81, 29.79, 29.73, 29.66, 29.51, 22.84, 22.61, 14.27, 10.75 ppm. ^{11}B NMR (160 MHz, $CDCl_3$): δ = 2.9 ppm. HRMS (ESI in THF, pos. mode) *m*/*z* = 1567.1582 ([M]$^{+}$, 100 %, calcd for $^{12}C_{112}{}^{1}H_{144}{}^{11}B_2{}^{14}N_4$ 1567.1580).

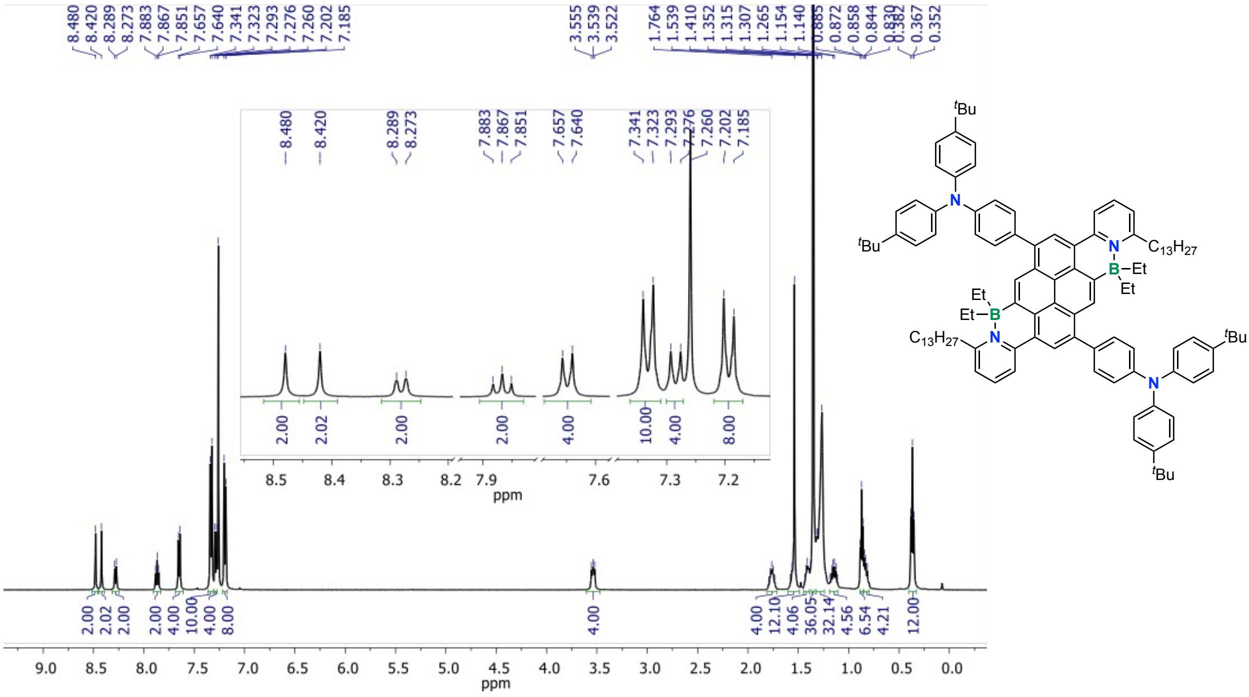

Figure S24. ^{1}H NMR spectrum and expansions of **6-Et** in $CDCl_3$

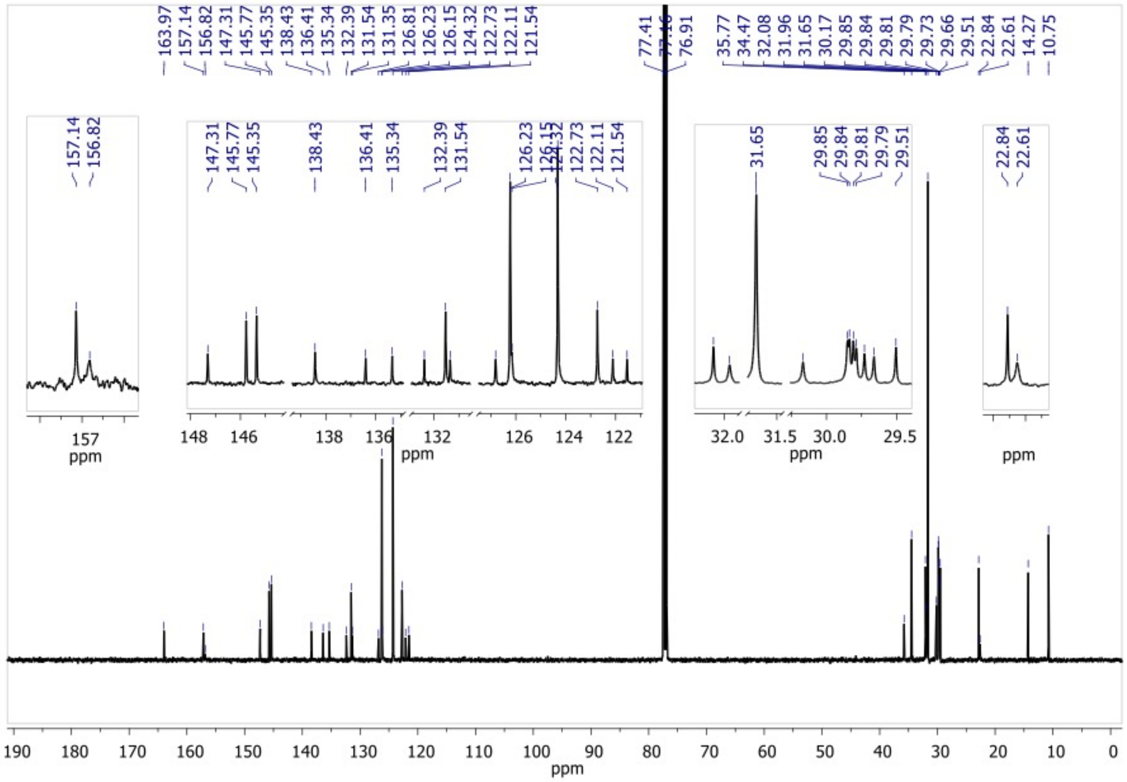

Figure S25. $^{13}C\{^1H\}$ NMR spectrum and expansions of **6-Et** in $CDCl_3$

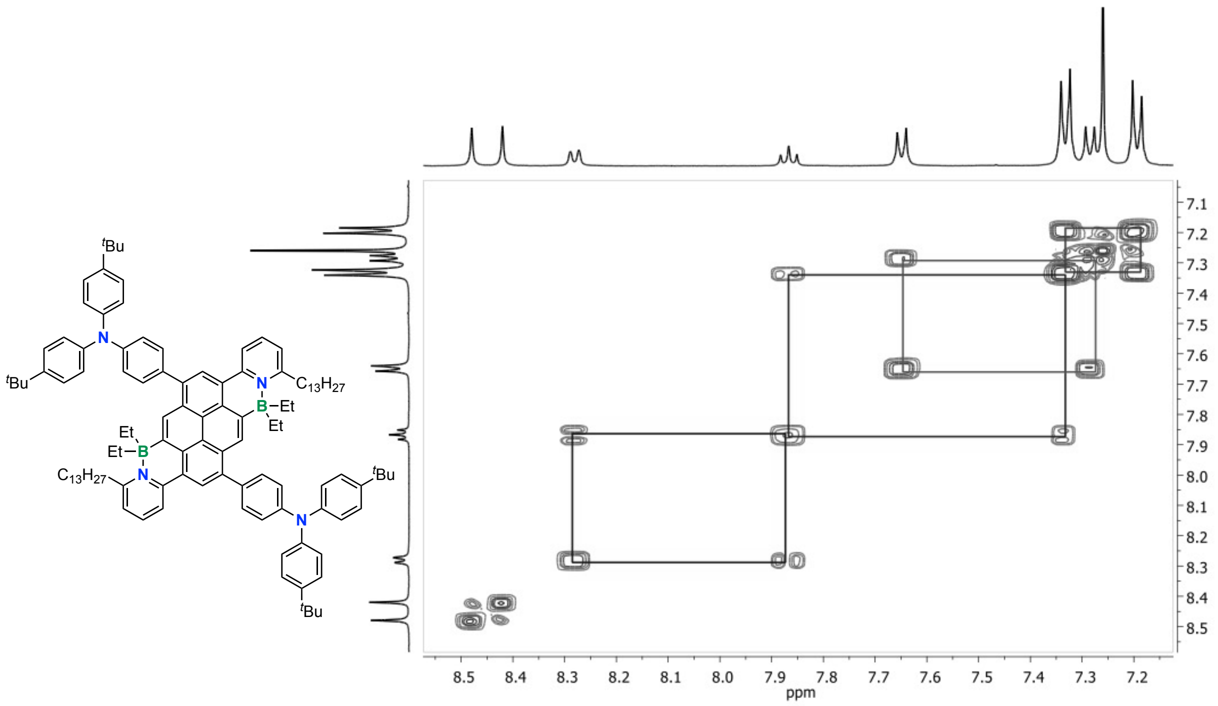

Figure S26. Aromatic region of the H,H-COSY NMR spectrum of **6-Et** in $CDCl_3$

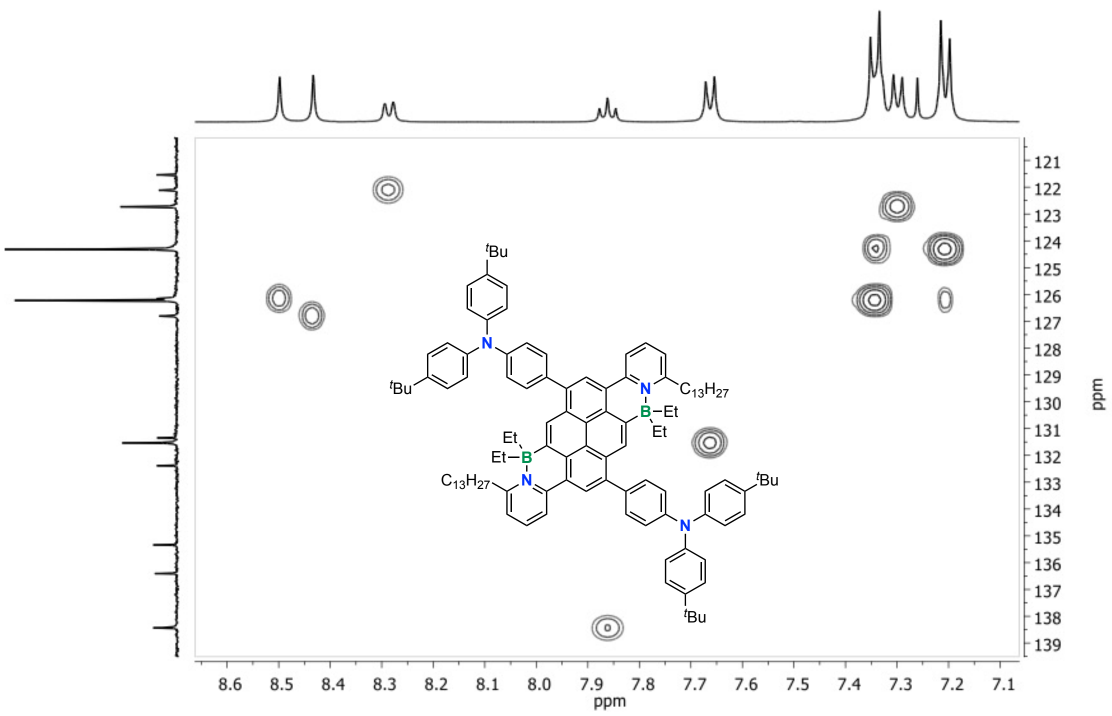

Figure S27a. Section of the HSQC NMR spectrum of **6-Et** in $CDCl_3$ (aromatic region)

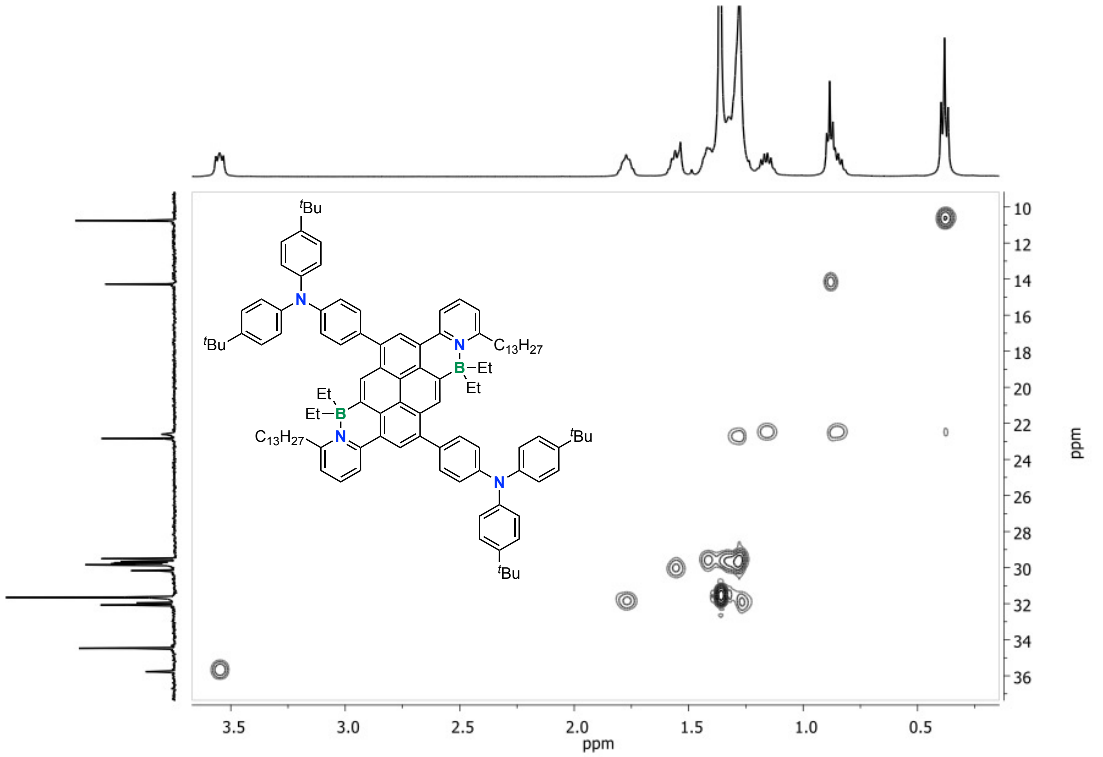

Figure S27b. Section of the HSQC NMR spectrum of **6-Et** in $CDCl_3$ (aliphatic region)

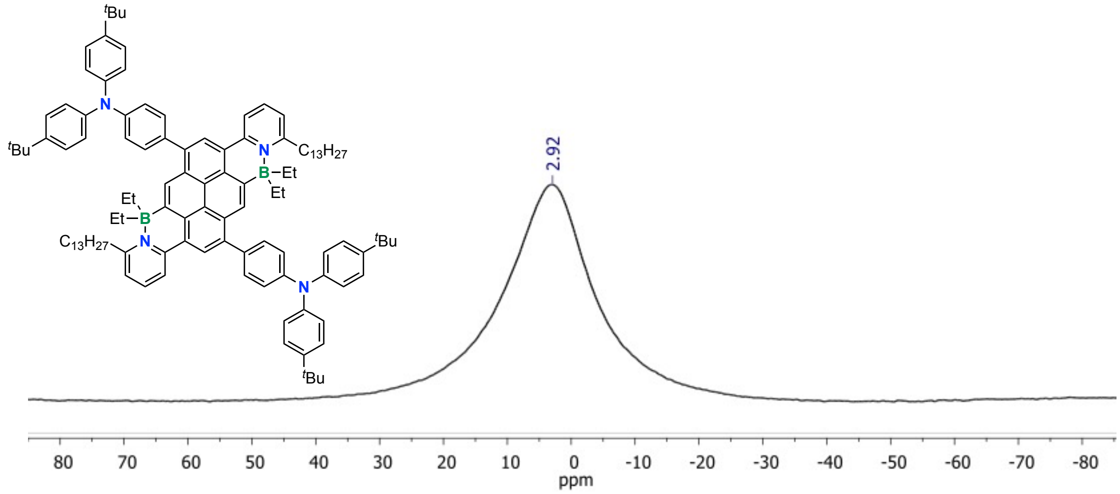

Figure S28. ^{11}B NMR spectrum of **6-Et** in $CDCl_3$

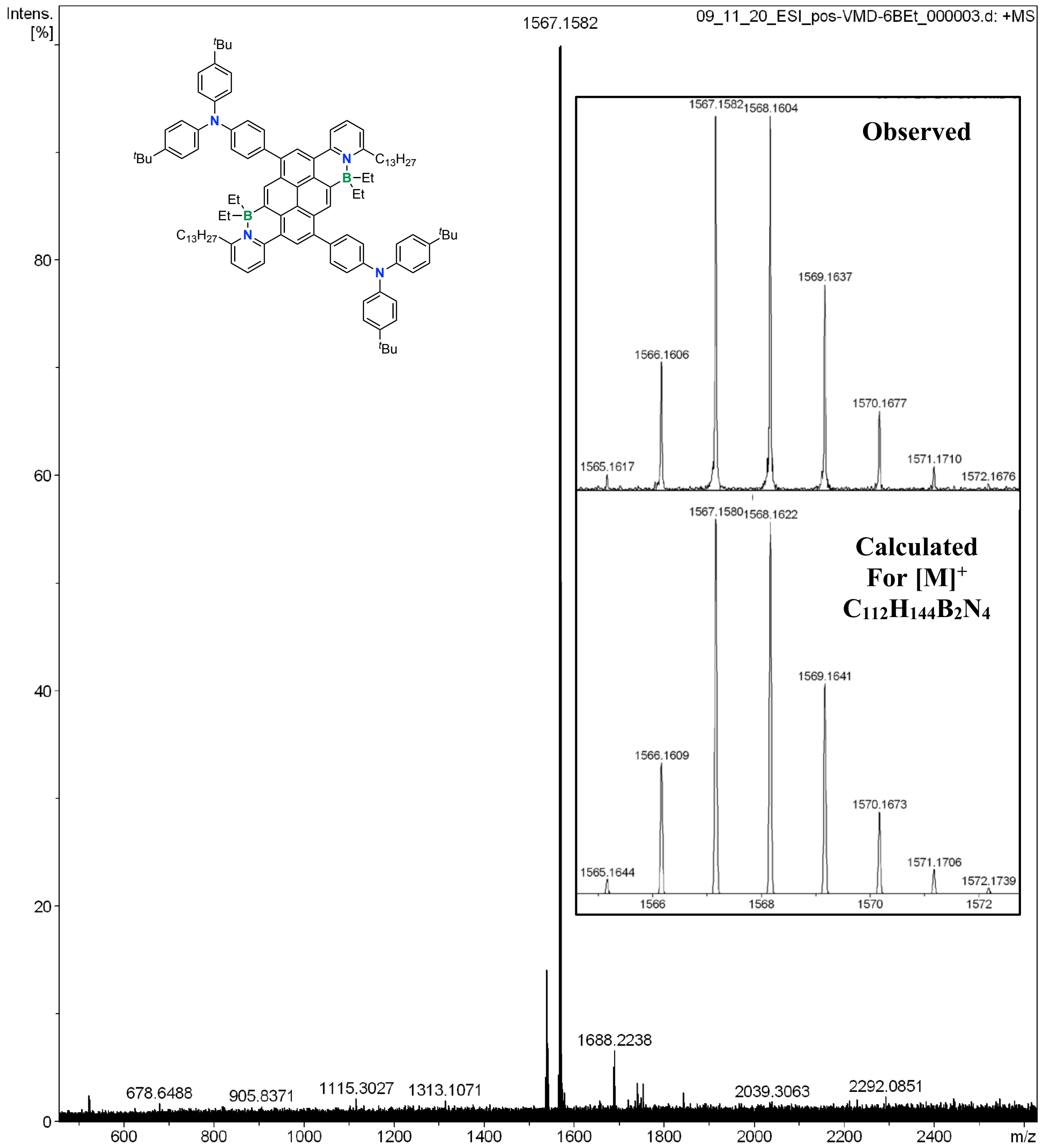

Figure S29. ESI-MS (pos. mode) of **6-Et** in THF

Synthesis of 6-Pf

In a glove box, to a solution of **L2** (300 mg, 0.209 mmol) in anhydrous CH_2Cl_2 (50 mL) were added BCl_3 (0.84 mL, 0.84 mmol, 1M in dichloromethane, 4 equiv), 2,6-di-*tert*-butylpyridine (95 μL, 0.42 mmol, 2 equiv) and $AlCl_3$ (112 mg, 0.84 mmol, 4 equiv) sequentially. After stirring at room temperature for 16 h, Bu_4NCl (123 mg, 0.42 mmol, 2 equiv) was added, the mixture was stirred for 1 h and then evaporated to dryness. The resultant residue was suspended in anhydrous toluene (30 mL) and then bis(perfluorophenyl)zinc (330 mg, 0.84 mmol, 4 equiv) was added while stirring. The mixture was stirred for 24 h at room temperature. The reaction flask was then removed from the glove box and stirring continued for a further 2 h at 40 ºC. The mixture was filtered through a small plug of basic alumina and the residue rinsed with dichloromethane (30 mL). The filtrate was concentrated under reduced pressure and the residue was subjected to a base-treated silica gel column chromatography using hexanes and ethyl acetate as eluent (9:2). The product was obtained as a red colored solid. Yield: 0.27 g (61%).

^{1}H NMR (600 MHz, $CDCl_3$): δ = 8.43 (s, 2H, Pyr), 8.22 (d, *J* = 7.9 Hz, 2H, Py), 8.18 (br s, 2H, Pyr), 8.04 (t, *J* = 7.9 Hz, 2H, Py), 7.42 (d, *J* = 7.6 Hz, 2H, Py), 7.39 (d, *J* = 8.6 Hz, 8H, PhN), 7.36 (d, *J* = 8.4 Hz, 4H, Ph-Pyr), 7.15 – 7.13 (m, 12H, PhN, Ph-Pyr), 2.95 (t, *J* = 7.2 Hz, 4H), 1.38 (s, 36H, ^{t}Bu), 1.27 (br s, 34H), 1.22 (br s, 10H), 0.88 (t, *J* = 6.9 Hz, 6H, methyl groups of tridecyl chains). ^{13}C{^{1}H} NMR (151 MHz, $CDCl_3$): δ = 163.82, 155.37, 148.40 (br d, $^1J(^{13}C, ^{19}F)$ = 243 Hz, Pf), 148.02, 146.08, 145.14, 140.59, 139.62 (br d, $^1J(^{13}C, ^{19}F)$ = 254 Hz, Pf), 138.43, 137.40 (br d, $^1J(^{13}C, ^{19}F)$ = 251 Hz, Pf), 133.13, 131.93, 131.16, 128.88, 128.03, 126.84, 126.36, 124.41, 124.26, 123.40, 121.71 (br m, i-Pf), 122.39, 122.25, 35.29, 34.50, 32.08, 31.61, 29.81, 29.75, 29.52, 29.46, 22.85, 14.28. ^{11}B NMR (160 MHz, $CDCl_3$): δ = -4.7 ppm. ^{19}F NMR (471 MHz, $CDCl_3$): δ = -130.97 (8F, *o*-Pf), -157.53 (4F, *p*-Pf), -163.80 (8F, *m*-Pf) ppm. HRMS (APCI in toluene, pos. mode) *m*/*z* = 2118.9729 ($[M]^+$, 100 %, calcd for $^{12}C_{128}{}^{1}H_{124}{}^{11}B_2{}^{19}F_{20}{}^{14}N_4$ 2118.9695).

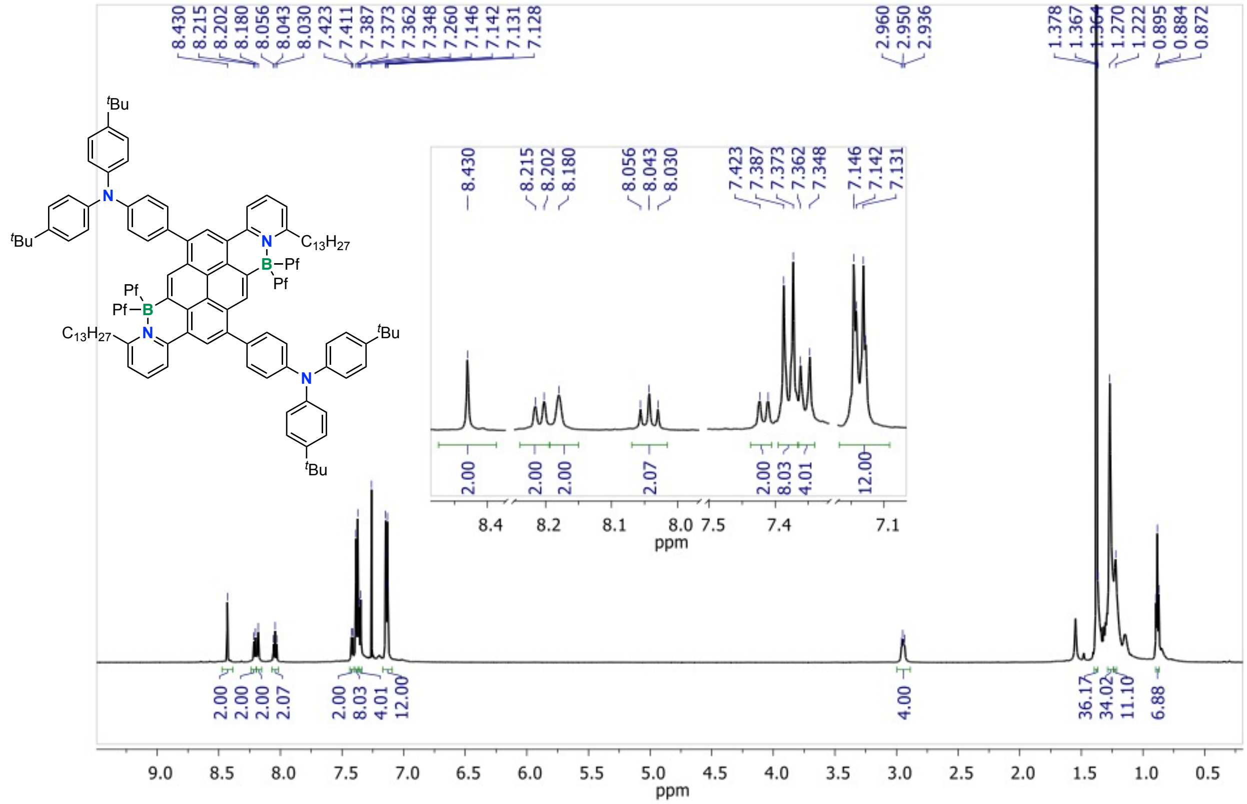

Figure S30. ^{1}H NMR spectrum and expansions of **6-Pf** in $CDCl_3$

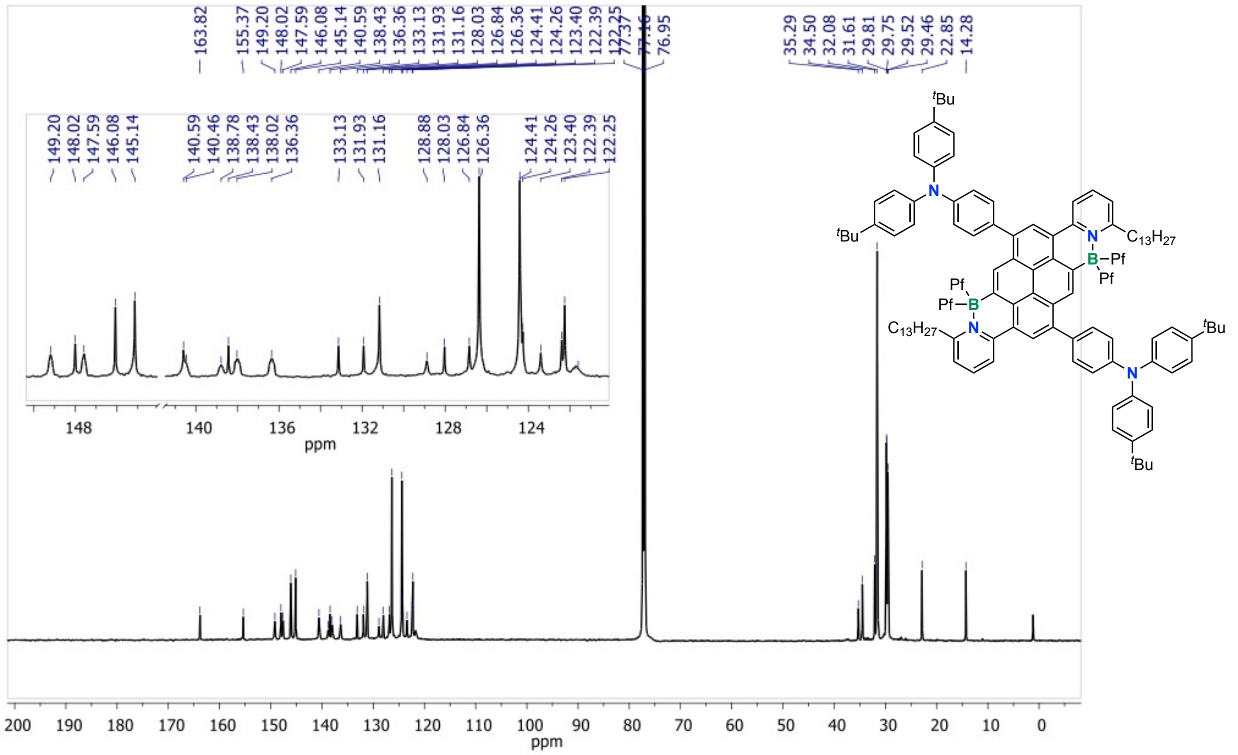

Figure S31. $^{13}C\{^{1}H\}$ NMR spectrum and expansion of **6-Pf** in $CDCl_3$

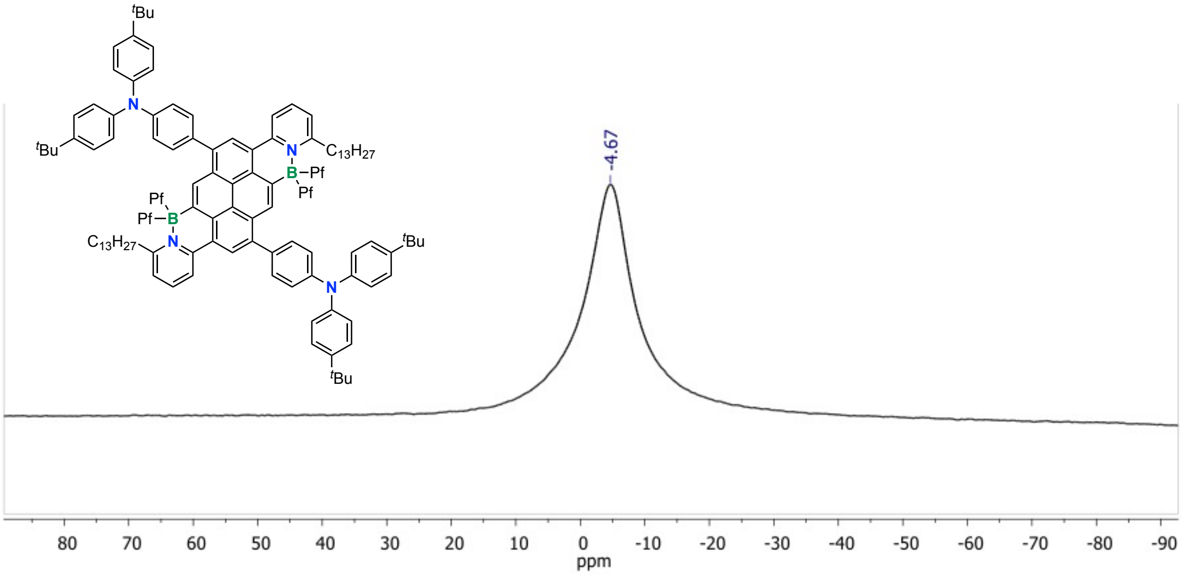

Figure S32. ^{11}B NMR spectrum of **6-Pf** in $CDCl_3$

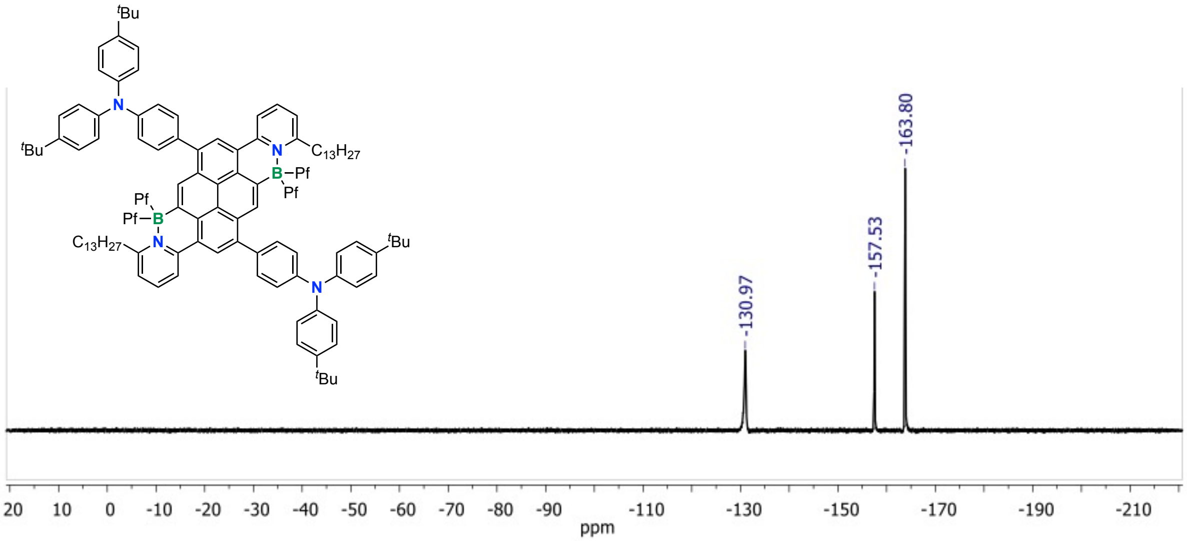

Figure S33. ^{19}F NMR spectrum of **6-Pf** in $CDCl_3$

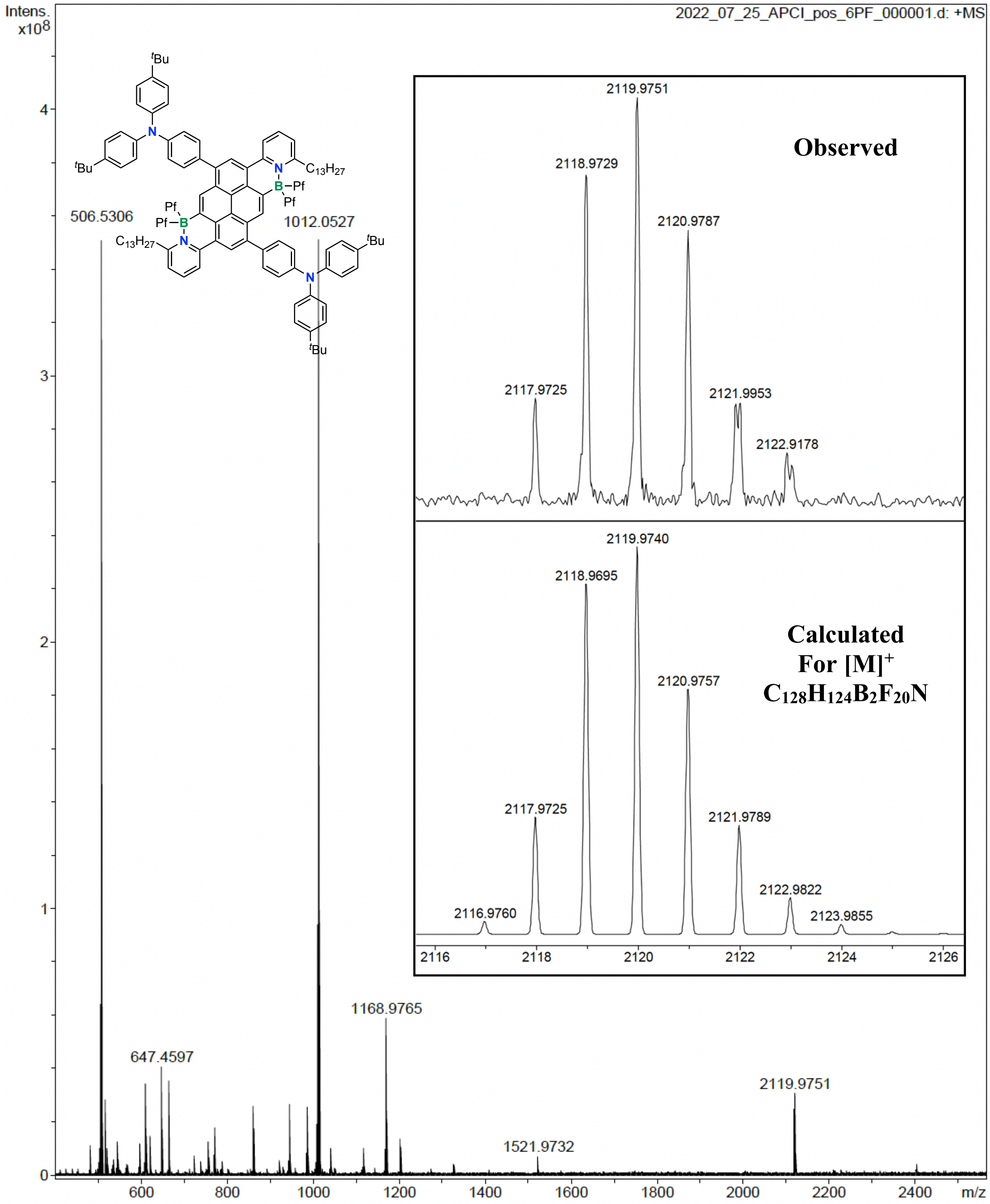

Figure S34. APCI-MS (pos. mode) of **6-Pf** in toluene

3. Crystallographic Details

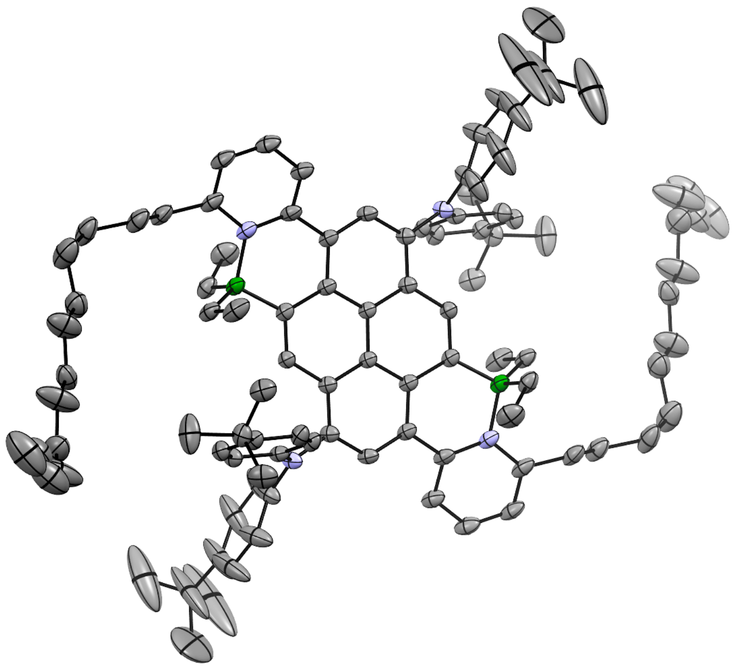

Figure S35a. Top view of the X-ray crystal structure of **5-Et** (B green, N blue, F yellow; thermal ellipsoid at 50% probability; hydrogen atoms are omitted for clarity).

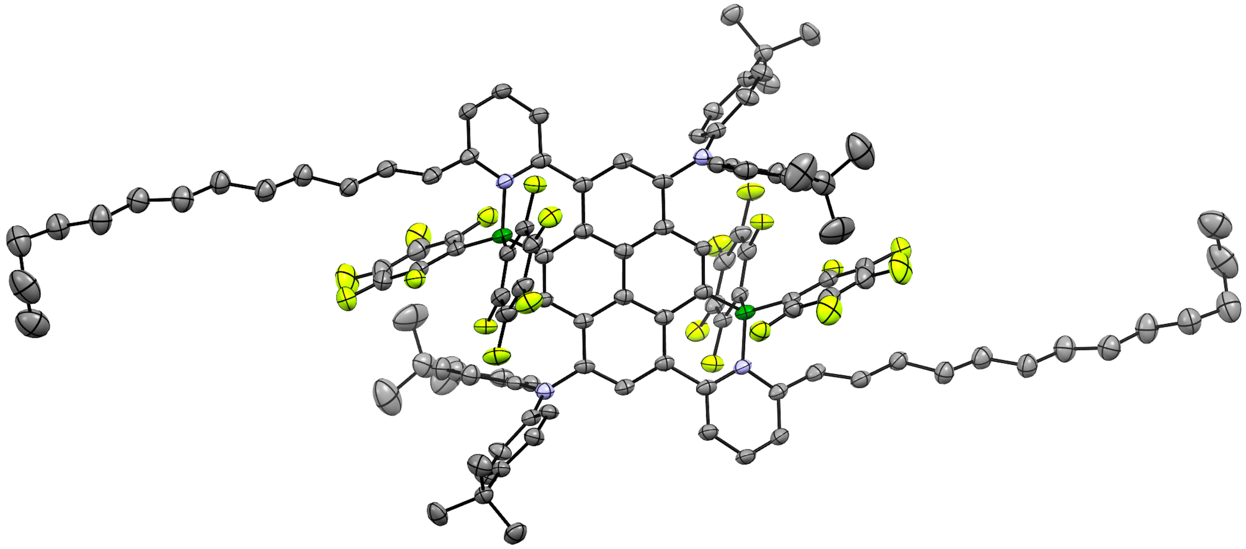

Figure S35b. Top view of the X-ray crystal structures of **5-Pf** (B green, N blue, F yellow; thermal ellipsoid at 50% probability; hydrogen atoms and a disordered THF molecule are omitted for clarity).

4. Variable Temperature ^{1}H and ^{19}F NMR Studies on 5-Pf and 6-Pf

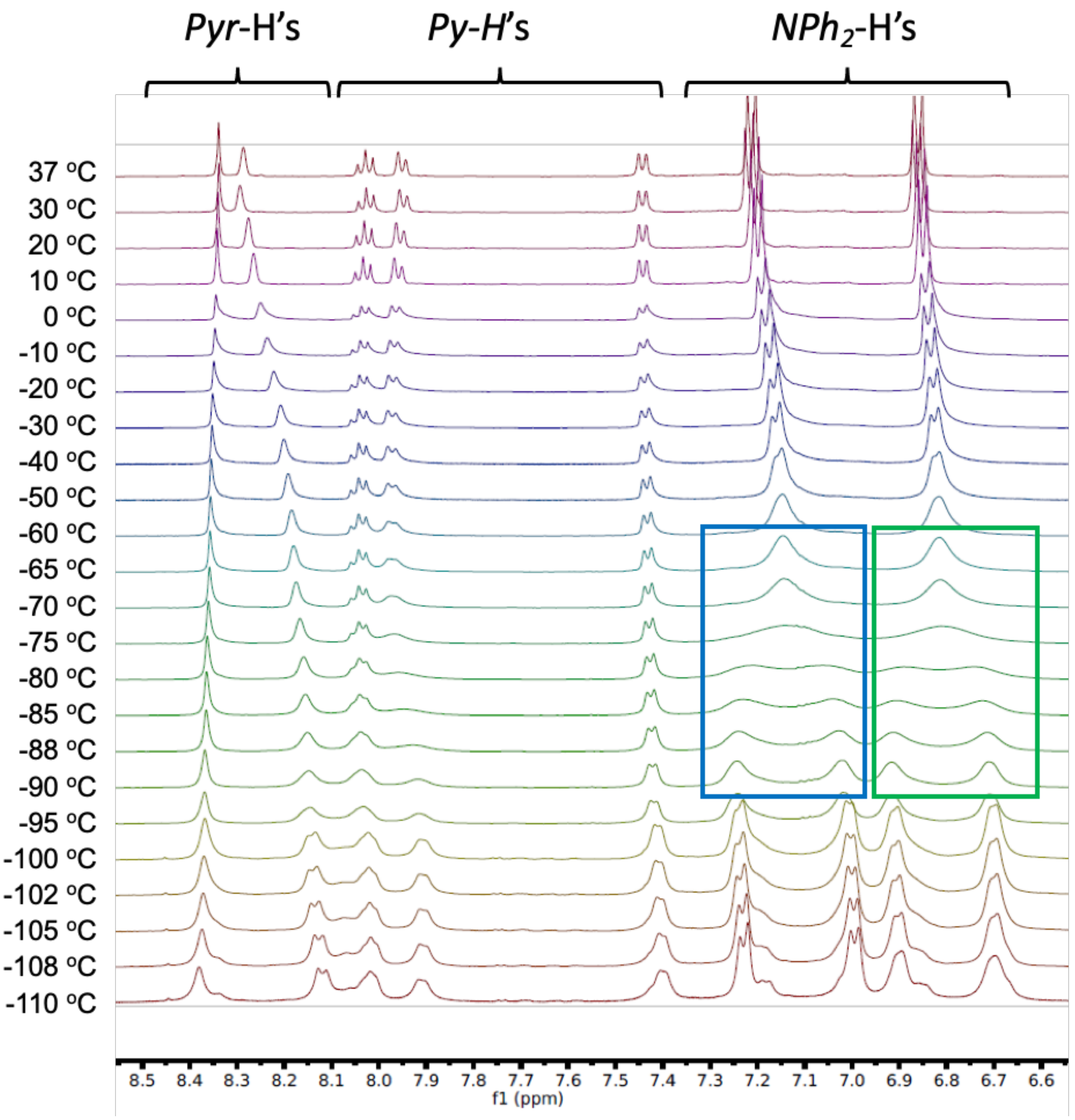

Figure S36. VT ^{1}H NMR spectra of **5-Pf** in CD_2Cl_2; blue and green rectangles highlight the regions used for fitting the rate constant.

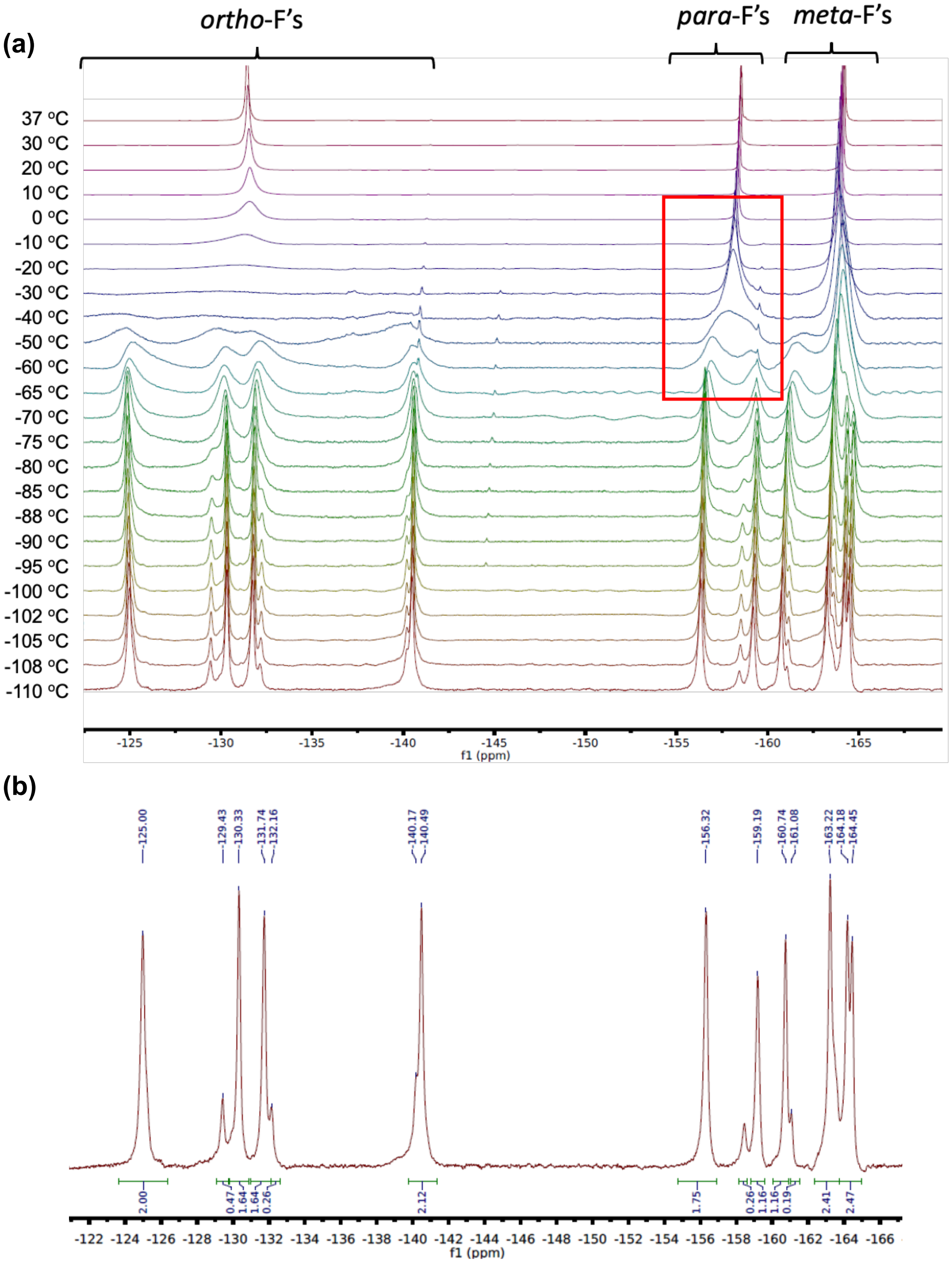

Figure S37. (a) VT ^{19}F NMR spectra of **5-Pf** in CD_2Cl_2; for temperatures between between -30 °C and -65 °C (red box); an exchange process is observed between the *para*-fluorines of the C_6F_5 groups. (b) Integrated ^{19}F NMR spectrum of **5-Pf** acquired at -110 °C, revealing an approximate ratio of 80:20 (4:1) for the major: minor isomer.

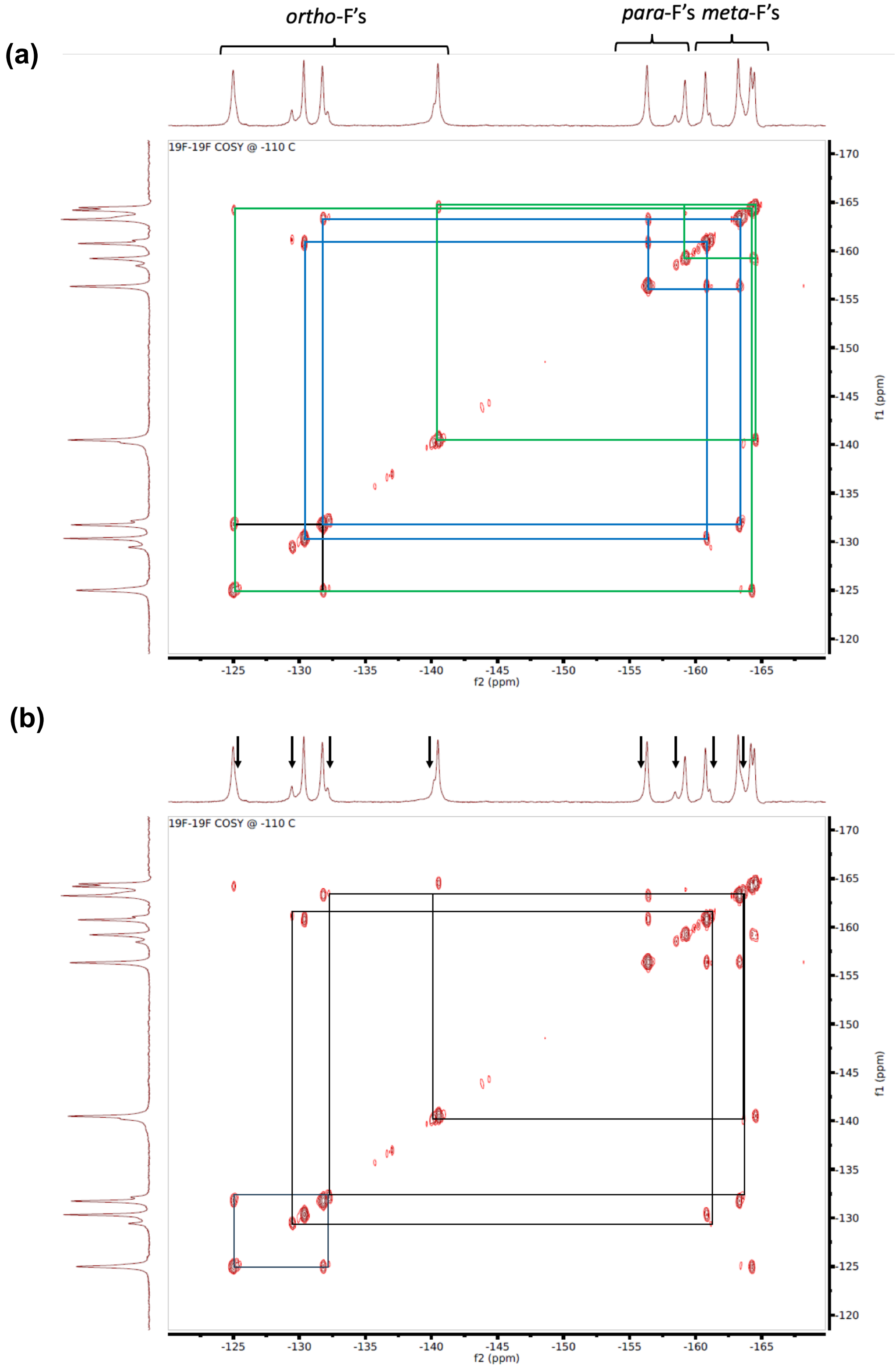

Figure S38. ^{19}F,^{19}F-COSY NMR spectrum of **5-Pf** in CD_2Cl_2 at -100 °C. (a) The spin systems for the major isomer are indicated in blue and green, showing two separate C_6F_5 groups with 5 inequivalent F atoms for each. (b) The signals for the minor isomer are indicated with black arrows.

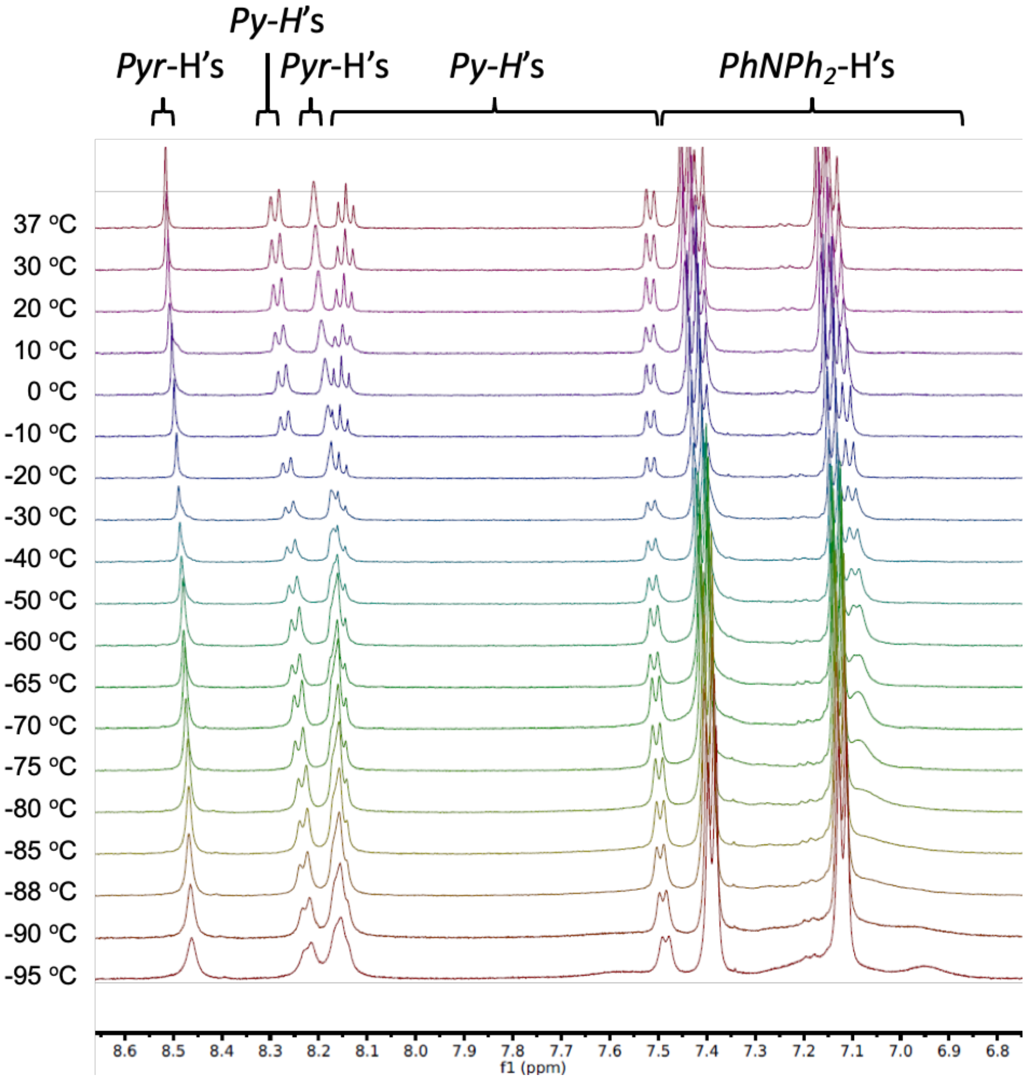

Figure S39. VT ^{1}H NMR spectra of **6-Pf** in CD_2Cl_2.

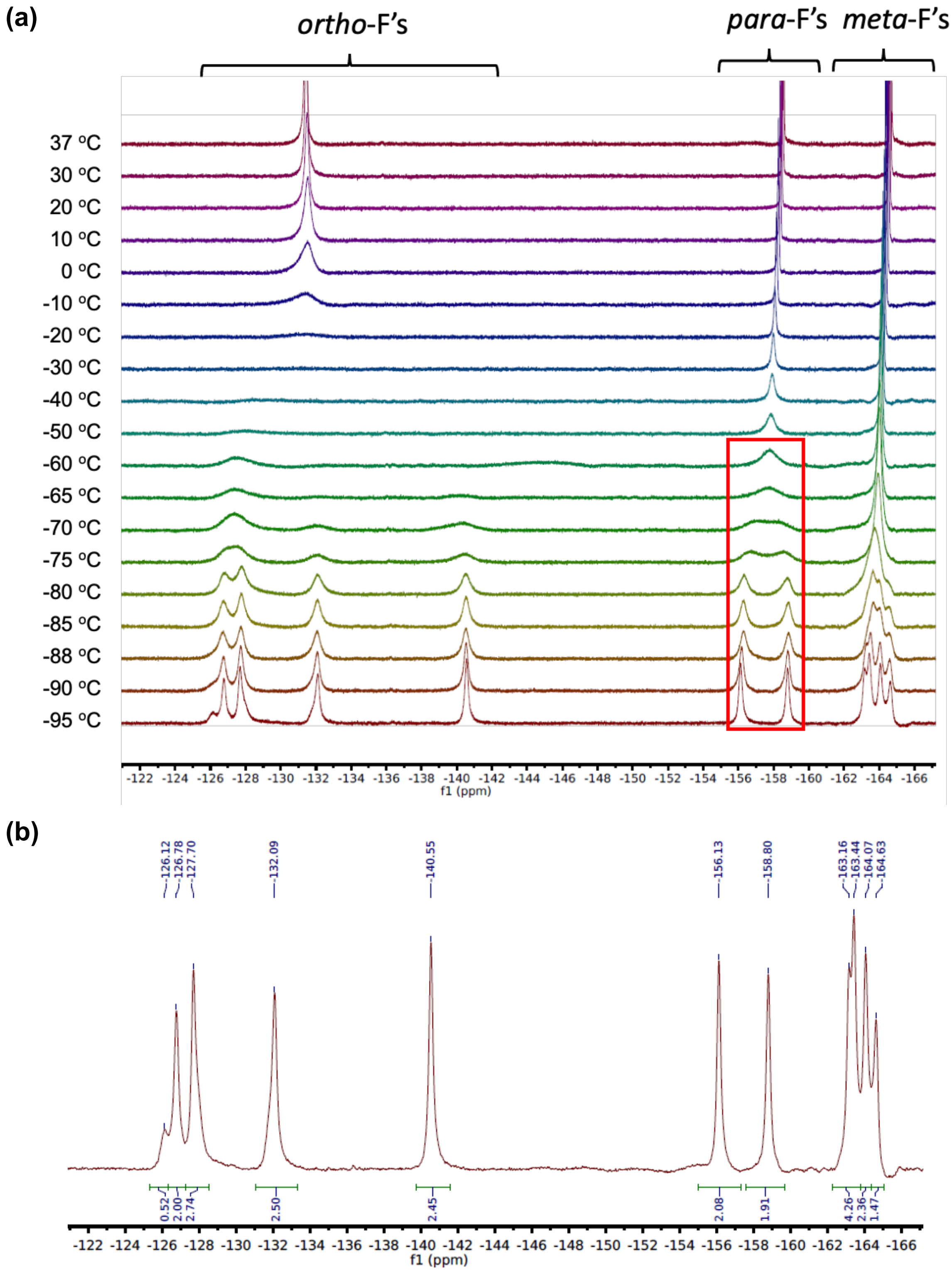

Figure S40. (a) VT ^{19}F NMR spectra of **6-Pf** in CD_2Cl_2; for temperatures between between -60 °C and -95 °C (red box); an exchange process is observed between the *para*-fluorines of the C_6F_5 groups. (b) Integrated ^{19}F NMR spectrum of **6-Pf** acquired at -95 °C, revealing an approximate ratio of 80:20 (4:1) for the major: minor isomer.

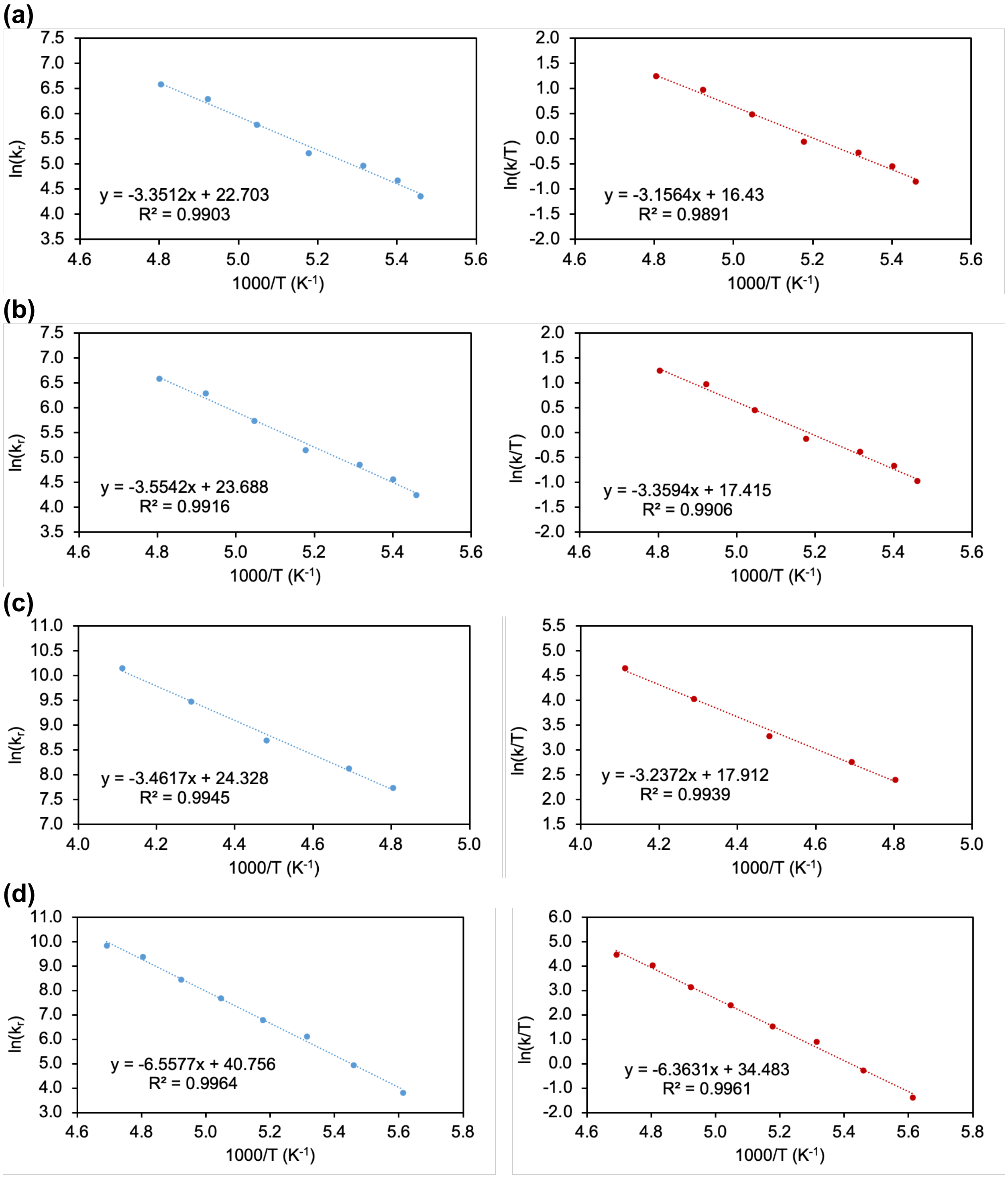

Figure S41. Arrhenius (left) and Eyring (right) plots for analysis of VT NMR spectra in CD_2Cl_2. (a) Merging of ^{1}H NMR signals for NPh_2 groups of **5-Pf** in "blue box" of Figure S36. (b) Merging of ^{1}H NMR signals for NPh_2 groups of **5-Pf** in "green box" of Figure S36. (c) Merging of ^{19}F NMR signals for *para*-fluorines of **5-Pf** in "red box" of Figure S37. (d) Merging of ^{19}F NMR signals for *para*-fluorines of **6-Pf** in "red box" of Figure S40.

Activation Energies and Thermodynamic Parameters Derived from VT NMR Results

Table S1a. Activation energies and thermodynamic parameters from Arrhenius and Eyring analysis of ^{1}H VT NMR spectra of **5-Pf** in CD_2Cl_2 (from Figures S36 and S41a/b).

**5-Pf, ^{1}H NMR – merging *ortho*- and *meta*-NPh_2 signals respectively (Ph group exchange)**			
	#1	#2	Average
E_a (kJ/mol)	29.54	27.86	28.70
$\Delta H^{\neq}$ (kJ/mol)	27.93	26.24	27.09
$\Delta S^{\neq}$ (J/mol K)	-52.75	-60.94	-56.85

At T_c = 195±3 K: $\Delta G^{\neq}_{195}$ = 38.2 kJ/mol; at 298 K: $\Delta G^{\neq}_{298}$ = 44.0 kJ/mol

Table S1b. Activation energies and thermodynamic parameters from Arrhenius and Eyring analysis of ^{19}F VT NMR spectra of **5-Pf** in CD_2Cl_2 (from Figures S37 and S41c).

**5-Pf, ^{19}F NMR – merging *para*-F signals (C_6F_5 group exchange)**	
E_a (kJ/mol)	28.78
$\Delta H^{\neq}$ (kJ/mol)	26.91
$\Delta S^{\neq}$ (J/mol K)	-48.62

At T_c = 223±3 K: $\Delta G^{\neq}_{223}$ = 37.8 kJ/mol; at 298 K: $\Delta G^{\neq}_{298}$ = 41.4 kJ/mol

Table S1c. Activation energies and thermodynamic parameters from Arrhenius and Eyring analysis of ^{19}F VT NMR spectra of **5-Pf** in CD_2Cl_2 (from Figures S40 and S41d).

**6-Pf, ^{19}F NMR – merging *para*-F signals (C_6F_5 group exchange)**	
E_a (kJ/mol)	54.52
$\Delta H^{\neq}$ (kJ/mol)	52.90
$\Delta S^{\neq}$ (J/mol K)	89.15

At T_c = 205±3 K: $\Delta G^{\neq}_{205}$ = 34.6 kJ/mol; at 298 K: $\Delta G^{\neq}_{298}$ = 26.3 kJ/mol

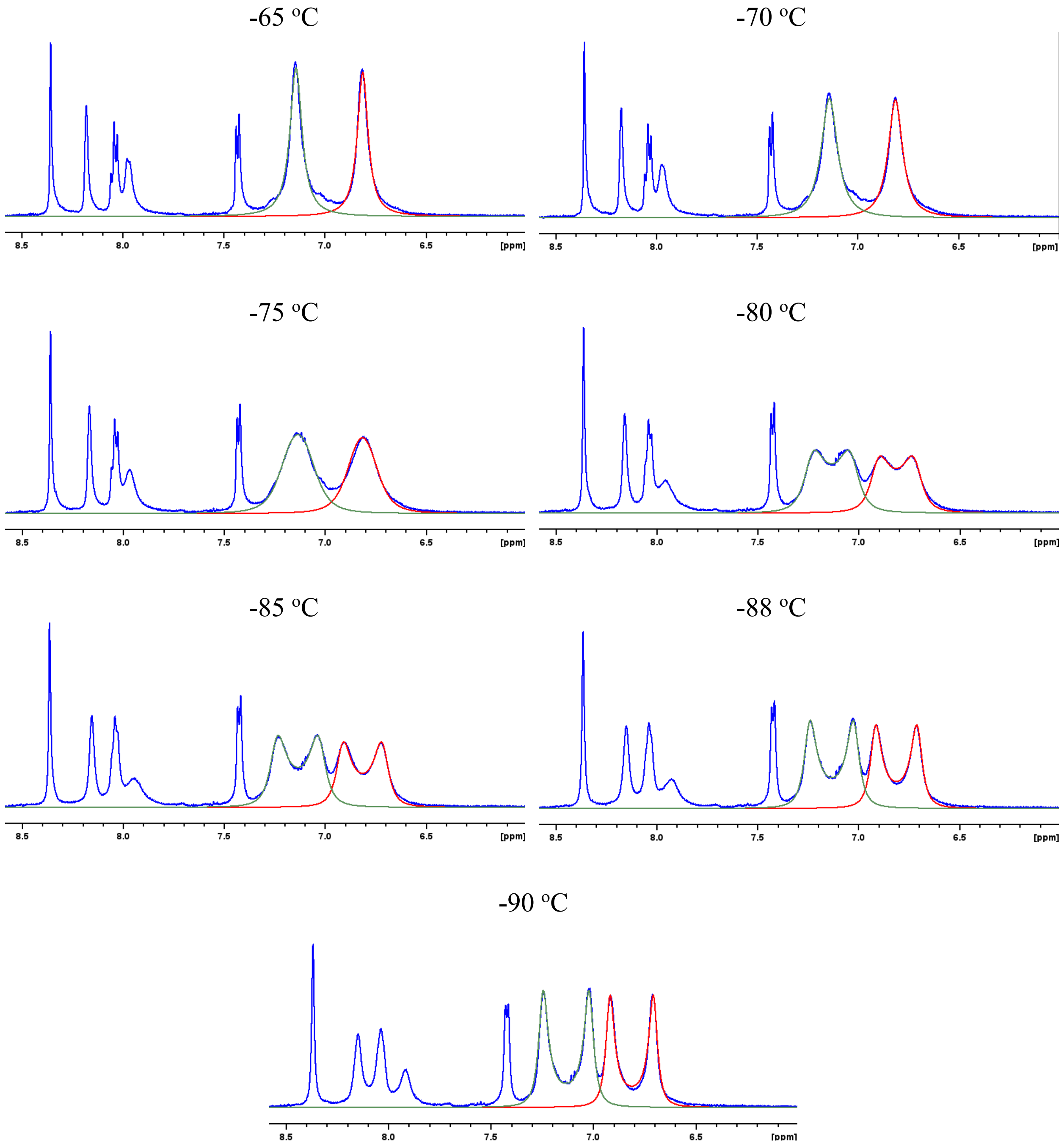

Figure S42a. DNMR fitting of ^{1}H VT NMR spectra of **5-Pf** in CD_2Cl_2 (see Figure S36).

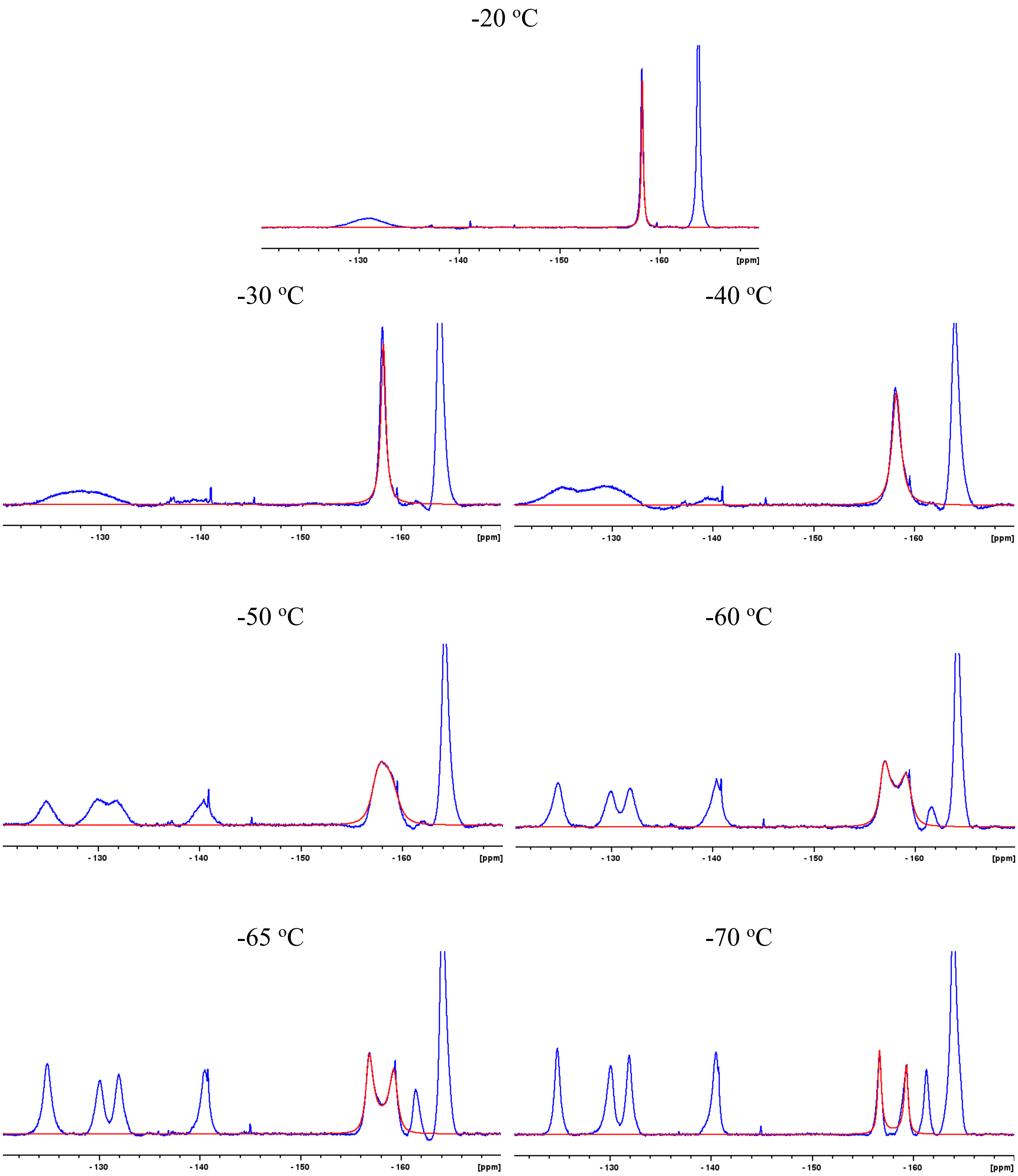

Figure S42b. DNMR fitting of ^{19}F VT NMR spectra of **5-Pf** in CD_2Cl_2 (see Figure S37).

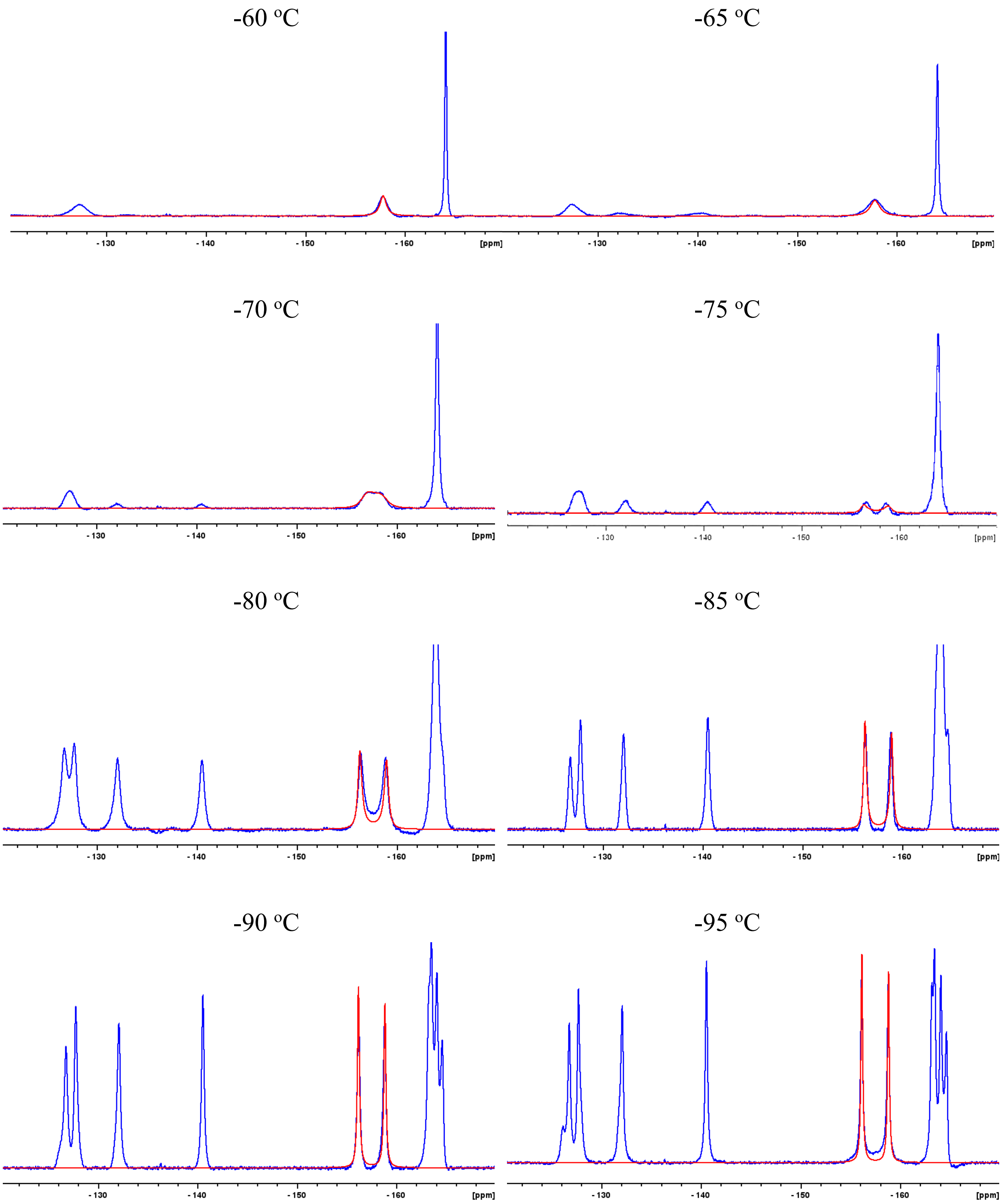

Figure S42c. DNMR fitting of ^{19}F VT NMR spectra of **6-Pf** in CD_2Cl_2 (see Figure S40).

5. Electrochemical Studies

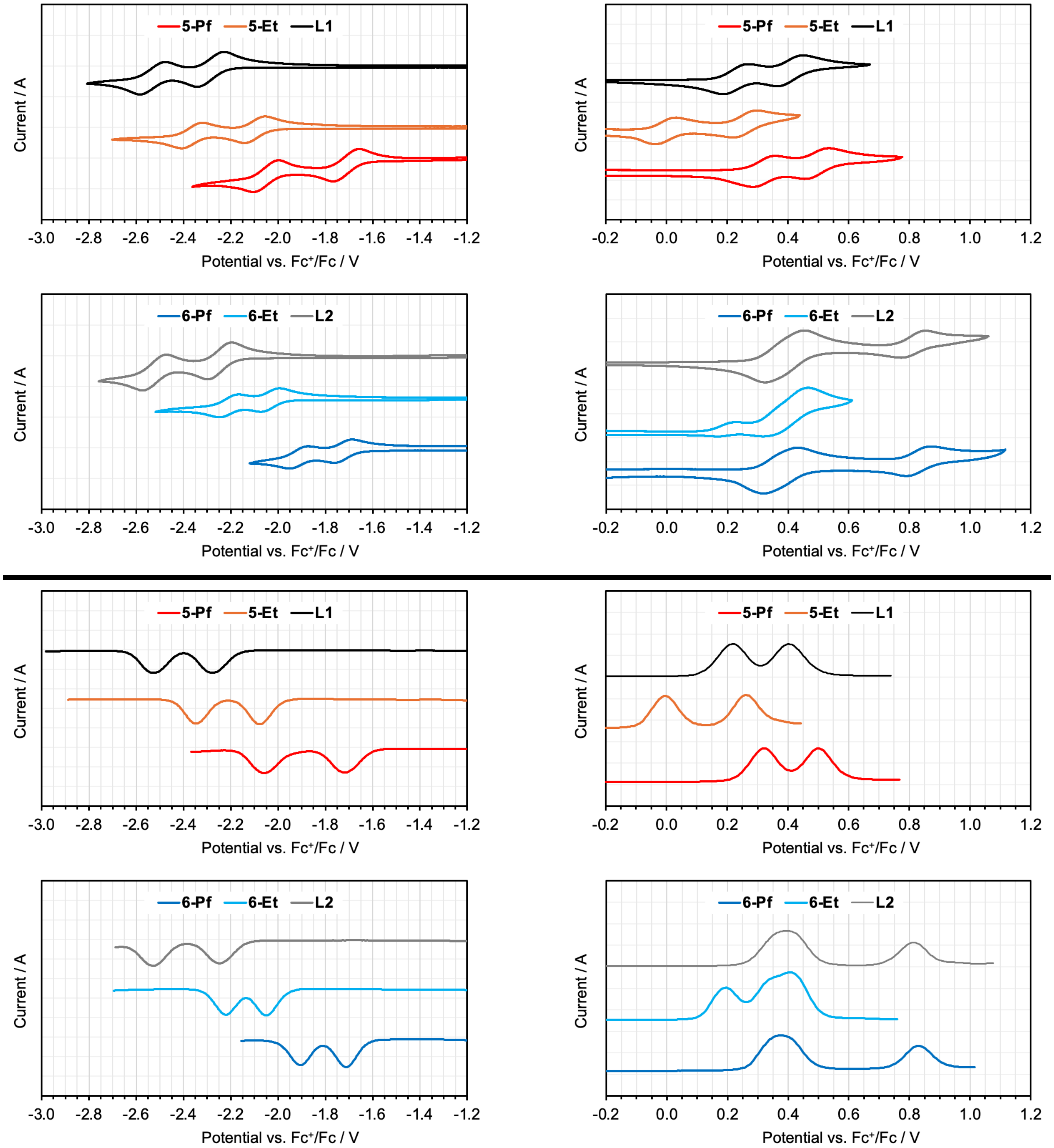

Figure S43. Cyclic voltammetry (CV, top) and square wave voltammetry (SWV, bottom) data for the reduction (left) and oxidation (right) of ca. 1 mM B-N Lewis pair functionalized donor-acceptor pyrenes and their precursors in THF (reduction) or DCM (oxidation) containing 0.1M $Bu_4N[PF_6]$, recorded at v = 100 mV s^{-1}. Data for reduction and oxidation scans reported *vs* $Fc^{+/0}$. For oxidation scans decamethylferrocene (Fc*) was used as the internal reference and the data converted to $E(Fc^{0/+})$ = 0 V using the equation $E(Fc^{*0/+}) = E(Fc^{0/+}) - 0.54$ V.

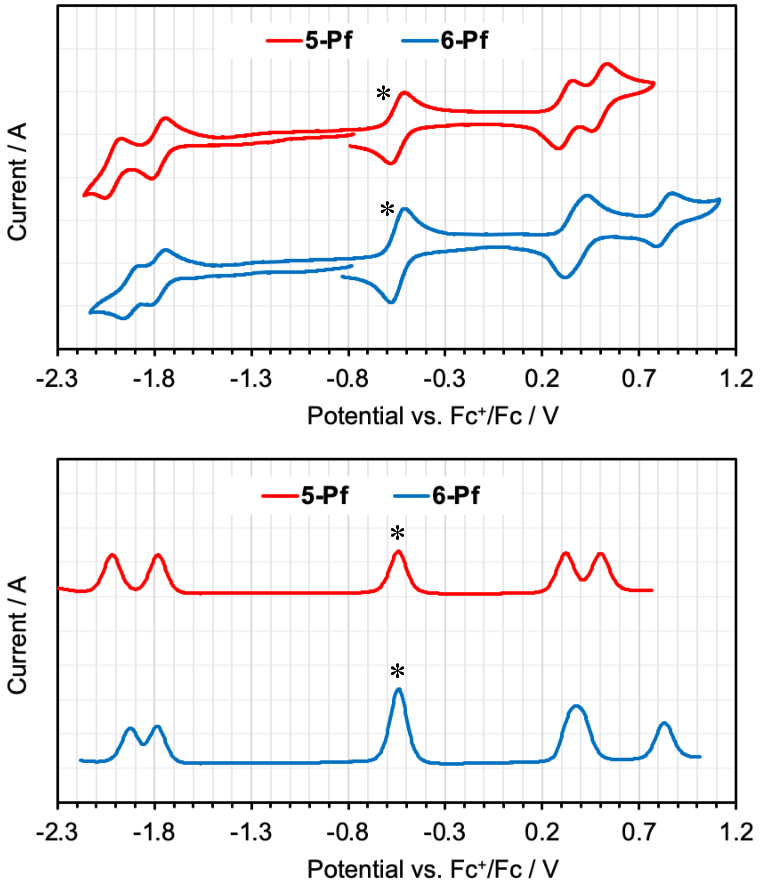

Figure S44. Cyclic voltammetry (CV, top) and square wave voltammetry (SWV, bottom) data for ca. 1 mM solutions of **5-Pf** and **6-Pf** in DCM containing 0.1M $Bu_4N[PF_6]$, recorded at v = 100 mV s^{-1}. Data reported *vs* $Fc^{+/0}$, decamethylferrocene (Fc*, indicated with asterisk) was used as the internal reference and the data converted to $E(Fc^{0/+}) = 0$ V using the equation $E(Fc^{*0/+}) = E(Fc^{0/+}) - 0.54$ V.

*Note that compounds **5-Et** and **6-Et** did not show good reversibility of both oxidation and reduction scans in either THF or DCM.*

Table S2. Electrochemical data of B-N Lewis pair functionalized donor-acceptor pyrenes.

Compd	E_{ox}[a] / V (CV)	ΔE_{ox1-2}[a] / V (CV)	E_{red}[a] / V (CV)	ΔE_{red1-2}[a] / V (CV)	E_{ox}[b] / V (SWV)	E_{red}[b] / V (SWV)	HOMO[c] / eV (CV)	LUMO[c] / eV (CV)
**2**	0.85	N/A	-2.31<br>-2.61	0.30	0.79	n.d.	-5.65	-2.49
**L1**	0.23<br>0.41	0.18	-2.29<br>-2.53	0.24	0.22<br>0.40	-2.28<br>-2.53	-5.03	-2.51
**5-Et**	0.04<br>0.26	0.22	-2.10<br>-2.37	0.27	0.00<br>0.26	-2.08<br>-2.37	-4.84	-2.70
**5-Pf**	0.32<br>0.50	0.18	-1.71<br>-2.05	0.34	0.32<br>0.50	-1.72<br>-2.06	-5.12	-3.09
**L2**	0.39[d]<br>0.81	0.42[d]	-2.25<br>-2.52	0.27	0.39[d]<br>0.81	-2.25<br>-2.53	-5.19	2.55
**6-Et**	0.20[e]<br>0.39	0.19[e]	-2.03<br>-2.21	0.18	0.19[e]<br>0.40	-2.05<br>-2.22	-5.00	-2.77
**6-Pf**	0.38[d]<br>0.83	0.45[d]	-1.72<br>-1.91	0.19	0.38[d]<br>0.83	-1.71<br>-1.91	-5.18	-3.08

[a] Derived from cyclic voltammetry data, E_{red} or $E_{ox} = 0.5$ ($E_{pc} + E_{pa}$); [b] derived from square-wave voltammetry data; [c] $E_{LUMO} = -(4.8+E_{red})$, $E_{HOMO} = -(4.8+E_{ox})$; [d] first and second oxidation process overlap (2e process), *ΔE given* for separation to third oxidation event; [e] first 1e-oxidation process is irreversible and the following oxidations overlap (2e process).

Table S3. Comparison of HOMO/LUMO levels and band gaps derived from electrochemical data, UV-vis data, and DFT calculation results.

	E_{HOMO}[a] / eV (CV)	E_{LUMO}[a] / eV (CV)	**ΔE_{gap} / eV (CV)**	E_{HOMO}[b] / eV (DFT)	E_{LUMO}[b] / eV (DFT)	**ΔE_{gap} / eV (DFT)**	**ΔE_{gap}[c] / eV (UV-Vis)**
**2**	-5.65	-2.49	**3.16**	n.d.	n.d.	**n.d.**	**3.12**
**L1**	-5.03	-2.51	**2.52**	-4.53	-1.69	**2.84**	**2.53**
**5-Et**	-4.84	-2.70	**2.14**	-4.40	-2.11	**2.29**	**2.06**
**5-Pf**	-5.12	-3.09	**2.03**	-4.75	-2.65	**2.10**	**2.00**
**L2**	-5.19	-2.55	**2.64**	-4.60	-1.64	**2.96**	**2.68**
**6-Et**	-5.00	-2.77	**2.23**	-4.47	-2.09	**2.38**	**2.21**
**6-Pf**	-5.18	-3.08	**2.10**	-4.72	-2.61	**2.11**	**2.12**

[a] Derived from CV data, $E_{LUMO} = -(4.8+E_{red})$, $E_{HOMO} = -(4.8+E_{ox})$; [b] from DFT calculations for compounds with Me in place of longer alkyl and ^{t}Bu groups at RB3LYP/6-31G(d) level of theory; [c] from absorption onset.

6. Photophysical Studies

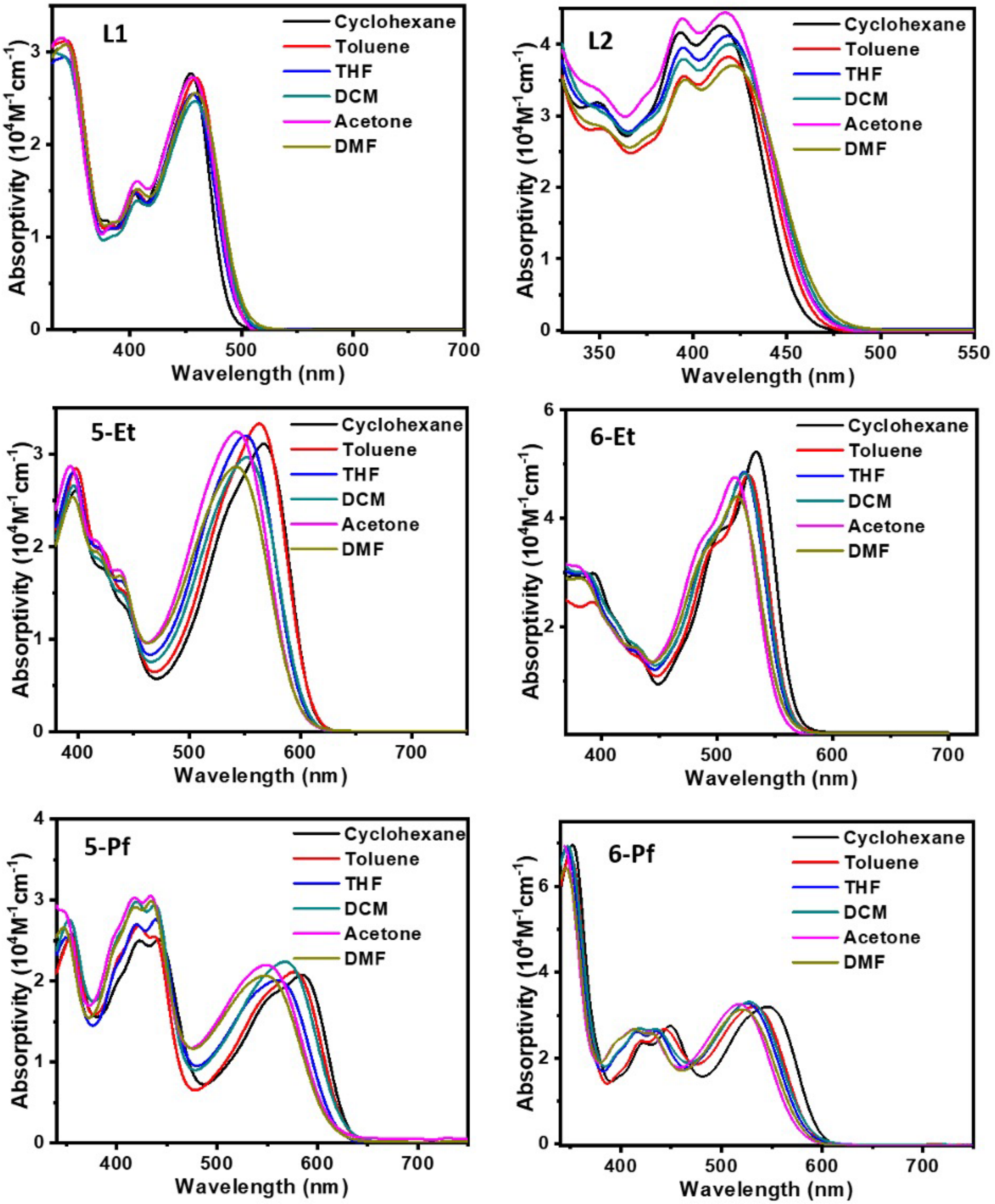

Figure S45. UV-Vis absorption spectra of **L1**, **5-Et**, **5-Pf**, **L2**, **6-Et** and **6-Pf** in solvents of different polarity.

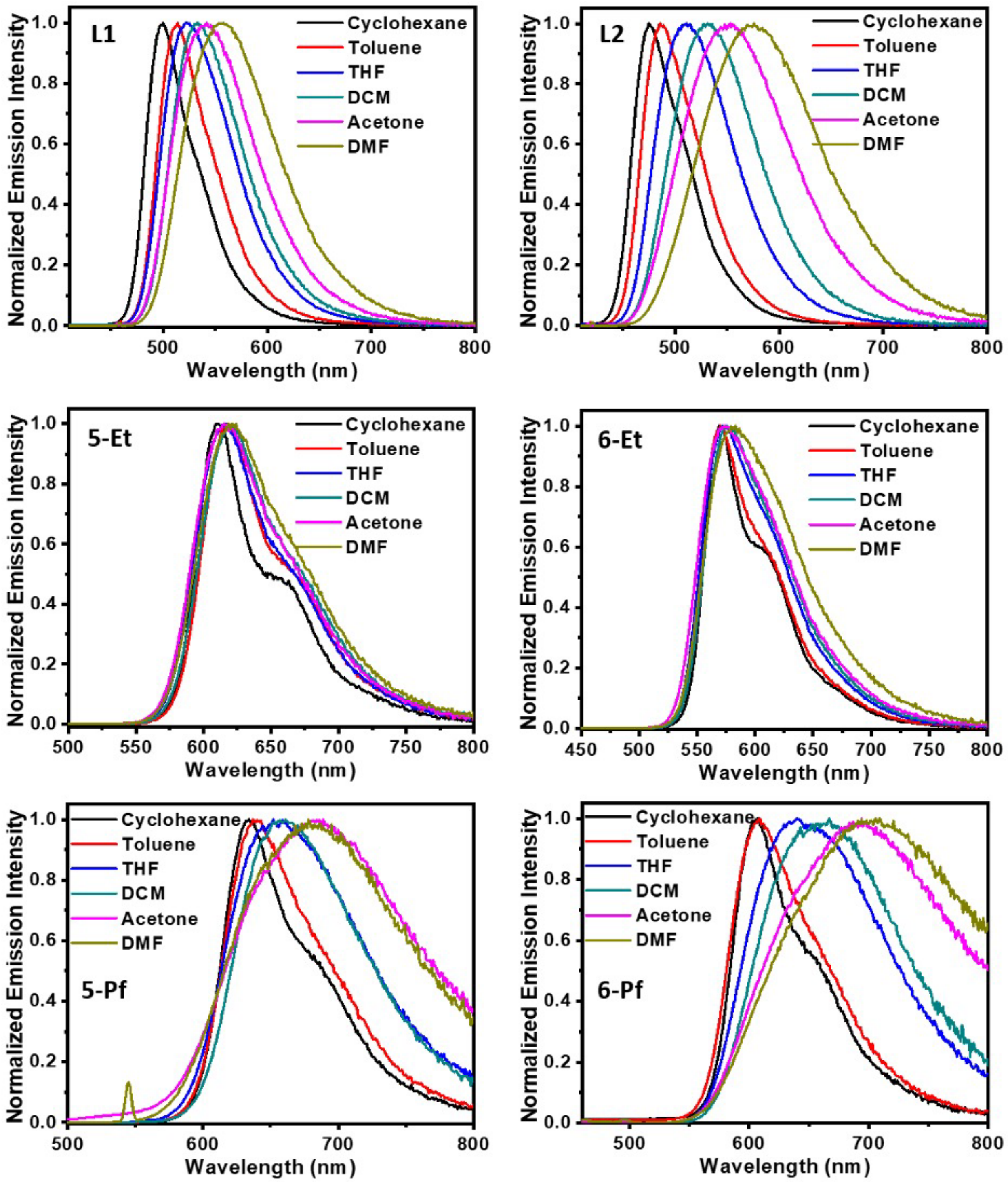

Figure S46. Fluorescence spectra of **L1**, **5-Et**, **5-Pf**, **L2**, **6-Et** and **6-Pf** in solvents of different polarity.

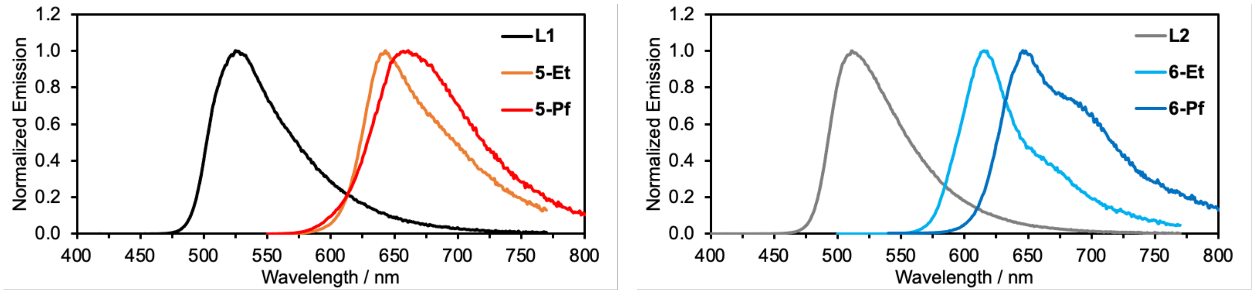

Figure S47a. Solid state fluorescence spectra of (left) **L1**, **5-Et**, **5-Pf** and (right) **L2**, **6-Et**, **6-Pf**; excited at 390 nm for **L1**, **L2**, **5-Et**, **6-Et**; 500 nm for **5-Pf**; at 526 nm for **6-Pf**.

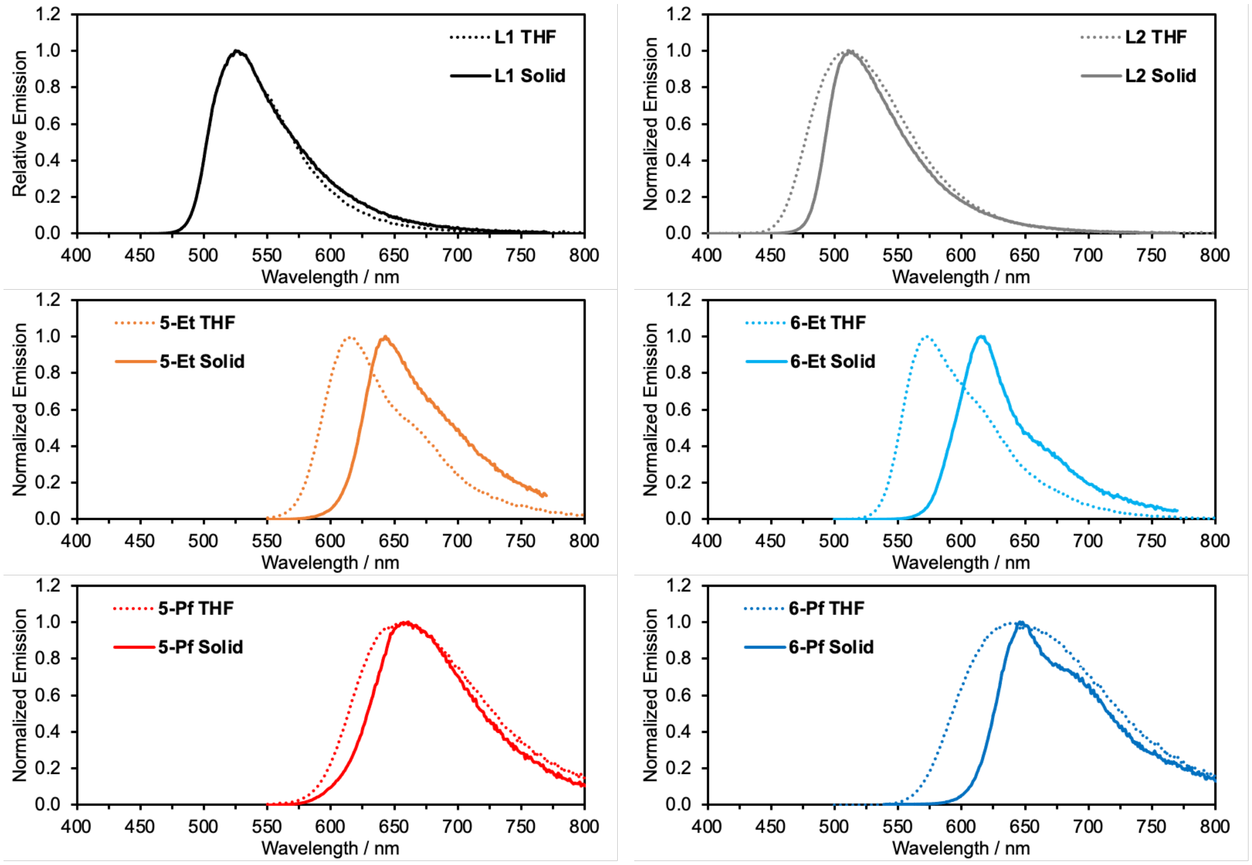

Figure S47b. Comparison of solution (THF) and solid state fluorescence spectra of (left) **L1**, **5-Et**, **5-Pf** and (right) **L2**, **6-Et**, **6-Pf**.

Table S4. Photophysical data in solvents of different polarity and in the solid state.

Compd	Solvent	λ_{abs} /nm	ε_{abs} /10^4 M^{-1} cm^{-1}	λ_{em} /nm
**2**	Cyclohexane	364	3.93	406
	Toluene	366	3.83	412
	THF	364	3.87	408
	DCM	363	3.53	409
	Acetone	362	3.96	408
	DMF	364	3.67	411
	Solid			*454*
**L1**	Cyclohexane	455, 402, 377	2.77, 1.50, 1.18	500
	Toluene	459, 406, 343	2.72, 1.49, 3.12	514
	THF	456, 405, 340	2.54, 1.48, 2.95	522
	DCM	458, 407, 333	2.47, 1.40, 2.99	533
	Acetone	456, 406, 338	2.73, 1.60, 3.15	542
	DMF	459, 407, 342	2.56, 1.52, 3.08	558
	Solid			*525*
**5-Et**	Cyclohexane	567, 445, 425, 398	3.11, 1.30, 1.73, 2.61	611
	Toluene	563, 443,423, 398	3.33, 1.50, 1.95, 2.85	618
	THF	550, 440, 420, 396	3.20, 1.63, 1.98, 2.80	617
	DCM	552, 440, 420, 396	2.97, 1.50, 1.86, 2.66	621
	Acetone	542, 437, 416, 393	3.24, 1.75, 2.10, 2.87	615
	DMF	542, 438, 418, 395	2.87, 1.68, 1.94, 2.53	621
	Solid			*643*
**5-Pf**	Cyclohexane	584, 442, 423, 356	2.08, 2.52, 2.50, 2.59	634
	Toluene	577, 437, 422, 355	2.11, 2.55, 2.68, 3.00	637
	THF	560, 439, 420, 349	2.01, 2.76, 2.70, 3.00	652
	DCM	567, 438, 420, 353	2.24, 2.94, 2.98, 2.98	661
	Acetone	549, 434, 418	2.20, 3.05, 3.03	689
	DMF	548, 434, 419, 347	2.07, 2.99, 2.91, 2.66	678
	Solid			*661*
**L2**	Cyclohexane	419, 395, 351	3.83, 3.55, 2.82	475
	Toluene	419, 395, 349	3.83, 3.55, 2.82	486
	THF	418, 395, 348	4.26, 4.09, 3.30	511
	DCM	420, 395, 348	4.00, 3.79, 3.11	531
	Acetone	417, 394, 347	4.44, 4.36, 3.40	553

	DMF	421, 396, 349	3.70, 3.51, 2.87	576
	Solid			*511*
**6-Et**	Cyclohexane	534, 503, 393, 377	5.23, 3.78, 2.99,2.95	569, 609
	Toluene	527, 497, 431, 412, 393	4.80, 3.49, 1.45, 1.93, 2.46	570
	THF	524, 494, 431, 409, 385	4.85, 3.62, 1.54, 2.04, 2.98	574
	DCM	524, 495, 431, 412, 386	4.84, 3.64, 1.65, 2.12, 3.00	576
	Acetone	516, 488, 428, 379	4.76, 3.63, 1.62, 3.12	574
	DMF	518, 489, 429, 382	4.40, 3.37, 1.62, 2.90	583
	Solid			*616*
**6-Pf**	Cyclohexane	546, 449, 422, 352	3.19, 2.75, 2.35, 6.97	607
	Toluene	532, 444, 421, 349	3.20, 2.68, 2.40, 6.72	608
	THF	525, 435, 417, 347	3.27, 2.62, 2.61, 6.91	640
	DCM	527, 434, 418, 347	3.31, 2.67, 2.70, 6.95	667
	Acetone	517, 428, 413	3.25, 2.61, 2.68	694
	DMF	521, 430, 414, 345	3.13, 2.58, 2.67, 6.43	709
	Solid			*646*

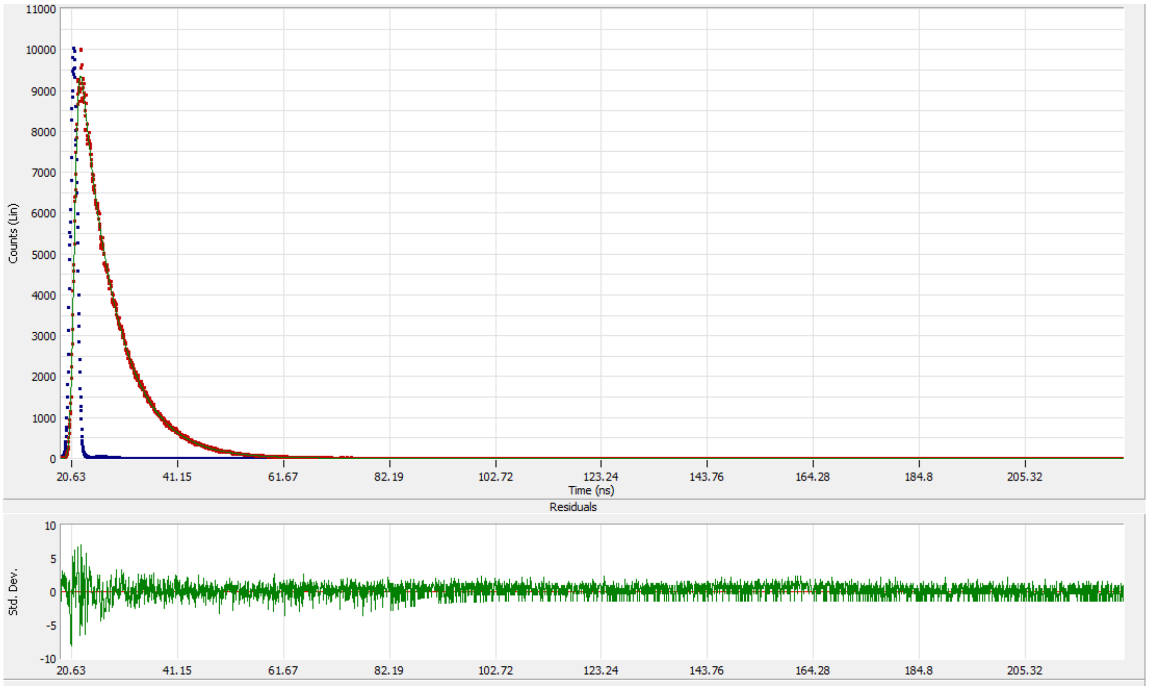

Figure S48a. Single-exponential fit of fluorescence decay of **L1 in THF** excited with a 450 nm nanoLED. τ_1 = 6.8 ns, 100% (χ^2 = 1.25)

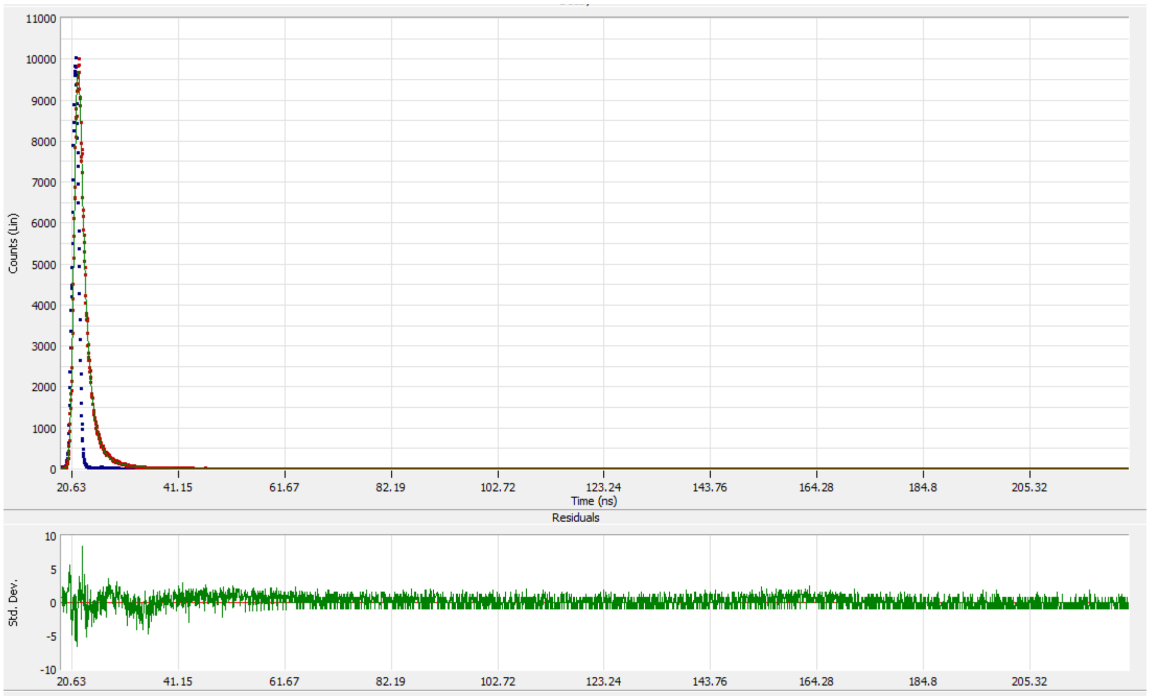

Figure S48b. Double-exponential fit of fluorescence decay of **L1 in the solid state** excited with a 390 nm nanoLED. τ_1 = 1.2 ns, 87%; τ_2 = 4.4 ns, 13% (χ^2 = 1.01)

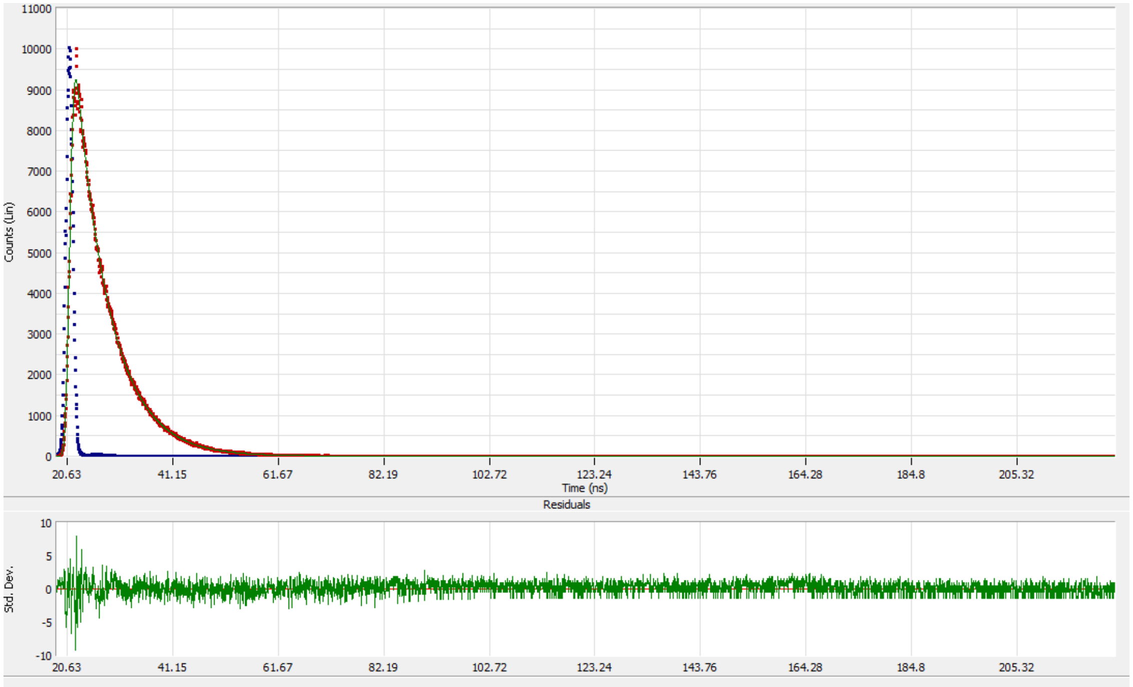

Figure S49a. Single-exponential fit of fluorescence decay of **5-Et in THF** excited with a 450 nm nanoLED. τ_1 = 6.6 ns, 100% (χ^2 = 1.18)

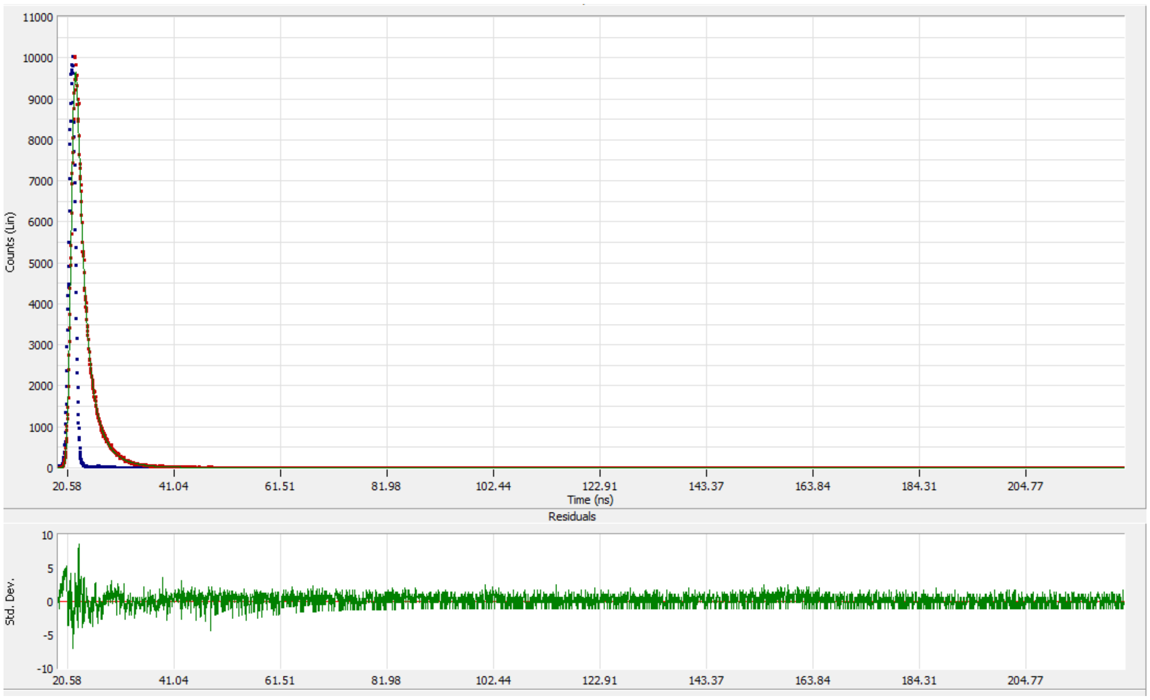

Figure S49b. Triple-exponential fit of fluorescence decay of **5-Et in the solid state** excited with a 390 nm nanoLED. τ_1 = 1.4 ns, 57%; τ_2 = 3.1 ns, 41% τ_3 = 9.3 ns, 2% (χ^2 = 1.08)

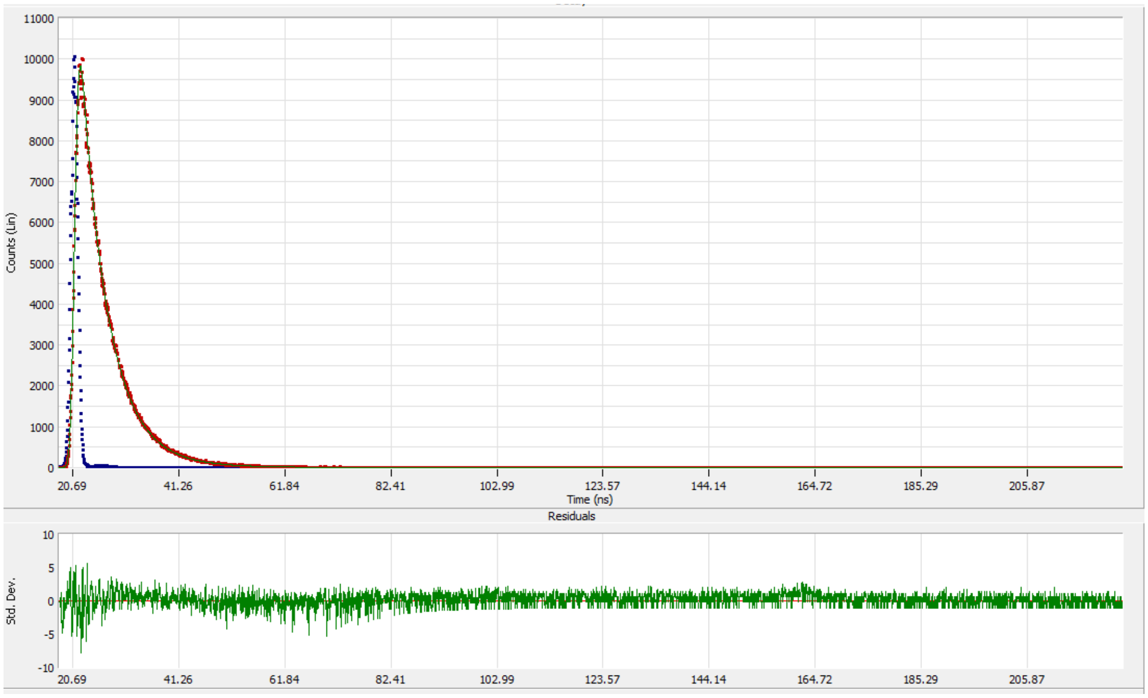

Figure S50a. Double-exponential fit of fluorescence decay of **5-Pf in THF** excited with a 450 nm nanoLED. τ_1 = 4.8 ns, 81%; τ_2 = 8.5 ns, 19% (χ^2 = 1.28)

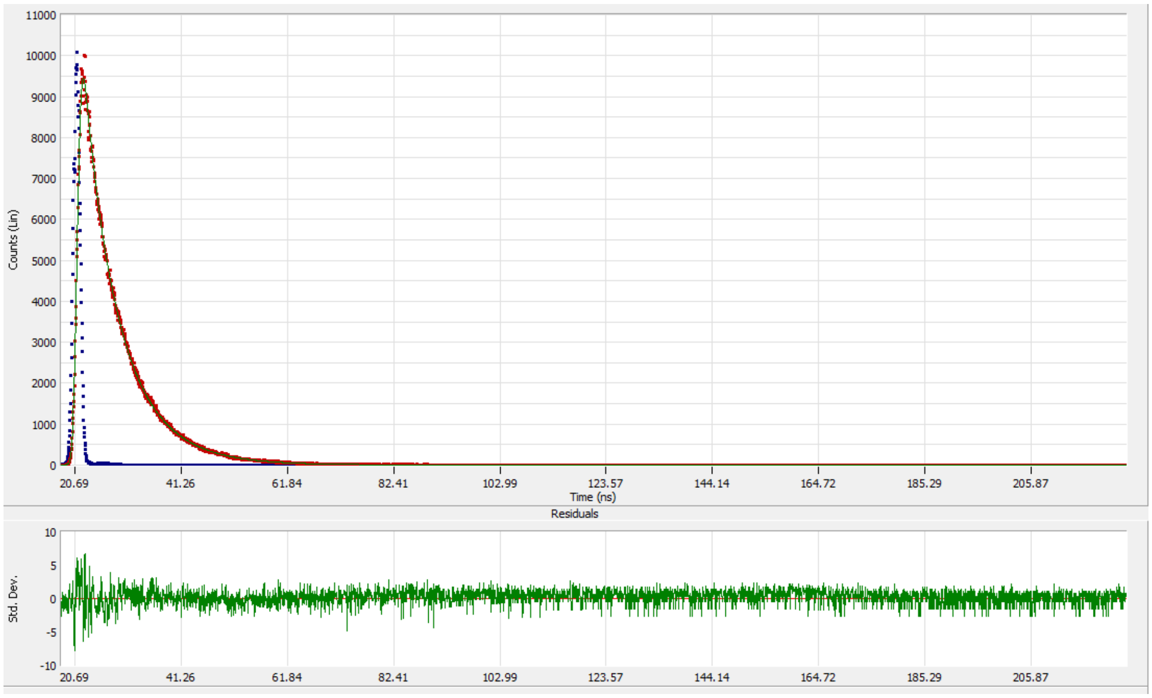

Figure S50b. Double-exponential fit of fluorescence decay of **5-Pf in the solid state** excited with a 450 nm nanoLED. τ_1 = 4.7 ns, 29%; τ_2 = 8.3 ns, 71% (χ^2 = 1.41)

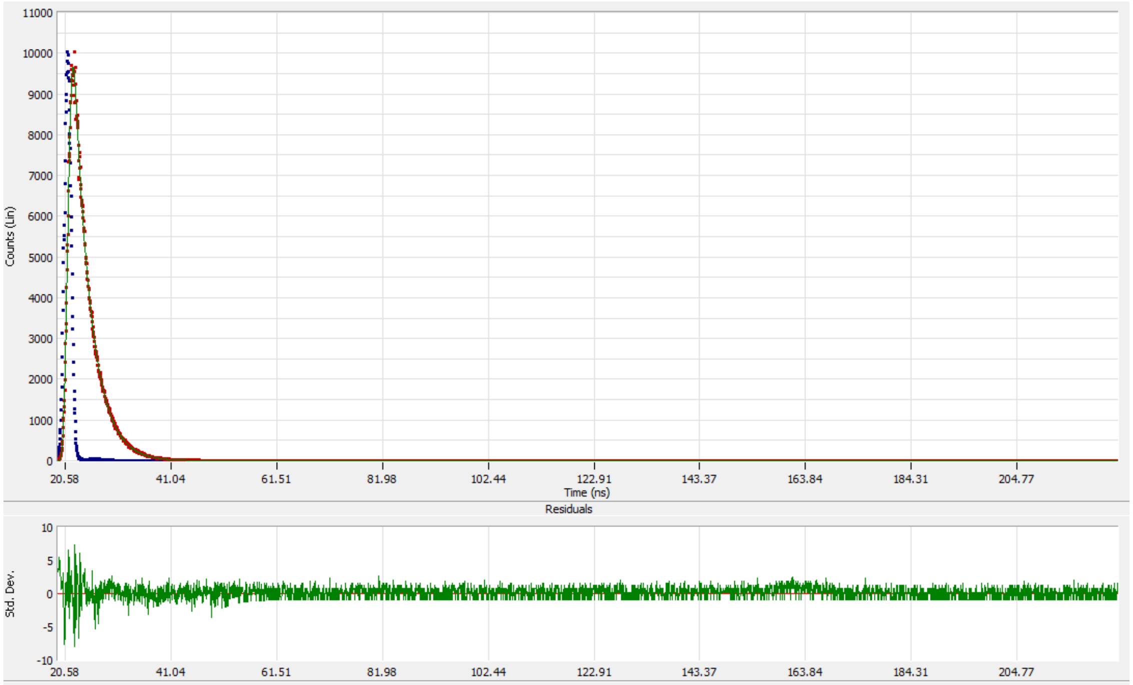

Figure S51a. Single-exponential fit of fluorescence decay of **L2 in THF** excited with a 450 nm nanoLED. τ_1 = 3.2 ns, 100% (χ^2 = 1.00)

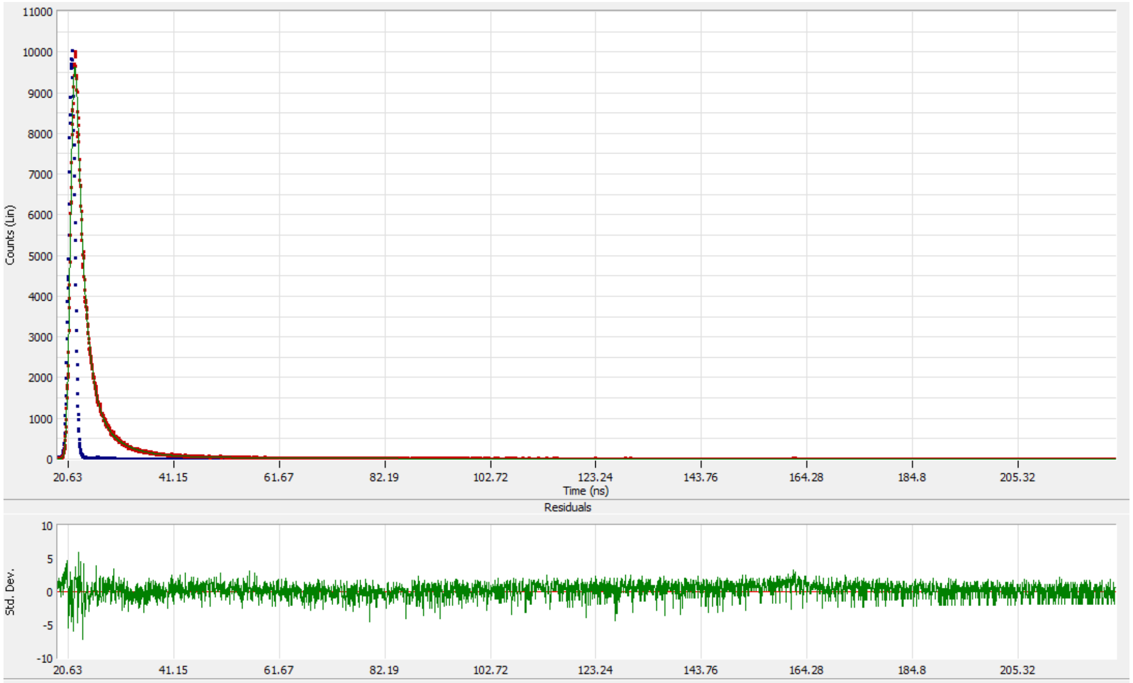

Figure S51b. Triple-exponential fit of fluorescence decay of **L2 in the solid state** excited with a 390 nm nanoLED. τ_1 = 1.6 ns, 61%; τ_2 = 4.7 ns, 31%; τ_3 = 29.3 ns, 8% (χ^2 = 1.38)

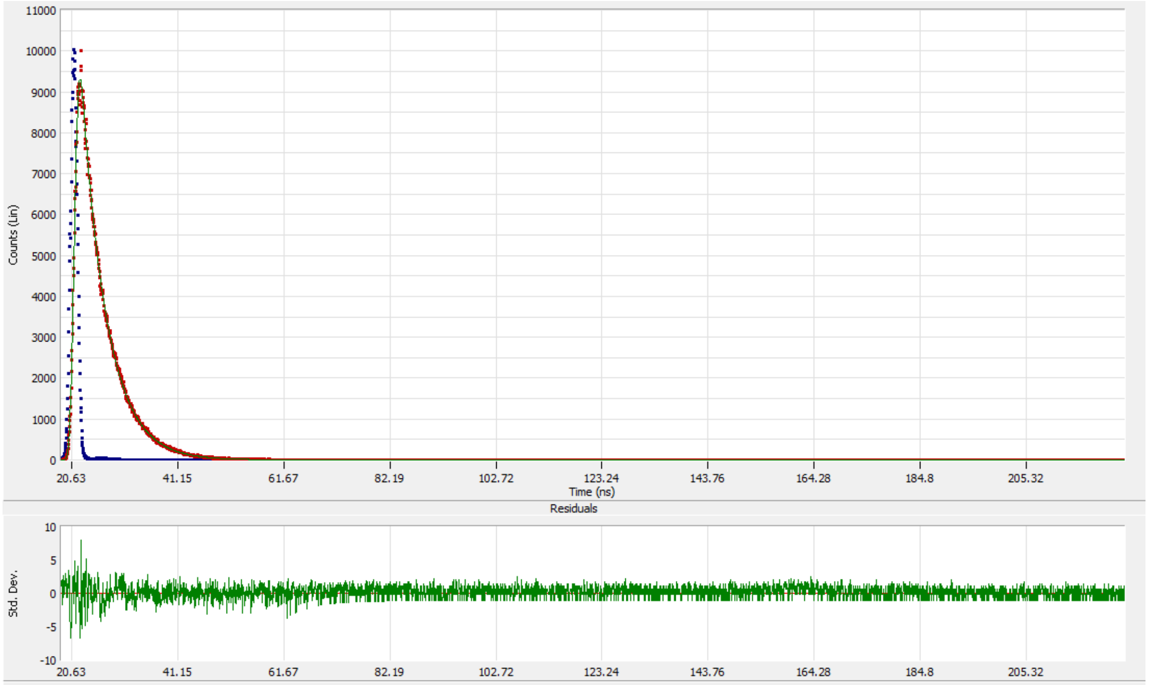

Figure S52a. Single-exponential fit of fluorescence decay of **6-Et in THF** excited with a 450 nm nanoLED. τ_1 = 4.8 ns, 100% (χ^2 = 1.09)

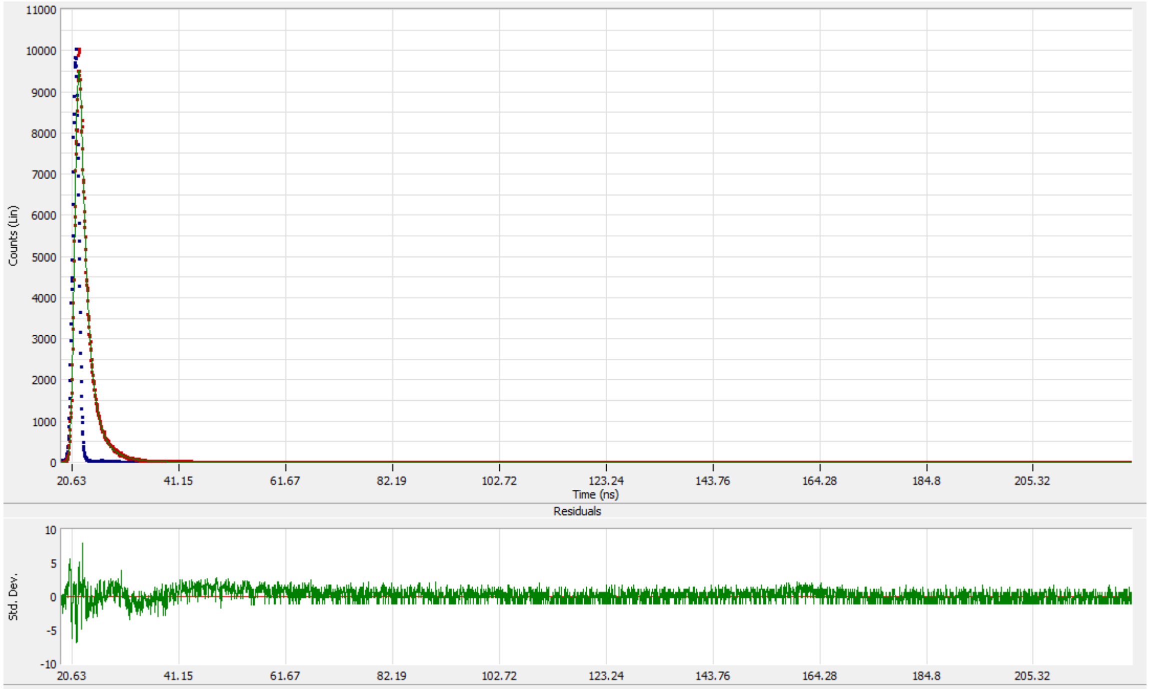

Figure S52b. Double-exponential fit of fluorescence decay of **6-Et in the solid state** excited with a 390 nm nanoLED. τ_1 = 1.3 ns, 76%; τ_2 = 3.4 ns, 24% (χ^2 = 1.08)

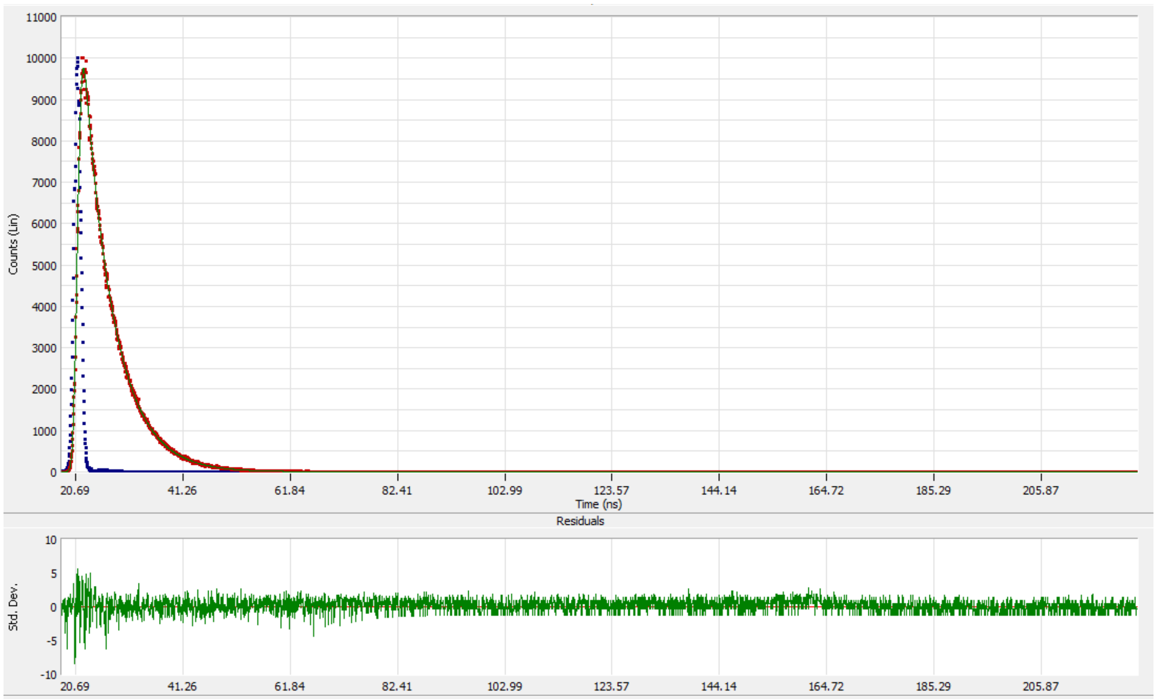

Figure S53a. Single-exponential fit of fluorescence decay of **6-Pf in THF** excited with a 450 nm nanoLED. τ_1 = 5.6 ns, 100% (χ^2 = 1.13)

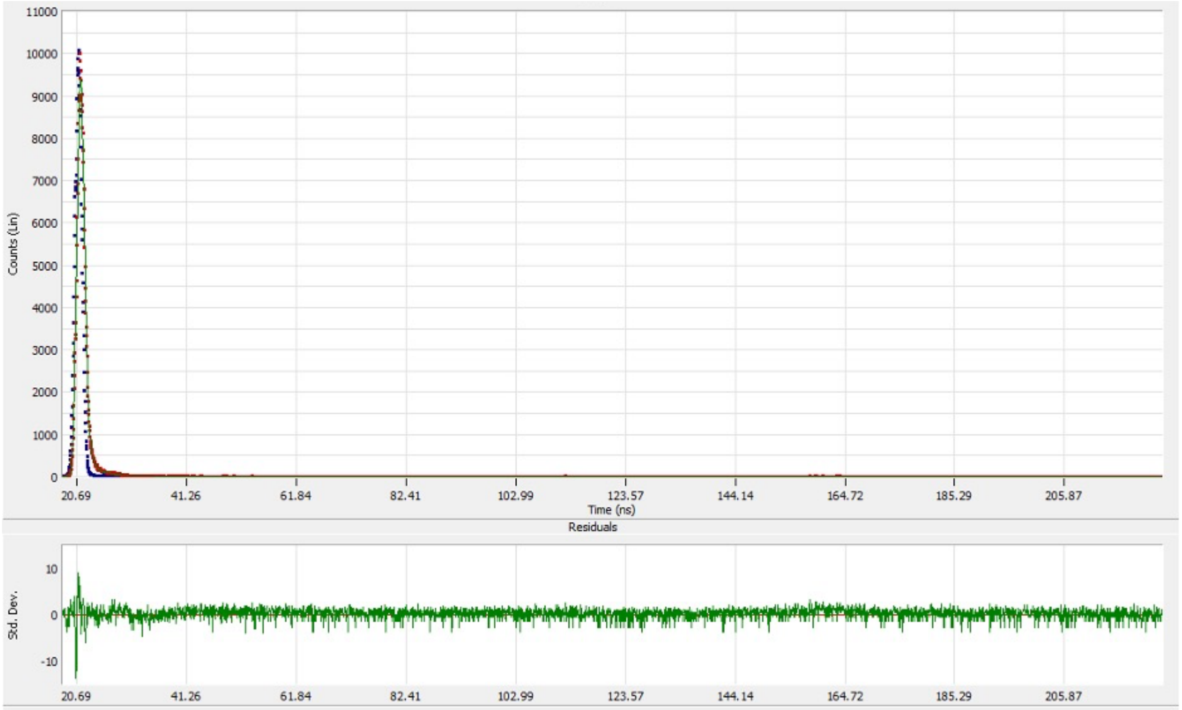

Figure S53b. Triple-exponential fit of fluorescence decay of **6-Pf in the solid state** excited with a 450 nm nanoLED. τ_1 = 1.0 ns, 15%; τ_2 = 4.9 ns, 4%; τ_3 = 0.2 ns, 81% (χ^2 = 1.57)

6. Computational Studies

Density functional theory (DFT) calculations were performed with the Gaussian16 suite of programs.[S5] The input files were generated from X-ray data when available (**5-Et**, **5-Pf**) or in Chem3D and then pre-optimized in Spartan '08 V 1.2.0. Geometries were optimized in Gaussian16 using the hybrid density functional RB3LYP with a 6-31G(d) basis set. Frequency calculations were performed to confirm the presence of local minima (no imaginary frequencies). Vertical excitations were calculated by time-dependent DFT (TD-DFT) methods at the rcam-B3LYP/6-31G(d) level.

5-Me'

5-Pf'

6-Me'

6-Pf'

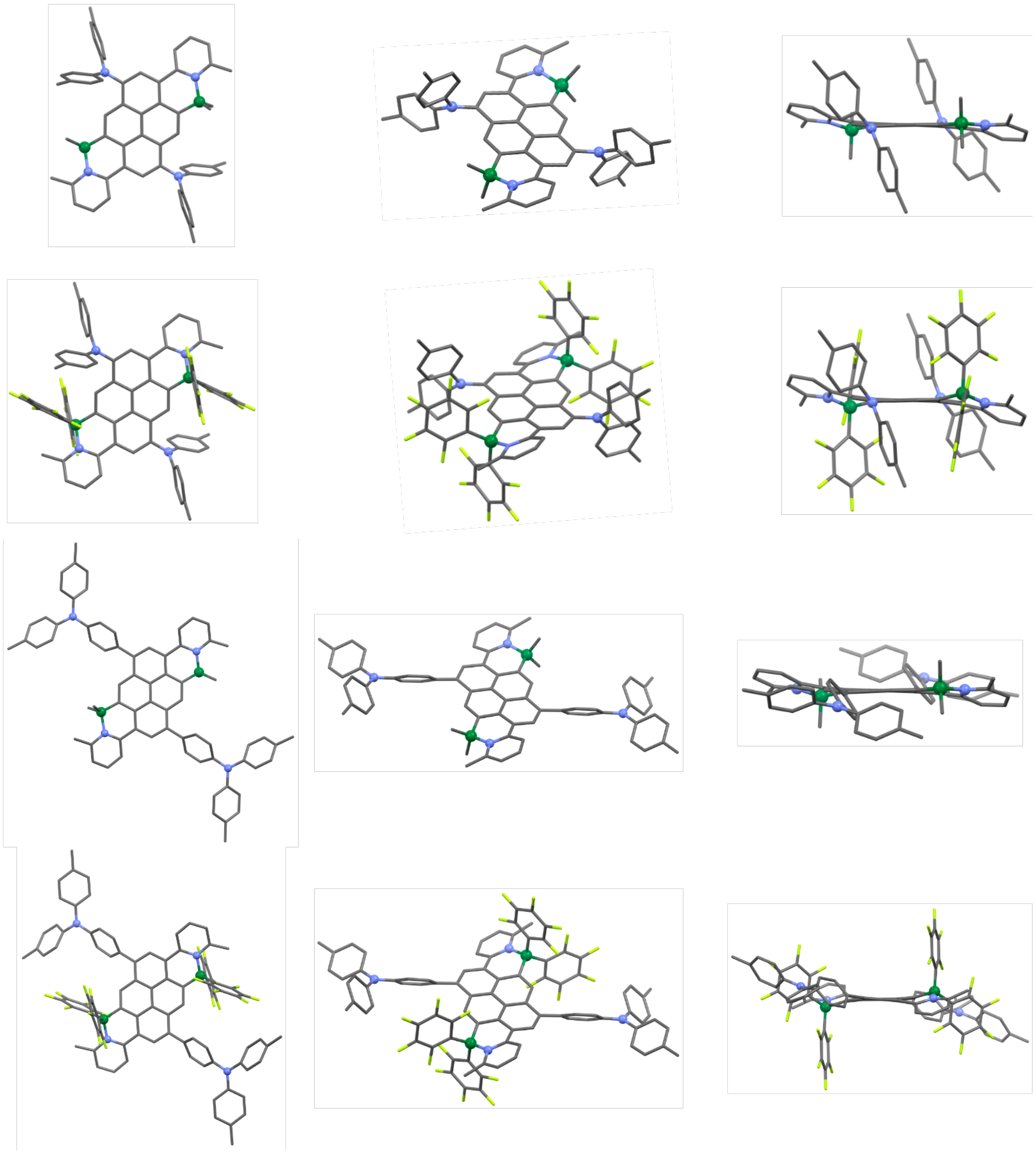

Figure S54. Different views of the DFT-optimized structures of D-A BN pyrene model systems with all longer alkyl and ethyl groups replaced by methyl groups (RB3LYP/6-31G(d)). C grey, B green, N blue, F yellow.

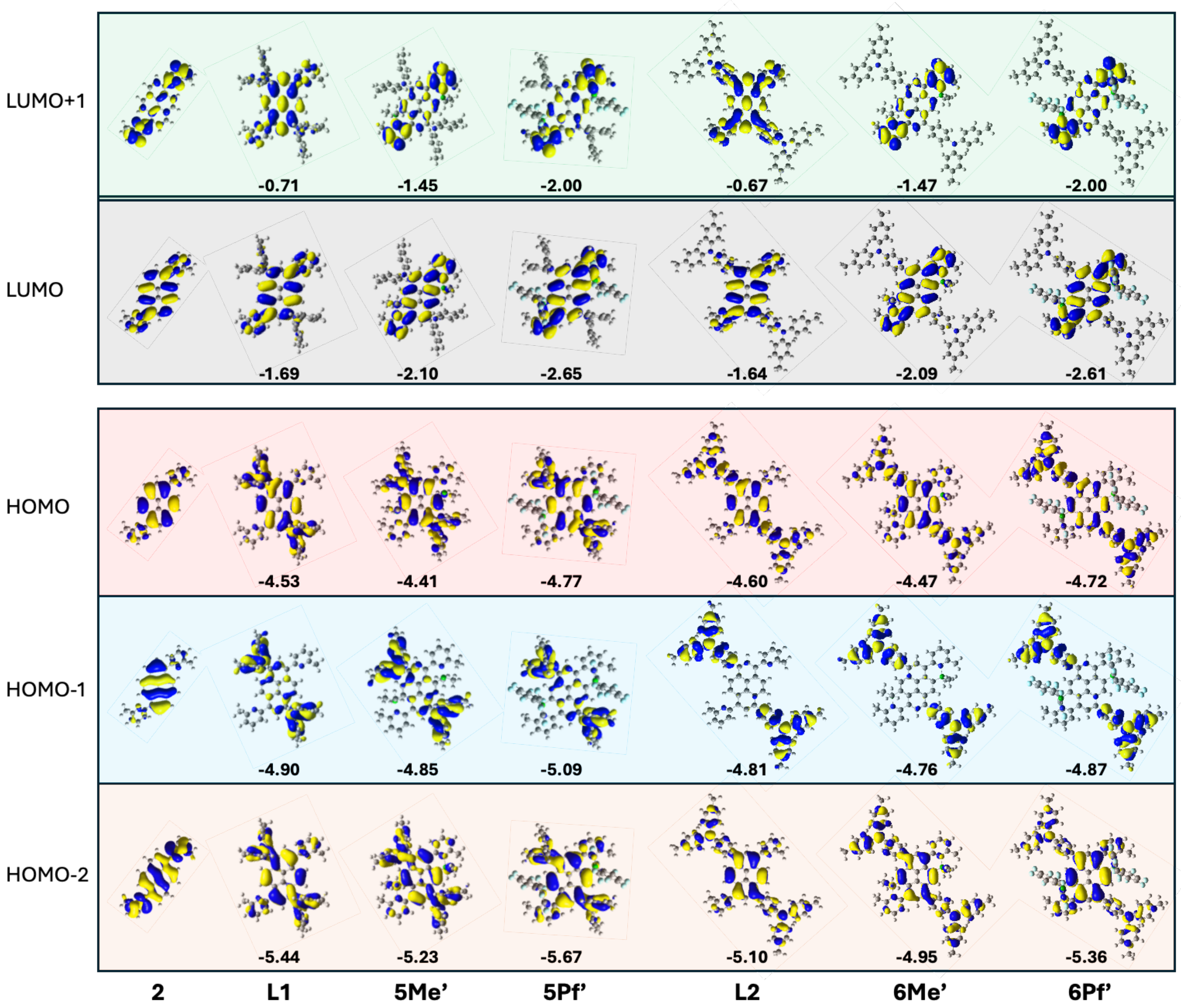

Figure S55. Calculated orbital energy levels and frontier orbital plots of free ligands and B←N Lewis pair complexes (RB3LYP/6-31G(d), energies given in eV; Me in place of longer alkyl and ^{t}Bu groups, Pf = pentafluorophenyl).

Table S5. Comparison of computed *cis*- and *trans*-isomers of **5-Pf'** (RB3LYP/6-31G(d))

	trans-**5-Pf**	*cis*-**5-Pf**	**Difference**
**E(RB3LYP)** (Hartree/Particle)	**-5340.51068835**	**-5340.50879767**	**-0.00189068**
E (Thermal) [kcal/mol]	721.118	721.076	0.048
C_v [cal/mol-K]	346.851	346.938	-0.087
S [cal/mol-K]	478.590	478.011	0.579
S [J/mol-K]	2002.42056	1999.99802	2.4225
Zero-point correction (Hartree/Particle)	1.058932	1.058852	0.000080
Thermal correction to Energy (Hartree/Particle)	1.149175	1.14910	0.000075
Thermal correction to Enthalpy (Hartree/Particle)	1.150120	1.150051	0.000069
Thermal correction to Gibbs Free Energy (Hartree/Particle)	0.922726	0.922933	-0.000207
Sum of electronic and zero-point Energies (Hartree/Particle)	-5339.451756	-5339.449946	-0.001810
Sum of electronic and thermal Energies (Hartree/Particle)	-5339.361513	-5339.359691	-0.001809
Sum of electronic and thermal Enthalpies (Hartree/Particle)	-5339.360569	-5339.358747	-0.001822 **(-4.78 kJ/mol)**
Sum of electronic and thermal Free Energies (Hartree/Particle)	-5339.587962	-5339.585865	-0.002097 **(-5.51 kJ/mol)**

Table S6. Comparison of computed thermodynamic parameters for *cis*- and *trans*-isomers of **5-Pf'** at different temperatures (RB3LYP/6-31G(d))

Temperature (K)	ΔΔH (kJ/mol)	ΔΔS (J/mol K)	ΔΔG (kJ/mol)	K_{eq}	% **trans-5-Pf**	% **trans-5-Pf**
298	4.784	-2.423	5.506	9.227	90.2	88.1 from E(RB3LYP)
190	4.784	-2.423	5.244	27.65	96.5	95.9 from E(RB3LYP)
163	4.784	-2.423	5.179	45.66	97.9	97.5 from E(RB3LYP)

Table S7a. TD-DFT data for boranes **L1’**, **5-Me’** and **5-Pf’** (rcam-B3LYP/6-31G(d))

Compd	Transition [a]	E_{ex} (eV)	λ (nm)	Oscillator strength f	Assignment (coefficients, c) [b]
**L1’**	**$S_0 \rightarrow S_1$**	**3.04**	**408**	**0.7865**	**H → L (0.69)**
	$S_0 \rightarrow S_4$	4.09	303	0.5311	H-2 → L (0.62)<br>H → L+1 (0.26)
	$S_0 \rightarrow S_6$	4.48	277	0.2270	H-3 → L (0.39)<br>H-2 → L+1 (-0.21)<br>H-1 → L+2 (0.13)<br>H-1 → L+4 (-0.12)<br>H-1 → L+5 (0.14)<br>H-1 → L+7 (0.22)<br>H → L+1 (-0.26)<br>H → L+8 (-0.21)<br>H → L+10 (0.13)
**5-Me’**	**$S_0 \rightarrow S_1$**	**2.56**	**485**	**0.6697**	**H → L (0.69)**
	$S_0 \rightarrow S_5$	3.67	338	0.4799	H-4 → L (-0.20)<br>H-2 → L (0.44)<br>H → L+4 (0.47)
	$S_0 \rightarrow S_9$	4.16	298	0.3509	H-4 → L (0.50)<br>H-2 → L+4 (0.18)<br>H-1 → L+1 (-0.19)<br>H-1 → L+3 (0.11)<br>H → L+2 (0.12)<br>H → L+4 (0.30)
	$S_0 \rightarrow S_{13}$	4.54	273	0.2073	H-2 → L+2 (-0.11)<br>H-2 → L+5 (0.16)<br>H-2 → L+9 (0.14)<br>H-1 → L+1 (-0.10)<br>H-1 → L+6 (-0.33)<br>H-1 → L+8 (0.22)<br>H → L+5 (0.42)<br>H → L+9 (0.19)
	$S_0 \rightarrow S_{15}$	4.644	267	0.4982	H-2 → L+7 (0.20)<br>H-1 → L+1 (0.11)<br>H-1 → L+6 (-0.25)<br>H-1 → L+8 (-0.30)<br>H → L+7 (0.46)
**5-Pf’**	**$S_0 \rightarrow S_1$**	**2.42**	**512**	**0.4972**	**H → L (0.69)**
	$S_0 \rightarrow S_3$	3.30	376	0.2587	H-3→ L (-0.24)<br>H-2 → L (0.57)<br>H → L+2 (0.10)<br>H → L+4 (0.20)

$S_0 \rightarrow S_5$	3.55	349	0.2652	H-3 → L (-0.31)<br>H-2 → L (-0.34)<br>H→ L+4 (0.46)
$S_0 \rightarrow S_7$	3.88	319	0.1460	H-2 → L (-0.10)<br>H-2 → L+2 (0.17)<br>H-1 → L+1 (-0.18)<br>H → L+2 (0.60)<br>H → L+4 (-0.13)
$S_0 \rightarrow S_9$	4.05	306	0.4092	H-14 → L (-0.11)<br>H-3 → L (0.43)<br>H-2 → L+4 (-0.12)<br>H-1 → L+1 (0.28)<br>H-1 → L+3 (-0.11)<br>H → L+2 (0.24)<br>H → L+4 (0.31)
$S_0 \rightarrow S_{11}$	4.18	296	0.1670	H-3 → L (-0.22)<br>H-2 → L+4 (-0.10)<br>H-1 → L+1 (0.57)<br>H → L+4 (-0.28)

[a] Only transitions with oscillator strengths >0.1 are presented. [b] % contribution = 2 c^2 * 100%.

Table S7b. TD-DFT data for boranes **L2'**, **6-Me'** and **6-Pf'** (rcam-B3LYP/6-31G(d))

Compd	Transition [a]	E_{ex} (eV)	λ (nm)	Oscillator strength f	Assignment (coefficients, c) [b]
**L2**	**$S_0 \rightarrow S_1$**	**3.24**	**383**	**1.2410**	**H-2 → L (-0.22)**<br>**H → L (0.64)**
	$S_0 \rightarrow S_4$	4.11	302	1.1217	H-2 → L (0.52)<br>H-2 → L+10 (-0.12)<br>H-1→ L+3 (-0.25)<br>H → L (0.12)<br>H → L+1 (0.27)<br>H → L+10 (-0.13)
	$S_0 \rightarrow S_8$	4.53	273	0.3789	H-2 → L+8 (-0.3)<br>H-1 → L+8 (0.45)<br>H → L+8 (-0.36)
**6-Me'**	**$S_0 \rightarrow S_1$**	**2.64**	**470**	**0.7838**	**H-2 → L (0.19)**<br>**H → L (0.66)**
	$S_0 \rightarrow S_6$	3.75	331	1.0640	H-2 → L (0.49)<br>H-1 → L+5 (-0.15)<br>H → L (-0.13)<br>H → L+2 (-0.12)<br>H → L+4 (-0.38)
	$S_0 \rightarrow S_7$	3.93	315	0.1655	H-2 → L (0.18)<br>H-2 → L+2 (0.21)<br>H → L+2 (0.60)
	$S_0 \rightarrow S_9$	4.16	298	0.8688	H-3 → L (0.41)<br>H-2 → L (-0.25)<br>H-1 → L+5 (-0.17)<br>H → L (0.12)<br>H → L+2 (0.12)<br>H → L+4 (-0.38)
	$S_0 \rightarrow S_{13}$	4.49	276	0.1248	H-4 → L+1 (0.10)<br>H-3 → L (-0.19)<br>H-3 → L+1 (0.10)<br>H-2 → L+1 (0.13)<br>H-2 → L+2 (0.12)<br>H-2 → L+4 (0.29)<br>H-2 → L+11 (0.10)<br>H-1 → L+1 (0.24)<br>H-1 → L+5 (-0.28)<br>H → L+4 (0.12)<br>H → L+10 (0.21)
	$S_0 \rightarrow S_{16}$	4.53	274	0.4217	H-2 → L+9 (-0.33)<br>H-1 → L+9 (0.45)<br>H → L+9 (0.30)

	**$S_0 \rightarrow S_1$**	**2.58**	**480**	**0.6740**	**H-2 → L (-0.33)**<br>**H → L (0.61)**
	$S_0 \rightarrow S_3$	3.33	372	0.3346	H-3→ L (0.33)<br>H-2 → L (0.51)<br>H-2 → L+4 (0.11)<br>H → L (0.26)
	$S_0 \rightarrow S_5$	3.58	346	0.2177	H-3 → L (0.37)<br>H-2 → L (-0.29)<br>H-2 → L+4 (0.21)<br>H → L (-0.18)<br>H → L +4 (-0.33)
**6-Pf'**	$S_0 \rightarrow S_7$	3.90	318	0.8156	H-3 → L (-0.12)<br>H-2 → L+2 (-0.28)<br>H-1 → L+6 (0.14)<br>H → L (0.11)<br>H → L+2 (0.50)<br>H → L+4 (-0.24)
	$S_0 \rightarrow S_8$	4.10	303	0.8685	H-3 → L (0.33)<br>H-2 → L+2 (-0.23)<br>H-1 → L+1 (-0.12)<br>H-1 → L+6 (-0.16)<br>H → L+2 (0.27)<br>H → L+4 (0.39)
	$S_0 \rightarrow S_{19}$	4.59	270	0.4142	H-2 → L+14 (-0.22)<br>H-1 → L+13 (-0.14)<br>H-1 → L+14 (0.43)<br>H → L+14 (-0.41)

[a] Only transitions with oscillator strengths >0.1 are presented. [b] % contribution = 2 c^2 * 100%.

7. Aggregation in THF-Water Solvent Mixtures

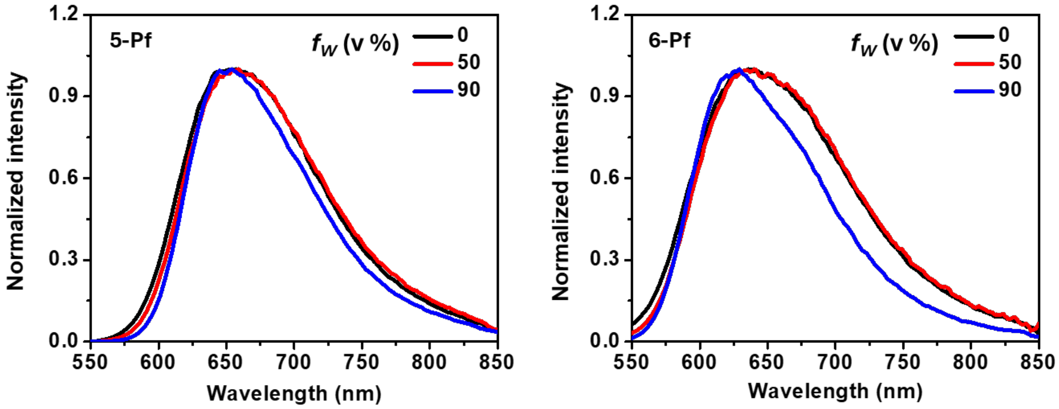

Figure S56. Normalized fluorescence intensity of **5-Pf** and **6-Pf** in THF-water solvent mixtures.

Dynamic light scattering (DLS) measurements were performed at 25±1 °C with a Malvern Zetasizer Nano-ZS instrument, equipped with a 4 mW, 633 nm He-Ne laser and an Avalanche photodiode detector at an angle of 173°.

Table S8. Dynamic light scattering data for particles of **5-Pf** and of **6-Pf** in THF-water solvent mixtures.

			Intensity		Volume	
	Z-Avg d [nm]	PDI	Avg (I) d [nm]	Std Dev d [nm]	Avg (V) d [nm]	Std Dev d [nm]
**5-Pf** 50% H_2O	567.1	0.125	645.7	221.5	700.2	274.8
**5-Pf** 90% H_2O	263.9	0.167	314.2	144.1	309.0	132.8
**6-Pf** 50% H_2O	656.1	0.122	724.6	197.5	789.6	233.6
**6-Pf** 90% H_2O	286.3	0.148	324.1	128.3	312.1	116.8

5-Pf, 50% H_2O | **5-Pf, 90% H_2O**

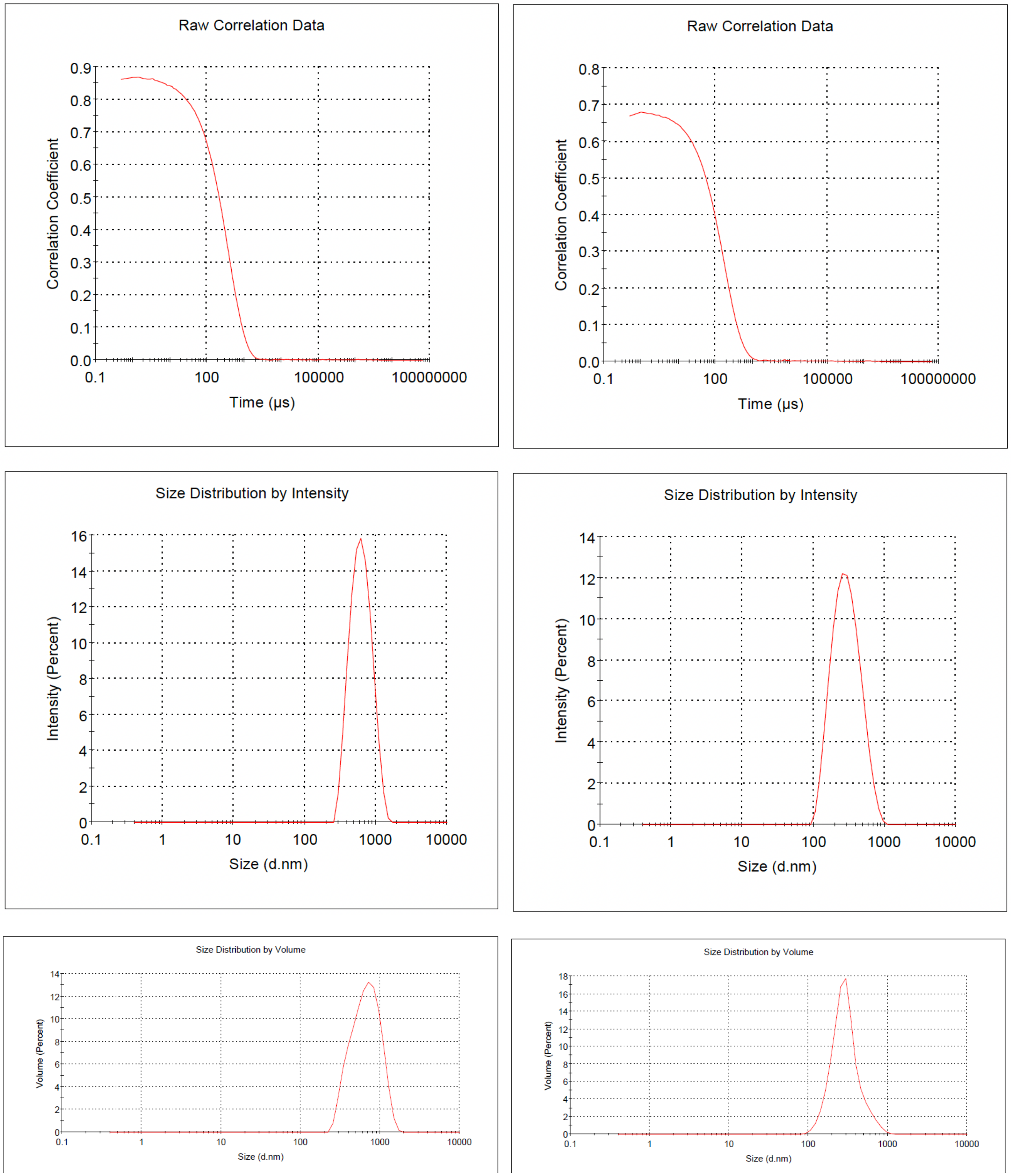

Figure S57. Raw dynamic light scattering correlation data (top), intensity-based size distribution (middle), and volume-based size distribution (bottom) of **5-Pf** particles in THF-water solvent mixtures.

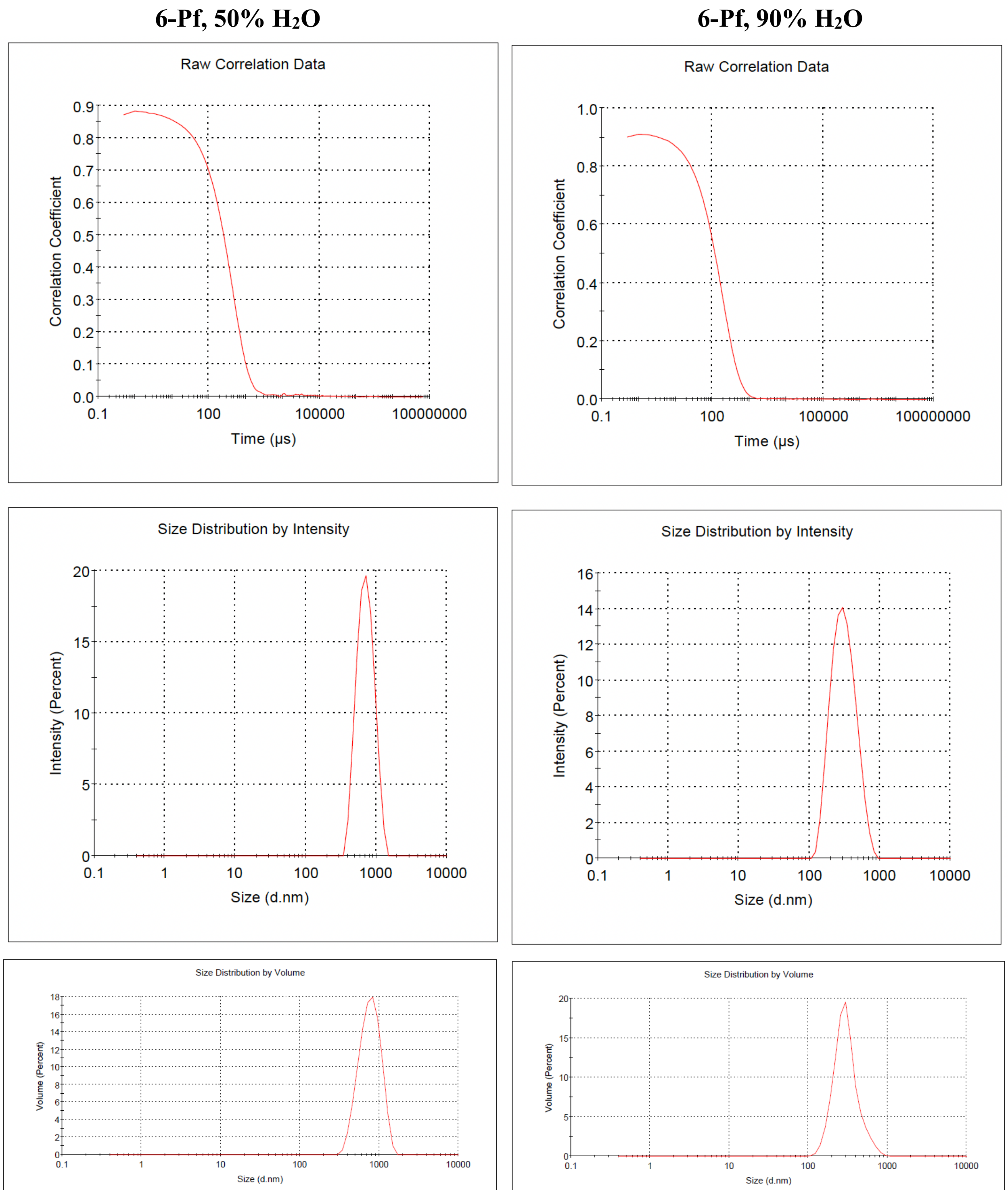

Figure S58. Raw dynamic light scattering correlation data (top) and intensity-based size distribution (bottom) of **6-Pf** particles in THF-water solvent mixtures.

8. Live Cell Imaging Studies

Cell Culture

Madin-Darby Canine Kidney cells (MDCK – NBL2; ATCC® CCL-34™) were cultured in Dulbecco's Modified Eagle Medium (DMEM) supplemented with 10% (v/v) Fetal Bovine Serum (FBS), 100 I.U./mL Penicillin, and 100 µg/mL Streptomycin. For all live cell experiments, cells were trypsinized into a single cell suspension, pelleted out of trypsin, resuspended in DMEM, and plated into 35 mm MatTek glass bottom microwell dishes (P35G-1.5-20-C) at a density of 15,000 cells per well. Plated cells were grown overnight at 37 ºC in 5% CO_2 prior to experimentation.

Vital Staining of MDCK Cells

Prior to staining, a 0.5 mM stock solution of **5-Pf** in 100% DMF was diluted 5000-fold into DMEM, giving a final concentration of 100 nM **5-Pf** (DMF from the **5-Pf** solution constituted a negligible 0.02% of the culture/staining media). DMEM with a final concentration of 100 nM of **5-Pf**, 100 nM of LysoTracker Green, and 10 µM of Verapamil was prepared for co-staining experiments. For live cell staining, DMEM was removed, replaced with either staining solutions or DMEM, incubated for 1 hour at 37 ºC, and then the staining solution was removed, the cells were rinsed with PBS, and DMEM containing LysoTracker green and 10 µM of Verapamil was added back to the MatTek dishes. Cells were then imaged by confocal microscopy. To access the dependence of vesicular pH on staining 20 mM NH_4Cl (a weak penetrating base) was added during all staining steps.

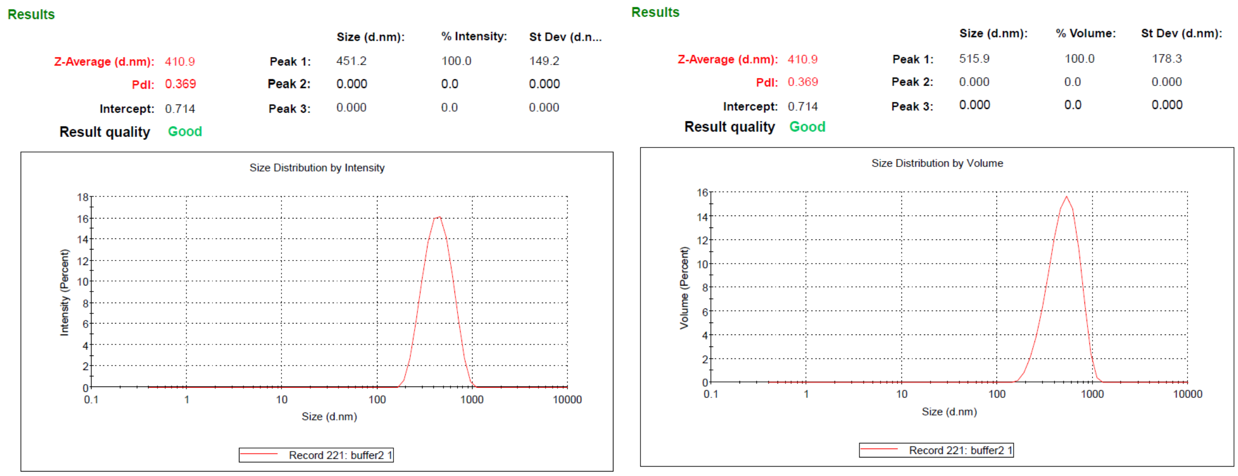

Figure S59. Dynamic light scattering data for particles generated upon diluting a 0.5 mM solution of **5-Pf** in DMF to 100 nM in DMEM solution; (left) intensity-based size distribution and (right) volume-based size distribution. The data are indicative of aggregate formation of the **5-Pf** dye in the medium used for cell imaging studies.

Laser Scanning Confocal Microscopy

Live cell imaging was conducted using a Zeiss LSM980 AxioObserver 7 with an Airyscan 2 detector mounted to an inverted microscope equipped with an incubator housing that maintained the temperature and CO_2. Primary documentation was conducted using a Zeiss Plan-Apo 63x 1.4 NA objective and Airyscan 2 detection mode. Laser strength was set as follows: 561 nm – 2% and 488 nm – 0.10%; Scan Zoom 3x; pixel dwell time 0.99 µs; bidirectional scanning direction. All settings were kept constant across all experiments.

Photostability under Live Cell Imaging Conditions

To assess the photostability of the **5-Pf** dye, we used a point source laser scanning confocal microscope and live **5-Pf** and LysoTracker co-stained MDCK cells to measure the fluorescence intensity of individual vesicles over time. Using a 561 nm diode (25 mW) laser set to 2.0% output, the fluorescence intensity of individual vesicles was determined every 2 seconds and plotted to a one phase decay function. Under these conditions, the dye's half-life was approximately 24 seconds (Figure S60, top). Increasing the time interval between individual readings to 1 minute increased the dye's half-life to 178 seconds (Figure S60, bottom). These data suggest that the dye could be used for both short- and long-term live cell imaging. Importantly, even under these conditions, the fluorescence intensity of vesicles was more than adequate to monitor individual vesicles for extended periods of time.

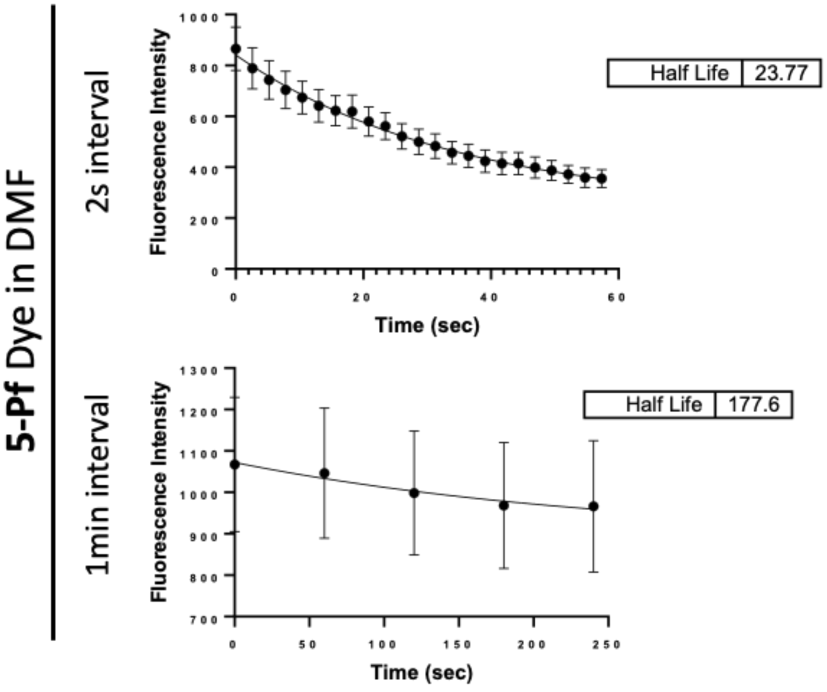

Figure S60. Photostability of **5-Pf** dye under live cell imaging conditions.

9. Electrochemiluminescence Measurements

A three-electrode configuration was employed for conducting electrochemiluminescence (ECL) experiments and the corresponding cyclic voltammograms (CVs) and differential pulse voltammograms (DPVs). A 2 mm diameter disk platinum (Pt) working electrode (WE) was sealed in a glass tube with two platinum wire coils as the counter electrode (CE) and quasi-reference electrode (RE). The experimental potentials were all recorded relative to Fc/Fc^+ redox couple, which is taken as 0.39 V vs SCE.[S6] Before utilization, the working electrode (WE) underwent polishing, which involved the application of a 0.3 and 0.05 μm alumina mixture (Buehler Ltd.) with water on a polishing pad (Buehler Ltd. Lake Bluff, IL) to achieve a reflective surface. Afterward, the electrode was rinsed with copious deionized water. Electrochemical polishing of the Pt working electrode was done by scanning the applied potentials between -0.84 V and 0.75 V in 0.1 M sulfuric acid (H_2SO_4) for 400 cycles. Finally, the electrode was rinsed with copious deionized water and dried using argon (Ar) gas. Both CE and RE were prepared by first ultrasonicating in acetone, isopropyl alcohol, and lastly ultrapure water for 5 minutes each. Finally, they were dried in a 120 °C oven for 10 minutes and cooled off before use.

ECL experiments were conducted within a glass cylinder tube equipped with a flat Pyrex window at the bottom, allowing for the transmission of ECL light. To mitigate the influence of oxygen and moisture, the ECL cell was assembled in a glovebox filled with nitrogen gas (Model Nexus 1, Vacuum Atmospheres Company, Hawthorne, CA). An airtight cap housed the electrodes with electrical connection wires to allow electrical signals to transmit to and from the ECL cell. The ECL cell was filled with a solution comprising 3 mL of DCM containing 0.20 mM of **5-Pf** and 0.1 M $TBAPF_6$. An additional 5 mM of benzoyl peroxide (BPO) was added to the cell for coreactant studies. The cell assembly was subsequently removed from the glovebox for the actual ECL experiments.

Figure S61a illustrates the ECL instrumentation used for general measurements. CVs, DPVs, and ECL-voltage curves were performed using an Autolab electrochemical workstation (PGSTAT 30, Metrohm, Switzerland). An ECL signal, measured as photocurrent with a corresponding gain factor of 6.86×10^5 was detected using a photomultiplier tube (PMT) (R3896, Hamamatsu, Japan) biased at -750 V.[S7, S8] This photocurrent from the PMT was subsequently converted into voltage using a picoammeter (Keithley 6487, Cleveland, OH). The output ECL signal, in conjunction with electrochemical potential and current data, was acquired through an auxiliary channel of an electrochemical workstation, which was controlled by a Metrohm Nova software program[S7]. Potential pulsing experiments between the first oxidation and first reduction electrochemical peaks were performed at potential values 100 mV beyond the respective redox potentials. A pulsing ECL plot depicts the relationship between ECL intensity and time during potential steps. This curve is widely utilized to establish a connection between the acquired ECL spectra and the CV data, as shown in Figure S61a.

A Princeton Instruments Spectrograph (Model SP2300i, Trenton, NJ) coupled with a CCD camera (Andor DU401-BR-DD-352, Oxford Instruments, UK) cooled at -65 °C was employed to capture ECL spectra, as depicted in Figure 57b. During a complete CV process, a single accumulation ECL spectrum was acquired by collecting all the emission generated over the entire CV scan. Furthermore, spooling ECL spectra were collected individually at 1 s time intervals while performing the CV scan. These spectra were then assembled into a three-dimensional plot using a custom-made MATLAB program[S9]. Synchronization of electrochemical control and ECL spectroscopy was achieved using a voltage output from the CHI electrochemical analyzer (Model 610A, CH Instruments Inc., Austin, TX), acting as an external trigger for the CCD camera through programming (Figure S61b). ECL spectra were collected and processed using AndorSolis software (Andor, Oxford Instruments, UK). The spectrograph-CCD camera system's wavelength calibration was performed using a mercury argon lamp (HG-1 lamp, Ocean Insight, Orlando, FL).

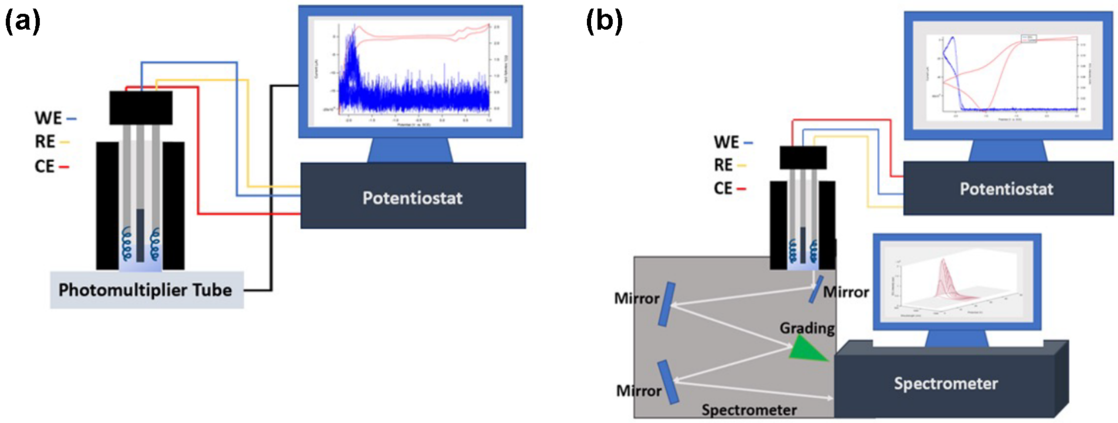

Figure S61. Demonstration of equipment for electrochemiluminescence experiment with a photomultiplier tube and spectrometer set.

Absolute ECL Quantum Efficiency Strategy

To determine the absolute ECL quantum efficiency (Φ_{ECL}) it is necessary to find the total number of photons emitted during ECL emission. The number of photons emitted by the ECL system are represented by the photocurrent signal from a calibrated PMT. The instrumental setup in Figure S61a only captures a small portion of the total ECL emission produced. So, to transform a signal originating from the PMT into the total number of photons emitted, it is imperative to fulfill the prescribed set of tasks. These tasks are an important part of the calculation process which first includes measuring the distance from the electrode surface to the PMT surface and the surface area of the PMT. The required distance from the light source should be at least 10 mm or more since the Pt electrode surface exhibits a diameter of 2 mm. This permits an error calculation of less than 1%.[S10] Subsequently, we must determine the reflectivity of the electrode at the maximum wavelength (%). This can be achieved by initially identifying the maximum wavelength detected within the experimental accumulation spectra. The percentage of reflectivity will be dependent on the Pt electrode that is used. Next, we input all other required values on the MATLAB App

developed in our lab, which includes optical density (OD), electrode diameter (m), sensitivity of PMT (nA/V), and lastly the absorption correction (determined by fitting and integrating the accumulation spectra using Igor software). Once all the values are entered into the MATLAB App, we then import the experimental and background data along with the calibrated PMT responsivity data (photons/electron) and an ECL spectrum. The PMT response spans from 200 nm to 900 nm, but we do not integrate the entire range due to the availability of the calibration curve (PMT response curve). To determine the specific wavelength range for integrating the PMT photocurrent, we utilize the ECL spectrum. This enable identification of the wavelength range at which the luminophore emits, allowing us to calculate the correction factor and ultimately determine the Φ_{ECL} %. Comprehensive details of the experimental methods and information regarding the calibration of absolute irradiance can be found in references [S11-S12]. The experiment's total electric charge was determined by summing the electrons introduced into the system. This was derived by integrating the electrochemical current over the duration of the experiment, as depicted in Equation 1. The number of electrons is derived from the integrated electrochemical current measured by the potentiostat. This was calculated by dividing the total charge by the electric charge of a single electron (e) which is -1.602×10^{-19} C (Equation 2). Finally, the Φ_{ECL} referring to the measure of how efficiently a given electrochemical reaction generates light in an ECL system is quantified by the ratio of the number of emitted photons (light) to the number of electrons (Equation 3).

$$Q_{\text{total}} = \int_{t_{\text{initial}}}^{t_{\text{final}}} I(t)dt \tag{1}$$

$$\text{Total electrons} = \frac{Q_{\text{total}}}{e} \tag{2}$$

$$\Phi_{\text{ECL}} = \frac{\text{Total photons}}{\text{Total electrons}} \times 100\% \tag{3}$$

Classic electrochemical techniques were used to acquire differential pulse voltammetry (DPV) and cyclic voltammetry (CV) data, long with ECL-voltage curves, to study the redox properties of ECL solutions containing **5-Pf**. DPV is a slow scan which makes sure the potential at the working electrode is incremented in small steps and a pulse of potential is applied at each step. The resulting current measurement is taken differentially, meaning the current is measured at the end of a potential pulse with the initial potential. This differential aspect enhances the sensitivity to changes in concentration of reactive intermediates near the electrode surface.[S13] In the slightly faster CV scan, current is graphed with applied potential cycled back and forth at a specific scan rate to generate a cyclic voltammogram, showing electron transfer and electrochemical behavior of our analyte to study their reaction mechanisms.[S13] Pulsing ECL-voltage experiments were conducted to see whether the intensity of ECL could be enhanced by rapid stepping between the oxidation and reduction potentials.

An accumulation ECL spectrum of the **5-Pf** annihilation system acquired during a cyclic potential scan spanning from -2.20 V to +1.00 V is displayed in Figure S62a. An evident peak emerged at ca. 656 nm which corresponds to the predominant emission from the **5-Pf** luminophore, but under

these conditions the ECL signal was weak. A spooling ECL spectrum of a 0.1 mM solution of **5-Pf** in dichloromethane (DCM) containing 5 mM BPO, and 0.1 M of $TBAPF_6$ with a scan rate of 0.025 V/s is displayed in Figure S62b, and the corresponding photoluminescence spectra are illustrated in Figure S62c.

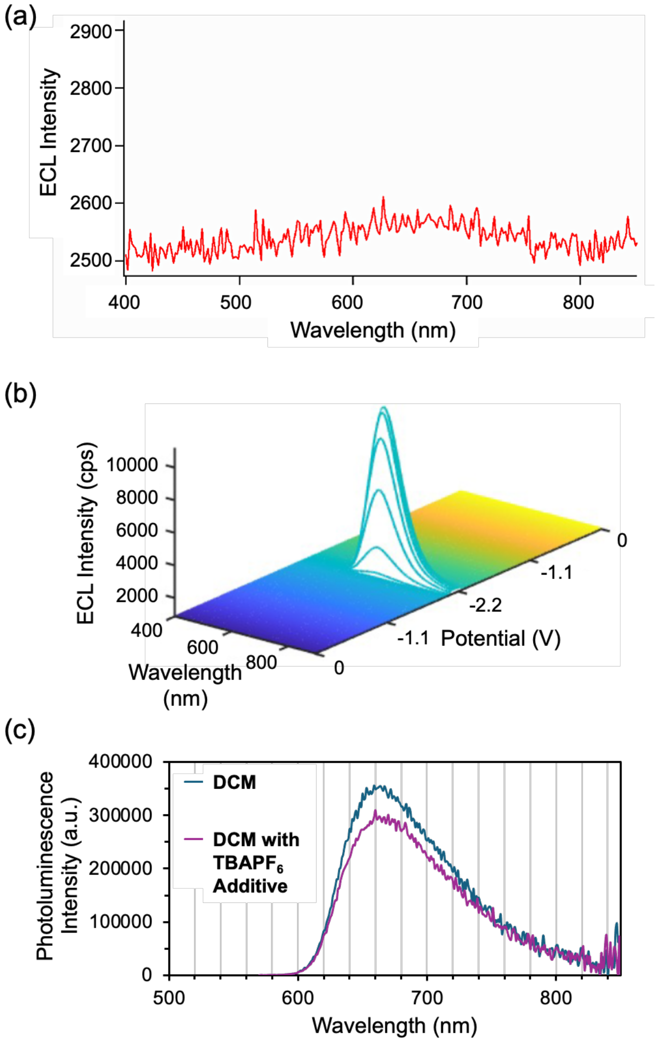

Figure S62. (a) ECL annihilation experiment showing the single CV accumulation ECL spectrum of a 0.2 mM solution of **5-Pf** at a 2 mm platinum electrode submerged in a 3 mL electrolyte solution of dichloromethane (DCM) containing 0.1 M of $TBAPF_6$ with a scan rate of 0.1 V/s; (b) spooling ECL spectra of a 0.1 mM solution of 5-Pf at a 2 mm platinum electrode submerged in a 3 mL electrolyte solution of dichloromethane (DCM) containing 5 mM BPO, and 0.1 M of $TBAPF_6$ with a scan rate of 0.025 V/s and exposure time for each spectrum is 1s; (c) photoluminescence spectra of **5-Pf** in DCM with and without added $TBAPF_6$ (0.1 M).

Table S9. Results from ECL experiments for **5-Pf** along with quantum efficiency values (Φ_{ECL}).

**ECL method**	**ECL efficiency (%), 5-Pf**
Annihilation ECL in CV scan	0.00016 ± 0.00002
Annihilation ECL in Pot. Pulsing (1st ox 1st red)	0.0304 ± 0.0004
Annihilation ECL in Pot. Pulsing (1st red 2nd ox)	0.0377 ± 0.0001
Annihilation ECL in Pot. Pulsing (1st ox 2nd red)	0.0313 ± 0.0004
Annihilation ECL in Pot. Pulsing (2nd ox 2nd red)	0.269 ± 0.002
BPO Coreactant in CV scan	0.9 ± 0.1
BPO Coreactant in Pot. Pulsing	0.40 ± 0.02

10. Optimized Structures and Energies for Computed Molecules

Table S9. Coordinates for Optimized Structure of **5-Me'** in the ground state (S_0).
E(RB3LYP) = -2588.98237246 Hartree.

```
# opt freq rb3lyp/6-31g(d) geom=connectivity
```

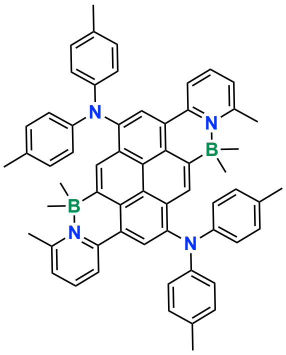

Center Number	Atomic Number	Atomic Type	Coordinates (Angstroms) X	Y	Z
1	7	0	-4.309157	-0.337453	0.156011
2	7	0	-0.831611	4.634764	1.119720
3	6	0	-1.838225	-0.364204	-0.051152
4	6	0	-0.593762	1.744361	0.417623
5	6	0	-4.591045	-1.348180	1.115907
6	6	0	-0.638739	-2.439041	-0.650406
7	6	0	-0.612031	0.350922	0.095337
8	6	0	-1.825049	2.435190	0.520178
9	6	0	-3.022614	1.700437	0.455844
10	1	0	-3.973817	2.183157	0.644704
11	6	0	-3.050216	0.335906	0.192946
12	6	0	-1.803561	-1.731817	-0.451941
13	1	0	-2.757332	-2.211198	-0.638853
14	6	0	-4.010140	-1.317396	2.390712
15	1	0	-3.351265	-0.498641	2.661058
16	6	0	-1.893610	3.899693	0.651362
17	6	0	-5.444361	-2.418274	0.795692
18	1	0	-5.896792	-2.465145	-0.189907
19	6	0	-5.127196	-3.396468	3.007251
20	6	0	-5.710472	-3.412204	1.731631
21	1	0	-6.375809	-4.228376	1.457355
22	6	0	-4.273027	-2.331537	3.311084
23	1	0	-3.807431	-2.283269	4.293195
24	6	0	-5.393200	-4.503232	4.000563
25	6	0	-3.081763	4.547639	0.260446
26	1	0	-3.864197	3.971342	-0.213220
27	6	0	-1.828322	-4.839407	-0.632632
28	1	0	-2.641143	-4.208959	-0.254566
29	1	0	-2.305297	-5.570586	-1.295451
30	6	0	-3.241692	5.905577	0.448293
31	1	0	-4.159135	6.402999	0.146929

32	6	0	-1.006654	5.971355	1.352734
33	6	0	-2.202963	6.613592	1.038527
34	1	0	-2.293055	7.674818	1.242561
35	6	0	0.076285	6.826063	1.962261
36	1	0	0.661401	6.293165	2.707207
37	1	0	-0.389429	7.694656	2.436654
38	6	0	-5.345936	0.213246	-0.644788
39	6	0	-0.868201	-3.653285	-2.953164
40	1	0	-0.891431	-4.556199	-3.579001
41	1	0	-1.824349	-3.138633	-3.120226
42	6	0	-5.062915	0.654586	-1.948415
43	1	0	-4.054530	0.551604	-2.337212
44	5	0	-0.680275	-3.905234	-1.341938
45	6	0	-6.657735	0.351950	-0.165074
46	1	0	-6.897092	0.030716	0.843375
47	6	0	-7.650705	0.901275	-0.974429
48	1	0	-8.658856	0.998571	-0.576554
49	6	0	-6.061821	1.217639	-2.737107
50	1	0	-5.813540	1.550441	-3.742923
51	6	0	-7.378834	1.346538	-2.273729
52	6	0	-8.466686	1.917031	-3.153386
53	7	0	4.309154	0.337452	-0.156021
54	7	0	0.831607	-4.634771	-1.119707
55	6	0	1.838222	0.364202	0.051154
56	6	0	0.593760	-1.744365	-0.417617
57	6	0	4.591043	1.348179	-1.115916
58	6	0	0.638736	2.439036	0.650418
59	6	0	0.612029	-0.350925	-0.095331
60	6	0	1.825048	-2.435192	-0.520180
61	6	0	3.022611	-1.700435	-0.455852
62	1	0	3.973814	-2.183152	-0.644720
63	6	0	3.050213	-0.335906	-0.192951
64	6	0	1.803558	1.731814	0.451947
65	1	0	2.757329	2.211195	0.638859
66	6	0	4.010130	1.317405	-2.390715
67	1	0	3.351244	0.498658	-2.661060
68	6	0	1.893614	-3.899694	-0.651373
69	6	0	5.444367	2.418269	-0.795701
70	1	0	5.896801	2.465135	0.189897
71	6	0	5.127194	3.396472	-3.007255
72	6	0	5.710477	3.412201	-1.731636
73	1	0	6.375816	4.228370	-1.457358
74	6	0	4.273016	2.331550	-3.311085
75	1	0	3.807409	2.283292	-4.293191
76	6	0	5.393230	4.503227	-4.000569
77	6	0	3.081782	-4.547635	-0.260493
78	1	0	3.864223	-3.971334	0.213157
79	6	0	1.828323	4.839398	0.632675
80	1	0	2.641149	4.208949	0.254622
81	1	0	2.305292	5.570579	1.295495
82	6	0	3.241716	-5.905570	-0.448353
83	1	0	4.159172	-6.402986	-0.147019
84	6	0	1.006650	-5.971362	-1.352724
85	6	0	2.202974	-6.613590	-1.038557

86	1	0	2.293068	-7.674815	-1.242595
87	6	0	-0.076305	-6.826079	-1.962206
88	1	0	-0.661423	-6.293199	-2.707166
89	1	0	0.389391	-7.694694	-2.436573
90	6	0	5.345933	-0.213242	0.644782
91	6	0	0.868190	3.653256	2.953186
92	1	0	0.891401	4.556162	3.579034
93	1	0	1.824347	3.138617	3.120245
94	6	0	5.062913	-0.654569	1.948414
95	1	0	4.054529	-0.551582	2.337212
96	5	0	0.680271	3.905221	1.341963
97	6	0	6.657730	-0.351952	0.165066
98	1	0	6.897085	-0.030727	-0.843386
99	6	0	7.650701	-0.901271	0.974424
100	1	0	8.658851	-0.998573	0.576548
101	6	0	6.061820	-1.217618	2.737109
102	1	0	5.813541	-1.550410	3.742928
103	6	0	7.378832	-1.346523	2.273729
104	6	0	8.466684	-1.917011	3.153388
105	1	0	0.767198	7.192247	1.197821
106	1	0	-0.767218	-7.192223	-1.197748
107	1	0	8.902935	-1.147641	3.805215
108	1	0	9.284330	-2.337344	2.557722
109	1	0	8.083015	-2.709921	3.805332
110	1	0	-8.902940	1.147664	-3.805213
111	1	0	-9.284330	2.337366	-2.557718
112	1	0	-8.083016	2.709941	-3.805329
113	1	0	-0.087389	-3.001187	-3.369389
114	1	0	-1.459048	-5.387432	0.247172
115	1	0	0.087388	3.001140	3.369402
116	1	0	1.459060	5.387421	-0.247136
117	1	0	6.452295	4.786458	-4.015249
118	1	0	4.821746	5.408893	-3.756101
119	1	0	5.111981	4.204586	-5.015956
120	1	0	-5.112494	-4.204382	5.016040
121	1	0	-4.821243	-5.408689	3.756425
122	1	0	-6.452155	-4.786889	4.014823

Table S10a. Coordinates for Optimized Structure of **5-Pf'** in the ground state (S_0).
E(RB3LYP) = -5340.51068835 Hartree.

```
# opt freq rb3lyp/6-31g(d) geom=connectivity
```

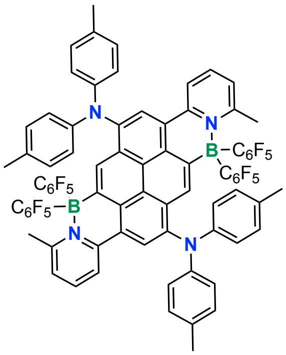

Center Number	Atomic Number	Atomic Type	Coordinates (Angstroms) X	Y	Z
1	9	0	-4.489968	-0.054534	-2.030778
2	9	0	-6.489576	-1.530648	-1.049086
3	9	0	-2.985183	-4.586429	-1.826932
4	9	0	-3.552386	-1.498112	2.693700
5	9	0	-3.038520	-4.693582	-4.456062
6	9	0	-3.796208	-2.522497	-5.949387
7	9	0	-4.514156	-0.206806	-4.668133
8	9	0	-8.335730	-0.285053	0.406001
9	9	0	-5.449385	-0.295548	4.149601
10	7	0	-2.916264	3.192090	-0.121678
11	9	0	-7.853839	0.357689	3.021704
12	7	0	3.192859	3.551038	-0.578028
13	6	0	-3.661045	3.139557	1.085990
14	6	0	-0.102456	0.700680	-0.071773
15	6	0	1.020309	1.565679	-0.225673
16	6	0	-4.886735	-1.514090	0.722539
17	6	0	-4.118272	5.695642	-3.348823
18	6	0	-1.433158	1.209735	-0.063226
19	6	0	4.156938	4.488948	-0.826643
20	6	0	-2.525831	0.296328	0.021789
21	1	0	-3.521649	0.712316	-0.041523
22	6	0	-2.356141	-1.064901	0.110014
23	6	0	-0.520887	3.430020	-0.458567
24	1	0	-0.709190	4.490278	-0.580149
25	6	0	0.792225	2.930323	-0.511742
26	6	0	-3.377505	3.534329	-2.499417
27	1	0	-3.147765	2.487028	-2.672230
28	6	0	-3.332436	4.040449	-1.191933
29	6	0	-6.148028	-1.194333	0.213854
30	6	0	-4.118787	-1.237070	-2.568914
31	6	0	1.592531	4.946474	-1.702742
32	1	0	0.579958	5.087795	-2.054995
33	6	0	-4.516337	6.581778	-4.506123
34	6	0	-3.381181	-3.454750	-2.460118

35	6	0	-3.741799	-2.306753	-1.744123
36	6	0	1.890275	3.814912	-0.925084
37	6	0	-1.618763	2.613142	-0.199453
38	6	0	-3.778524	-2.454466	-4.613376
39	6	0	-3.028820	2.974581	2.326224
40	1	0	-1.946833	2.908996	2.376394
41	6	0	-4.145302	-1.283678	-3.958445
42	6	0	-3.395191	-3.549956	-3.852519
43	6	0	5.558421	4.346624	-0.290765
44	1	0	5.611193	3.770210	0.630687
45	1	0	5.953132	5.347614	-0.092516
46	6	0	-3.694383	5.375568	-0.969064
47	1	0	-3.675208	5.773519	0.041157
48	6	0	-4.088523	6.182510	-2.035831
49	1	0	-4.370365	7.215261	-1.842336
50	6	0	2.580330	5.852541	-2.037118
51	1	0	2.350059	6.722782	-2.644620
52	6	0	-3.756932	4.356436	-3.556971
53	1	0	-3.794748	3.941665	-4.561951
54	6	0	-5.064353	3.230289	1.058303
55	1	0	-5.575438	3.346154	0.107943
56	6	0	-4.715054	-1.197411	2.076513
57	6	0	-3.782329	2.891710	3.497473
58	1	0	-3.265293	2.756859	4.444840
59	6	0	-7.148216	-0.571083	0.959589
60	6	0	-6.911030	-0.255013	2.291872
61	6	0	3.866264	5.636950	-1.556658
62	1	0	4.661158	6.351207	-1.740469
63	6	0	-5.686478	-0.584628	2.861741
64	6	0	-5.177293	2.988968	3.484797
65	6	0	-5.797873	3.164517	2.238134
66	1	0	-6.882788	3.230632	2.186815
67	6	0	-5.993785	2.898356	4.752646
68	5	0	-3.586373	-2.103920	-0.101068
69	9	0	4.490052	0.054374	2.030016
70	9	0	6.489680	1.530427	1.048237
71	9	0	2.985768	4.586484	1.827026
72	9	0	3.551943	1.498652	-2.694111
73	9	0	3.039170	4.693133	4.456172
74	9	0	3.796558	2.521660	5.949083
75	9	0	4.514169	0.206089	4.667345
76	9	0	8.335303	0.284291	-0.407112
77	9	0	5.448404	0.295581	-4.150295
78	7	0	2.916395	-3.191591	0.122044
79	9	0	7.852784	-0.358371	-3.022763
80	7	0	-3.192767	-3.550677	0.577925
81	6	0	3.661125	-3.140082	-1.085669
82	6	0	0.102475	-0.700287	0.071433
83	6	0	-1.020279	-1.565284	0.225414
84	6	0	4.886611	1.514296	-0.723165
85	6	0	4.118227	-5.692870	3.351032
86	6	0	1.433176	-1.209343	0.062912
87	6	0	-4.156795	-4.488637	0.826579
88	6	0	2.525846	-0.295969	-0.022270

89	1	0	3.521657	-0.711989	0.040905
90	6	0	2.356159	1.065264	-0.110395
91	6	0	0.520969	-3.429549	0.458718
92	1	0	0.709293	-4.489769	0.580596
93	6	0	-0.792167	-2.929873	0.511703
94	6	0	3.378947	-3.531740	2.499857
95	1	0	3.150377	-2.484029	2.671729
96	6	0	3.332541	-4.039206	1.192954
97	6	0	6.147892	1.194208	-0.214665
98	6	0	4.119010	1.236873	2.568356
99	6	0	-1.592445	-4.945828	1.703019
100	1	0	-0.579891	-5.087022	2.055379
101	6	0	4.516212	-6.578167	4.509001
102	6	0	3.381636	3.454635	2.459989
103	6	0	3.742085	2.306728	1.743764
104	6	0	-1.890201	-3.814420	0.925142
105	6	0	1.618827	-2.612708	0.199469
106	6	0	3.778874	2.453890	4.613060
107	6	0	3.028959	-2.975326	-2.325969
108	1	0	1.947015	-2.909052	-2.376139
109	6	0	4.145497	1.283187	3.957895
110	6	0	3.395677	3.549573	3.852408
111	6	0	-5.558227	-4.346560	0.290505
112	1	0	-5.610930	-3.770388	-0.631103
113	1	0	-5.952833	-5.347632	0.092464
114	6	0	3.693117	-5.374881	0.971223
115	1	0	3.672959	-5.773823	-0.038592
116	6	0	4.087184	-6.181093	2.038569
117	1	0	4.367953	-7.214305	1.845978
118	6	0	-2.580208	-5.851912	2.037448
119	1	0	-2.349934	-6.722036	2.645116
120	6	0	3.758301	-4.353105	3.558022
121	1	0	3.797174	-3.937323	4.562544
122	6	0	5.064371	-3.231954	-1.058049
123	1	0	5.575437	-3.347837	-0.107683
124	6	0	4.714633	1.197663	-2.077106
125	6	0	3.782454	-2.893911	-3.497333
126	1	0	3.265446	-2.759427	-4.444770
127	6	0	7.147789	0.570647	-0.960531
128	6	0	6.910306	0.254587	-2.292768
129	6	0	-3.866104	-5.636503	1.556803
130	1	0	-4.660957	-6.350801	1.740628
131	6	0	5.685763	0.584575	-2.862449
132	6	0	5.177331	-2.992375	-3.484720
133	6	0	5.797850	-3.167632	-2.237986
134	1	0	6.882697	-3.234896	-2.186707
135	6	0	5.993838	-2.902452	-4.752609
136	5	0	3.586454	2.104182	0.100686
137	1	0	-6.721470	2.081551	4.694766
138	1	0	-5.356215	2.716027	5.623506
139	1	0	-6.554776	3.823390	4.937925
140	1	0	-5.061778	7.467115	-4.163572
141	1	0	-5.154976	6.047675	-5.218834
142	1	0	-3.637779	6.933705	-5.063743

143	1	0	3.637694	-6.928505	5.067681
144	1	0	5.155981	-6.043978	5.220633
145	1	0	5.060417	-7.464479	4.167009
146	1	0	5.355143	-2.732581	-5.625173
147	1	0	6.713210	-2.077918	-4.699949
148	1	0	6.564336	-3.822969	-4.930934
149	1	0	-6.218092	-3.870725	1.022825
150	1	0	6.218149	3.870928	-1.023286

Table S10b. Coordinates for Optimized Structure of **cis-5-Pf’** in the ground state (S_0). E(RB3LYP) = -5340.50879766 Hartree.

```
# opt freq rb3lyp/6-31g(d) geom=connectivity
```

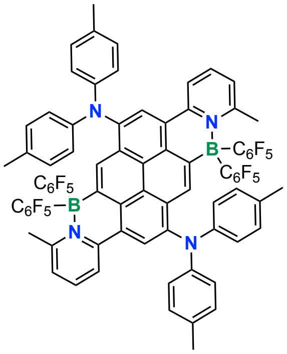

Center Number	Atomic Number	Atomic Type	Coordinates (Angstroms) X	Y	Z
1	1	0	0.748208	4.517365	-0.727554
2	6	0	0.543683	3.453788	-0.694571
3	6	0	0.108342	0.704105	-0.570643
4	6	0	-0.780042	2.981721	-0.643259
5	6	0	1.644176	2.600782	-0.669663
6	6	0	1.445374	1.195376	-0.603242
7	6	0	-1.006700	1.591262	-0.525184
8	6	0	-1.445726	-1.194076	-0.606488
9	6	0	-2.337651	1.086999	-0.374710
10	6	0	-0.108703	-0.702883	-0.572149
11	6	0	-2.525757	-0.269831	-0.485159
12	1	0	-3.522682	-0.682647	-0.418410
13	6	0	-0.544165	-3.452282	-0.701410
14	6	0	1.006310	-1.590150	-0.528019
15	6	0	0.779594	-2.980336	-0.649141
16	6	0	-1.644632	-2.599351	-0.675242
17	1	0	-0.748721	-4.515795	-0.736308
18	6	0	2.525340	0.270873	-0.482823
19	6	0	2.337211	-1.086237	-0.375733
20	1	0	3.522207	0.683579	-0.414298
21	1	0	-2.464642	7.057535	-2.039277
22	6	0	-2.653585	6.107161	-1.548882
23	7	0	-3.166742	3.616213	-0.396890
24	6	0	-3.893140	5.826830	-0.987944

25	6	0	-1.655079	5.158056	-1.446524
26	6	0	-1.896186	3.923443	-0.820511
27	6	0	-4.133844	4.583524	-0.412508
28	1	0	-4.689436	6.562801	-0.988559
29	1	0	-0.678760	5.351422	-1.867885
30	6	0	-5.482588	4.366967	0.224358
31	1	0	-6.214805	4.010000	-0.506770
32	1	0	-5.839458	5.328092	0.606408
33	1	0	-5.461211	3.658265	1.049677
34	5	0	-3.525932	2.087679	0.093378
35	6	0	-4.903500	1.630920	-0.688440
36	6	0	-7.076622	0.616670	-2.235999
37	6	0	-6.118519	1.239903	-0.120233
38	6	0	-4.857497	1.516315	-2.083983
39	6	0	-5.903014	1.025137	-2.859487
40	6	0	-7.189785	0.733803	-0.855912
41	6	0	-3.095407	3.071928	2.599281
42	6	0	-3.746063	0.698170	3.818429
43	6	0	-2.983057	2.956679	3.985640
44	6	0	-3.531857	2.043472	1.755282
45	6	0	-3.845917	0.861455	2.441170
46	6	0	-3.308444	1.758190	4.605332
47	9	0	-2.746514	4.286893	2.108929
48	9	0	-2.561712	3.997652	4.719022
49	9	0	-3.205423	1.625038	5.932295
50	9	0	-4.061541	-0.474156	4.388561
51	9	0	-4.278527	-0.226889	1.767100
52	9	0	-6.340469	1.384942	1.204125
53	9	0	-3.749844	1.903000	-2.752899
54	9	0	-5.785241	0.927073	-4.191937
55	9	0	-8.089490	0.119869	-2.959819
56	9	0	-8.327109	0.370416	-0.245484
57	7	0	2.955888	3.156541	-0.699227
58	1	0	7.116194	2.910851	-2.620726
59	6	0	6.040616	2.821621	-2.759549
60	6	0	3.291932	2.582018	-3.067172
61	6	0	5.537113	2.454766	-4.017302
62	6	0	5.202824	3.051217	-1.673382
63	6	0	3.807223	2.938043	-1.812334
64	6	0	4.149172	2.337659	-4.140414
65	1	0	5.625813	3.313851	-0.709211
66	1	0	3.720520	2.055973	-5.099660
67	1	0	2.219373	2.491588	-3.204530
68	1	0	2.927445	2.788923	1.932853
69	6	0	3.194644	3.802243	1.648302
70	6	0	3.980390	6.368016	0.917825
71	6	0	3.284972	4.133626	0.288918
72	6	0	3.478151	4.755589	2.622624
73	6	0	3.879345	6.054533	2.279420
74	6	0	3.682757	5.428999	-0.068530
75	1	0	3.402003	4.478149	3.671592
76	1	0	3.760880	5.693911	-1.118922
77	1	0	4.285789	7.369288	0.621525
78	6	0	6.465664	2.181951	-5.177373

79	1	0	7.135950	1.343766	-4.956749
80	1	0	7.094797	3.052027	-5.404071
81	1	0	5.905641	1.930360	-6.083724
82	6	0	4.220822	7.069943	3.344786
83	1	0	5.233854	6.911332	3.738395
84	1	0	3.532347	7.007181	4.194911
85	1	0	4.180101	8.091393	2.952202
86	7	0	3.166231	-3.615521	-0.404449
87	6	0	2.652889	-6.103490	-1.562839
88	6	0	1.895636	-3.921662	-0.828674
89	6	0	4.133297	-4.582832	-0.422663
90	6	0	3.892506	-5.824637	-1.001293
91	6	0	1.654428	-5.154638	-1.457895
92	1	0	4.688788	-6.560621	-1.003897
93	1	0	0.678088	-5.346797	-1.879763
94	1	0	2.463894	-7.052563	-2.055728
95	5	0	3.525421	-2.088223	0.089900
96	6	0	4.856668	-1.512306	-2.086429
97	6	0	7.189309	-0.732535	-0.857341
98	6	0	4.902927	-1.629658	-0.691131
99	6	0	5.902100	-1.019803	-2.861203
100	6	0	7.075861	-0.612638	-2.237156
101	6	0	6.118156	-1.239992	-0.122425
102	6	0	3.846611	-0.868744	2.440979
103	6	0	2.984804	-2.968722	3.979813
104	6	0	3.531826	-2.048695	1.751842
105	6	0	3.748498	-0.709946	3.818928
106	6	0	3.311359	-1.772334	4.602914
107	6	0	3.095711	-3.079603	2.592996
108	9	0	6.340595	-1.388011	1.201515
109	9	0	8.326814	-0.370471	-0.246428
110	9	0	8.088565	-0.114274	-2.960184
111	9	0	5.784062	-0.919167	-4.193435
112	9	0	3.748874	-1.897631	-2.755899
113	9	0	4.278557	0.221520	1.769772
114	9	0	4.065242	0.460227	4.392681
115	9	0	3.209899	-1.643349	5.930415
116	9	0	2.563759	-4.011853	4.710316
117	9	0	2.745863	-4.292920	2.099159
118	6	0	5.482131	-4.368072	0.214642
119	1	0	6.214309	-4.009253	-0.515618
120	1	0	5.838946	-5.330246	0.594094
121	1	0	5.460922	-3.661582	1.041853
122	7	0	-2.956283	-3.155196	-0.705413
123	1	0	-5.626030	-3.319271	-0.715227
124	6	0	-5.203736	-3.052712	-1.678617
125	6	0	-4.152396	-2.329553	-4.143810
126	6	0	-6.042295	-2.821991	-2.763986
127	6	0	-3.808419	-2.935715	-1.817463
128	6	0	-3.294319	-2.574914	-3.071498
129	6	0	-5.540053	-2.450219	-4.020763
130	1	0	-7.117595	-2.914592	-2.625173
131	1	0	-2.222017	-2.481703	-3.208975
132	1	0	-3.724599	-2.044239	-5.102374

133	6	0	-3.285779	-4.130183	0.284945
134	6	0	-3.888162	-6.044004	2.279636
135	6	0	-3.678697	-5.427658	-0.069588
136	6	0	-3.209182	-3.791378	1.643414
137	6	0	-3.498924	-4.740593	2.619881
138	6	0	-3.982311	-6.362780	0.918802
139	1	0	-3.755947	-5.695180	-1.119406
140	1	0	-2.955849	-2.773702	1.925032
141	1	0	-3.440253	-4.455770	3.668004
142	1	0	-4.289652	-7.364012	0.624538
143	6	0	-6.469514	-2.175307	-5.179627
144	1	0	-7.103598	-3.042583	-5.403069
145	1	0	-5.910000	-1.928578	-6.087638
146	1	0	-7.135060	-1.333144	-4.959632
147	6	0	-4.182096	-7.071063	3.347937
148	1	0	-3.257657	-7.515070	3.741317
149	1	0	-4.797559	-7.889086	2.959363
150	1	0	-4.710885	-6.625321	4.197887

Table S11. Coordinates for Optimized Structure of **6-Me'** in the ground state (S_0).
E(RB3LYP) = -3051.09504386 Hartree.

```
# opt freq rb3lyp/6-31g(d) geom=connectivity
```

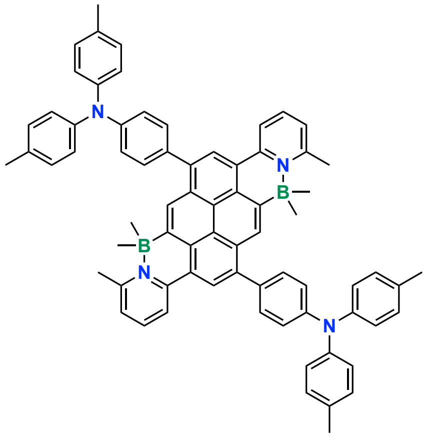

Center Number	Atomic Number	Atomic Type	Coordinates (Angstroms) X	Y	Z
1	1	0	3.258717	3.221065	0.037166
2	6	0	2.474734	2.476121	-0.038017
3	6	0	0.500950	0.510753	-0.041993
4	6	0	1.126116	2.878061	-0.001944
5	6	0	2.873421	1.143234	-0.083124
6	6	0	1.873654	0.129241	-0.150549
7	6	0	0.122367	1.884671	0.078822
8	6	0	-1.870184	-0.121654	0.079783
9	6	0	-1.241897	2.263534	0.301564
10	6	0	-0.499947	-0.504279	-0.048703

11	6	0	-2.179413	1.256148	0.292598
12	1	0	-3.215039	1.499367	0.499981
13	6	0	-2.473668	-2.468456	-0.031041
14	6	0	-0.122744	-1.879054	-0.173913
15	6	0	-1.127533	-2.873343	-0.098702
16	6	0	-2.870109	-1.135119	0.033415
17	1	0	-3.262393	-3.208246	-0.101095
18	6	0	2.183035	-1.247564	-0.358188
19	6	0	1.242837	-2.253168	-0.383853
20	1	0	3.223526	-1.501222	-0.530613
21	1	0	2.306048	7.210353	-1.085019
22	6	0	1.562285	6.544320	-0.656919
23	7	0	-0.402815	4.810165	0.345425
24	6	0	0.386945	7.040725	-0.108243
25	6	0	1.765383	5.179739	-0.637675
26	6	0	0.804251	4.311588	-0.082747
27	6	0	-0.585103	6.165886	0.372828
28	1	0	0.195294	8.107141	-0.064772
29	1	0	2.662056	4.760785	-1.071780
30	6	0	-1.840529	6.793716	0.924926
31	1	0	-2.626121	6.839518	0.165597
32	1	0	-1.612599	7.818184	1.232871
33	1	0	-2.238486	6.252794	1.779613
34	5	0	-1.652750	3.757398	0.780378
35	6	0	4.328546	0.840643	-0.049893
36	6	0	7.129469	0.363099	0.067534
37	6	0	4.877647	-0.068347	0.872122
38	6	0	5.221818	1.505335	-0.907225
39	6	0	6.591987	1.269078	-0.861707
40	6	0	6.247263	-0.298485	0.939051
41	1	0	4.221760	-0.580854	1.569613
42	1	0	4.829686	2.195182	-1.650230
43	1	0	7.253048	1.779069	-1.554992
44	1	0	6.642770	-0.990045	1.675872
45	7	0	8.524581	0.120487	0.123627
46	1	0	12.372424	1.895720	-1.657566
47	6	0	11.492742	2.057730	-1.038160
48	6	0	9.237958	2.434529	0.539891
49	6	0	11.289062	3.310954	-0.445865
50	6	0	10.586581	1.013968	-0.866910
51	6	0	9.444730	1.187582	-0.070214
52	6	0	10.143149	3.474471	0.343887
53	1	0	10.762827	0.055608	-1.345185
54	1	0	9.962120	4.430226	0.831455
55	1	0	8.367179	2.581718	1.171151
56	1	0	7.615688	-2.155195	-0.965271
57	6	0	8.439325	-2.303488	-0.273990
58	6	0	10.570263	-2.695601	1.464841
59	6	0	9.013041	-1.194099	0.366338
60	6	0	8.921615	-3.586173	-0.027996
61	6	0	9.996110	-3.811718	0.843120
62	6	0	10.086010	-1.408179	1.244032
63	1	0	10.541761	-0.561185	1.747277
64	1	0	11.406002	-2.834680	2.147399

65	6	0	12.255009	4.450800	-0.670083
66	1	0	11.976102	5.047986	-1.549180
67	1	0	13.274236	4.086203	-0.837952
68	1	0	12.278153	5.131524	0.188040
69	6	0	10.497863	-5.209622	1.119856
70	1	0	10.408116	-5.850594	0.235680
71	1	0	9.925894	-5.689794	1.925641
72	1	0	11.549139	-5.205040	1.427162
73	7	0	0.377919	-4.789376	-0.590698
74	6	0	-1.609409	-6.577834	0.249337
75	6	0	-0.818247	-4.312647	-0.113433
76	6	0	0.523953	-6.137542	-0.782271
77	6	0	-0.455922	-7.038792	-0.375507
78	6	0	-1.789550	-5.215602	0.366082
79	1	0	-0.292404	-8.098179	-0.537127
80	1	0	-2.674967	-4.827772	0.849393
81	1	0	-2.355892	-7.270502	0.627504
82	5	0	1.721866	-3.767310	-0.694694
83	6	0	1.727175	-6.703765	-1.491407
84	1	0	1.728255	-6.399212	-2.541857
85	1	0	1.684879	-7.795233	-1.453039
86	1	0	2.670405	-6.376496	-1.058649
87	6	0	-4.326107	-0.834145	0.036645
88	6	0	-7.129687	-0.360986	-0.009622
89	6	0	-5.195989	-1.499566	0.917066
90	6	0	-4.899919	0.072954	-0.871991
91	6	0	-6.271156	0.300924	-0.904256
92	6	0	-6.567258	-1.265137	0.906483
93	1	0	-4.783695	-2.188340	1.650070
94	1	0	-4.262663	0.585526	-1.586531
95	1	0	-6.686486	0.991186	-1.631321
96	1	0	-7.209591	-1.775420	1.616954
97	7	0	-8.526292	-0.120966	-0.030435
98	1	0	-7.583588	2.161396	1.018549
99	6	0	-8.432929	2.304585	0.358039
100	6	0	-10.612418	2.688218	-1.321758
101	6	0	-8.922789	3.586209	0.119832
102	6	0	-9.024853	1.192198	-0.259449
103	6	0	-10.121494	1.402321	-1.108997
104	6	0	-10.025455	3.806875	-0.716183
105	1	0	-8.441973	4.432224	0.606231
106	1	0	-10.584281	0.553825	-1.603200
107	1	0	-11.462043	2.824981	-1.987537
108	6	0	-9.439654	-1.189687	0.185727
109	6	0	-11.267395	-3.317418	0.613673
110	6	0	-10.557593	-1.019572	1.016855
111	6	0	-9.245607	-2.436805	-0.427438
112	6	0	-10.141261	-3.479826	-0.203818
113	6	0	-11.454502	-2.066017	1.215809
114	1	0	-10.717132	-0.064292	1.507018
115	1	0	-8.387616	-2.585124	-1.075706
116	1	0	-9.966466	-4.437993	-0.688802
117	1	0	-12.311638	-1.909287	1.867436
118	6	0	-10.576167	5.196472	-0.935894

119	1	0	-11.070524	5.282227	-1.909698
120	1	0	-9.785318	5.953268	-0.891944
121	1	0	-11.319333	5.460526	-0.170921
122	6	0	-12.257791	-4.440024	0.817250
123	1	0	-13.066426	-4.402999	0.074363
124	1	0	-12.726317	-4.387161	1.806217
125	1	0	-11.778196	-5.420425	0.723980
126	1	0	8.460277	-4.429216	-0.538014
127	6	0	-3.034448	4.233467	0.031076
128	1	0	-2.858579	4.698407	-0.950688
129	1	0	-3.664572	4.919571	0.608421
130	1	0	-3.663979	3.359712	-0.173493
131	6	0	-1.714220	3.723744	2.419992
132	1	0	-2.489879	3.010196	2.730106
133	1	0	-1.959242	4.676870	2.909032
134	1	0	-0.773385	3.372835	2.866639
135	6	0	2.369163	-3.809329	-2.201663
136	1	0	1.606272	-3.843921	-2.993857
137	1	0	3.074410	-4.626486	-2.391918
138	1	0	2.931680	-2.883085	-2.373891
139	6	0	2.718733	-4.235455	0.524841
140	1	0	3.142961	-5.245376	0.453090
141	1	0	2.229619	-4.166591	1.506877
142	1	0	3.576731	-3.550231	0.559765

Table S12. Coordinates for Optimized Structure of **6-Pf'** in the ground state (S_0).
E(RB3LYP) = -5802.62334142 Hartree.

```
# opt freq rb3lyp/6-31g(d) geom=connectivity
```

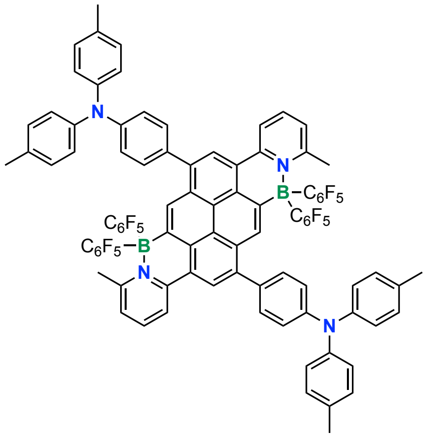

Center Number	Atomic Number	Atomic Type	Coordinates (Angstroms) X	Y	Z
1	1	0	-2.525964	-3.733842	-0.808691
2	6	0	-1.917663	-2.849453	-0.654066
3	6	0	-0.390368	-0.584111	-0.116115
4	6	0	-0.517102	-2.967056	-0.627409

5	6	0	-2.583767	-1.651879	-0.397858
6	6	0	-1.811209	-0.470535	-0.197799
7	6	0	0.257985	-1.846404	-0.258698
8	6	0	1.810997	0.470279	0.198642
9	6	0	1.666849	-1.983296	-0.040276
10	6	0	0.390136	0.583836	0.117182
11	6	0	2.398364	-0.833468	0.140423
12	1	0	3.465503	-0.901435	0.304306
13	6	0	1.917480	2.849274	0.654493
14	6	0	-0.258232	1.846117	0.259861
15	6	0	0.516913	2.966800	0.628383
16	6	0	2.583569	1.651705	0.398163
17	1	0	2.525780	3.733731	0.808728
18	6	0	-2.398600	0.833173	-0.139283
19	6	0	-1.667125	1.982984	0.041680
20	1	0	-3.465743	0.901138	-0.303091
21	1	0	-0.636097	-7.008144	-2.862758
22	6	0	-0.092680	-6.343458	-2.197675
23	7	0	1.363784	-4.581062	-0.590033
24	6	0	1.110688	-6.734198	-1.623322
25	6	0	-0.596626	-5.097379	-1.880137
26	6	0	0.109523	-4.232596	-1.026770
27	6	0	1.823605	-5.848825	-0.821146
28	1	0	1.515907	-7.726236	-1.789110
29	1	0	-1.535543	-4.763816	-2.299855
30	6	0	3.092483	-6.362221	-0.190978
31	1	0	3.948160	-6.240633	-0.862728
32	1	0	2.972247	-7.433459	-0.003517
33	1	0	3.333207	-5.876364	0.751885
34	5	0	2.307958	-3.463022	0.172767
35	6	0	3.781382	-3.509774	-0.574377
36	6	0	6.232975	-3.396538	-2.046535
37	6	0	5.015678	-3.862548	-0.020876
38	6	0	3.850463	-3.117312	-1.917881
39	6	0	5.030663	-3.050122	-2.655060
40	6	0	6.220626	-3.811978	-0.720588
41	6	0	1.363887	-4.600644	2.456978
42	6	0	3.034822	-3.132666	4.070904
43	6	0	1.274303	-4.735032	3.843009
44	6	0	2.270300	-3.755837	1.805643
45	6	0	3.085582	-3.029522	2.684773
46	6	0	2.116023	-3.994397	4.661093
47	9	0	0.492086	-5.370692	1.760330
48	9	0	0.379956	-5.574847	4.385584
49	9	0	2.047701	-4.108326	5.992418
50	9	0	3.858592	-2.406837	4.840088
51	9	0	4.002487	-2.163365	2.203823
52	9	0	5.101251	-4.352569	1.233895
53	9	0	2.720367	-2.795643	-2.582649
54	9	0	5.016933	-2.664741	-3.938884
55	9	0	7.380742	-3.349243	-2.733544
56	9	0	7.368486	-4.177277	-0.123004
57	6	0	-4.066658	-1.664550	-0.337049
58	6	0	-6.902614	-1.808512	-0.164693

59	6	0	-4.760774	-1.124368	0.759932
60	6	0	-4.830929	-2.281270	-1.342330
61	6	0	-6.217844	-2.345113	-1.269958
62	6	0	-6.144732	-1.201640	0.853788
63	1	0	-4.201976	-0.668711	1.572482
64	1	0	-4.330504	-2.684724	-2.219056
65	1	0	-6.780226	-2.800940	-2.077996
66	1	0	-6.647872	-0.798167	1.725877
67	7	0	-8.310900	-1.865961	-0.079537
68	1	0	-11.863762	-3.699385	-2.340661
69	6	0	-10.940002	-3.876427	-1.793922
70	6	0	-8.580581	-4.291729	-0.387216
71	6	0	-10.492023	-5.190659	-1.605952
72	6	0	-10.222917	-2.785326	-1.309530
73	6	0	-9.030652	-2.979082	-0.596059
74	6	0	-9.298967	-5.371995	-0.893968
75	1	0	-10.586588	-1.776233	-1.476056
76	1	0	-8.931483	-6.380359	-0.714993
77	1	0	-7.669553	-4.460987	0.178329
78	1	0	-8.002487	0.757616	-0.506334
79	6	0	-8.752363	0.528941	0.244982
80	6	0	-10.687077	-0.048542	2.153246
81	6	0	-9.025897	-0.810553	0.561632
82	6	0	-9.424393	1.557874	0.896607
83	6	0	-10.404606	1.291163	1.863969
84	6	0	-10.005619	-1.089352	1.522388
85	1	0	-9.179899	2.586467	0.648647
86	1	0	-10.226243	-2.121329	1.777714
87	1	0	-11.439494	-0.286932	2.901807
88	6	0	-11.254062	-6.366761	-2.170315
89	1	0	-10.931773	-6.600952	-3.194240
90	1	0	-12.330351	-6.166637	-2.208918
91	1	0	-11.100803	-7.269580	-1.569048
92	6	0	-11.121131	2.421827	2.563752
93	1	0	-11.779090	2.969346	1.876178
94	1	0	-10.407053	3.147268	2.971442
95	1	0	-11.738605	2.054943	3.389985
96	7	0	-1.364146	4.580656	0.591952
97	6	0	0.092902	6.343060	2.199057
98	6	0	-0.109637	4.232282	1.028050
99	6	0	-1.824057	5.848303	0.823514
100	6	0	-1.110842	6.733684	1.625417
101	6	0	0.596843	5.097078	1.881130
102	1	0	-1.516139	7.725627	1.791580
103	1	0	1.536011	4.763596	2.300349
104	1	0	0.636588	7.007747	2.863920
105	5	0	-2.308504	3.462700	-0.170675
106	6	0	-1.365766	4.601136	-2.455054
107	6	0	-3.037365	3.133517	-4.068597
108	6	0	-2.271754	3.756023	-1.803508
109	6	0	-1.276909	4.735989	-3.841085
110	6	0	-2.118969	3.995535	-4.658983
111	6	0	-3.087400	3.029910	-2.682472
112	6	0	-5.016274	3.861320	0.024647

113	6	0	-5.029360	3.048342	2.658678
114	6	0	-3.781516	3.508916	0.577340
115	6	0	-6.220808	3.809975	0.725023
116	6	0	-6.232184	3.394316	2.050910
117	6	0	-3.849629	3.116193	1.920821
118	9	0	-4.003964	2.163471	-2.201383
119	9	0	-3.861452	2.407840	-4.837589
120	9	0	-2.051341	4.109908	-5.990307
121	9	0	-0.382926	5.576074	-4.383844
122	9	0	-0.493667	5.371051	-1.758632
123	9	0	-5.102777	4.351643	-1.229945
124	9	0	-7.369195	4.174782	0.128155
125	9	0	-7.379523	3.346364	2.738591
126	9	0	-5.014714	2.662774	3.942434
127	9	0	-2.718998	2.794890	2.584852
128	6	0	-3.093416	6.361546	0.194185
129	1	0	-2.973606	7.432883	0.007028
130	1	0	-3.948705	6.239457	0.866337
131	1	0	-3.334495	5.875930	-0.748715
132	6	0	4.066408	1.664626	0.336352
133	6	0	6.902063	1.809981	0.161390
134	6	0	4.831233	2.282465	1.340534
135	6	0	4.759836	1.123868	-0.760801
136	6	0	6.143670	1.201791	-0.855896
137	6	0	6.218058	2.347011	1.266878
138	1	0	4.331370	2.686327	2.217390
139	1	0	4.200552	0.667335	-1.572520
140	1	0	6.646355	0.797897	-1.728067
141	1	0	6.780941	2.803803	2.074037
142	7	0	8.310284	1.868192	0.074694
143	1	0	7.999250	-0.754216	0.507653
144	6	0	8.750644	-0.527896	-0.242851
145	6	0	10.689348	0.043450	-2.148890
146	6	0	9.423243	-1.558899	-0.890566
147	6	0	9.025580	0.810655	-0.562416
148	6	0	10.007387	1.086312	-1.521977
149	6	0	10.405439	-1.295322	-1.856787
150	1	0	9.177684	-2.586704	-0.640392
151	1	0	10.229138	2.117478	-1.779569
152	1	0	11.443340	0.279458	-2.896624
153	6	0	9.030542	2.982489	0.587989
154	6	0	10.489682	5.194937	1.599070
155	6	0	10.220071	2.789244	1.306665
156	6	0	8.579240	4.294510	0.379769
157	6	0	9.295426	5.375278	0.889275
158	6	0	10.934710	3.880452	1.793708
159	1	0	10.578363	1.779791	1.482462
160	1	0	7.664424	4.463160	-0.179796
161	1	0	8.923054	6.382910	0.716691
162	1	0	11.852470	3.703788	2.350685
163	6	0	11.122370	-2.428267	-2.552451
164	1	0	11.742067	-2.063855	-3.378115
165	1	0	10.408431	-3.153849	-2.960145
166	1	0	11.778206	-2.974965	-1.862205

167	6	0	11.283539	6.373681	2.111753
168	1	0	12.037447	6.698743	1.381704
169	1	0	11.816342	6.126660	3.036655
170	1	0	10.637833	7.234835	2.314830

Table S13. Coordinates for Optimized Structure of **2** in the ground state (S_0).
E(RB3LYP) = -1188.59648238 Hartree.

```
# opt freq rb3lyp/6-31g(d) geom=connectivity
```

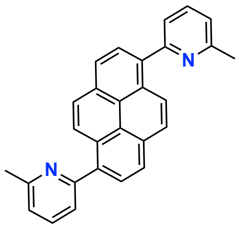

Center Number	Atomic Number	Atomic Type	Coordinates (Angstroms) X	Y	Z
1	1	0	-3.477819	3.020347	-0.183003
2	6	0	-2.662699	2.302539	-0.196819
3	6	0	-2.967077	0.932443	-0.169132
4	6	0	-0.279661	1.863432	-0.184982
5	6	0	-1.903211	-0.010383	-0.157479
6	6	0	-1.354563	2.763488	-0.200059
7	6	0	-0.548168	0.461777	-0.167957
8	6	0	-2.121715	-1.431821	-0.177950
9	6	0	-1.081476	-2.308643	-0.192580
10	1	0	-1.271376	-3.379383	-0.210892
11	6	0	0.279661	-1.863435	-0.184983
12	6	0	1.354563	-2.763491	-0.200057
13	6	0	0.548168	-0.461780	-0.167956
14	6	0	2.662699	-2.302542	-0.196813
15	1	0	3.477819	-3.020350	-0.182996
16	6	0	2.967078	-0.932445	-0.169126
17	6	0	1.903212	0.010381	-0.157474
18	6	0	1.081476	2.308640	-0.192573
19	1	0	1.271376	3.379380	-0.210883
20	6	0	2.121715	1.431819	-0.177941
21	6	0	-4.408114	0.549175	-0.161180
22	6	0	-7.075780	-0.105660	-0.048280
23	7	0	-4.811538	-0.411147	0.689961
24	6	0	-5.314997	1.213921	-1.006997
25	6	0	-6.662985	0.879932	-0.942828
26	6	0	-6.112873	-0.735629	0.748528
27	1	0	-4.959313	1.960558	-1.709201
28	1	0	-7.383493	1.376073	-1.588124
29	6	0	4.408114	-0.549178	-0.161176
30	6	0	7.075786	0.105635	-0.048252
31	6	0	5.315002	-1.213944	-1.006971
32	7	0	4.811537	0.411153	0.689956
33	6	0	6.112873	0.735629	0.748531
34	6	0	6.662993	-0.879967	-0.942789
35	1	0	4.959322	-1.960594	-1.709163
36	1	0	7.383505	-1.376131	-1.588062
37	1	0	3.139284	1.797610	-0.162957
38	1	0	-3.139284	-1.797613	-0.162971

39	1	0	-8.121348	-0.389120	0.028532
40	1	0	8.121356	0.389082	0.028575
41	6	0	6.482945	1.820615	1.730573
42	1	0	6.163605	1.545095	2.741679
43	1	0	5.971438	2.756914	1.478921
44	6	0	-6.482958	-1.820545	1.730642
45	1	0	-6.164355	-1.544592	2.741868
46	1	0	-5.970816	-2.756639	1.479542
47	1	0	7.561092	2.006743	1.742191
48	1	0	-7.561020	-2.007206	1.741702
49	1	0	1.153461	-3.831957	-0.204847
50	1	0	-1.153461	3.831954	-0.204848

Table S14. Coordinates for Optimized Structure of **L1** in the ground state (S_0).
E(RB3LYP) = -2380.75932514 Hartree.

```
# opt freq rb3lyp/6-31g(d) geom=connectivity
```

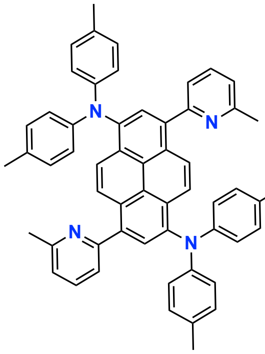

Center Number	Atomic Number	Atomic Type	Coordinates (Angstroms) X	Y	Z
1	1	0	-4.143515	-2.007847	-0.146025
2	6	0	-3.161390	-1.546833	-0.169964
3	6	0	-2.024819	-2.359481	-0.181174
4	6	0	-1.828095	0.485169	-0.157515
5	6	0	-0.740059	-1.751167	-0.160279
6	6	0	-3.090242	-0.154751	-0.146875
7	6	0	-0.644673	-0.319664	-0.145667
8	6	0	0.474804	-2.510606	-0.210551
9	6	0	1.695204	-1.908418	-0.206256
10	1	0	2.594256	-2.511630	-0.249436
11	6	0	1.826873	-0.484835	-0.157011
12	6	0	3.088987	0.155089	-0.147547
13	6	0	0.643468	0.320033	-0.146234
14	6	0	3.160216	1.547107	-0.172498
15	1	0	4.142358	2.008127	-0.149402
16	6	0	2.023675	2.359790	-0.184824
17	6	0	0.738903	1.751511	-0.162996
18	6	0	-1.696374	1.908676	-0.209017
19	1	0	-2.595380	2.511882	-0.253037
20	6	0	-0.475975	2.510858	-0.214359
21	7	0	-4.301785	0.602197	-0.120356
22	7	0	4.300506	-0.601975	-0.120174
23	6	0	-2.243239	-3.834705	-0.206406
24	6	0	-2.695467	-6.544751	-0.160495
25	7	0	-1.510073	-4.606493	0.615323
26	6	0	-3.224787	-4.380591	-1.053833
27	6	0	-3.450205	-5.752096	-1.023254
28	6	0	-1.725675	-5.931252	0.640815
29	1	0	-3.781267	-3.738833	-1.728876
30	1	0	-4.200523	-6.200059	-1.669779
31	6	0	2.242163	3.834977	-0.212270
32	6	0	2.694422	6.545075	-0.170364
33	6	0	3.224133	4.379517	-1.060078
34	7	0	1.508624	4.608057	0.607913

35	6	0	1.724239	5.932852	0.631440
36	6	0	3.449570	5.751061	-1.031515
37	1	0	3.781018	3.736758	-1.733829
38	1	0	4.200254	6.197963	-1.678344
39	6	0	5.288171	-0.317234	-1.102536
40	6	0	7.218491	0.281352	-3.097929
41	6	0	6.642259	-0.160298	-0.768150
42	6	0	4.911149	-0.163879	-2.446806
43	6	0	5.861304	0.138811	-3.417943
44	6	0	7.583767	0.124854	-1.755089
45	1	0	6.954629	-0.258121	0.266400
46	1	0	3.867950	-0.282116	-2.723324
47	1	0	5.540344	0.253603	-4.451291
48	1	0	8.626485	0.244379	-1.467899
49	6	0	4.613751	-1.388439	1.021549
50	6	0	5.203529	-2.998882	3.285585
51	6	0	5.396792	-2.549264	0.896020
52	6	0	4.130185	-1.045926	2.292094
53	6	0	4.418391	-1.846327	3.396679
54	6	0	5.690378	-3.326507	2.011869
55	1	0	5.776067	-2.835861	-0.079888
56	1	0	3.528464	-0.151319	2.414622
57	1	0	4.029622	-1.556342	4.370517
58	1	0	6.302217	-4.217553	1.885797
59	6	0	-5.289327	0.315153	-1.102130
60	6	0	-7.219198	-0.279769	-3.099148
61	6	0	-4.911739	0.162680	-2.446716
62	6	0	-6.644223	0.164806	-0.769492
63	6	0	-7.585963	-0.116330	-1.757874
64	6	0	-5.861767	-0.135820	-3.418807
65	1	0	-3.869315	0.287304	-2.723359
66	1	0	-6.959196	0.273037	0.263204
67	1	0	-8.630470	-0.223942	-1.472722
68	1	0	-5.541581	-0.242929	-4.453281
69	6	0	-4.613972	1.391815	1.019551
70	6	0	-5.200911	3.008342	3.279924
71	6	0	-4.131719	1.050835	2.290973
72	6	0	-5.394220	2.554202	0.891217
73	6	0	-5.686453	3.334468	2.005307
74	6	0	-4.418511	1.854208	3.393784
75	1	0	-3.532092	0.155095	2.415554
76	1	0	-5.772389	2.839635	-0.085471
77	1	0	-6.296120	4.226698	1.877113
78	1	0	-4.030821	1.565378	4.368394
79	6	0	-5.490704	3.885626	4.475294
80	1	0	-4.820168	4.755236	4.509119
81	1	0	-5.358768	3.337675	5.414304
82	1	0	-6.516361	4.271611	4.452839
83	6	0	-8.240596	-0.621354	-4.158789
84	1	0	-8.282828	-1.703396	-4.345042
85	1	0	-8.004160	-0.140176	-5.114621
86	1	0	-9.246260	-0.304242	-3.862928
87	6	0	8.249790	0.566281	-4.164598
88	1	0	8.607582	-0.358974	-4.636918

89	1	0	9.125647	1.076267	-3.748896
90	1	0	7.840274	1.196606	-4.961859
91	6	0	5.494796	-3.872995	4.482916
92	1	0	6.521033	-4.257468	4.461209
93	1	0	4.825580	-4.743541	4.518825
94	1	0	5.362141	-3.323108	5.420694
95	1	0	-0.408948	3.589784	-0.242172
96	1	0	0.407779	-3.589577	-0.236699
97	1	0	-2.847515	-7.618961	-0.110818
98	1	0	2.846470	7.619359	-0.122289
99	6	0	0.860737	6.729175	1.579454
100	1	0	0.974529	6.356655	2.603441
101	1	0	-0.197992	6.620775	1.316999
102	6	0	-0.862692	-6.726061	1.590569
103	1	0	-0.977726	-6.352485	2.614037
104	1	0	0.196232	-6.617341	1.329065
105	1	0	1.115294	7.793215	1.563389
106	1	0	-1.116661	-7.790255	1.575439

Table S15. Coordinates for Optimized Structure of **L2** in the ground state (S_0).
E(RB3LYP) = -2842.87183002 Hartree.

```
# opt freq rb3lyp/6-31g(d) geom=connectivity
```

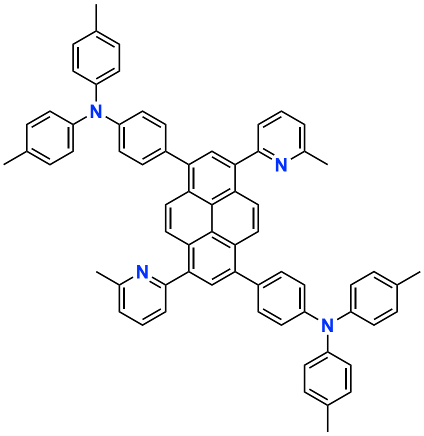

Center Number	Atomic Number	Atomic Type	Coordinates (Angstroms) X	Y	Z
1	1	0	-3.368392	3.136765	-0.107871
2	6	0	-2.571197	2.401140	-0.055852
3	6	0	-0.525666	0.492693	-0.023652
4	6	0	-1.247669	2.843917	-0.134008
5	6	0	-2.926022	1.051389	0.002810
6	6	0	-1.895592	0.076883	0.043746
7	6	0	-0.200402	1.884970	-0.137223
8	6	0	1.893377	-0.076513	-0.043802
9	6	0	1.181918	2.253316	-0.224745
10	6	0	0.523450	-0.492325	0.023610
11	6	0	2.172128	1.321457	-0.187935
12	1	0	3.204130	1.638096	-0.284511
13	6	0	2.568967	-2.400776	0.055808
14	6	0	0.198182	-1.884602	0.137199
15	6	0	1.245446	-2.843555	0.133989
16	6	0	2.923793	-1.051027	-0.002890
17	1	0	3.366162	-3.136400	0.107816
18	6	0	-2.174345	-1.321083	0.187907
19	6	0	-1.184138	-2.252943	0.224741
20	1	0	-3.206348	-1.637713	0.284503
21	1	0	-2.115669	7.239256	1.142146
22	6	0	-1.571842	6.552044	0.498928
23	7	0	-0.159302	4.788811	-1.130764
24	6	0	-0.682145	7.034021	-0.459309
25	6	0	-1.748199	5.179192	0.628447
26	6	0	-1.025927	4.317054	-0.216850
27	6	0	0.013479	6.114529	-1.252578
28	1	0	-0.522084	8.100463	-0.588145
29	1	0	-2.417802	4.771565	1.378450
30	6	0	1.003220	6.561248	-2.301175
31	1	0	2.005511	6.182508	-2.069071

32	1	0	1.053413	7.651923	-2.373125
33	1	0	0.728936	6.156710	-3.281547
34	6	0	-4.373090	0.706132	0.004606
35	6	0	-7.163144	0.158207	-0.032713
36	6	0	-4.933216	-0.172423	-0.939408
37	6	0	-5.249958	1.308253	0.922779
38	6	0	-6.614130	1.036035	0.916800
39	6	0	-6.298297	-0.437809	-0.966394
40	1	0	-4.292354	-0.628562	-1.688627
41	1	0	-4.848003	1.979834	1.676954
42	1	0	-7.261889	1.498547	1.654496
43	1	0	-6.704503	-1.102947	-1.721576
44	7	0	-8.553026	-0.117523	-0.050534
45	1	0	-12.379427	1.483893	1.930483
46	6	0	-11.525246	1.693933	1.290196
47	6	0	-9.334514	2.193786	-0.343179
48	6	0	-11.371168	2.975850	0.746305
49	6	0	-10.600911	0.681417	1.044485
50	6	0	-9.491311	0.917545	0.219032
51	6	0	-10.256806	3.201528	-0.072731
52	1	0	-10.738132	-0.300454	1.486552
53	1	0	-10.114597	4.181465	-0.523649
54	1	0	-8.489096	2.389736	-0.995303
55	1	0	-7.559063	-2.415713	0.905280
56	6	0	-8.399356	-2.553797	0.232056
57	6	0	-10.574765	-2.919352	-1.456329
58	6	0	-9.018329	-1.430906	-0.338834
59	6	0	-8.859588	-3.835691	-0.057143
60	6	0	-9.955646	-4.048137	-0.904285
61	6	0	-10.113696	-1.631739	-1.191815
62	1	0	-10.605046	-0.774328	-1.640963
63	1	0	-11.428580	-3.047924	-2.118233
64	6	0	-12.355401	4.080734	1.051318
65	1	0	-12.060441	4.646793	1.945585
66	1	0	-13.359448	3.684103	1.237005
67	1	0	-12.423608	4.796395	0.224648
68	6	0	-10.434370	-5.443820	-1.228863
69	1	0	-10.285738	-6.125627	-0.384127
70	1	0	-9.890386	-5.866739	-2.084596
71	1	0	-11.499057	-5.453175	-1.485748
72	7	0	0.157004	-4.788436	1.130688
73	6	0	1.569715	-6.551702	-0.498814
74	6	0	1.023698	-4.316691	0.216820
75	6	0	-0.015792	-6.114143	1.252513
76	6	0	0.679956	-7.033659	0.459368
77	6	0	1.746042	-5.178844	-0.628388
78	1	0	0.519946	-8.100101	0.588273
79	1	0	2.415732	-4.771248	-1.378329
80	1	0	2.113667	-7.238925	-1.141915
81	6	0	-1.005998	-6.560849	2.300679
82	1	0	-0.735213	-6.151829	3.280123
83	1	0	-1.052443	-7.651448	2.376223
84	1	0	-2.009260	-6.186698	2.065185
85	6	0	4.370890	-0.705848	-0.004756

86	6	0	7.160891	-0.158428	0.032566
87	6	0	5.247619	-1.308149	-0.922956
88	6	0	4.931104	0.172695	0.939228
89	6	0	6.296250	0.437849	0.966187
90	6	0	6.611847	-1.036154	-0.916942
91	1	0	4.845534	-1.979695	-1.677088
92	1	0	4.290297	0.628962	1.688414
93	1	0	6.702653	1.103027	1.721255
94	1	0	7.259651	-1.498842	-1.654516
95	7	0	8.551009	0.116890	0.050454
96	1	0	7.549653	2.414448	-0.901943
97	6	0	8.395986	2.552807	-0.236412
98	6	0	10.570492	2.921691	1.452619
99	6	0	8.854299	3.835632	0.053182
100	6	0	9.017478	1.430987	0.333113
101	6	0	10.111563	1.633708	1.187872
102	6	0	9.953681	4.049428	0.895208
103	1	0	8.352244	4.688976	-0.397891
104	1	0	10.597464	0.777415	1.645008
105	1	0	11.418680	3.052255	2.121430
106	6	0	9.489684	-0.919222	-0.213357
107	6	0	11.366996	-2.980811	-0.737140
108	6	0	10.598507	-0.686659	-1.041396
109	6	0	9.329551	-2.194975	0.348333
110	6	0	10.249489	-3.205155	0.077566
111	6	0	11.520095	-1.701230	-1.287356
112	1	0	10.731768	0.291752	-1.492267
113	1	0	8.478834	-2.391886	0.993213
114	1	0	10.100547	-4.186770	0.522494
115	1	0	12.369711	-1.496274	-1.935415
116	6	0	10.470868	5.441963	1.170638
117	1	0	10.952728	5.503568	2.152505
118	1	0	9.664059	6.182517	1.144526
119	1	0	11.216354	5.749239	0.424332
120	6	0	12.383429	-4.069195	-0.991711
121	1	0	13.192391	-4.045954	-0.248642
122	1	0	12.848444	-3.961018	-1.977800
123	1	0	11.927489	-5.063906	-0.941770
124	1	0	-8.364300	-4.689545	0.400536
125	1	0	-1.431177	-3.297159	0.358625
126	1	0	1.428957	3.297539	-0.358573

----

11. References

[S1] Vanga, M.; Lalancette, R. A.; Jäkle, F. Controlling the Optoelectronic Properties of Pyrene by Regioselective Lewis Base-Directed Electrophilic Aromatic Borylation. *Chem. Eur. J.* **2019**, *25*, 10133.

[S2] Chen, M.; Nie, H.; Song, B.; Li, L.; Sun, J. Z.; Qin, A.; Tang, B. Z. Triphenylamine-functionalized tetraphenylpyrazine: facile preparation and multifaceted functionalities. *J. Mater. Chem. C* **2016**, *4*, 2901.

[S3] Rigaku Oxford Diffraction, (2021), CrysAlisPro Software system, Rigaku Corporation, Oxford, UK.

[S4] (a) Sheldrick, G. Crystal structure refinement with SHELXL. *Acta Cryst. C* **2015**, *71*, 3-8. (b) Dolomanov, O. V.; Bourhis, L. J.; Gildea, R. J.; Howard, J. A. K.; Puschmann, H. OLEX2: a complete structure solution, refinement and analysis program. *J. Appl. Cryst.* **2009**, *42*, 339-341.

[S5] Gaussian 16, Revision A.03, M. J. Frisch, G. W. Trucks, H. B. Schlegel, G. E. Scuseria, M. A. Robb, J. R. Cheeseman, G. Scalmani, V. Barone, G. A. Petersson, H. Nakatsuji, X. Li, M. Caricato, A. V. Marenich, J. Bloino, B. G. Janesko, R. Gomperts, B. Mennucci, H. P. Hratchian, J. V. Ortiz, A. F. Izmaylov, J. L. Sonnenberg, D. Williams-Young, F. Ding, F. Lipparini, F. Egidi, J. Goings, B. Peng, A. Petrone, T. Henderson, D. Ranasinghe, V. G. Zakrzewski, J. Gao, N. Rega, G. Zheng, W. Liang, M. Hada, M. Ehara, K. Toyota, R. Fukuda, J. Hasegawa, M. Ishida, T. Nakajima, Y. Honda, O. Kitao, H. Nakai, T. Vreven, K. Throssell, J. A. Montgomery, Jr., J. E. Peralta, F. Ogliaro, M. J. Bearpark, J. J. Heyd, E. N. Brothers, K. N. Kudin, V. N. Staroverov, T. A. Keith, R. Kobayashi, J. Normand, K. Raghavachari, A. P. Rendell, J. C. Burant, S. S. Iyengar, J. Tomasi, M. Cossi, J. M. Millam, M. Klene, C. Adamo, R. Cammi, J. W. Ochterski, R. L. Martin, K. Morokuma, O. Farkas, J. B. Foresman, and D. J. Fox, Gaussian, Inc., Wallingford CT, 2016.

[S6] Nepomnyashchii, A. B.; Bröring, M.; Ahrens, J.; Bard, A. J. Synthesis, Photophysical, Electrochemical, and Electrogenerated Chemiluminescence Studies. Multiple Sequential Electron Transfers in BODIPY Monomers, Dimers, Trimers, and Polymer. *J. Am. Chem. Soc.* **2011**, *133*, 8633-8645.

[S7] Hesari, M.; Ding, Z. Electrogenerated Chemiluminescence: Light Years Ahead. *J. Electrochem. Soc.* **2015**, *163*, H3116.

[S8] Brilmyer, G. H.; Bard, A. J. Electrogenerated Chemiluminescence: XXXVI. The Production of Steady Direct Current ECL in Thin Layer and Flow Cells. *J. Electrochem. Soc.* **1980**, *127*, 104.

[S9] Hesari, M.; Ding, Z. Spooling electrochemiluminescence spectroscopy: development, applications and beyond. *Nat. Protoc.* **2021**, *16*, 2109-2130.

[S10] A. Ryer, Light Measurement Handbook, International Light, **1997**.

[S11] Adsetts, J. R.; Chu, K.; Hesari, M.; Whitworth, Z.; Qin, X.; Zhan, Z.; Ding, Z. Absolute Electrochemiluminescence Quantum Efficiency of Au Nanoclusters by Means of a Spectroscopy Charge-Coupled Device Camera. *J. Phys. Chem. C* **2022**, *126*, 20155-20162.

[S12] Adsetts, J. R.; Chu, K.; Hesari, M.; Ma, J.; Ding, Z. Absolute Electrochemiluminescence Efficiency Quantification Strategy Exemplified with $Ru(bpy)_3^{2+}$ in the Annihilation Pathway. *Anal. Chem.* **2021**, *93*, 11626-11633.

[S13] S. Kumar, A. Kalkal, in *Nanotechnology in Cancer Management*, Elsevier, **2021**, pp. 43-71.
